# Image-based crystal detection: a machine-learning approach

**DOI:** 10.1107/S090744490802982X

**Published:** 2008-11-18

**Authors:** Roy Liu, Yoav Freund, Glen Spraggon

**Affiliations:** a University of California at San Diego, USA; b Genomics Institute of the Novartis Research Foundation, USA; c Joint Center for Structural Genomics, USA

**Keywords:** image analysis, machine learning, structural genomics, feature extraction

## Abstract

The ability of computers to learn from and annotate large databases of crystallization-trial images provides not only the ability to reduce the workload of crystallization studies, but also an opportunity to annotate crystallization trials as part of a framework for improving screening methods. Here, a system is presented that scores sets of images based on the likelihood of containing crystalline material as perceived by a machine-learning algorithm. The system can be incorporated into existing crystallization-analysis pipelines, whereby specialists examine images as they normally would with the exception that the images appear in rank order according to a simple real-valued score. Promising results are shown for 319 112 images associated with 150 structures solved by the Joint Center for Structural Genomics pipeline during the 2006–2007 year. Overall, the algorithm achieves a mean receiver opera­ting characteristic score of 0.919 and a 78% reduction in human effort per set when considering an absolute score cutoff for screening images, while incurring a loss of five out of 150 structures.

## Abstract 2

A system for scoring images based on the likelihood of containing crystalline material is described. A simulation carried out on a real set of crystallization images demonstrates the utility of such a system in high-throughput environments by substantially reducing the manual workload necessary to detect crystals for X-ray screening.

## 1. Introduction   

Recently, the use of robotics and parallel techniques for protein production and crystallization has become commonplace among structural genomics initiatives (Lesley *et al.*, 2002 ▶; DiDonato *et al.*, 2004 ▶; Lesley & Wilson, 2005 ▶; Chamberlain *et al.*, 2006 ▶) and within the general macromolecular crystallo­graphy community (Vincentelli *et al.*, 2003 ▶). Because of the parallel execution of protein expression and crystallization trials, structural genomics initiatives now provide 50% of all novel structures solved each year (Chandonia & Brenner, 2006 ▶). Despite the strides made in increasing physical trial throughput, the act of finding just a few crystals among potentially thousands of crystallization experiments still remains a task requiring human input. A number of processes (Spraggon *et al.*, 2002 ▶; Cumbaa & Jurisica, 2005 ▶; Kawabata, Saitoh *et al.*, 2006 ▶; Pan *et al.*, 2006 ▶; Watts *et al.*, 2008 ▶) that have achieved varying degrees of success have been proposed to accomplish this task. Whilst automating the crystal-detection part of such a pipeline may seem like a straightforward problem of recognizing the lines and textures indicative of crystals, devising an automated analyzer in practice proves challenging for two reasons. Firstly, computer vision is still a relatively young field. While many consider the detection of ubiquitous structured objects such as human faces (Viola & Jones, 2004 ▶) a well studied problem, detection of non-uniform objects such as crystals remains open and domain-specific. Secondly, the needle-in-a-haystack property of finding just a few crystals for diffraction analysis from among potentially thousands of trials necessitates that a system correctly rejects the vast majority of crystal-negative trials and that it rarely, if ever, rejects crystal-positive trials.

To learn from extracted features over sets of crystallization-trial images, we use the alternating decision-tree variant of boosting (Freund & Mason, 1999 ▶). Taken as a black-box learning algorithm, boosting has the same input–output interfaces as support vector machines (SVM; Pan *et al.*, 2006 ▶), linear discriminant analysis (LDA; Kawabata, Saitoh *et al.*, 2006 ▶) and neural networks (Spraggon *et al.*, 2002 ▶). We chose boosting over other techniques for its ability to automatically combine many marginally discriminative features into a single accurate ensemble classifier. The method has seen use in the bioinformatics community for its predictive capability (Mid­dendorf *et al.*, 2005 ▶); in our case, it serves the purpose of image analysis. Our choice seems timely in lieu of recent work on ensemble classification (Kawabata, Saitoh *et al.*, 2006 ▶; Walker *et al.*, 2007 ▶) that merges the outputs of disparate techniques into single classifications with hand-tuned rules. Consequently, we view boosting as a principled, automatic, theoretically motivated (Freund & Schapire, 1995 ▶) next step along these lines.

We report the scoring results of 319 112 crystallization trial images constituting the image sets of 150 structures solved by the Joint Center for Structural Genomics during the year 2006–2007. Our system achieves a mean receiver operating characteristic (ROC-AUC) score of 0.919 taken over the curves of individually scored image sets which represent a diverse array of families of novel proteins whose structures have hitherto not been determined. Simulations indicate that a huge saving in human effort can be achieved by searching, in rank order, for the first image of each set associated with a trial that will eventually yield an X-ray crystal structure. Alternatively, depending on an individual’s tolerance for missing a crystal, a hypothetical arbitrary cutoff can be assigned. It is shown that accepting only the top 20% ranked images of each set would have captured at least one image linked to a mounted and successfully diffracting crystal for 145 of the 150 sets. Our results suggest that computer-assisted analyses have the potential to augment existing image-based crystallization systems; ultimately, they may provide full annotation of trials and thus enhance our ability to automatically record crystallization results and derive optimal crystallization conditions for specific proteins.

## 2. Experimental procedures   

### 2.1. Protein crystallization   

All proteins were produced following protocols described in DiDonato *et al.* (2004 ▶), Lesley & Wilson (2005 ▶) and Chamberlain *et al.* (2006 ▶). Crystallization experiments were carried out using the sitting-drop vapour-diffusion method at 277 K in low-profile 96-well plates (Greiner) using sparse-matrix screens (Page *et al.*, 2003 ▶, 2005 ▶) on a Hydra Plus One (Krupka *et al.*, 2002 ▶) or Phoenix crystallization instrument (Art Robbins Instruments). The total drop size was 400 nl, using equal volumes of protein and crystallization reagent. Fine screens around promising conditions were generated *via* a RoboDesign Alchemist system with *CrystalTrak* software (RoboDesign Crystalmation System). Structures solved are detailed in Supplementary Data 1^1^ and have been deposited in the Protein Data Bank (http://www.rcsb.org).

### 2.2. Image acquisition   

**Figure 1:**
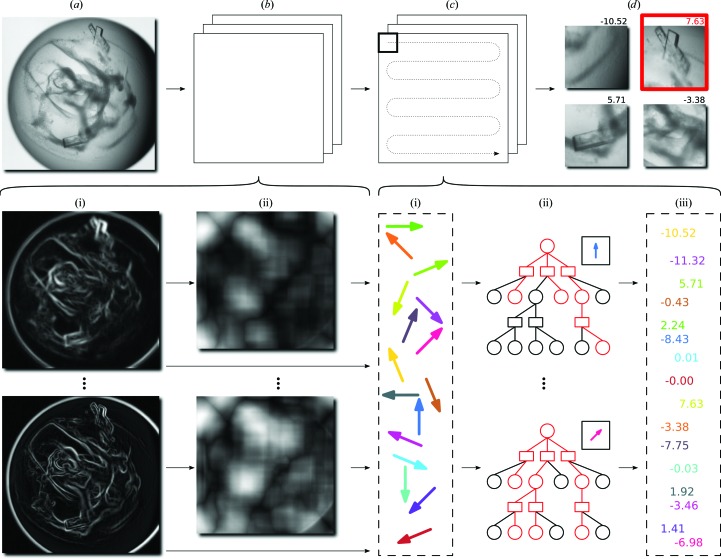
The scoring pipeline. (*a*) The original image. (*b*) An image stack obtained from image processing. (i) Heatmaps of Gabor responses. White areas represent pixels of high response. (ii) Heatmaps of orientation histograms. White areas represent square centers with high ‘largest bin value’ statistic. (*c*) Scanning the image and scoring each square. (i) Each square is associated with a feature vector encoding the values of 466 features. Each colored arrow is intended to represent a feature vector from one square subregion of the image. (ii) Each feature vector propagates differently through the alternating decision tree. (iii) A real-valued score is thereby associated with each feature vector. (*d*) The maximum score marked in red over all squares is taken as the image score.

Images were taken automatically using a custom imaging system (GNF Systems) integrated with an Optimag 1700 (Veeco) system equipped with a 5× magnification objective with fixed focus. Images have dimensions of 1024 × 1024 pixels and are eight-bit grayscale. They consist of a 2 × 2 mm shelf (Fig. 1 ▶ *a*) surrounded by a beveled edge that features prominently in every image.

### 2.3. Feature extraction   

The trained algorithm scores square image subregions of 127 × 127 pixels, as depicted in Fig. 1 ▶(*c*); the score for an entire image is the maximum over all square scores. This is not unlike previous work (Kawabata, Takahashi *et al.*, 2006 ▶; Pan *et al.*, 2006 ▶) that also avoids global heuristics in favor of accurate local classifiers. Feature extraction relies on Gabor wavelet responses to detect edges and textures (Pan *et al.*, 2006 ▶). Orientation histograms substitute for gray-level co-occurrence matrices (Spraggon *et al.*, 2002 ▶; Kawabata, Takahashi *et al.*, 2006 ▶) and attempt to capture morphological qualities.

The transformation of images into a computer-interpretable feature representation largely determines the kinds of concepts learned. We devise illumination/scale/orientation-invariant features that attempt to discriminate between lines and textures indicative of crystals and noncrystals. Furthermore, the use of convolution as the basis for all higher level calculations reduces *ad hoc* aspects of our design and consists of two conceptual stages.

In the first stage, we apply image processing to obtain an image stack as in Fig. 1 ▶(*b*): a data structure that, when queried for a given square, provides necessary and sufficient information for the derivation of a feature vector associated with that square. The majority of the stack arises from oriented Gabor magnitude calculations (Gabor, 1946 ▶; Lee, 1996 ▶). These calculations essentially perform image transformations from which features are calculated. The resulting set of derived features is then used by the machine-learning algorithm to discriminate crystal from noncrystal. Firstly, the original image of Fig. 1 ▶(*a*) is convolved with *n* = 6 orientations of a complex-valued Gabor filter determined by scale, frequency and elongation parameters. Taking complex magnitudes results in real-valued responses *G*_1_, …, *G*_*n*_. For each *S*_*i*_ subregion of the response *G*_*i*_, we calculate an aggregate Gabor response *S* using the formula
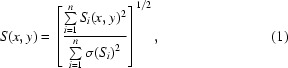
where σ(·) denotes the standard deviation over a matrix and *x* and *y* are two-dimensional coordinates. We also generate responses from gradient magnitude and non-oriented Gabor magnitude calculations in the same way, except that effectively *n* = 1. (i) in Fig. 1 ▶(*b*) demonstrates the above processes. For each Gabor and gradient magnitude response, we derive an orientation histogram that measures the distribution of gradients within any subregion; see (ii) in Fig. 1 ▶(*b*) for an illustration.

In the second stage, we scan over an image stack as in Fig. 1 ▶(*c*) and derive a feature vector from each square. Firstly, given a square response *S* as per (1), we threshold at 2^−*i*^ for *i* ∈ {1, …, 8}: the threshold value *T_v_* is the *v*th percentile value in the ascending sort of the values of *S*. Secondly, to complement straight thresholding, we take the delta, or total change, between pairs of thresholds. Thirdly, we take the standard deviation σ of *S* given by the denominator of (1) as a feature in itself. Fourthly and finally, we produce six statistics for each orientation histogram calculated at *S*: these are entropy, standard deviation of values divided by σ and the first, second, fourth and eighth largest bin values divided by σ (Table 1 ▶). In total, each feature vector is comprised of 466 features (Table 1 ▶). In the interests of brevity, we relegate a more detailed discussion of feature extraction to Supplementary Data 3^1^.

### 2.4. Training algorithm   

We use the alternating decision-tree variant of boosting (Freund & Mason, 1999 ▶) to learn classifiers that output real-valued scores, the signs of which represent the label and the magnitudes of which represent the confidence. Our training set consists of extracted feature vectors from 2659 images annotated with 21 477 squares, of which 10 823 and 10 654 are marked as crystal and noncrystal, respectively. We then perform a typical *n* = 8-fold cross-validation: we split the training set uniformly at random into *n* equal subsets, train using *n* − 1 subsets and test using the remaining one.

We summarize the averaged cross-validation performance in Table 2 ▶. Results of this type hint towards how the algorithm might score entire images; they do not exemplify experimental rigor, as many training squares overlap and might appear in both the training and test sets of a fold. After computing all eight folds, we settle on one of eight alternating decision trees emitted as a side effect for use in our main simulations. Although ideally the choice is arbitrary for a large number of training examples, we choose the tree with the highest ROC-AUC score for positive examples. To enable the design and testing of classifiers in a tight workflow loop, our system automatically generates ROC, precision-recall, test-set accuracy and boosting margin performance metrics for each fold.

### 2.5. Scoring the images   

The *JBoost* package (http://jboost.sourceforge.net) is used for learning alternating decision trees and the *Shared Scientific Toolbox in Java* (http://shared.sourceforge.net) and *FFTW* (Frigo & Johnson, 2005 ▶) are used for image processing and data analysis. A MySQL database stores scoring-data structures. The main simulation on 319 112 images took 67 h to run on 128 dual-core 1.6 GHz AMD Operator nodes of the UCSD FWGrid service (http://fwgrid.ucsd.edu/). This amounts to an amortized 97 s per image of size 1024 × 1024 pixels. With current technology trends, however, we estimate that sets of size 1536 in 6 h are surely feasible within a moderate budget. To provide a basis of comparison for future image-analysis systems with ours and with each other, all images along with human and computer scores are publicly available.

Since our system consists entirely of free open-source components, runs on commodity computer hardware and uses an illumination/scale/orientation-invariant feature representation, we envision that users can run it ‘off the shelf’ and observe noticeably better than random rank orderings. To enable the adaptation of the underlying boosting algorithm to laboratory-specific images, we offer a dedicated graphical user interface for visualizing and editing training annotations. We integrate the program into the database and workflow, so that a suboptimal classifier flags potentially crystal-positive images for subsequent validation and annotation by a human being; this has the effect of helping the user to generate quickly many training examples from which improved next-generation classifiers can bootstrap.

## 3. Results and discussion   

### 3.1. Image-scoring setup   

We selected 150 sets of crystallization trials for analysis by the system. Each set typically consists of 1536 images accumulated over a four-week period at 3, 7, 14 and 28 d for coarse screens (four sparse-matrix plates; Page *et al.*, 2005 ▶) and a variable number of fine-screen images (two-dimensional optimization of coarse-screen hits). Trial images were annotated as ‘Harvestable’ if they contained mountable crystals (usually with size >10 µm) and ‘Crystal Hit’ if they contained crystalline material deemed not suitable for mounting. For our simulation, we ignored the Crystal Hit annotation and focused on trials marked as Harvestable, which we refer to as diffraction candidates.

Among all images, specialists marked 11 934 as diffraction candidates, which on average account for a small 0.038 ± 0.012 (s.d.) fraction of each set. We refer to all other images as discarded trials. Of the diffraction-candidate images, 414 contained crystals that yielded X-ray structures; we refer to these as diffraction successes. We note that each of the 150 image sets has an associated structure and by extension at least one diffraction success; multiple diffraction-success images arise as a result of imaging the same underlying trial well over time. In some cases images are taken from a well after the crystals have been harvested. Images of this type are generally characterized by an unfocused translucent layer superimposed on top of the image.

**Figure 2:**
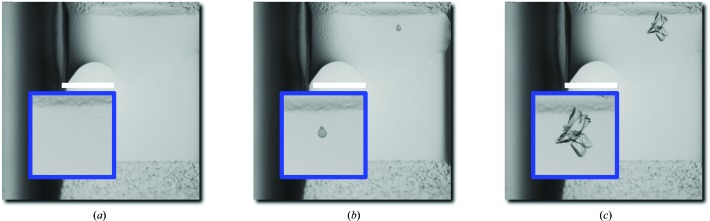
An illustration of how machine-learning scores assigned to images taken over different time periods of the same well increase over time. (*a*) A score of 0.15 at 7 d; (*b*) a score of 6.21 at 14 d; (*c*) a score of 9.01 at 28 d.

If crystals grow between image-acquisition periods, we would expect an increase in image scores with time over the same underlying well. To compute statistics, we computed the difference in score between the earliest image and the image immediately prior to harvesting; wells imaged two or fewer times prior to harvesting were ignored. In all, 1480 out of 2158 or 68.6% of diffraction-candidate wells registered a score increase over time. Fig. 2 ▶ illustrates an exemplar well where this was true.

Most of our training images originate from the previous year. Because of carry-over effects, an intersection with seven image sets accounts for 4497 annotation squares or 21% of the total image squares used for training. To ensure that the algorithm never scores an image it was trained on, images used for training were excluded from the images (563 in total) used for testing. Whereas we personally curated the training-image set as a representative collection of learning cases, we did not have any hand in defining the ground truth for test sets, which consisted of annotations by crystal-analysis experts who adjudicated crystallization-trial images from prior experience as a routine step of the normal operation of the JCSG pipeline. Since we train and validate on physically and temporally disjoint image sets, our algorithm should generalize to types of crystals never seen before.

### 3.2. Image-scoring results   

The definition of what constitutes crystalline material is highly subjective; it varies even among human annotators. For the purpose of evaluating our system, we consider trials marked by humans for X-ray diffraction analysis, a harvestable set, as true positives for the purposes of ROC analysis and refer to them as ‘diffraction candidates’. In addition to measuring how well our system scores putative crystals, we also explore its effectiveness as a pipeline optimization for identifying those that would eventually yield a crystal structure, which we refer to as ‘diffraction successes’. Whereas previous work mainly studies the imaging problem as a series of machine-learning experiments with ground truth selected by the experimenters, we frame it in terms of end performance as measured by the number of structures solved from images assigned with a high score.

**Figure 3:**
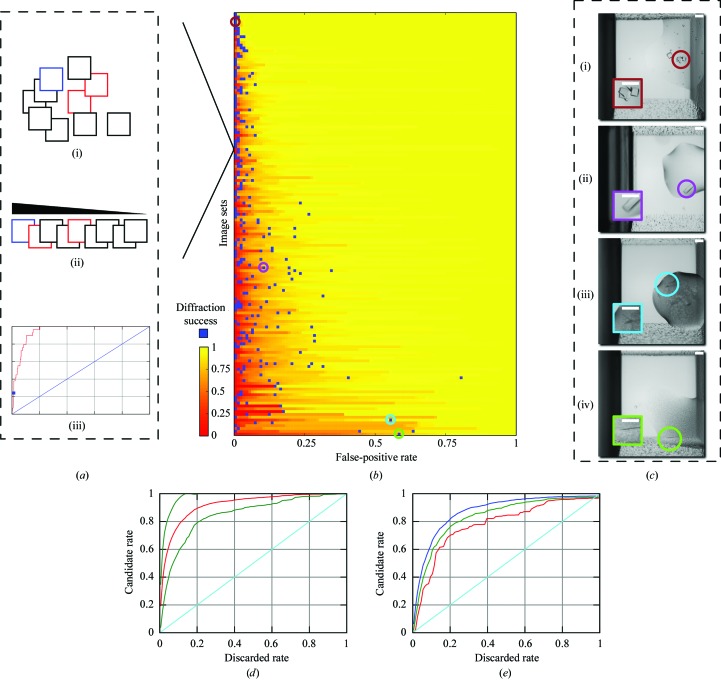
A summary of the main experimental results. (*a*) Scoring and performance evaluation of an image set: (i) images start out unscored with human annotations; (ii) the algorithm scores the set, inducing a rank ordering on it; (iii) an ROC curve is derived from scores and ground truth. (*b*) The ROC heatmap, a simultaneous view of all individual ROC curves. For the purposes of ROC analysis, we treat diffraction candidates as true-positive examples and discarded trials as false-positive examples. Rows delineate individual curves ordered from top to bottom in descending order of ROC-AUC score. The intensity values of the heatmap represent true positive rates, with an overlay marking the location of images containing the diffraction success in blue. (*c*) Diffraction successes and their images: representative rectangles are shaded in the same color in (*b*) and (*c*) to show position in the heatmap. (i) Crystals of an XisH-family protein from *Nostoc punctiforme* PCC 73102 at 1.60 Å resolution (PDB code 2inb). (ii) Crystals of *Bacillus cereus* ATCC 10987 at 2.10 Å resolution (PDB code 2p1a). (iii) Crystals of methyltransferase FkbM from *Methylobacillus flagellatus* KT at 2.20 Å resolution (PDB code 2py6). Much of the crystal contours are occluded by precipitant. (iv) Crystals of HD superfamily hydrolase from uncultured *Thermotogales* bacterium at 1.45 Å resolution (PDB code 2pq7). Aside from a telltale line, the rest of the crystal contours are barely visible. (*d*) An ‘average’ ROC curve (red line) with upper and lower standard deviation bands (green lines). (*e*) A ‘worst-case’ ROC curve for various confidences *p* (red for *p* = 0.05, green for *p* = 0.10, blue for *p* = 0.20).

We convey all singleton ROC curves with a compact visual representation in Fig. 3 ▶(*b*). To summarize a large number of results, we offer a variety of aggregate statistics that, in combination, attempt to capture the performance characteristics of our scoring-based system. While not always an appropriate surrogate for direct inspection of individual curves (Supplementary Data 2^1^), such statistics can demonstrate the consistency of a system and offer a basis of comparison to other systems. Additionally, the novel aspects of our derivations may facilitate the measurement of current and future high-throughput image-analysis pipelines.

Our system achieves a mean ROC-AUC score of 0.919 taken over the curves of individually scored image sets. To complement a single all-encompassing number, we include two aggregate ROC curves that summarize the expected and worst-case scoring capability of our system. Firstly, in Fig. 3 ▶(*d*), we take the mean over all sets of the true positive diffraction-candidate rate (TPR) for each fixed false-positive discarded trial rate (FPR). This kind of curve reveals the expected TPR as a function of the FPR. For example, upon encountering 20% of all discarded trials, one can expect to have seen 92% of all diffraction candidates. Secondly, in Fig. 3 ▶(*e*), we interpret the TPRs over all sets as samples drawn from a probability distribution for each fixed FPR. We then calibrate a maximum achievable TPR for differing levels of confidence: an estimate of the probabilistic worst-case TPR as a function of the FPR. For example, upon encountering 20% of all discarded trials, one may expect with 95% confidence to have seen 71% of all diffraction candidates. Note that in the analyses above the discarded-trial rate closely tracks the rate of all images, because of the assumed rarity of crystal-positive trials. Thus, one can reasonably attribute a cost saving of *s* to a cutoff rate of 1 − *s*.

**Figure 4:**
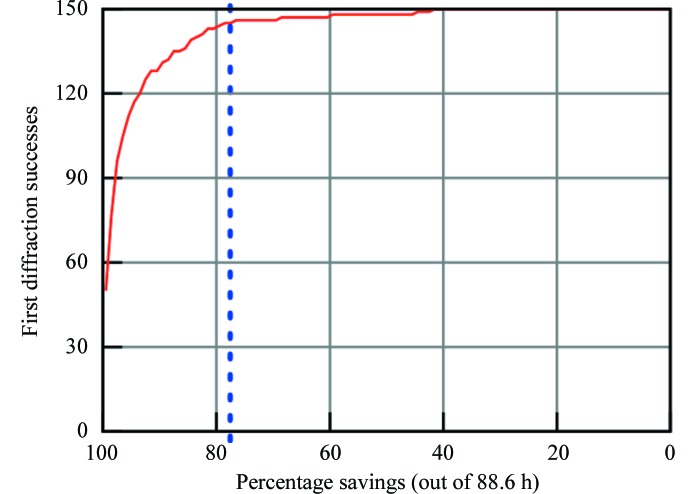
A graph derived from the number of solved structures associated with encountered images as a function of the estimated total human annotation time of 88 h. The blue dashed line represents the cutoff chosen in §[Sec sec3.2]3.2.

In addition to the ROC analyses above, we simulate the retrieval capability of our system to derive the same results as those carried out manually in terms of structures solved. We consider diffraction successes, the blue squares in Fig. 3 ▶(*b*), as images of interest: they contain crystals that eventually yielded X-ray structures. Note that an image set may contain multiple diffraction-success images which represent snapshots of the same underlying well over time. We calculate the average discarded-trial rate before finding the first diffraction success, determined in rank order, to be 4.5% (over all image sets), which translates into an expected saving of 95% per set. In other words, a specialist can expect to examine 4.5% of an image set before encountering a trial that would have successfully diffracted and yielded a structure, implying an expected saving of roughly 95% in human effort. Put into real terms, if one image takes 1 s to manually analyze, a set of size 1536 images containing at least one diffraction-quality crystal would require, on average, 93 s of inspection before finding said crystal. We also include a continuum of time–quality tradeoffs in Fig. 4 ▶ and calculate the number of first diffraction successes retrieved as a function of the time spent in analysis. We predicate our measurements on the simplifying assumption that each image takes 1 s to analyze and that each fractional second is spent ‘fairly’ among all image sets so they advance uniformly toward completion on a percentage basis (larger sets receive proportionately more analysis units).

Since the above metric is not a probabilistic proposition, the failure of our algorithm would simply mean more analysis work and not a loss of crystals. Alternatively, we could imagine the policy of applying a hard cutoff: for each set, a specialist peruses no more than *x* in rank order. Each image typically takes approximately 1 s to manually inspect, making the time to inspect the entire set of images approximately 88 h. Choosing to spend 20 h (out of a total of 88 h) of analysis effort, for example, realises a 78% cost saving and implies a cutoff of considering only the top 22% of each image set (Fig. 4 ▶); the system would have retrieved 145 out of 150 first diffraction successes and failed on five sets; in other words, those images from which crystals were harvested and diffracted successfully. Note that realising a 46% saving would have captured at least one diffraction success yielding a structure from every one of the 150 sets; one could expect to save this much under a zero-tolerance policy for misses.

To complement our quantitative results, we include diffraction successes associated with four solved structures in Fig. 3 ▶(*c*), as well as each set’s ROC curve and its top-ranked diffraction success (Supplementary Data 2^1^). An initial qualitative inspection suggests that human annotators would also have some difficulty identifying crystals in images with low scores assigned by the algorithm.

### 3.3. Correlation of computational scores to diffraction success   

**Figure 5:**
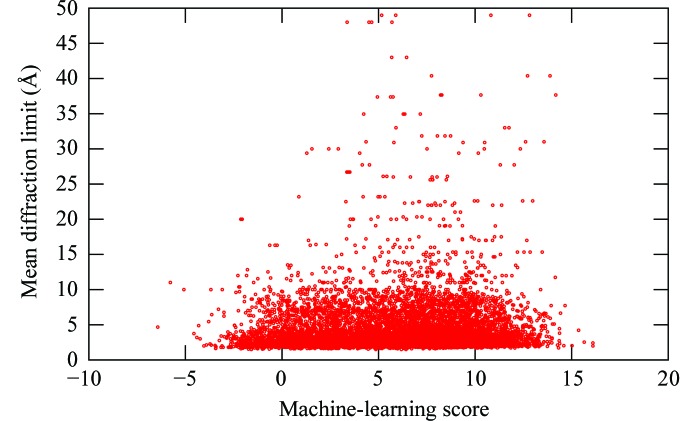
A scatterplot of crystal mean resolution *versus* machine-learning score from the image, taken from all harvestable crystals in the set that had measured data.

To ascertain whether the overall scores for images output by our machine-learning algorithm have any bearing on the microscopic qualities of crystals by way of their ability to diffract, we calculated a simple correlation between the scores and the diffraction limit of crystals harvested from a drop. We first filtered the JCSG database for crystals that have a detectable diffraction limit and ignored those that were salt crystals or had no measureable diffraction limits. For the purposes of analysis, we considered the 8751 images associated with wells in which these crystals were grown. Since only one score was produced per image and multiple crystals could be harvested from a drop, we then calculated the mean diffraction limit from each well, making no attempt to correct for factors such as retardation of diffraction resolution owing to ice or bad cooling of crystals or the effects of adding cryoconditions. Finally, we correlated diffraction limits and computational scores with the standard Pearson product–moment correlation coefficient (Duda *et al.*, 2001 ▶). For all images that produced solved structures, we calculated a value of 0.06 which, barring rounding effects, was constant when repeating the above calculation exclusively over images associated with coarse and fine screens, respectively. Consequently, the scatter plot in Fig. 5 ▶ suggests that no linear correlation exists between visual scores and diffraction scores, even though one might intuitively expect a negative correlation if the scoring algorithm has a linear response.

### 3.4. Quantitative study of fine screens *versus* coarse screens   

Of the structures solved in the period studied, 74% came from the standard coarse screens whilst 26% came from fine screens derived from these hits. Coarse-screen harvested crystals are invariably screened for diffraction prior to fine-screen hits, which may account for this trend, but in general it is found that whilst fine screens lead to many more diffraction-quality crystals of the same form, the diversity of crystal forms provided by coarse screens often provides adequate crystals to complete the structure before the need for fine screening. As an analysis, we consider the performance of the algorithm for coarse screens and fine screens separately, as image analysis might consider the trials of the former category before making a decision on whether to proceed with trials of the latter category. To run coarse-screen and fine-screen only experiments, we repeated all of the above procedures with the exception that we only considered trials corresponding to coarse and fine screens, respectively. We summarize our results in Table 3 ▶. Broadly speaking, the coarse-screen experiment achieved a mean ROC-AUC score of 0.930, while the fine-screen experiment achieved a mean score of 0.873. One might intuitively think that the fine-screen experiment would give a higher mean score; however, the results are explicable by the relative abundance of crystals in fine screens (8.5% as opposed to 1.8% among coarse screens). Given our absolute notion of ground truth and the inherently subjectivity of visual crystal quality, one would expect that with an abundance of crystalline material the system confuses false-positive ‘almost’ diffraction successes with true-positive diffraction successes. Under one interpretation, our system performs with respect to ground truth very much how two human annotators would perform with respect to each other: agreement is high when crystals are rare and lower when crystals are abundant.

## 4. Conclusion   

We offer a novel and generally applicable system whose requirements are well within the computational resources of most laboratories capable of generating large sets of crystallization-trial images. Our choice of well understood image-processing techniques and boosting as the core learning algorithm enables the amalgamation of hundreds of marginally discriminative features into a single accurate classifier. In addition, our measurement methods enable crystallographers to evaluate the system at varying levels of detail from individual ROC curves to aggregate ROC curves and under varying interpretations of performance.

A byproduct of our system is that it has the potential to address the often-asked question of whether visual crystal quality, as derived from a machine-learning algorithm, corresponds to physical crystal quality, as derived from X-ray diffraction pattern analysis. In other words, does the external regularity captured in the images and characterized by strong edges, symmetry and polygonal shapes correlate with microscopic regularity characterized by a molecular lattice structure? The ROC analysis of Fig. 3 ▶(*f*) strongly indicates that appropriate choices of scoring cutoffs lead to relatively few false negatives in the task of computationally identifying crystals. Given that our results imply no linear correlation between the learning-algorithm score and the diffraction limit, this reinforces the intuitive notion that features derived from the learning algorithm are not a good indicator of crystal diffraction quality. The negative results above do not preclude our system from being of use to high-throughput pipelines, where the identification of crystal candidates constitutes the main challenge.

Clearly, in our current analysis the simulations only take into account images that yield crystals capable of harvesting and result in a large reduction of annotation time with an arbitrarily small reduction in structures solved. In most cases these losses would be accounted for, as redundancy within a mounted crystal set for a particular target could still lead to solution of the structure. However, for cases where mountable crystals are very rare, missed crystals are unacceptable, but even in a zero-tolerance mode approximately 50% of image-analysis time can be saved (Fig. 4 ▶). For pipelines such as the JCSG which deals with millions of images a year, this can lead to a substantial saving in manpower.

The current body of work does not take into account those images that are annotated as crystalline but are used as starting conditions to further optimize crystals. The incorporation and use of this information in the structural genomics pipeline is the subject of ongoing work. As a further extension to this work, it is envisioned that one could annotate image sets as part of an effort to map the crystal phase space (Hansen *et al.*, 2004 ▶) of a specific protein and thus derive more efficient fine screens. Additional applications could include using steadiness over time of machine-learning scores of a well as indication that a crystal has reached its full growth potential and is ready for harvesting (Fig. 2 ▶).

As high-throughput methods become the norm rather than the exception, crystallographers are likely to face bottlenecks where physical experimental throughput outgrows the image-analysis capacity of a handful of specialists. In anticipation of this trend, we offer a complete system for augmenting current image-analysis pipelines that rank-orders images based on the likelihood of containing crystalline material. Thus, users of our system can achieve a reduction in effort as large as their tolerance for missing potential crystal structures.

## 5. Supplementary data^1^   

Supplementary Data 1^1^ contains details on deposited structures used within this study. ROC curve calculations for each set are contained within Supplementary Data 2^1^, whilst further information on software installation, obtaining image sets, preprocessing images, running the system as a distributed computation, interpreting cross-validation performance metrics and annotating images with our purpose-built user interface is contained within Supplementary Data 3^1^. All images associated with this study can be found at http://www.jcsg.org.

## Supplementary Material

Supplementary Data 1. DOI: 10.1107/S090744490802982X/yt5007sup1.pdf

Supplementary Data 2. DOI: 10.1107/S090744490802982X/yt5007sup2.pdf

Supplementary Data 3. DOI: 10.1107/S090744490802982X/yt5007sup3.pdf

## Figures and Tables

**Table 1:** An overview of the feature schema used for learning

Type	Variants	Features	Total
Oriented Gabor	9	25	225
Gradient magnitude	1	25	25
Non-oriented Gabor	6	25	150
Original image	1	0	0
Integral histograms	9 + 1 + 1	6	66
			466

**Table 2:** Cross-validation performance over eight folds and 160 rounds of boosting**

Square annotation	All	Positive	Negative
Mean test-set size	2684	1353	1331
Mean train-set size	18793	9470	9323
Test-set error (%)	6.6	6.2	7.0
Mean ROC-AUC score	N/A	0.856	0.905

**Table 3:** A summary of coarse and fine-screen experiments A retained set is one that includes at least one true-positive (diffraction-candidate) trial.

Type	Mean ROC-AUC	Retained sets	True/total
Coarse	0.930	147	4125/225574
Fine	0.873	55	7809/92098

## Footnotes

1 Supplementary material has been deposited in the IUCr electronic archive (Reference: YT5007). Services for accessing this material are described at the back of the journal.
