# Supplementary material for: Image-based crystal detection: a machine-learning approach
Source: Acta Crystallogr D Biol Crystallogr. 2008 Nov 18;64(Pt 12):1187–95. doi: 10.1107/S090744490802982X (PMC2585161; doi:10.1107/S090744490802982X)
Supplement: Supplementary file 1 [file d-64-01187-sup1.pdf]

Structures Solved

Target Name	PDB id	Best Res	R Factor	R Free	Space Group
FG7288A	2HX1	2.1	0.18	0.23	P222
TM0015	2RAA	2.12	0.22	0.25	
10173191	2HTI	2.5	0.21	0.26	P622
10175341	2OA2	1.41	0.17	0.19	
16420133	2I6G	1.9	0.19	0.23	P222
CM5490D	2PFX	1.7	0.11	0.17	P6
CM7979A	2QDR	2.6	0.24	0.29	P622
CM8004D	2PY6	2.15	0.17	0.22	I222
CM8073A	2O62	1.75	0.17	0.21	P222
CM8385A	2OOC	1.52	0.17	0.2	P3
FB10608A	2QCK	1.9	0.2	0.25	P622
FB7482B	2QEA	2.46	0.23	0.28	P622
FG7255A	2P1A	2.1	0.22	0.27	P222
FG7273A	2P11	2.2	0.2	0.25	C2
FG7279A	2HQ7	2	0.19	0.24	C2
FG7283A	2P4G	2.3	0.17	0.23	P6
FG7292A	2HCF	1.8	0.19	0.25	P222
FG7298A	2Q30	1.94	0.17	0.21	P2
FG7303A	2OZJ	1.6	0.18	0.21	C222
FG7328A	2NUJ	2	0.16	0.19	P422
FG7335A	2HI0	1.51	0.16	0.19	P2
FG7343A	2OWN	2	0.2	0.24	P622
FG7355A	2O2X	1.5	0.14	0.17	P2
FG7396A	2HX5	1.5	0.16	0.18	I23
FG7402A	2HLJ	2	0.17	0.23	P222
FG7406A	2IFX	2	0.17	0.2	P4
FG7419A	2IAB	2	0.18	0.23	P622
FG7459A	2HHZ	2	0.19	0.23	P422
FG7480A	2ILB	1.6	0.18	0.23	C2
FH7484A	2I51	1.4	0.17	0.19	P222
FH7486A	2I02	1.8	0.18	0.2	P622
FH7577A	2HSB	1.95	0.18	0.21	P422
FH7599A	2PNK	2	0.15	0.18	C2
FH7614A	2IG6	1.8	0.17	0.2	P222
FH7681A	2HTD	1.6	0.16	0.21	P21
FH7684A	2HUJ	1.74	0.17	0.21	P622
FH7700A	2HQ9	1.95	0.18	0.24	P222
FJ1648A	2OGI	1.85	0.17	0.23	P2
FJ5490A	2OUW	1.95	0.2	0.23	P3
FJ5490C	2PRR	2.15	0.21	0.26	P1
FJ8586A	2OZG	2	0.16	0.22	H32
FJ8786A	2OC5	1.68	0.16	0.2	P422

FJ8809A	2OAF	2	0.17	0.19	P622
FJ8827A	2O1Q	1.5	0.18	0.21	P422
FJ8832A	2QEU	1.65	0.15	0.18	P622
FJ8835A	2ONF	1.7	0.16	0.2	P2
FJ8838A	2PR7	1.44	0.17	0.2	P1
FJ8839A	2PN2	1.95	0.2	0.24	C222
FJ8891A	2OBP	1.7	0.19	0.22	P622
FJ8994A	2O8Q	1.55	0.17	0.19	P422
FJ9081A	2P2S	1.25	0.16	0.18	
FJ9092A	2PN1	2	0.19	0.24	C2
FJ9219A	2O2G	1.92	0.18	0.24	P222
FJ9230A	2O7T	2.1	0.18	0.23	P321
FJ9248A	2Q04	2.33	0.23	0.26	P2
FJ9319A	2QEC	1.9	0.19	0.24	P622
FJ9329A	2P97	1.65	0.16	0.19	P422
FJ9406A	2OU6	1.8	0.16	0.19	P622
FJ9446A	2OU5	1.6	0.18	0.22	P222
FJ9503A	2Q0T	1.7	0.15	0.19	P2
FJ9519A	2OH3	2	0.19	0.24	I222
FJ9546A	2Q7B	2	0.19	0.22	P321
FJ9548A	2PIM	2.2	0.18	0.22	P622
FK4030A	2P7H	1.85	0.15	0.18	P422
FK5490E	2OYO	1.51	0.18	0.22	
FK5749A	2PKE	1.81	0.18	0.23	P2
FK8795C	2OZH	1.4	0.14	0.16	I23
FK8798A	2QWW	2.07	0.24	0.29	
FK8801A	2Q0Y	1.8	0.14	0.17	P321
FK8817C	2OPK	2.1	0.17	0.21	P422
FK9098B	2PG3	2.4	0.19	0.22	P422
FK9279A	2Q02	2.4	0.18	0.23	P222
FK9414A	2OPL	1.5	0.15	0.17	P222
FK9428A	2OIK	1.65	0.18	0.22	P1
FK9436A	2OH1	1.46	0.17	0.19	
FK9452E	2OTM	1.85	0.15	0.18	C121
FK9665C	2PRX	1.5	0.2	0.28	P3
FK9742A	2PC1	1.28	0.17	0.18	P1
FK9784A	2PFW	1.9	0.19	0.26	P222
FL10929A	2QE8	1.35	0.12	0.14	P222
FL11009A	2QIW	1.8	0.14	0.17	P222
FL1347A	2QR6	1.5	0.17	0.19	I4
GN7730A	2OP5	2.2	0.23	0.29	P222
GN7738A	2OD4	1.7	0.16	0.19	P2
GN7747A	2OD5	1.79	0.19	0.21	P622
GN7757A	2OD6	1.85	0.2	0.23	P222

GN7773A	2PGC	2.53	0.21	0.25	P222
HP10645A	2QVP	2	0.16	0.21	P2
HP1666A	2RB7	1.6	0.15	0.18	P2
HP9625C	2QYV	2.11	0.22	0.24	P21212
ME9797A	2PQ7	1.45	0.18	0.19	C121
NP_663012.1	2HR2	2.54	0.2	0.23	P222
NP_811092.1	2HUH	1.54	0.17	0.19	P422
NP_813429.1	2OKC	2.2	0.18	0.23	P222
NP_841447.1	2ICH	2	0.18	0.23	P222
PC02830C	2OSD	2.3	0.22	0.24	P422
PC05163A	2HKV	1.7	0.18	0.23	P3
PC05870B	2P8G	1.36	0.13	0.15	P4132
PC06175E	2ITB	2.05	0.17	0.23	P222
PC06249B	2PYT	1.9	0.17	0.22	P1
PC06304A	2O3L	2.05	0.18	0.23	P321
PC06304B	2O4T	1.95	0.2	0.26	H32
PC07755A	2OBN	2.3	0.18	0.21	P2
PD01933B	2O2Z	2.6	0.19	0.22	P222
PD01933E	2PPV	2	0.17	0.21	C222
PD04303G	2H9F	1.95	0.17	0.2	I4
PD06751F	2QEZ	2.15	0.23	0.28	P222
PD07049G	2OU3	1.85	0.16	0.21	P222
PE00002B	2O08	1.9	0.17	0.22	C222
PE00003C	2HYT	1.64	0.16	0.2	P321
PE00012A	2PRV	1.3	0.14	0.18	P1
PE00012N	2ICG	1.65	0.2	0.24	P622
PE00025A	2OC6	1.75	0.17	0.22	P2
PE00025C	2I8D	1.69	0.17	0.2	P222
PE00035A	2OKF	1.6	0.14	0.16	P3
PE00035B	2NLV	1.3	0.18	0.2	P121
PE00035C	2INB	1.6	0.16	0.19	P321
PE00037D	2IT9	1.8	0.18	0.21	P222
PE00037E	2NVN	2.5	0.21	0.23	P422
PE00044A	2Q22	2.11	0.19	0.22	P3
PE00055D	2IAY	1.2	0.12	0.15	P222
PE00057A	2NVM	2.19	0.2	0.24	P6122
PE00057B	2NWV	1.85	0.19	0.23	P622
PE00092A	2NYH	1.7	0.18	0.22	C222
PE00092D	2PEB	1.46	0.19	0.21	
PE00139F	2IA7	1.44	0.17	0.2	P222
PE00238G	2OQM	1.83	0.16	0.2	
PE00293D	2P10	2.15	0.16	0.2	
PE00389E	2I9W	1.75	0.19	0.24	P222
PG8359F	2OWP	2	0.19	0.24	P321

PG9822C	2Q3L	2.25	0.18	0.24	C222
PG9822D	2OOK	1.8	0.18	0.23	C121
PG9878A	2OOJ	1.84	0.18	0.21	P222
PG9905A	2P8J	2	0.17	0.2	P321
PG9920A	2QJW	1.35	0.18	0.22	P2
PG9933A	2QS7	2.09	0.18	0.2	H3
PG9969A	2Q03	1.8	0.17	0.18	P422
PH10062D	2PYQ	1.5	0.18	0.2	P2
PH10070D	2PV4	1.95	0.21	0.25	P222
PH10071A	2PG4	2.21	0.2	0.26	P422
PJ02661S	2QC0	1.6	0.17	0.2	P222
PJ04222C	2Q9R	1.91	0.17	0.22	C222
PJ04672A	2QE6	1.95	0.17	0.21	P222
PJ04820A	2PYX	1.5	0.15	0.18	P222
PJ05013D	2Q7S	2	0.2	0.25	P222
PJ05163B	2QE9	1.9	0.19	0.23	P422
PJ07336D	2PW4	1.45	0.14	0.15	C2
RK10652A	2QCV	1.9	0.18	0.2	P622
RK10654A	2QHP	1.8	0.17	0.22	C222
2635576	2Q83	2.5	0.2	0.21	P622
