# Supplementary material for: Image-based crystal detection: a machine-learning approach
Source: Acta Crystallogr D Biol Crystallogr. 2008 Nov 18;64(Pt 12):1187–95. doi: 10.1107/S090744490802982X (PMC2585161; doi:10.1107/S090744490802982X)
Supplement: Supplementary file 2 [file d-64-01187-sup2.pdf]

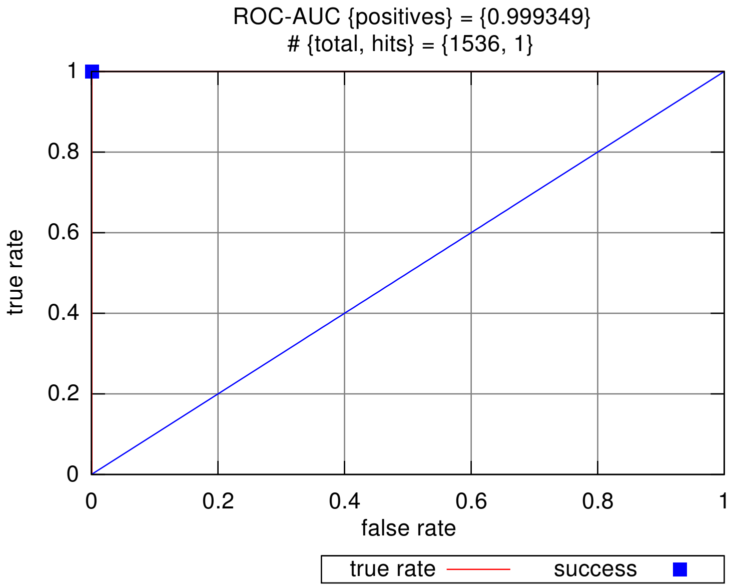

(a)

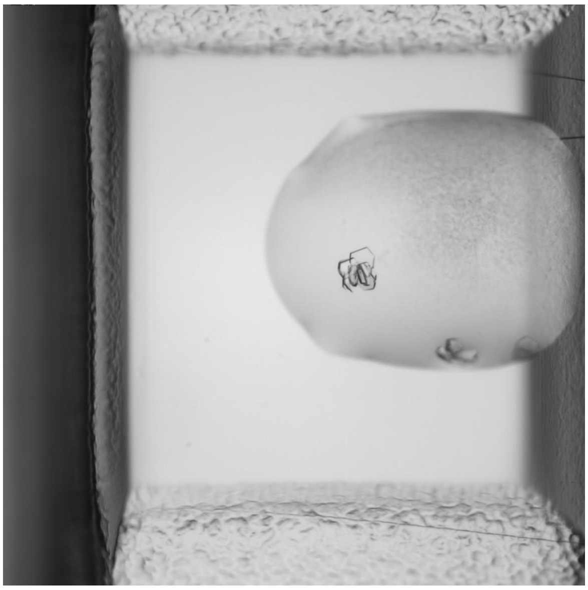

(b)

Figure 1: An ROC Curve for set ‘SpeedET-FG7292A-ctp-233-1-233’ along with its highest ranked diffraction success.

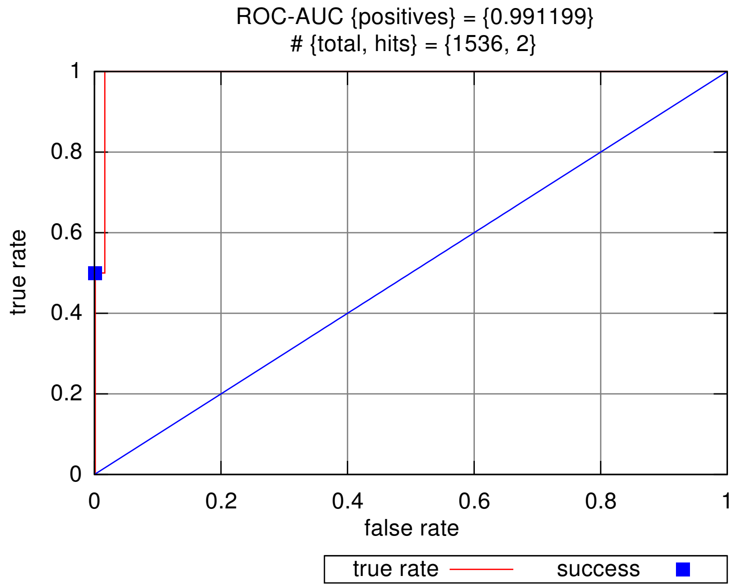

(a)

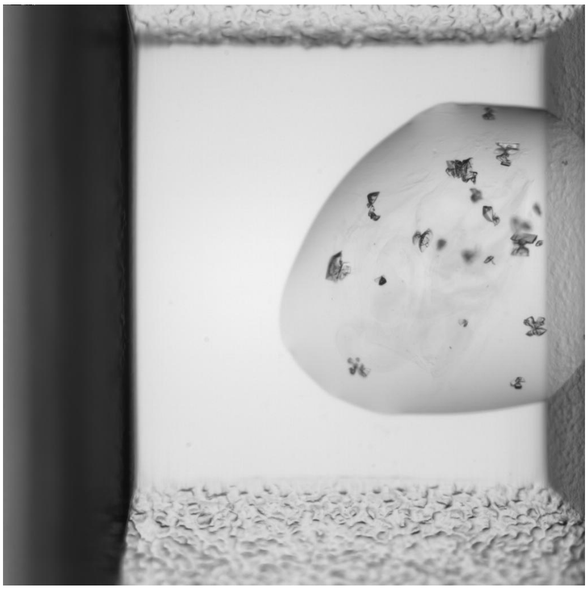

(b)

Figure 2: An ROC Curve for set 'SpeedET-FG7480A-xca-113-1-113' along with its highest ranked diffraction success.

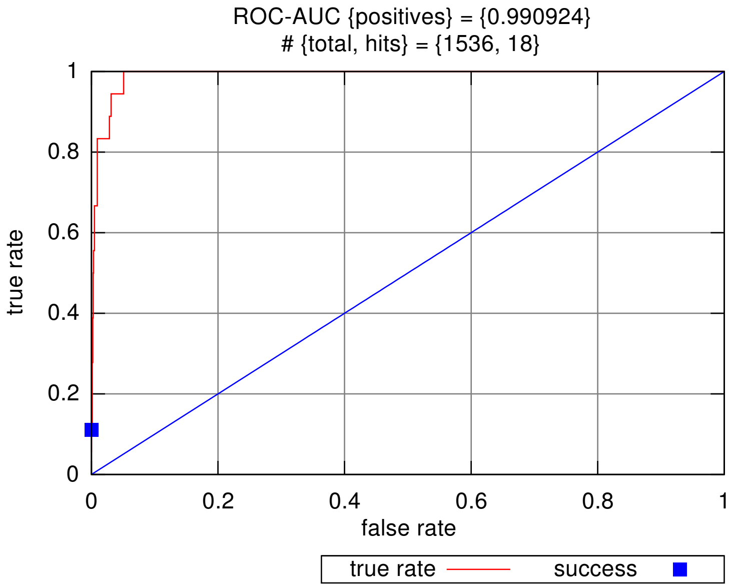

(a)

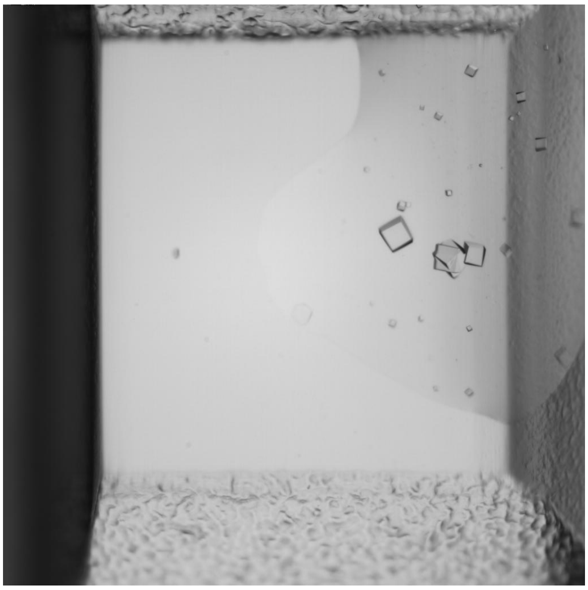

(b)

Figure 3: An ROC Curve for set 'SpeedET-PE00035C-npu-139-1-139' along with its highest ranked diffraction success.

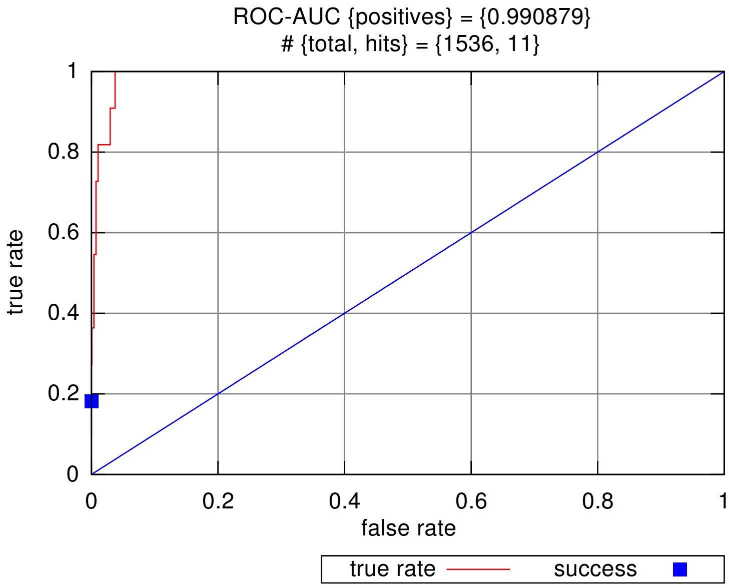

(a)

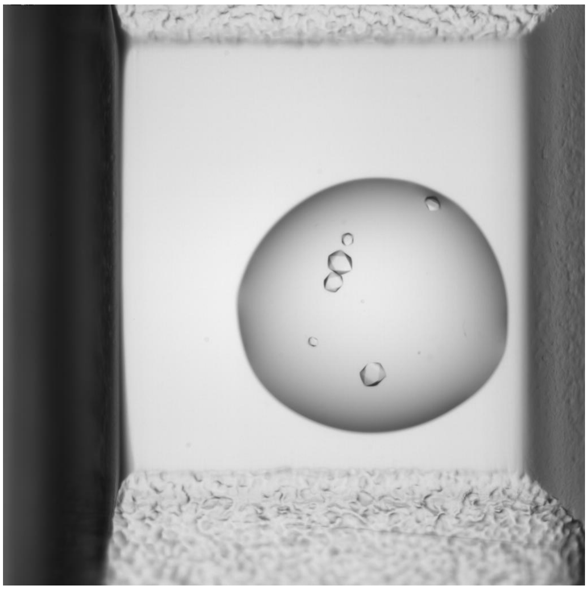

(b)

Figure 4: An ROC Curve for set ‘SpeedET-PD04303G-pae-395-1-395’ along with its highest ranked diffraction success.

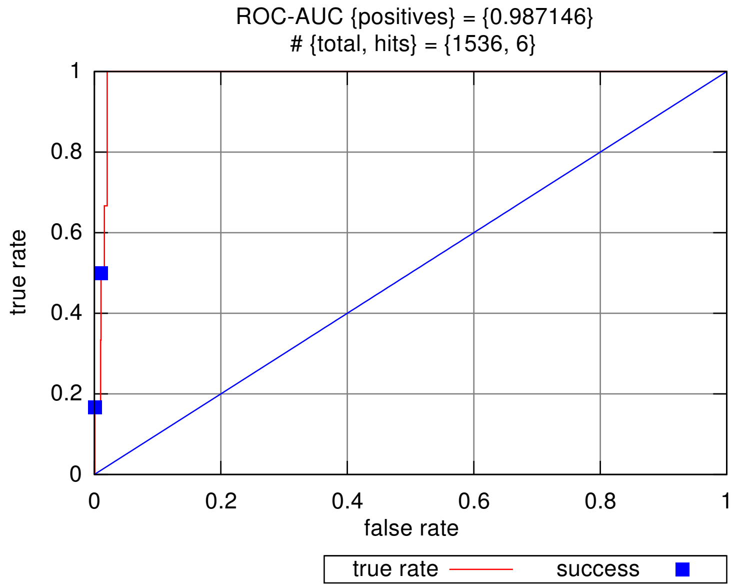

(a)

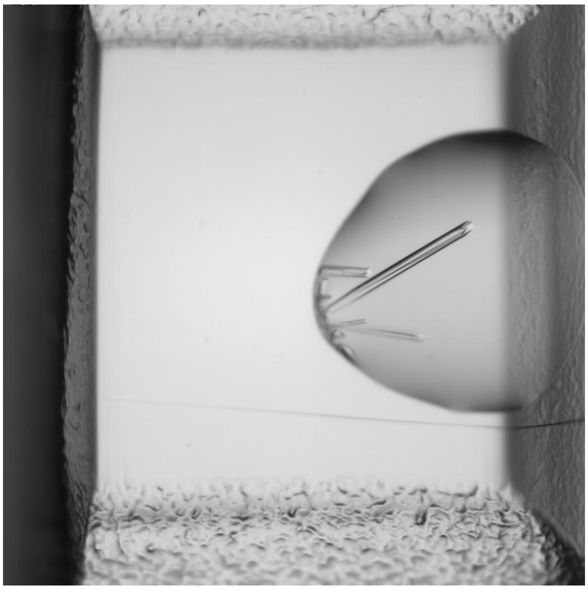

(b)

Figure 5: An ROC Curve for set 'SpeedET-PE00389E-par-183-1-183' along with its highest ranked diffraction success.

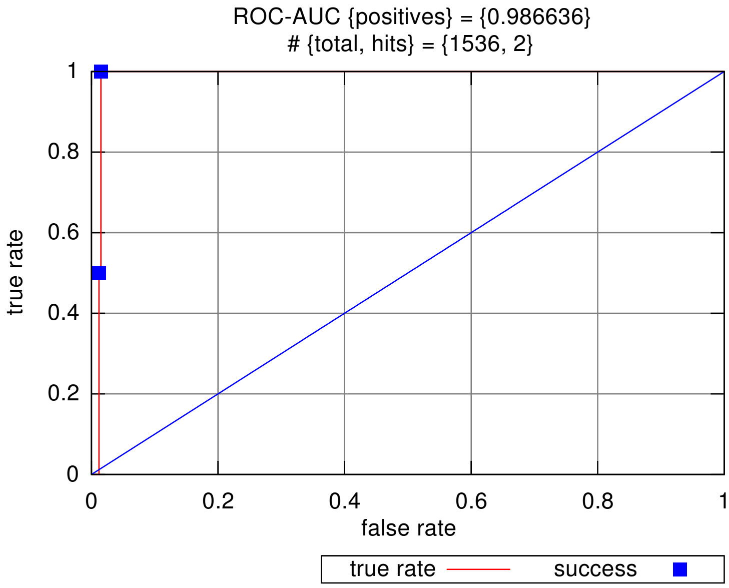

(a)

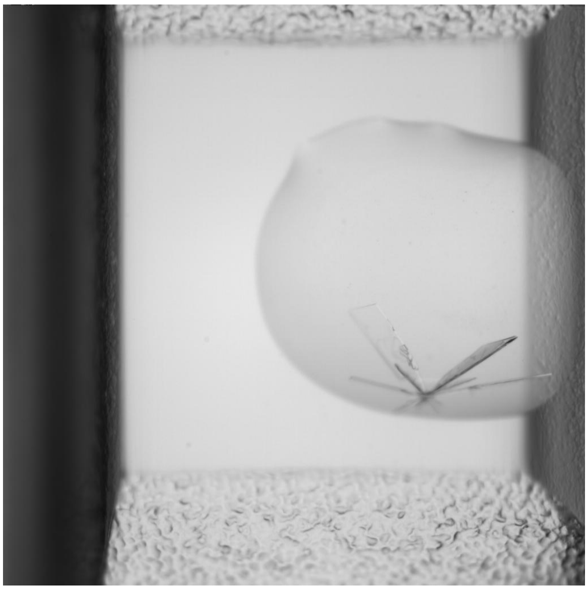

(b)

Figure 6: An ROC Curve for set ‘SpeedET-FG7335A-lbu-239-1-239’ along with its highest ranked diffraction success.

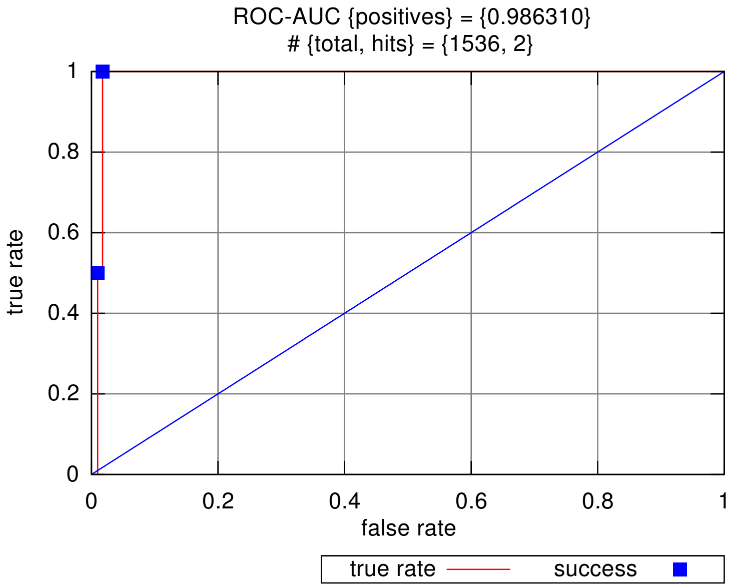

(a)

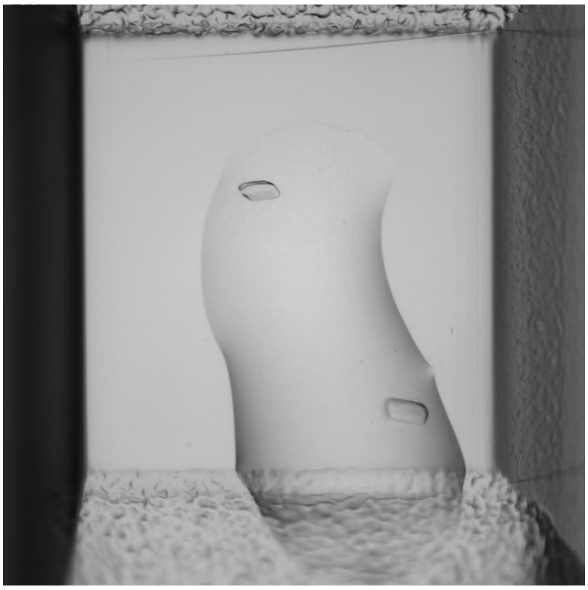

(b)

Figure 7: An ROC Curve for set ‘SpeedET-PH10062D-jsp-113-1-113’ along with its highest ranked diffraction success.

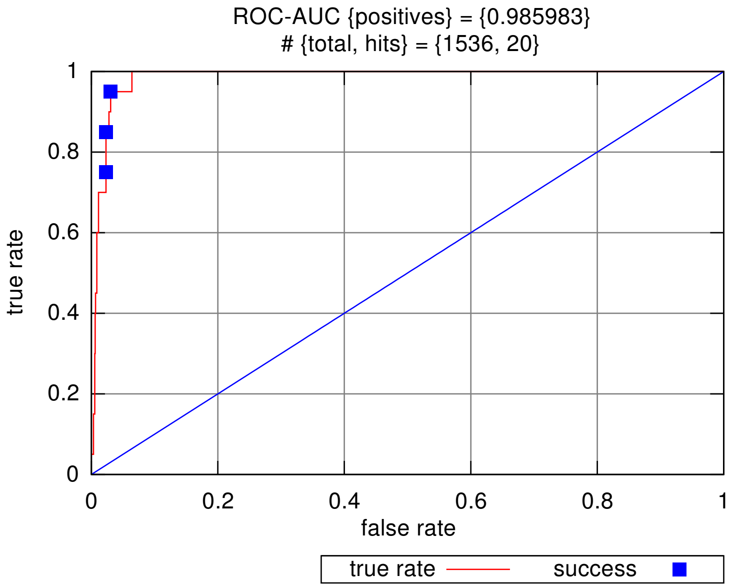

(a)

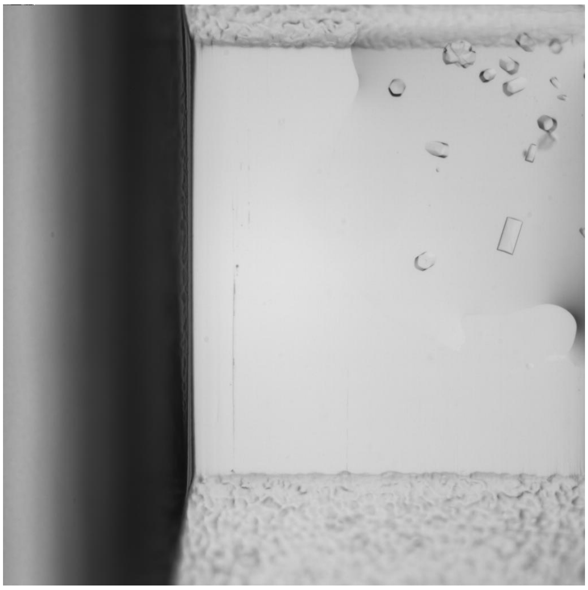

(b)

Figure 8: An ROC Curve for set 'SpeedET-CM5490D-sis-190-1-190' along with its highest ranked diffraction success.

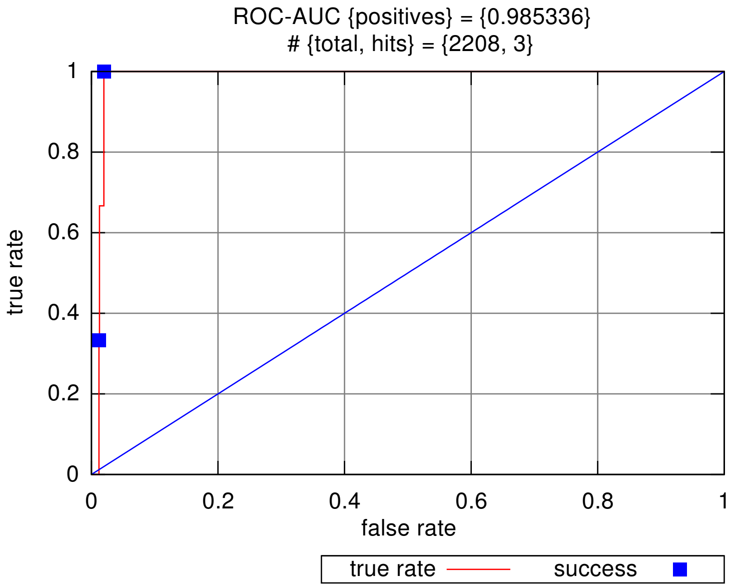

(a)

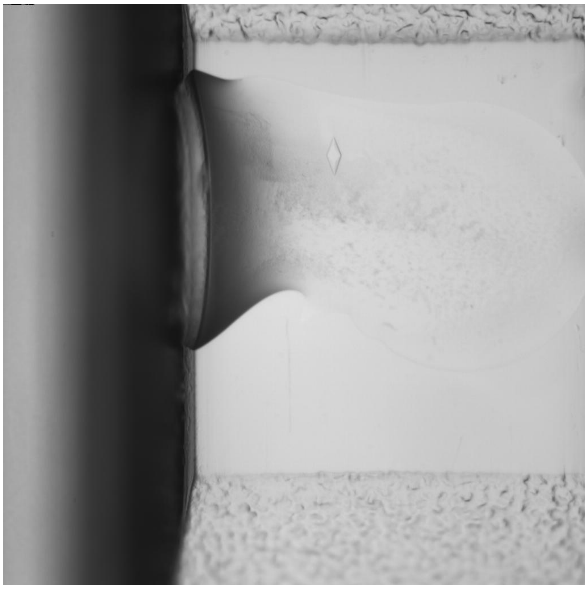

(b)

Figure 9: An ROC Curve for set ‘SpeedET-FG7343A-lpl-261-1-261’ along with its highest ranked diffraction success.

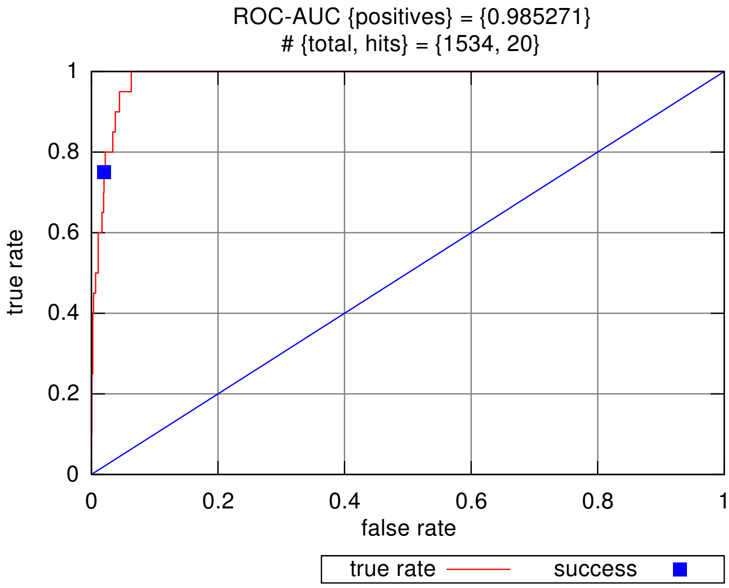

(a)

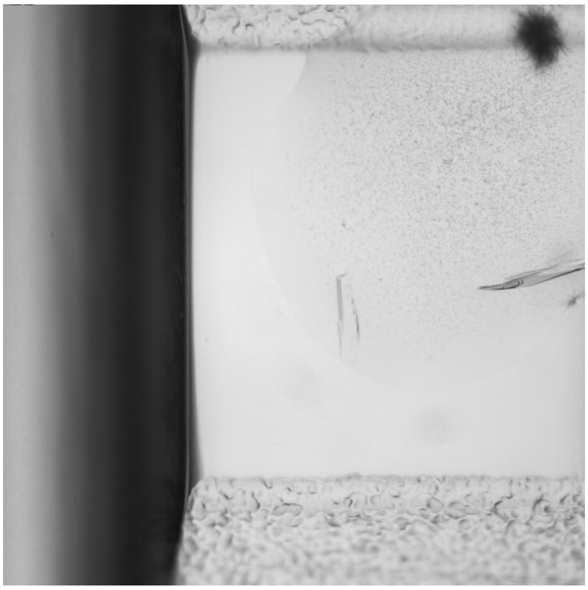

(b)

Figure 10: An ROC Curve for set 'SpeedET-CM8073A-npu-269-1-269' along with its highest ranked diffraction success.

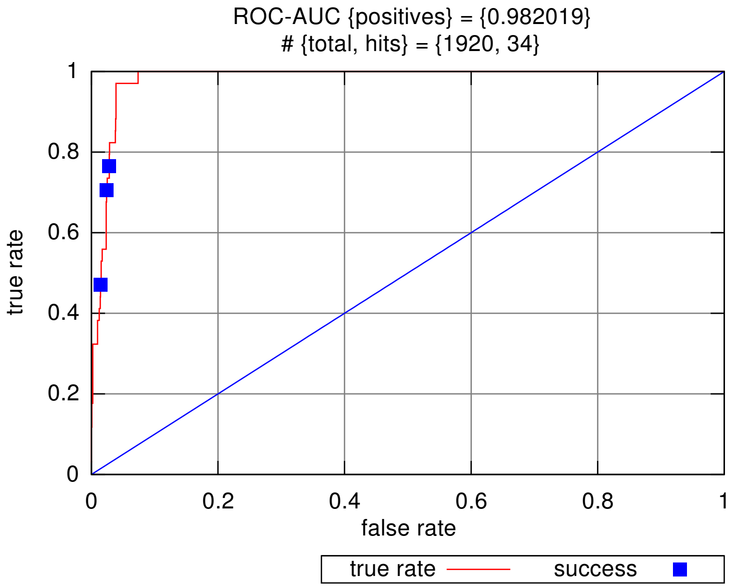

(a)

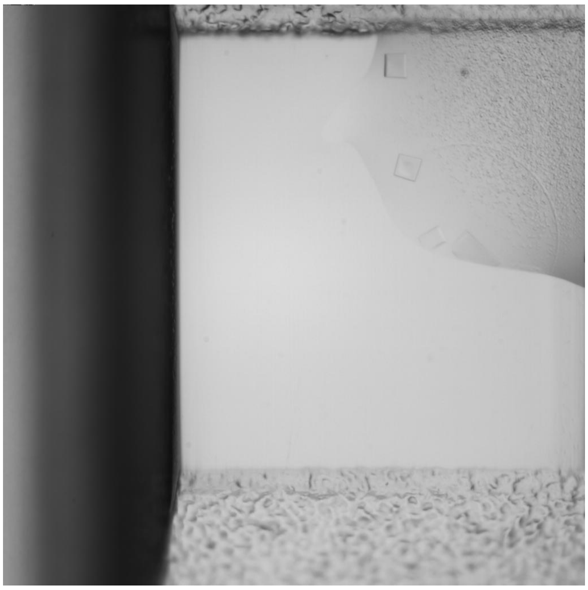

(b)

Figure 11: An ROC Curve for set 'SpeedET-PC06304B-bha-113-15-113' along with its highest ranked diffraction success.

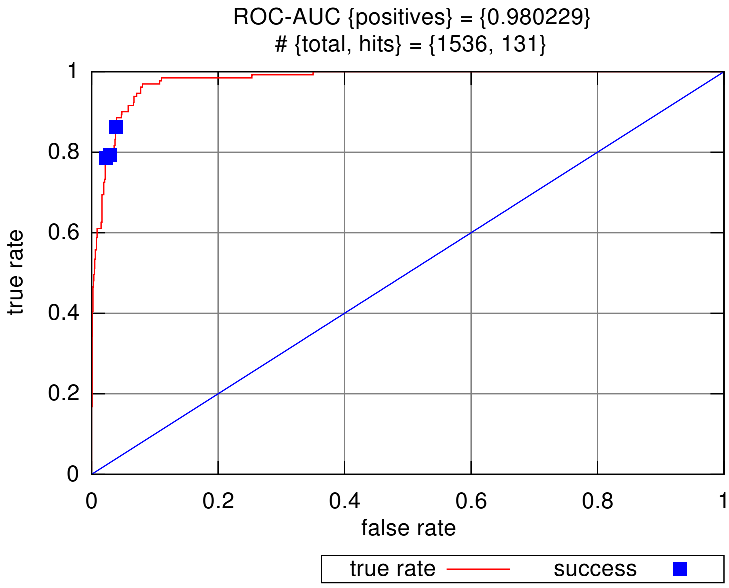

(a)

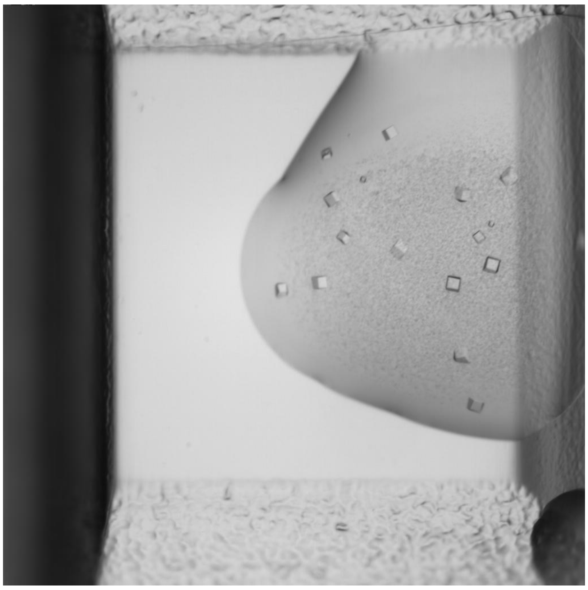

(b)

Figure 12: An ROC Curve for set 'SpeedET-FG7396A-pma-151-1-151' along with its highest ranked diffraction success.

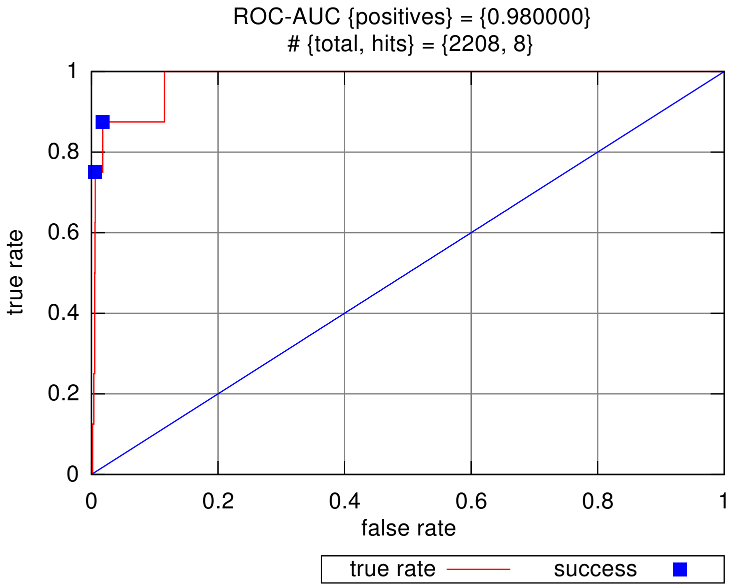

(a)

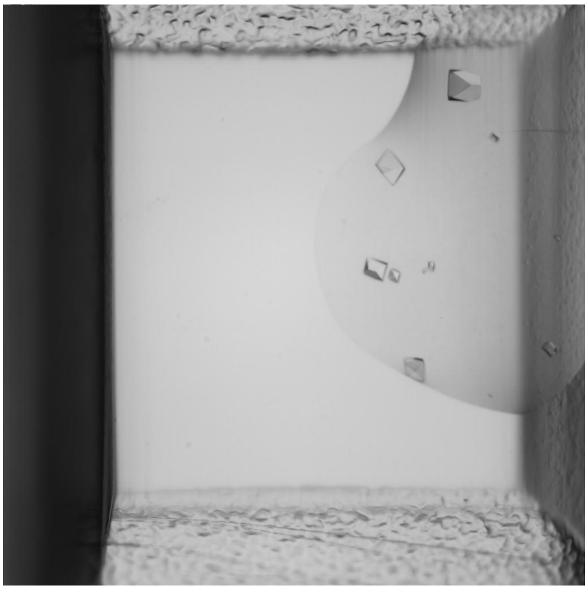

(b)

Figure 13: An ROC Curve for set 'SpeedET-PE00037D-pro-126-1-126' along with its highest ranked diffraction success.

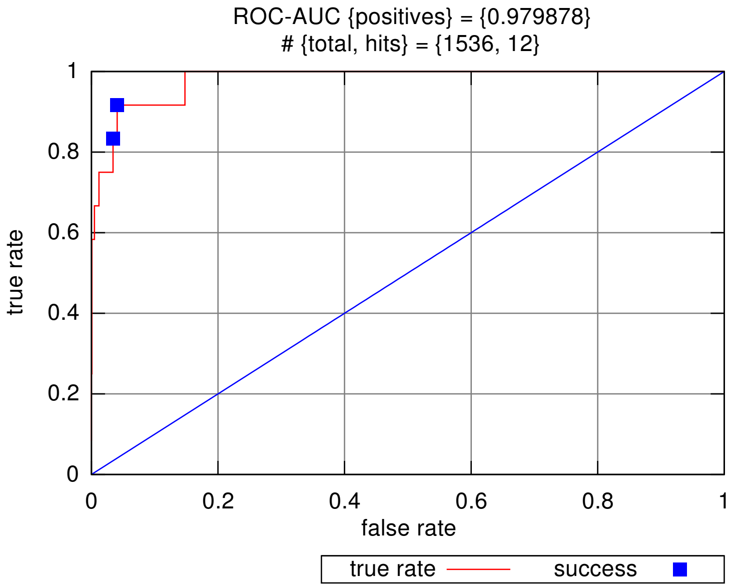

(a)

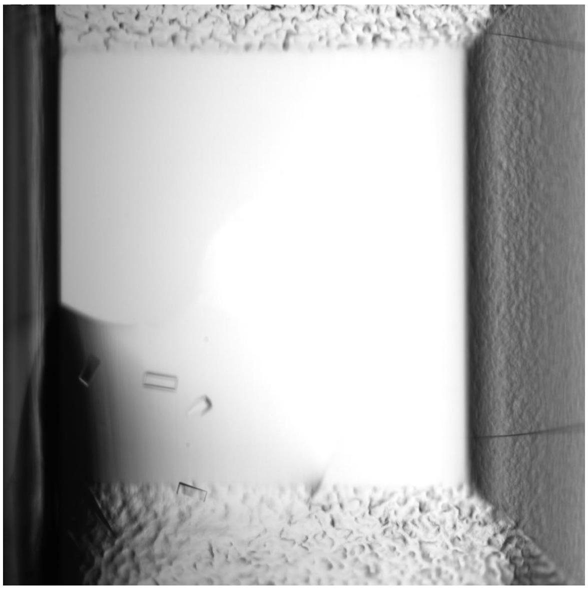

(b)

Figure 14: An ROC Curve for set 'SpeedET-FL10929A-ava-342-1-342' along with its highest ranked diffraction success.

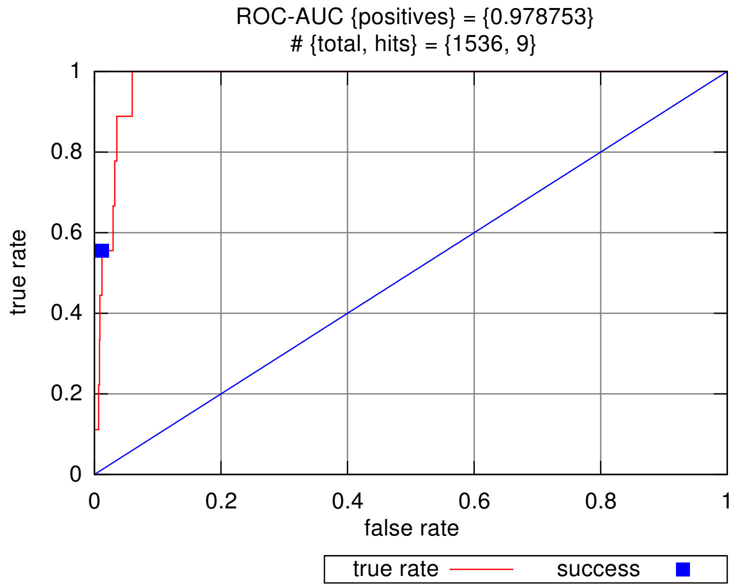

(a)

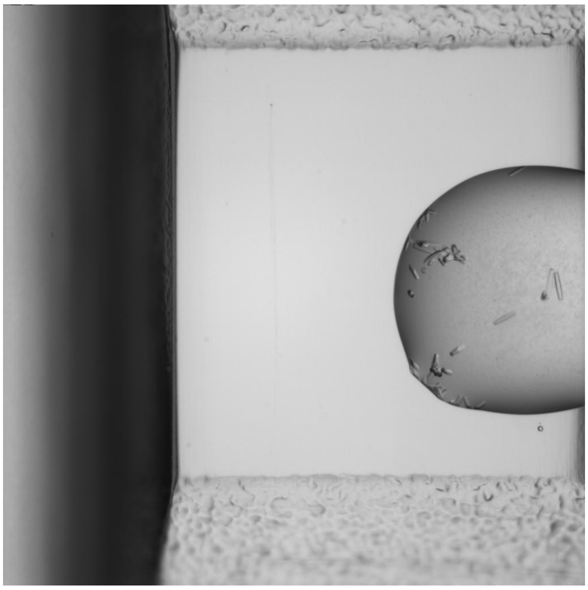

(b)

Figure 15: An ROC Curve for set 'SpeedET-FK8801A-reu-152-1-152' along with its highest ranked diffraction success.

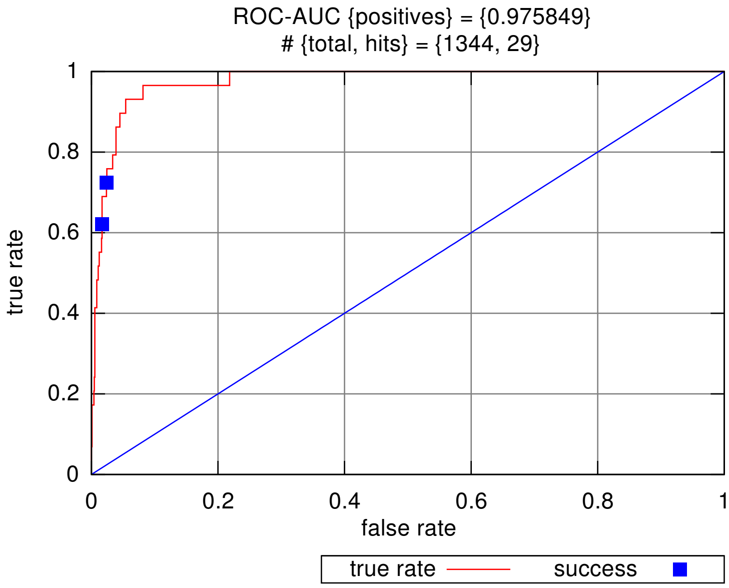

(a)

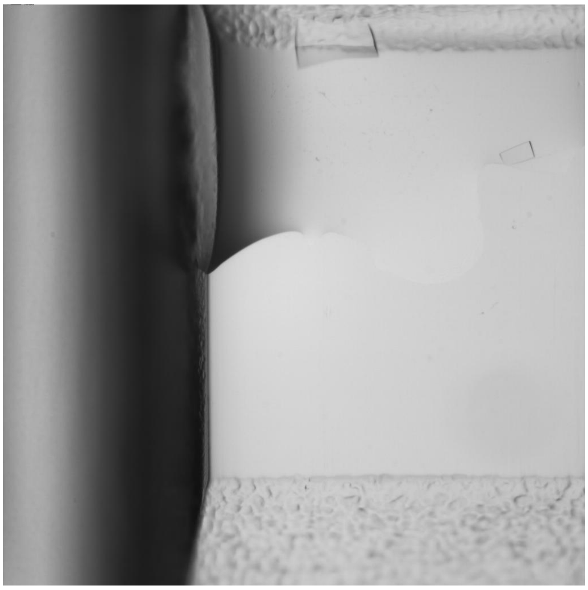

(b)

Figure 16: An ROC Curve for set 'SpeedET-FJ9519A-mma-166-1-166' along with its highest ranked diffraction success.

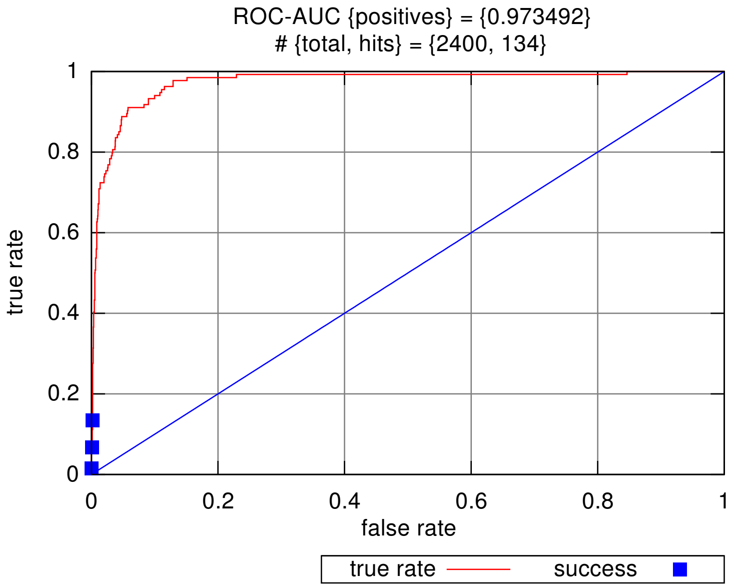

(a)

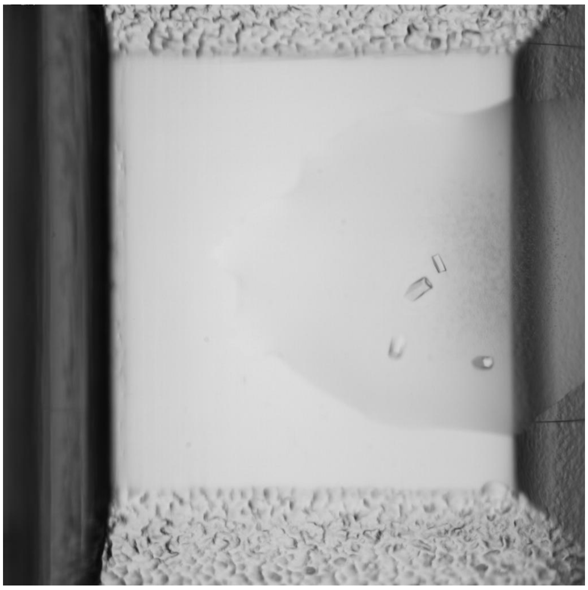

(b)

Figure 17: An ROC Curve for set 'SpeedET-CM8385A-ccr-112-1-112' along with its highest ranked diffraction success.

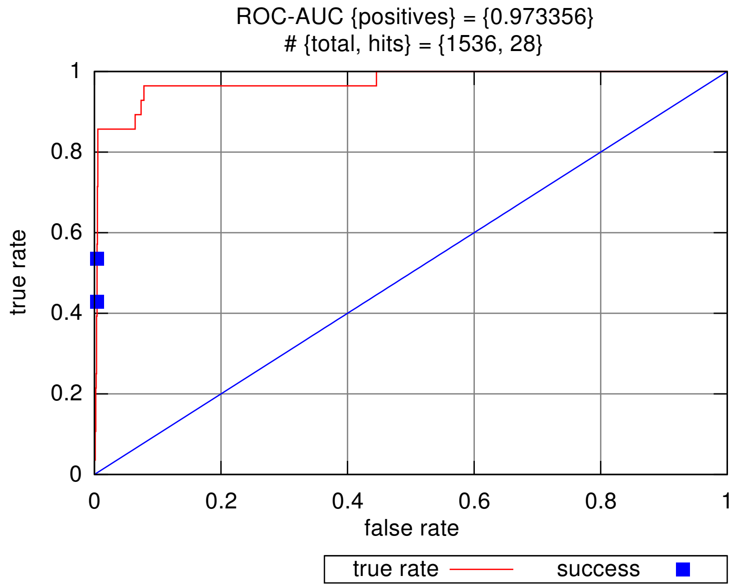

(a)

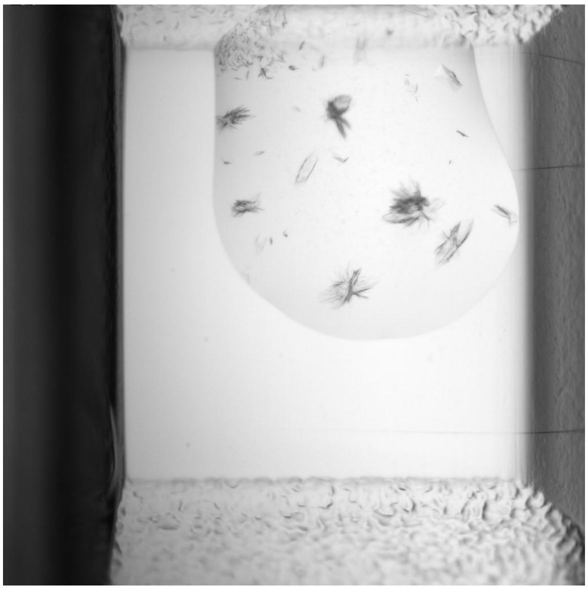

(b)

Figure 18: An ROC Curve for set 'SpeedET-FL11009A-cgl-254-1-254' along with its highest ranked diffraction success.

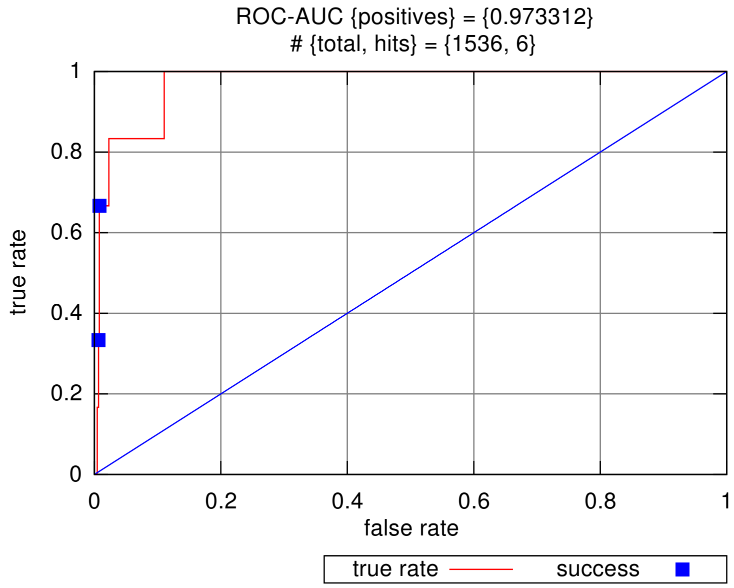

(a)

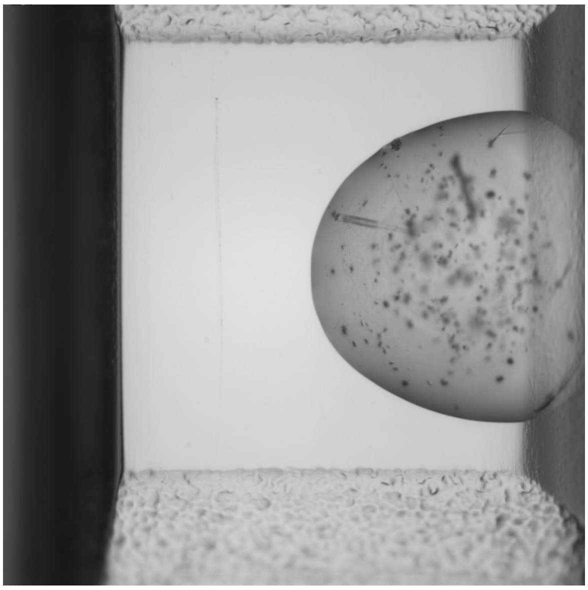

(b)

Figure 19: An ROC Curve for set 'SpeedET-PD07049G-npu-160-1-160-Q139Y-Q140Y-E141Y' along with its highest ranked diffraction success.

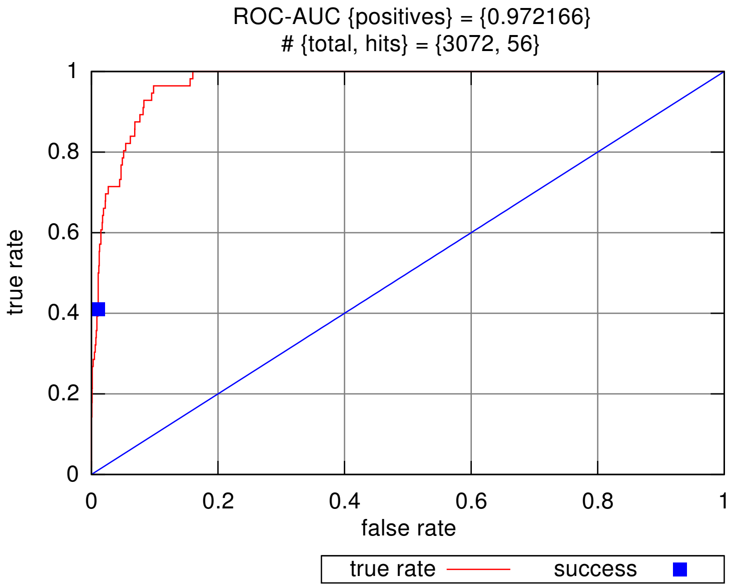

(a)

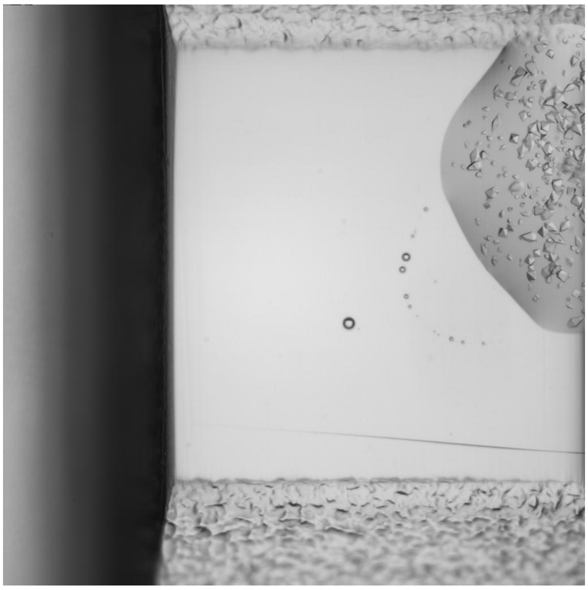

(b)

Figure 20: An ROC Curve for set 'SpeedET-FK8817C-reu-111-1-111' along with its highest ranked diffraction success.

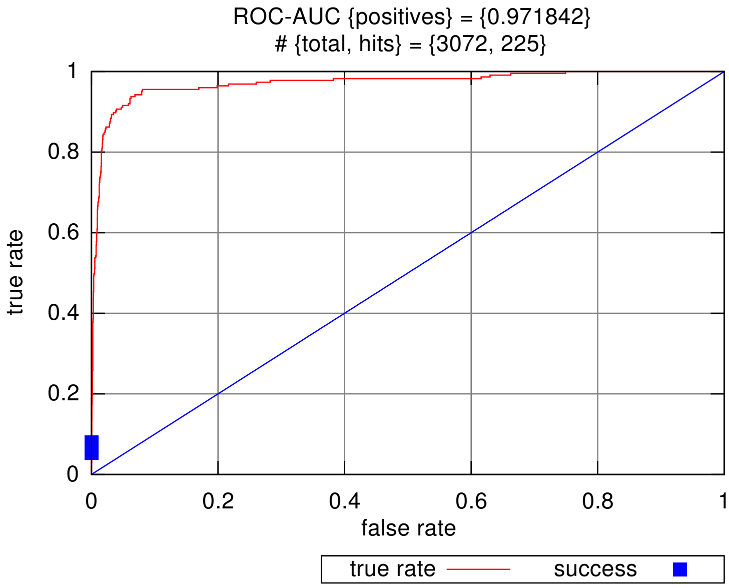

(a)

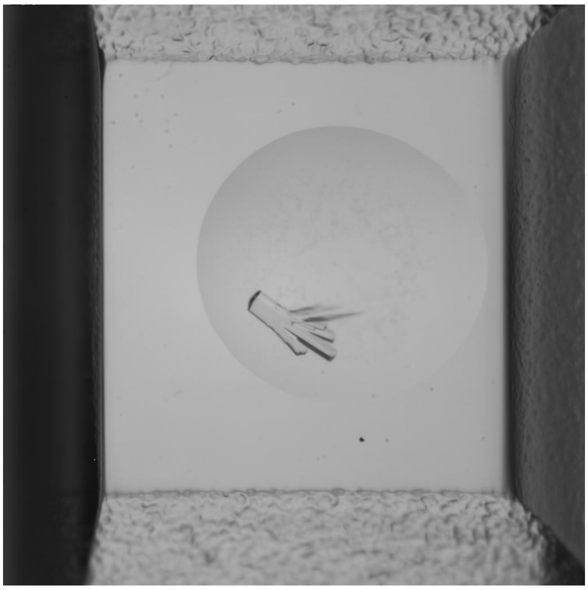

(b)

Figure 21: An ROC Curve for set ‘SpeedET-FJ8838A-cgl-136-1-136’ along with its highest ranked diffraction success.

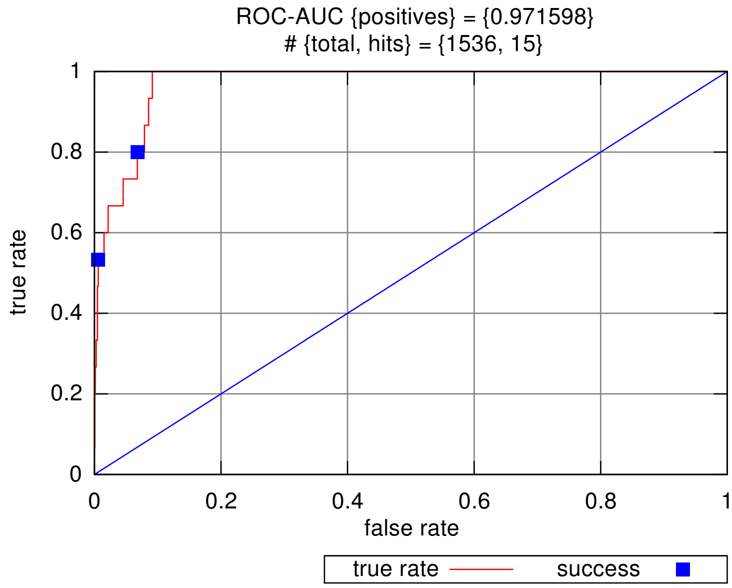

(a)

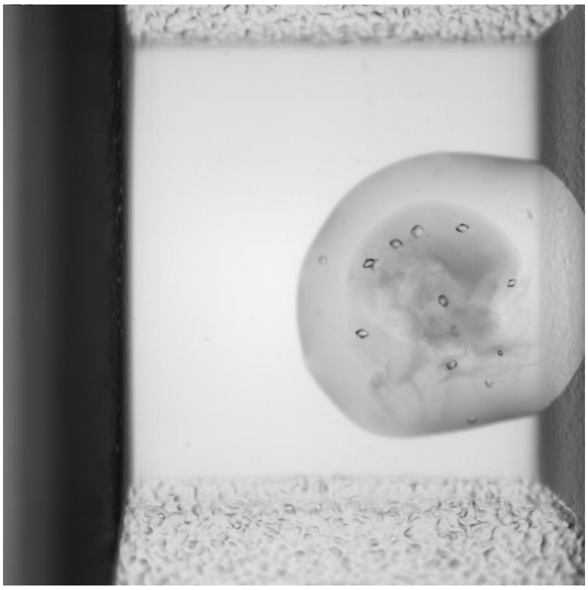

(b)

Figure 22: An ROC Curve for set 'SpeedET-FG7459A-ssu-149-1-149' along with its highest ranked diffraction success.

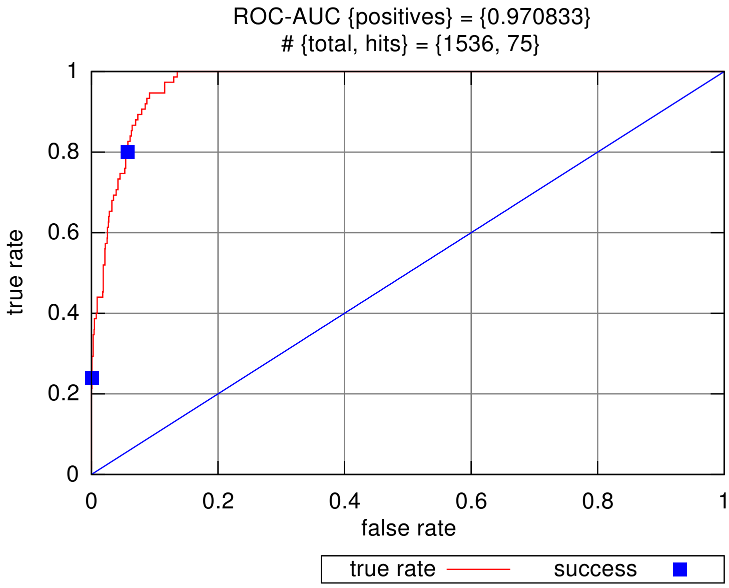

(a)

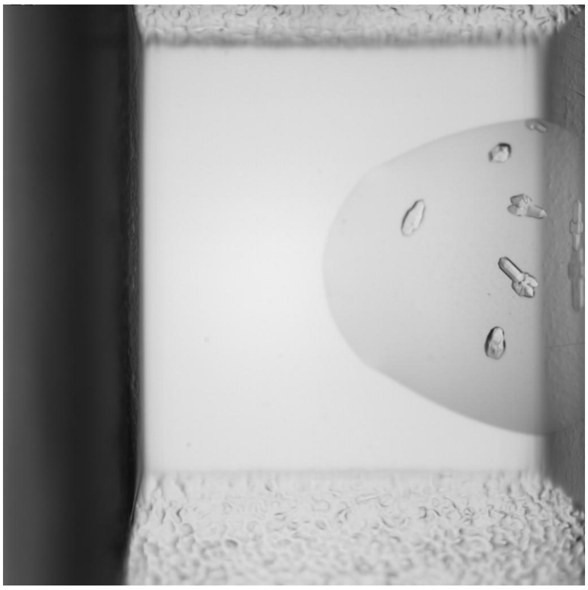

(b)

Figure 23: An ROC Curve for set 'SpeedET-FG7419A-sav-154-1-154' along with its highest ranked diffraction success.

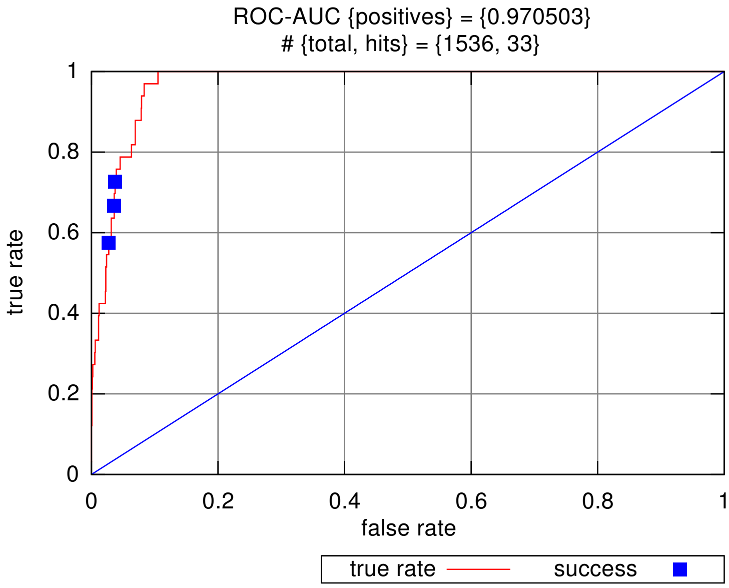

(a)

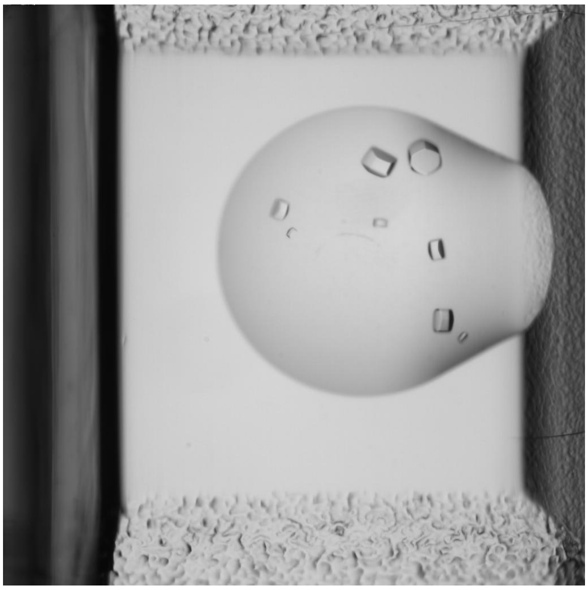

(b)

Figure 24: An ROC Curve for set 'SpeedET-PE00012N-lic-159-1-159' along with its highest ranked diffraction success.

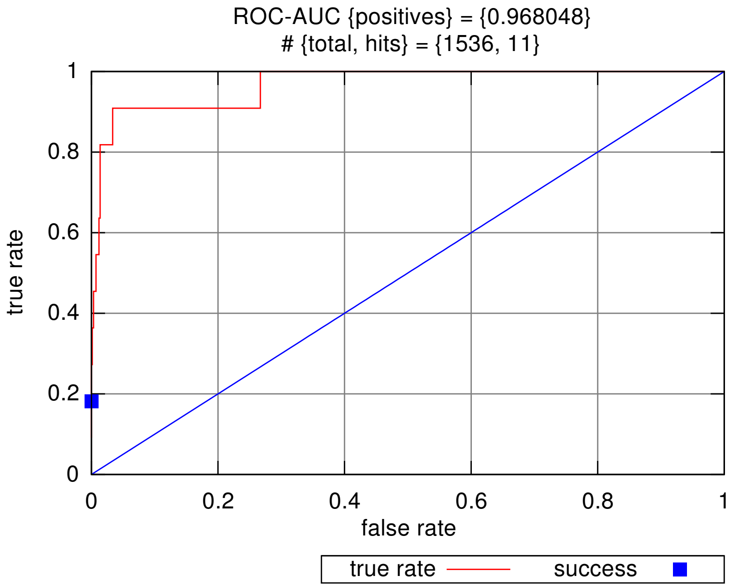

(a)

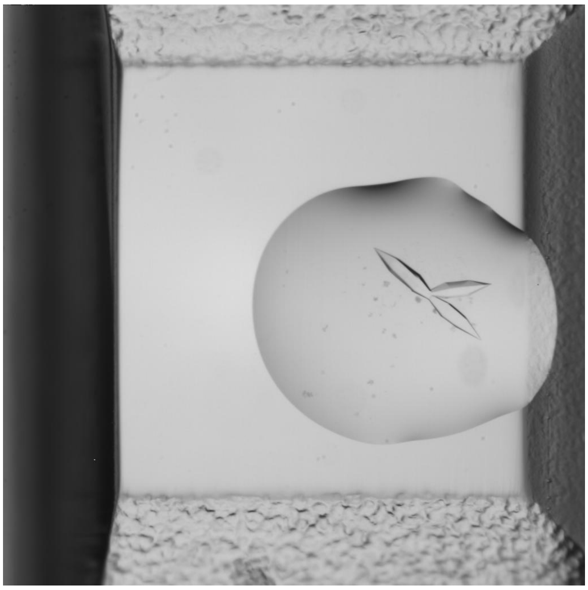

(b)

Figure 25: An ROC Curve for set ‘SpeedET-NP_811092.1-bth-354-1-354’ along with its highest ranked diffraction success.

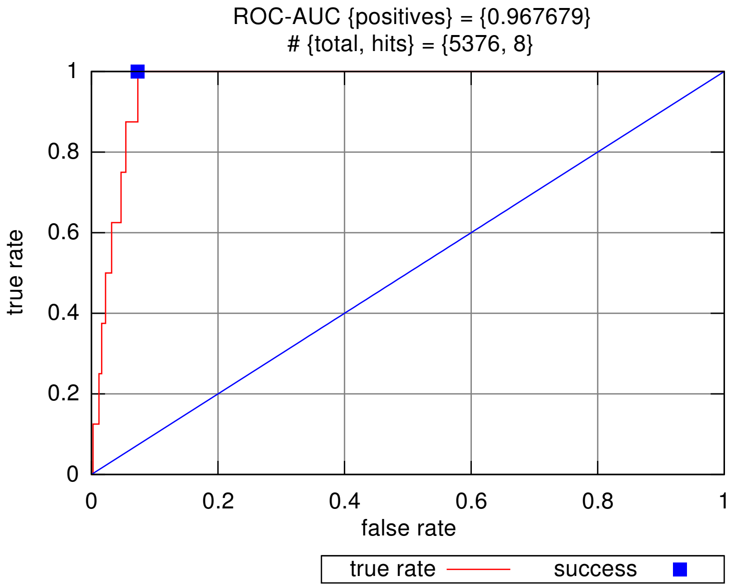

(a)

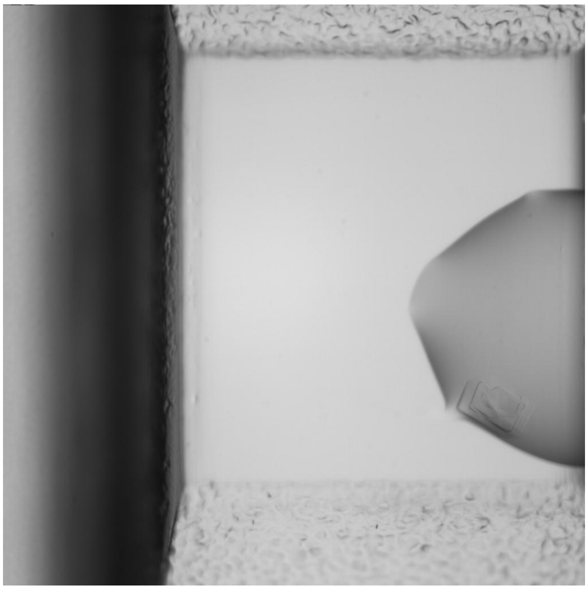

(b)

Figure 26: An ROC Curve for set 'SpeedET-16420133-sty-198-1-198' along with its highest ranked diffraction success.

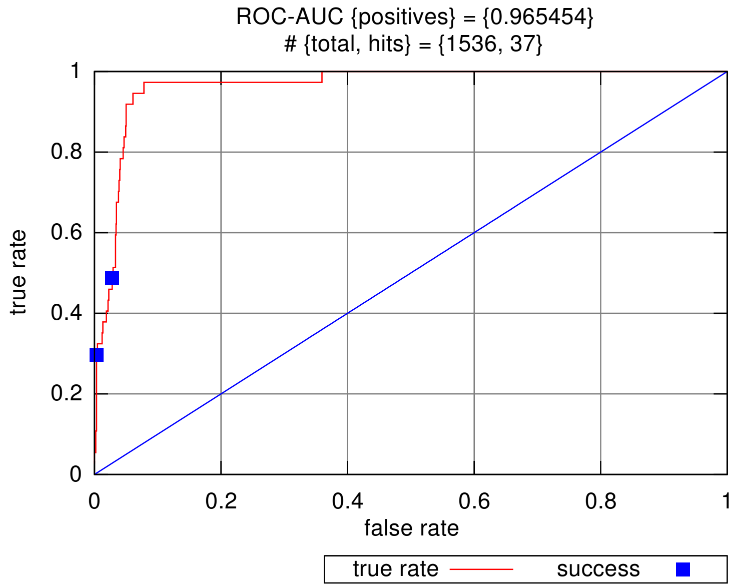

(a)

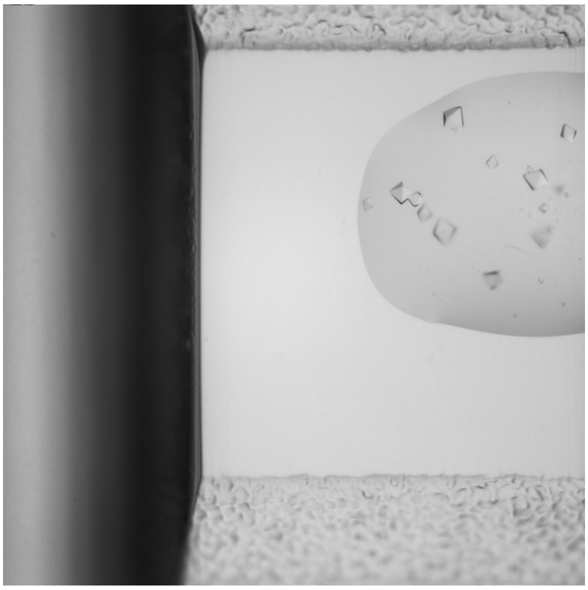

(b)

Figure 27: An ROC Curve for set 'SpeedET-PC05870B-bsu-161-1-161-K146Y' along with its highest ranked diffraction success.

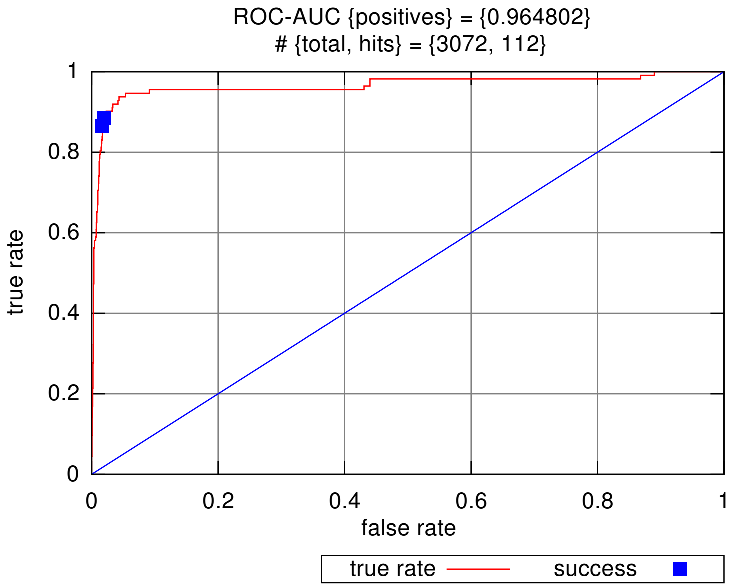

(a)

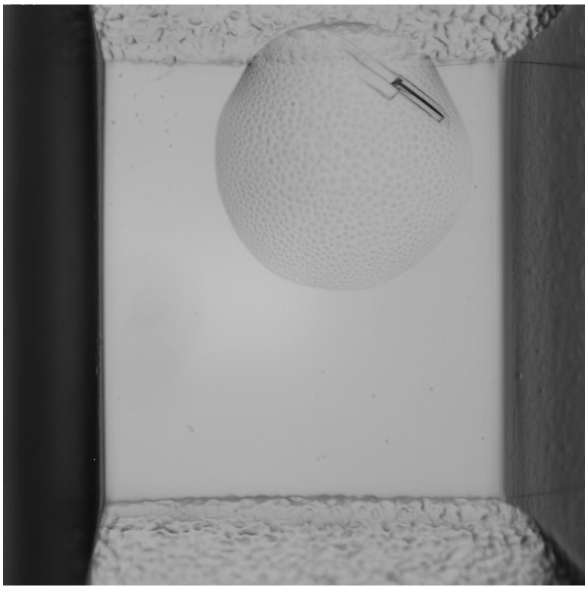

(b)

Figure 28: An ROC Curve for set ‘SpeedET-FK9742A-sag-182-1-182’ along with its highest ranked diffraction success.

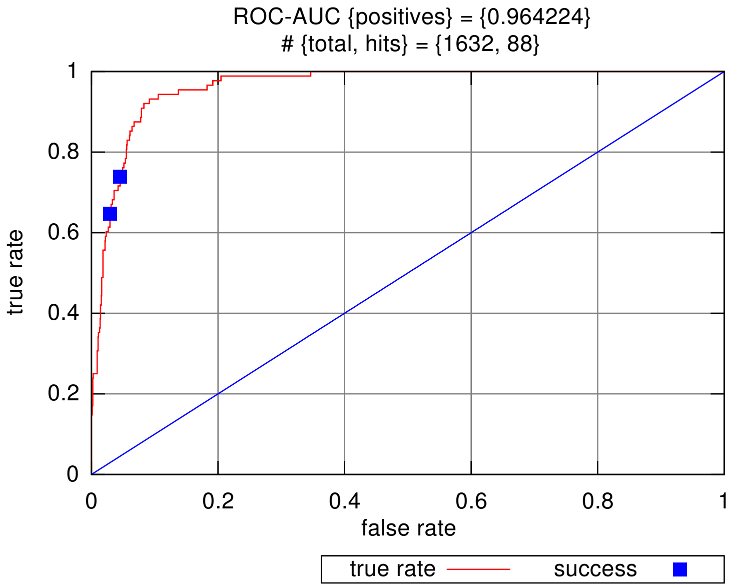

(a)

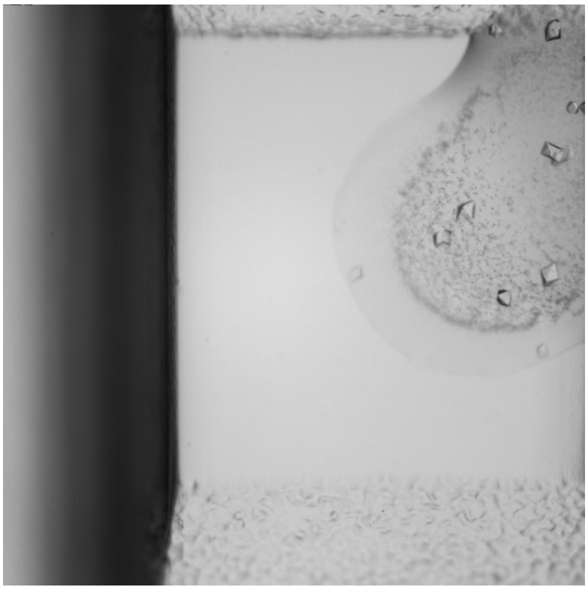

(b)

Figure 29: An ROC Curve for set 'SpeedET-PE00025C-lca-122-1-122' along with its highest ranked diffraction success.

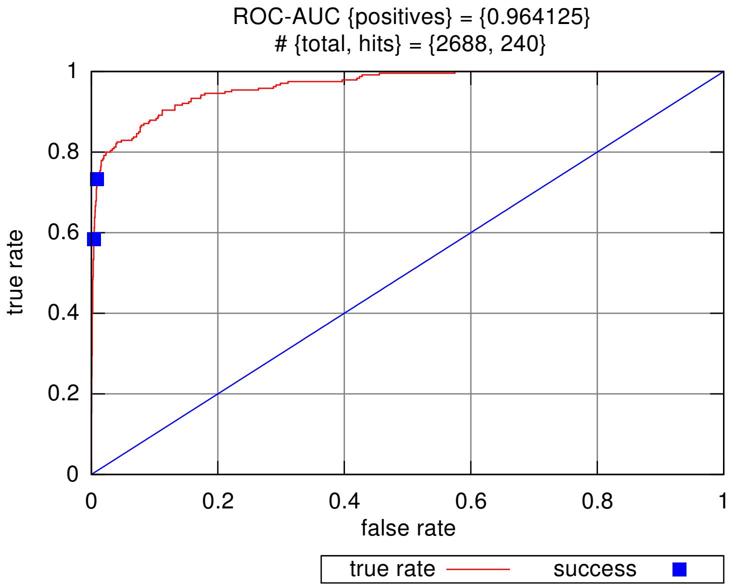

(a)

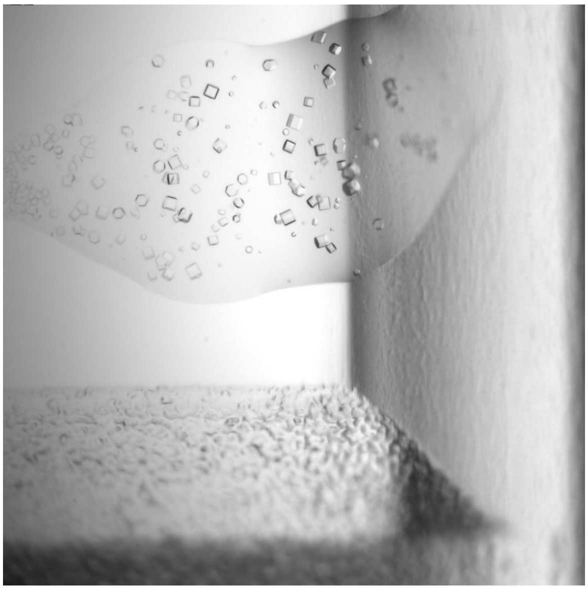

(b)

Figure 30: An ROC Curve for set 'SpeedET-FB7482B-jsp-159-1-159' along with its highest ranked diffraction success.

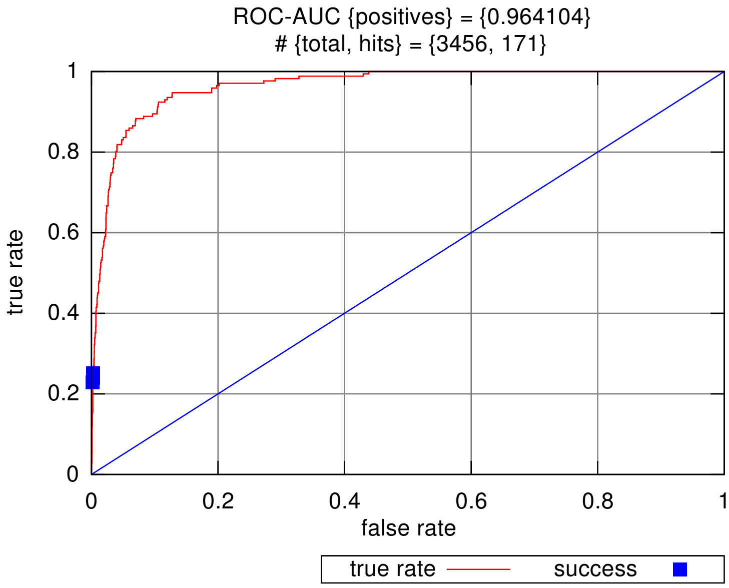

(a)

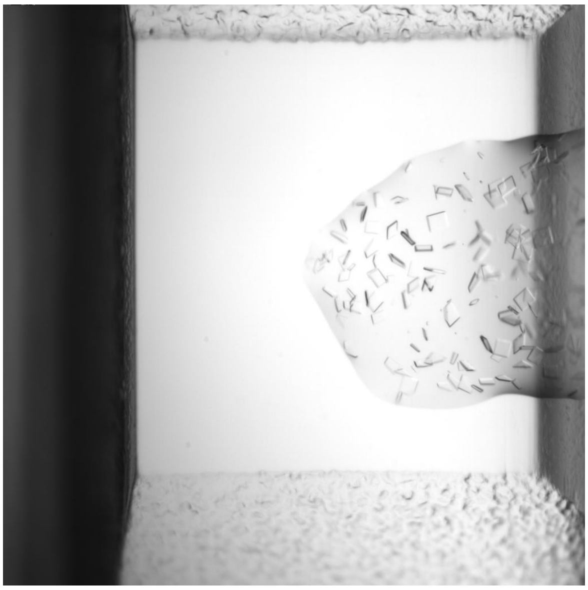

(b)

Figure 31: An ROC Curve for set 'SpeedET-HP10645A-sam-274-1-274' along with its highest ranked diffraction success.

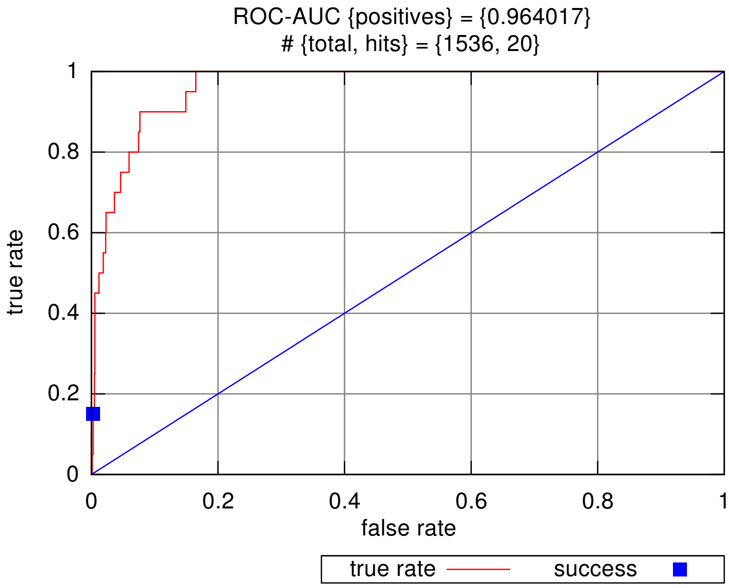

(a)

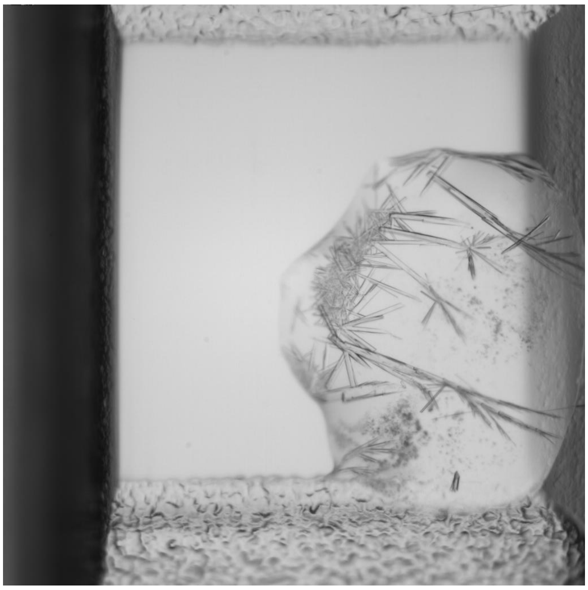

(b)

Figure 32: An ROC Curve for set 'SpeedET-FK5749A-xca-250-1-250' along with its highest ranked diffraction success.

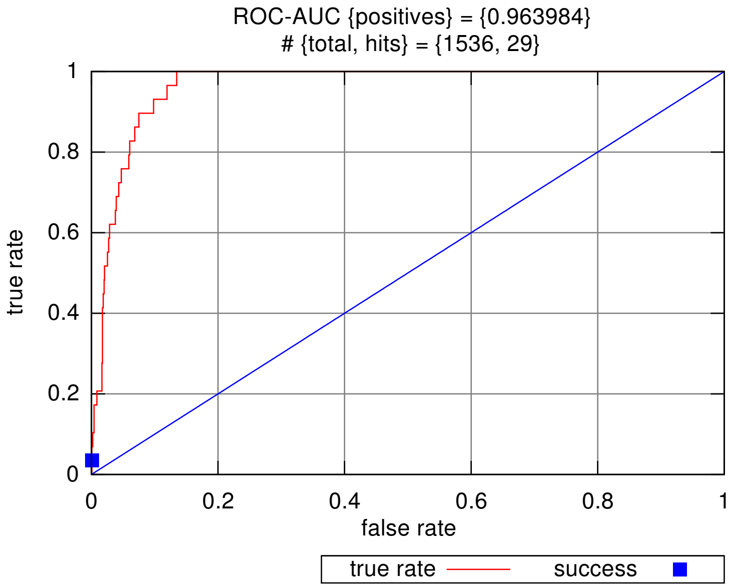

(a)

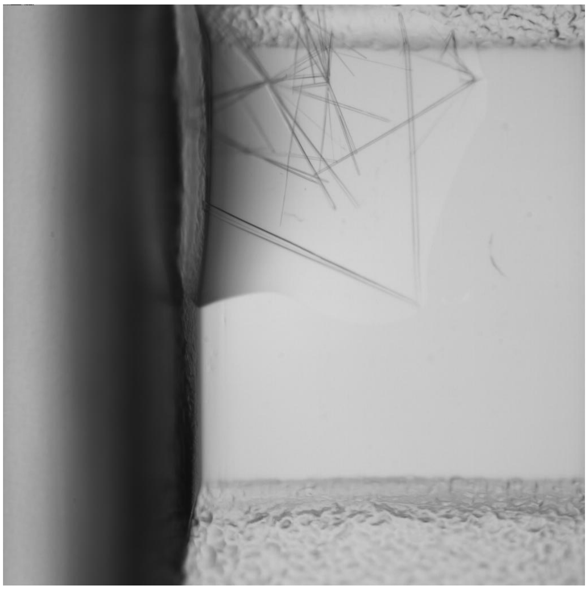

(b)

Figure 33: An ROC Curve for set ‘SpeedET-GN7730A-ocn-116-1-116’ along with its highest ranked diffraction success.

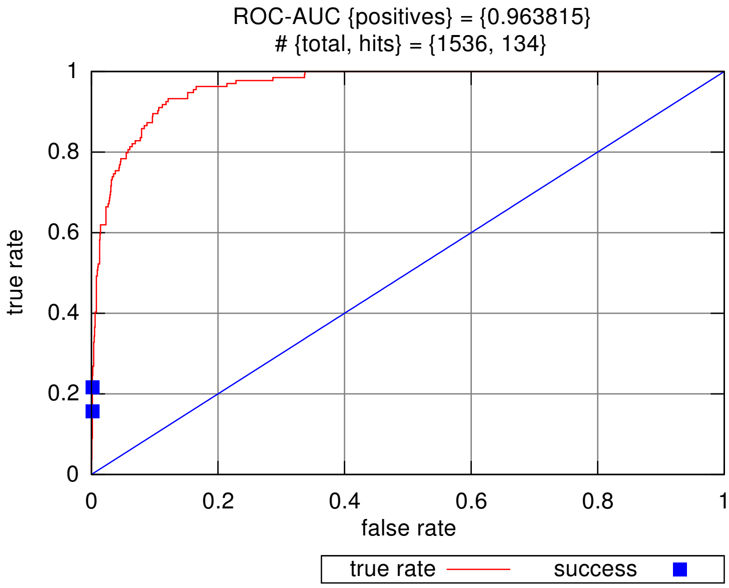

(a)

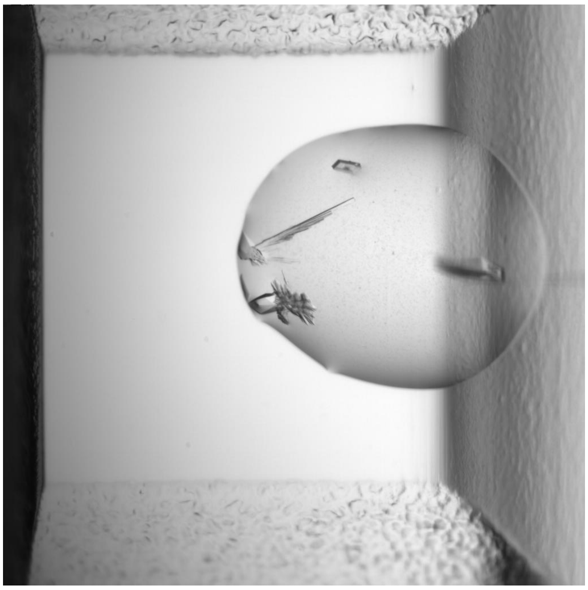

(b)

Figure 34: An ROC Curve for set 'SpeedET-HP1666A-dde-363-1-363' along with its highest ranked diffraction success.

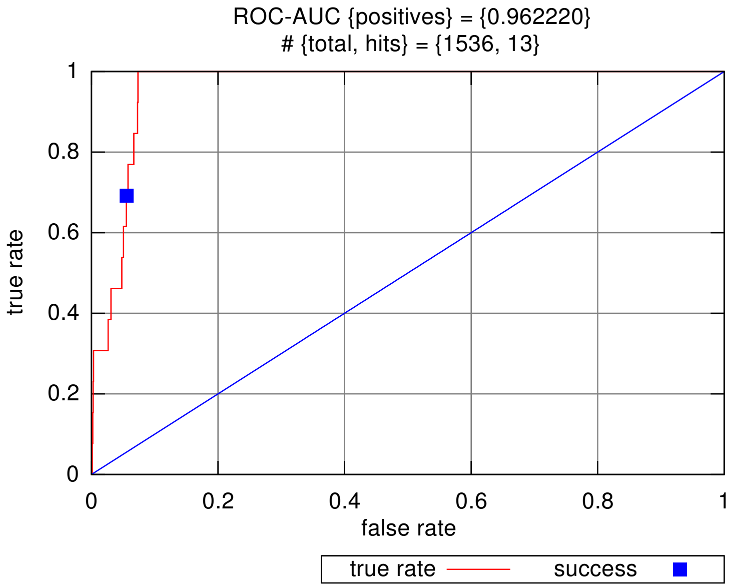

(a)

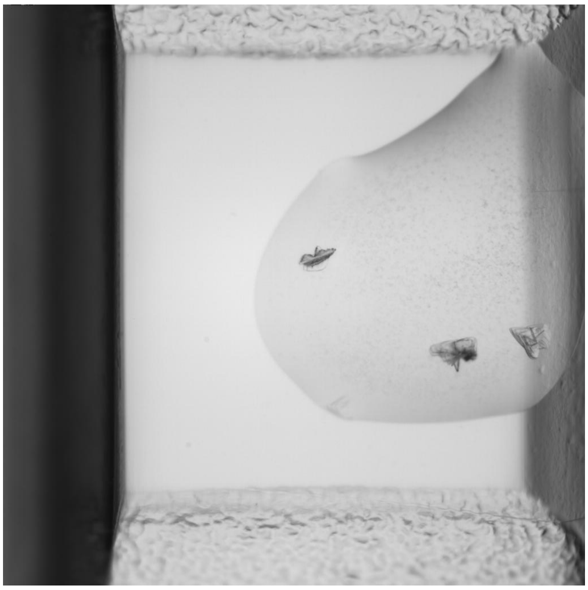

(b)

Figure 35: An ROC Curve for set 'SpeedET-FG7402A-ppu-156-1-156' along with its highest ranked diffraction success.

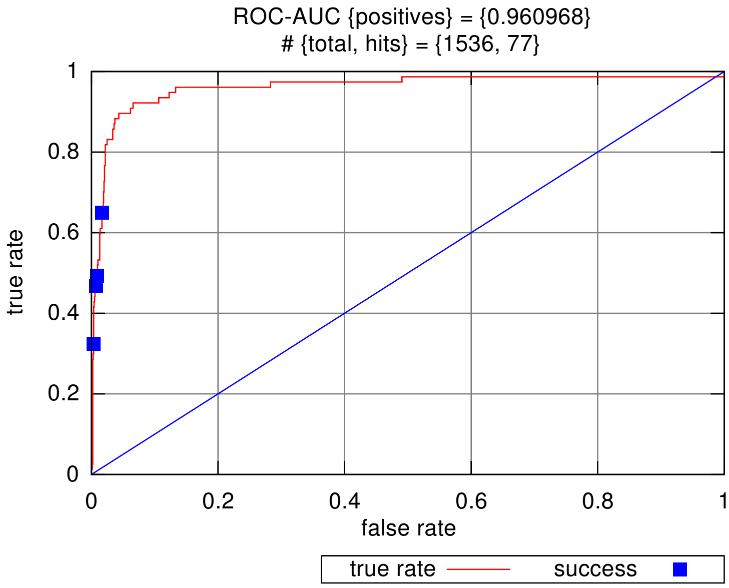

(a)

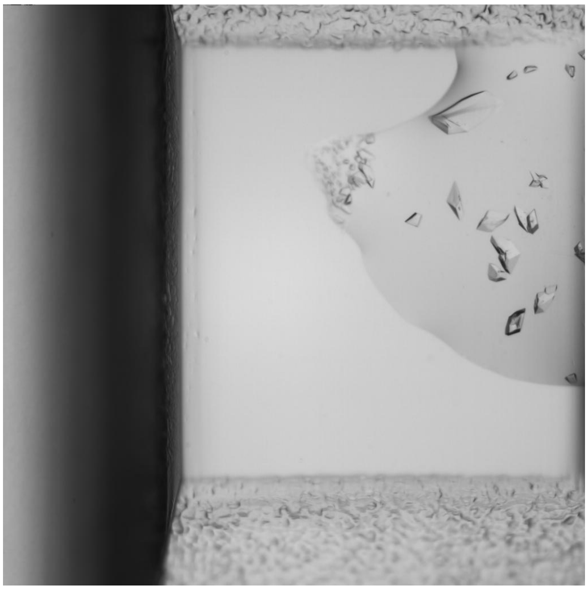

(b)

Figure 36: An ROC Curve for set 'SpeedET-PG9822D-sfr-126-1-126' along with its highest ranked diffraction success.

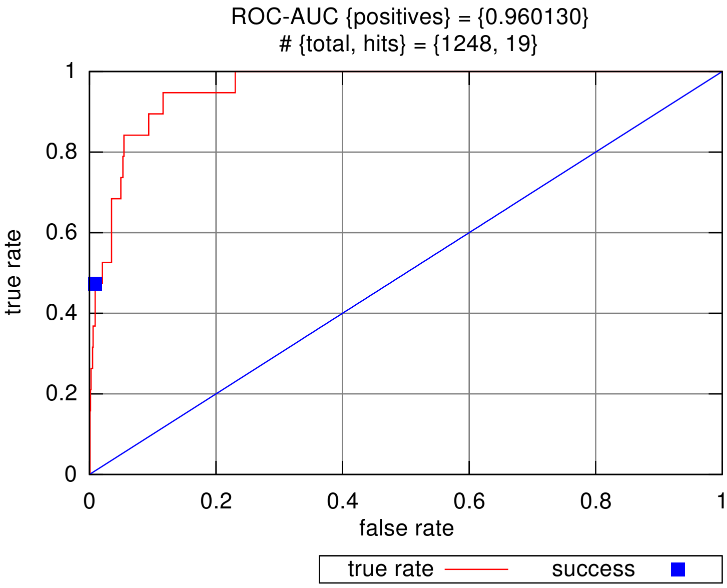

(a)

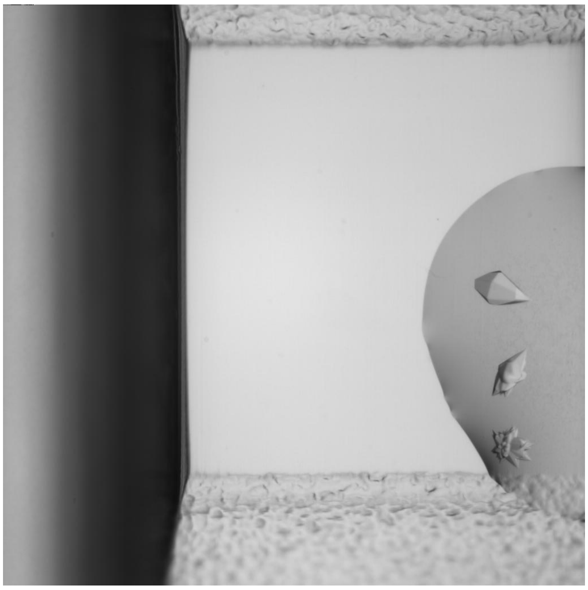

(b)

Figure 37: An ROC Curve for set 'SpeedET-FJ8809A-jsp-150-1-150' along with its highest ranked diffraction success.

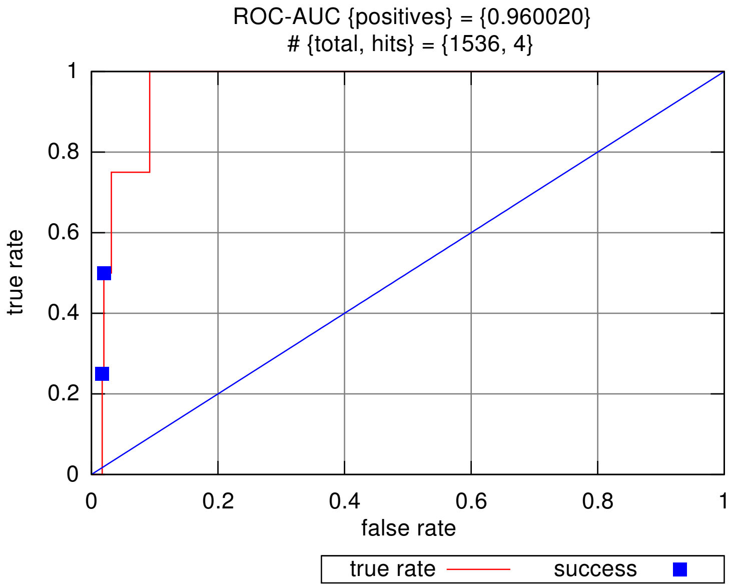

(a)

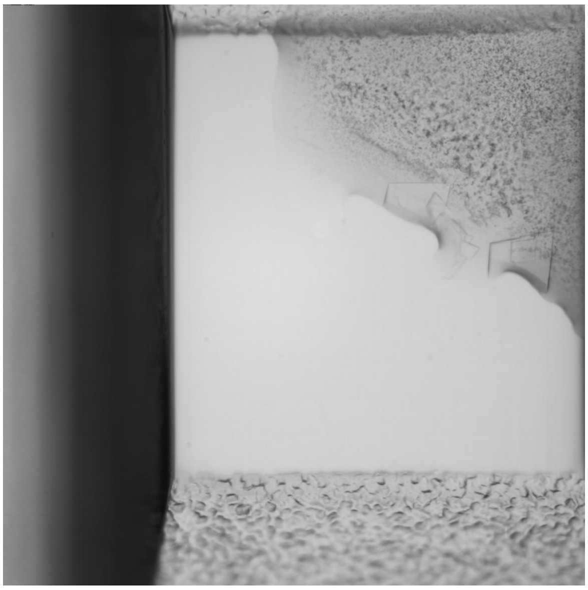

(b)

Figure 38: An ROC Curve for set 'SpeedET-PE00092D-npu-121-1-121-E103A-Q104A-D106A' along with its highest ranked diffraction success.

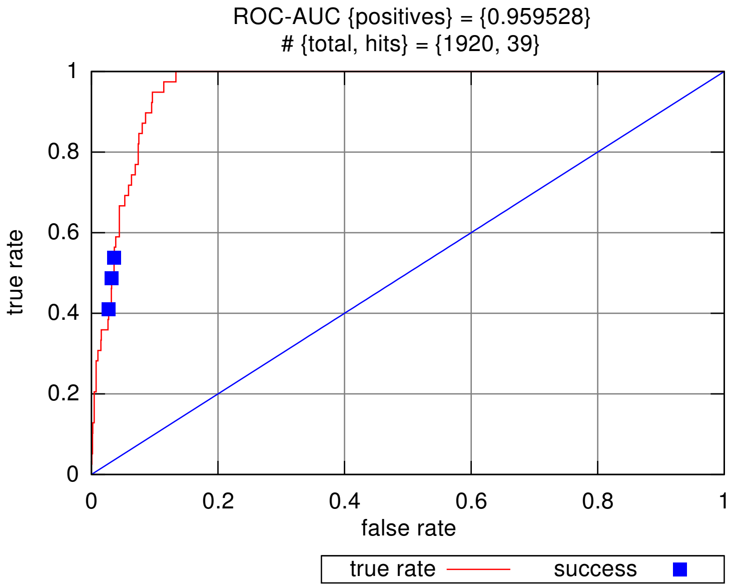

(a)

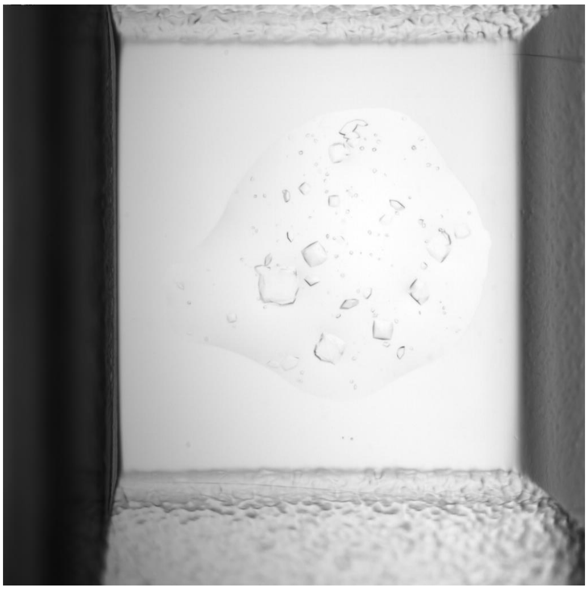

(b)

Figure 39: An ROC Curve for set ‘SpeedET-FL1347A-cgl-374-1-374’ along with its highest ranked diffraction success.

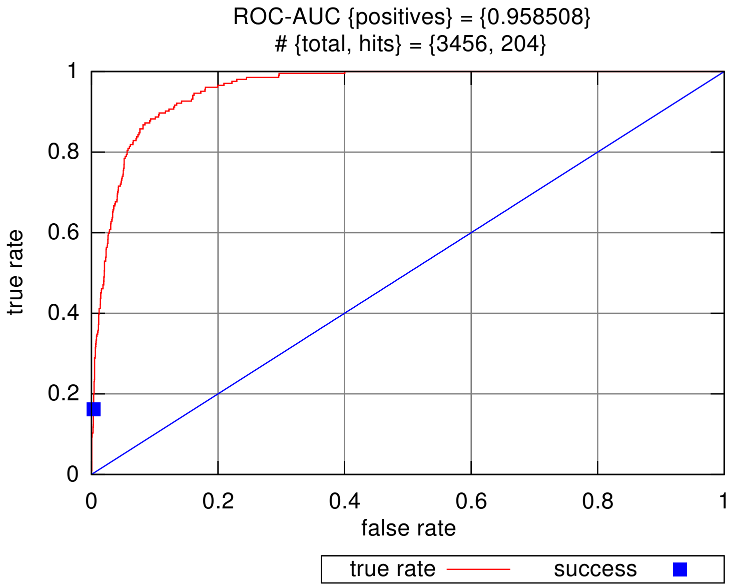

(a)

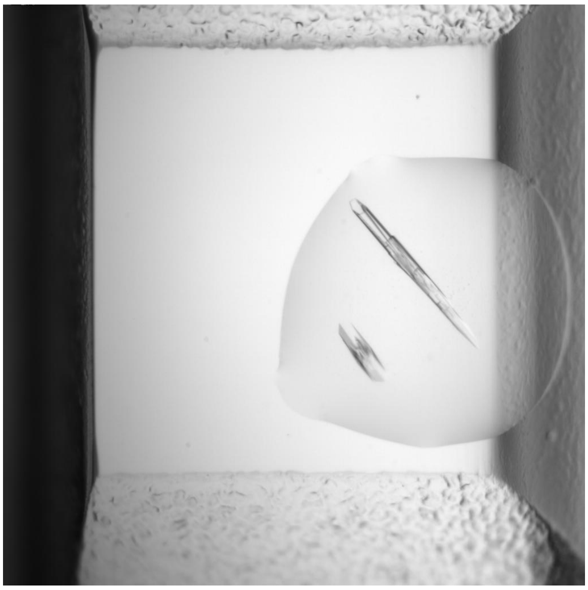

(b)

Figure 40: An ROC Curve for set 'SpeedET-FJ8832A-bxe-140-1-140' along with its highest ranked diffraction success.

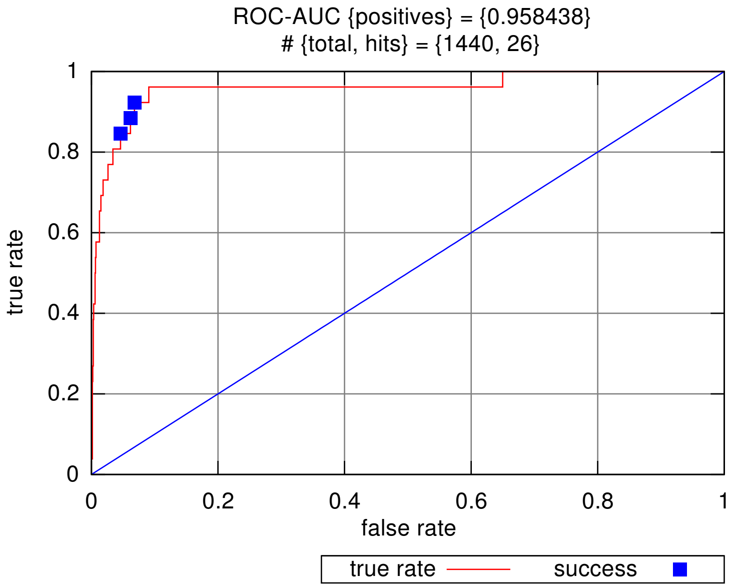

(a)

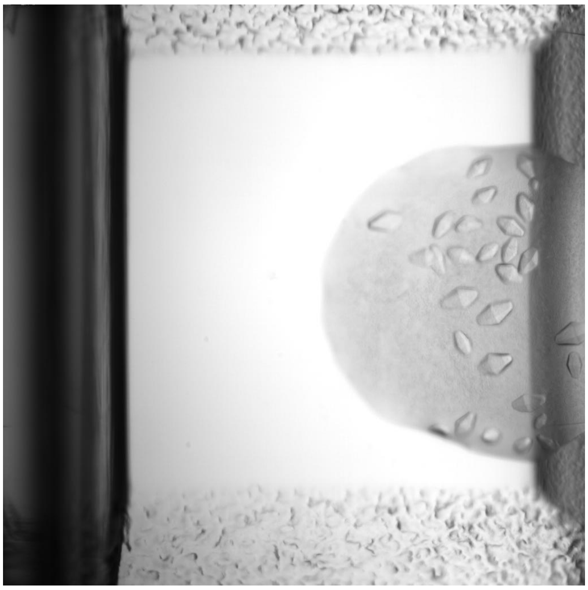

(b)

Figure 41: An ROC Curve for set 'SpeedET-FB10608A-asp-166-1-166' along with its highest ranked diffraction success.

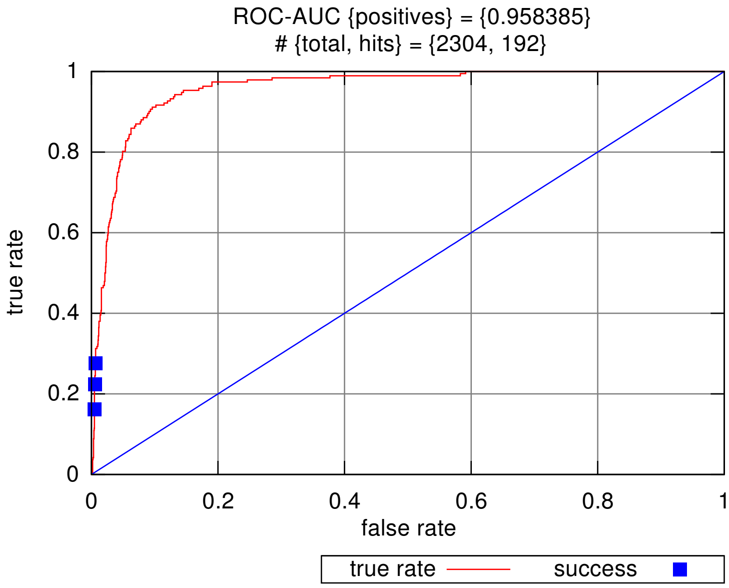

(a)

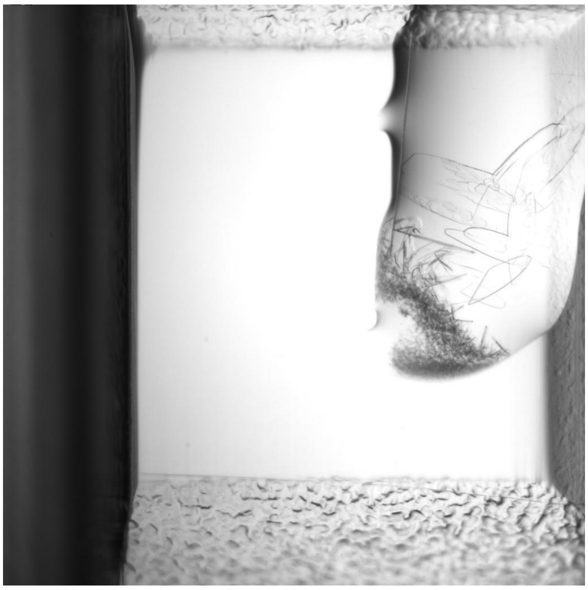

(b)

Figure 42: An ROC Curve for set ‘SpeedET-RK10654A-bth-295-1-295’ along with its highest ranked diffraction success.

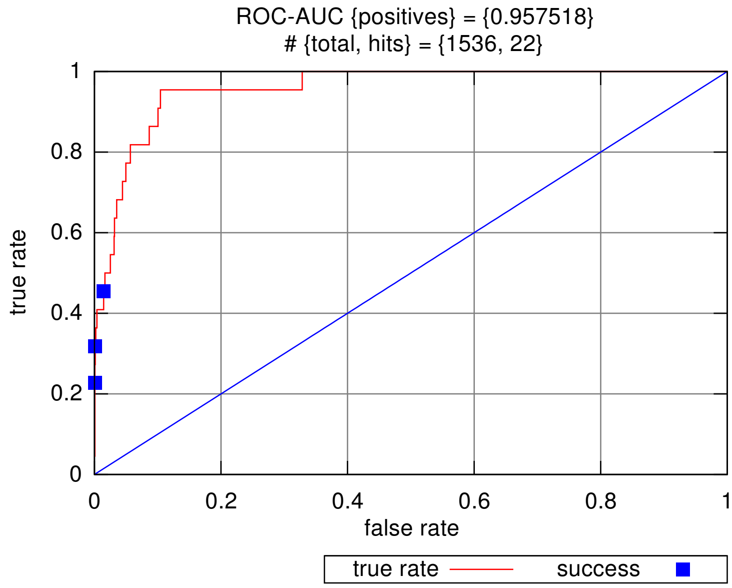

(a)

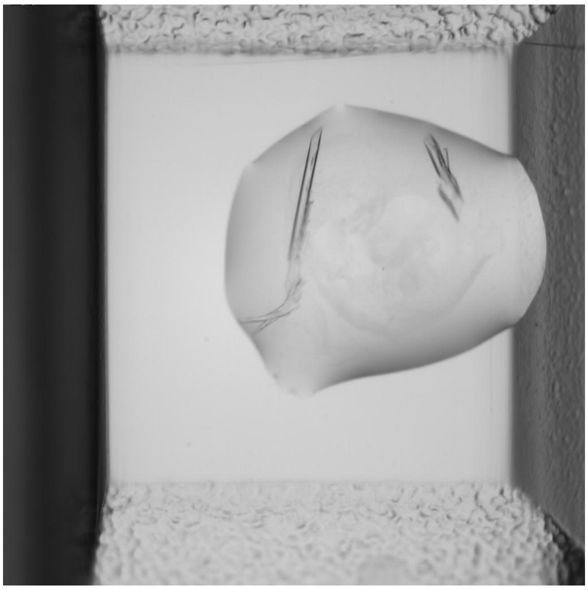

(b)

Figure 43: An ROC Curve for set 'SpeedET-FH7484A-npu-194-1-194' along with its highest ranked diffraction success.

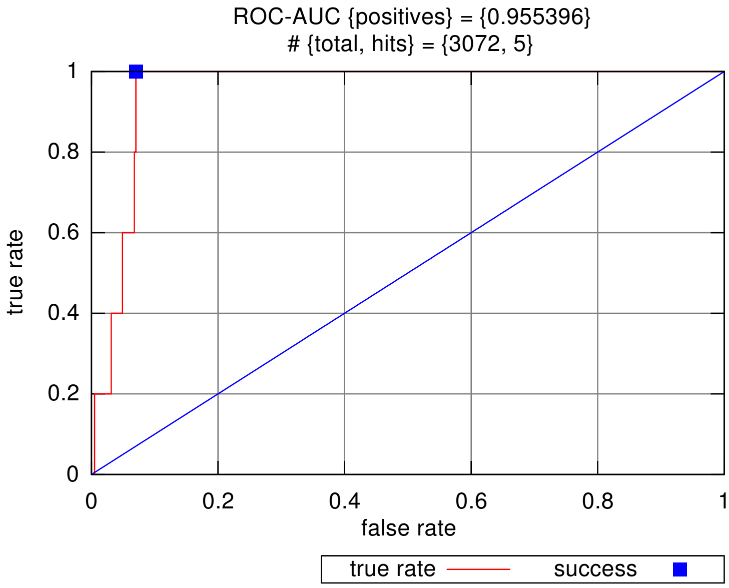

(a)

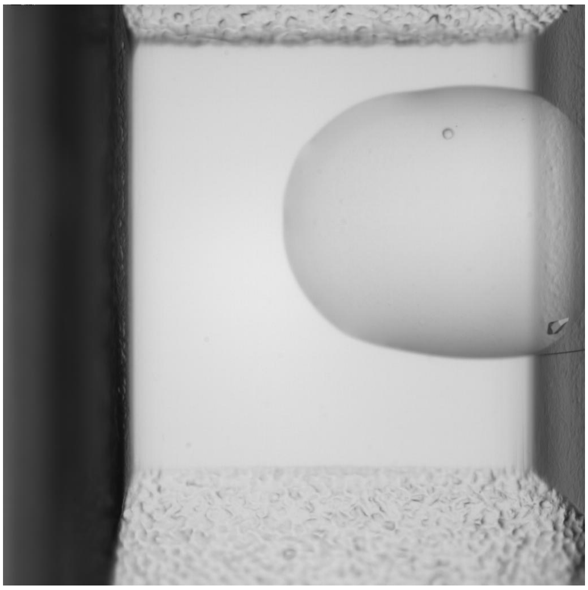

(b)

Figure 44: An ROC Curve for set ‘SpeedET-FG7406A-reu-113-1-113’ along with its highest ranked diffraction success.

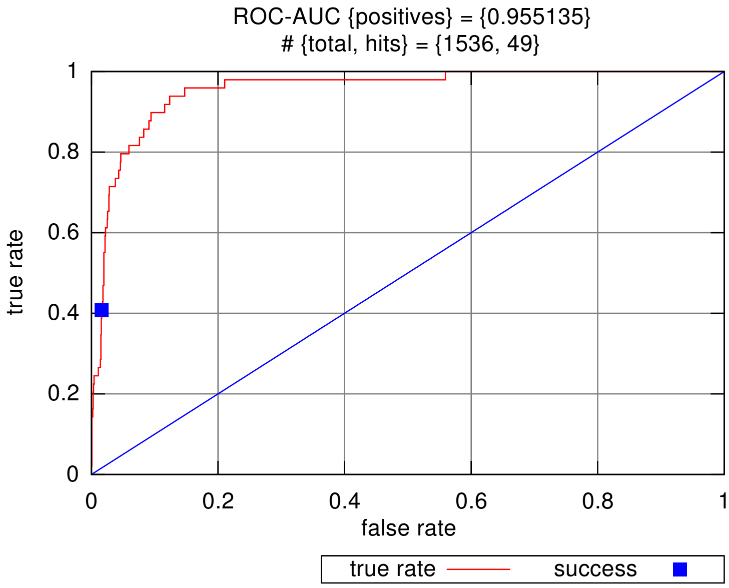

(a)

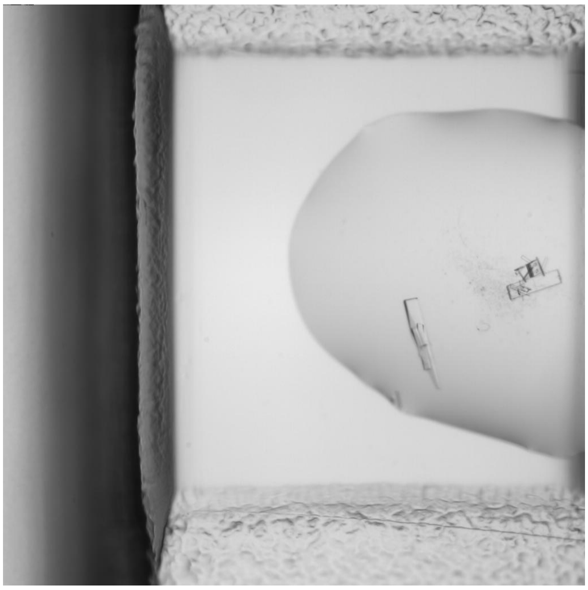

(b)

Figure 45: An ROC Curve for set 'SpeedET-FG7288A-chu-283-1-283' along with its highest ranked diffraction success.

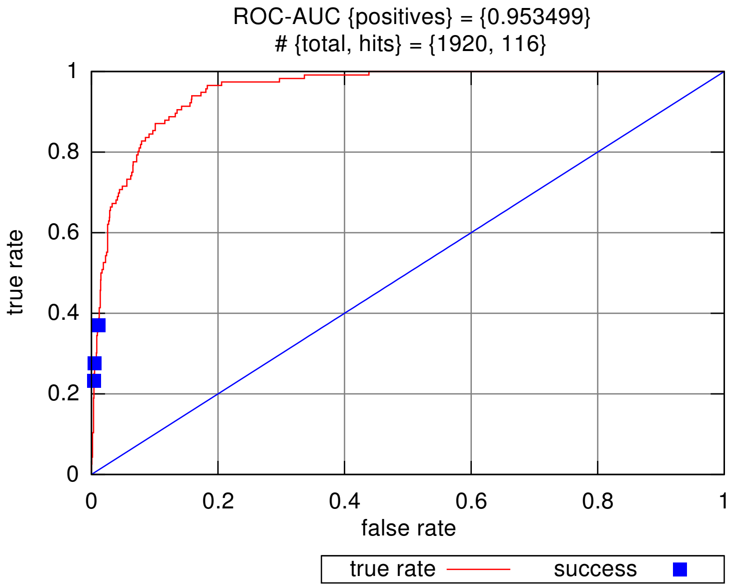

(a)

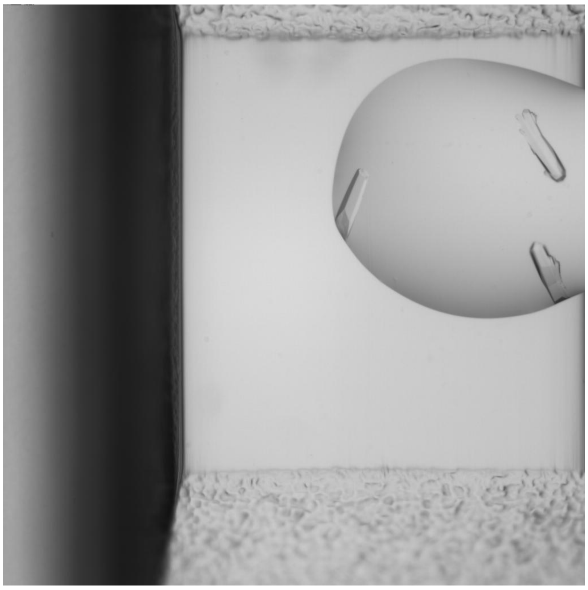

(b)

Figure 46: An ROC Curve for set 'SpeedET-PC06304A-bce-113-14-97' along with its highest ranked diffraction success.

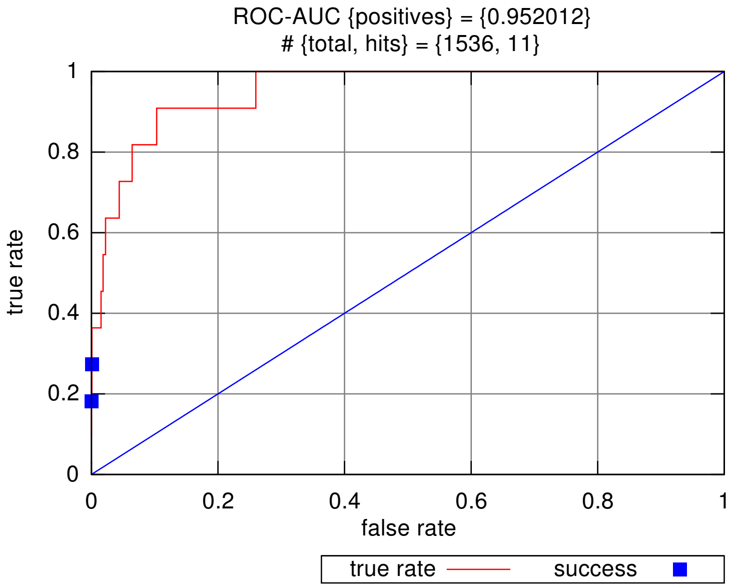

(a)

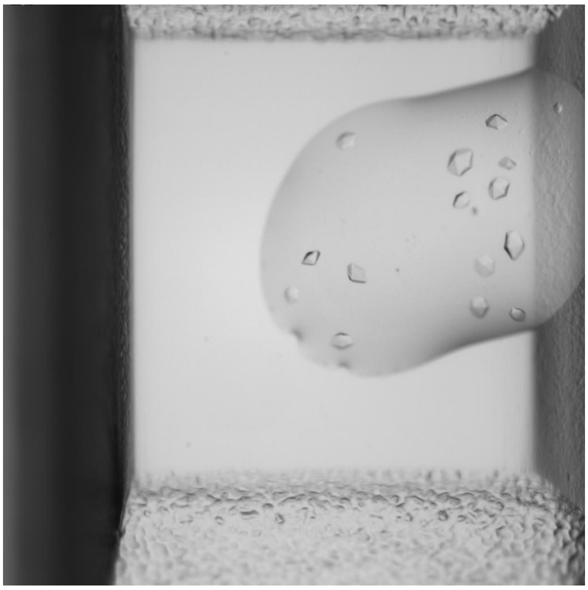

(b)

Figure 47: An ROC Curve for set 'SpeedET-PE00057A-ava-125-1-125' along with its highest ranked diffraction success.

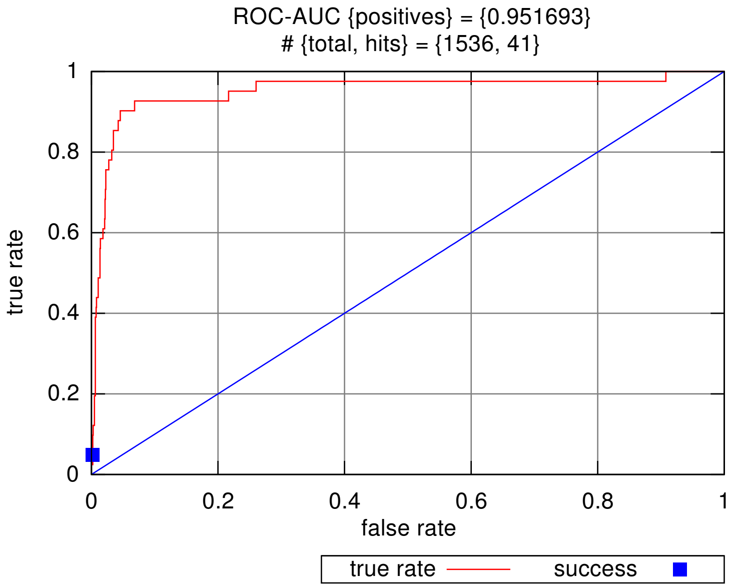

(a)

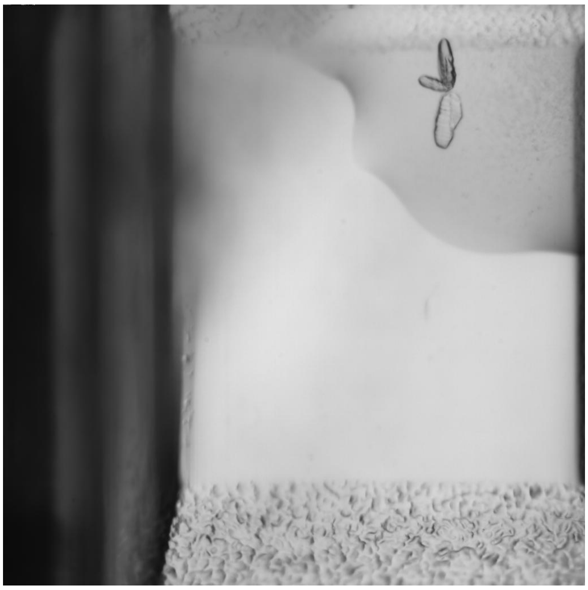

(b)

Figure 48: An ROC Curve for set ‘SpeedET-FJ8586A-ava-395-1-395’ along with its highest ranked diffraction success.

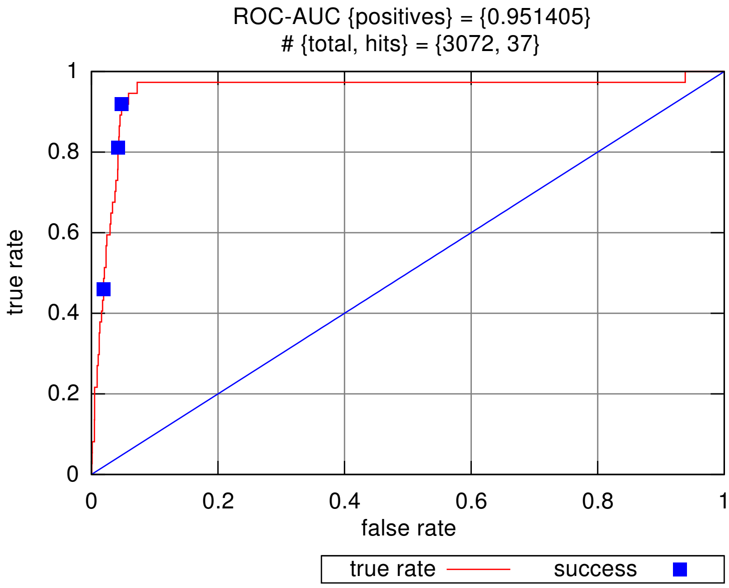

(a)

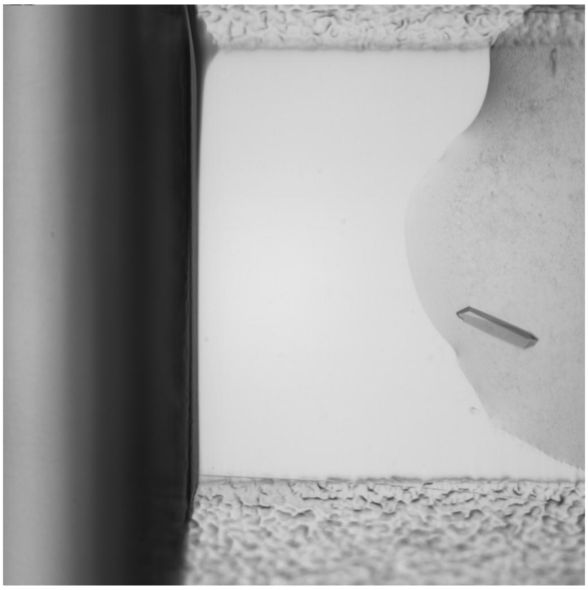

(b)

Figure 49: An ROC Curve for set 'SpeedET-PE00002B-bha-187-1-187' along with its highest ranked diffraction success.

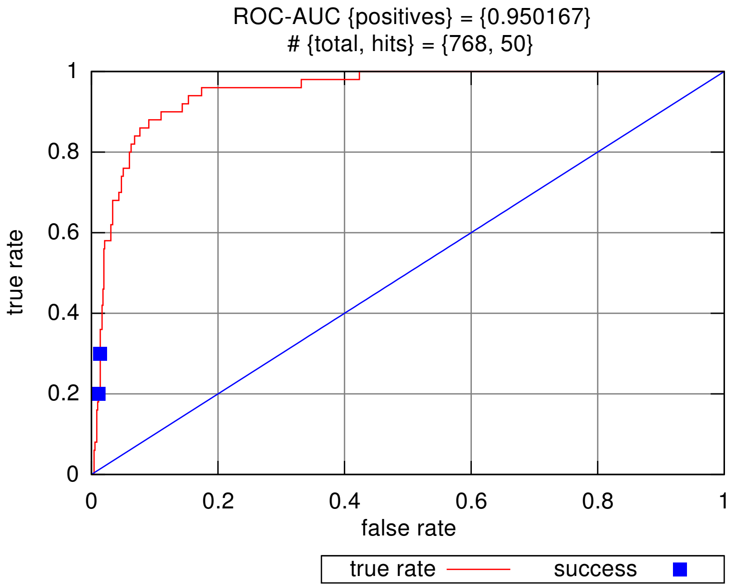

(a)

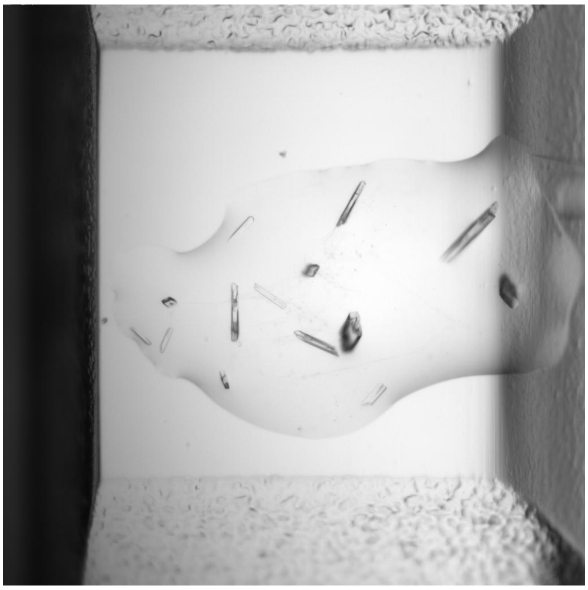

(b)

Figure 50: An ROC Curve for set 'SpeedET-PJ02661S-son-372-1-372-G109C' along with its highest ranked diffraction success.

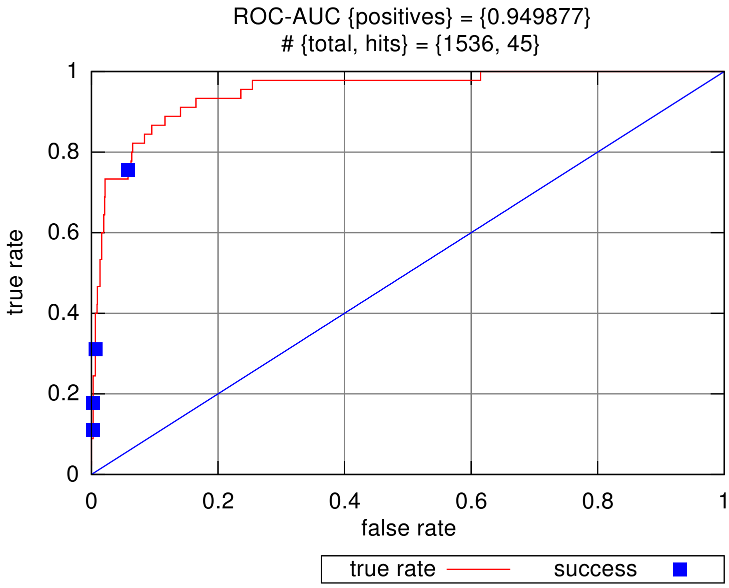

(a)

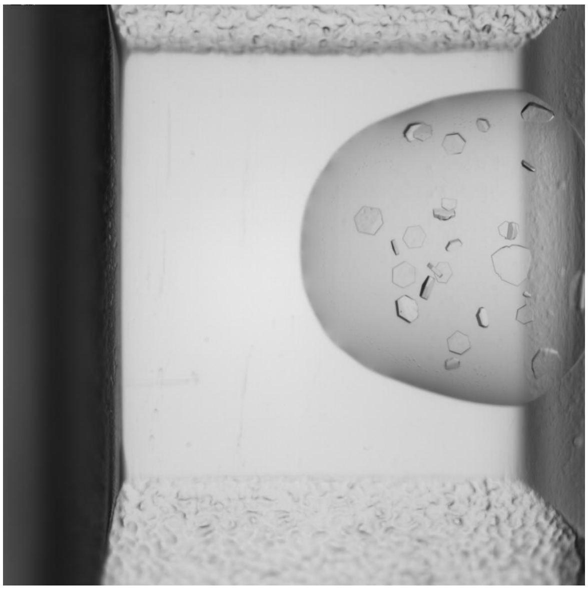

(b)

Figure 51: An ROC Curve for set ‘SpeedET-PE00003C-eca-196-1-196’ along with its highest ranked diffraction success.

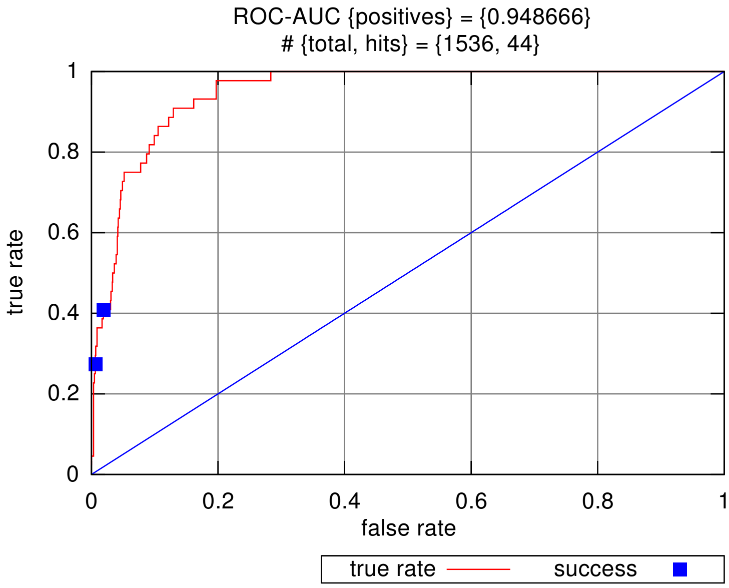

(a)

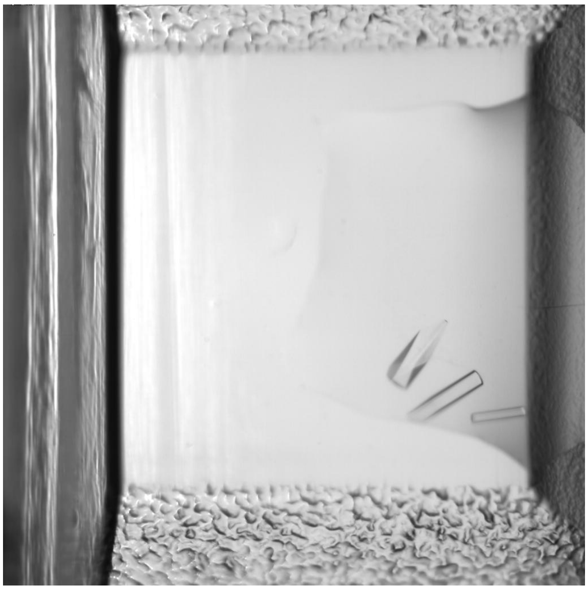

(b)

Figure 52: An ROC Curve for set 'SpeedET-PE00055D-lpl-113-1-113' along with its highest ranked diffraction success.

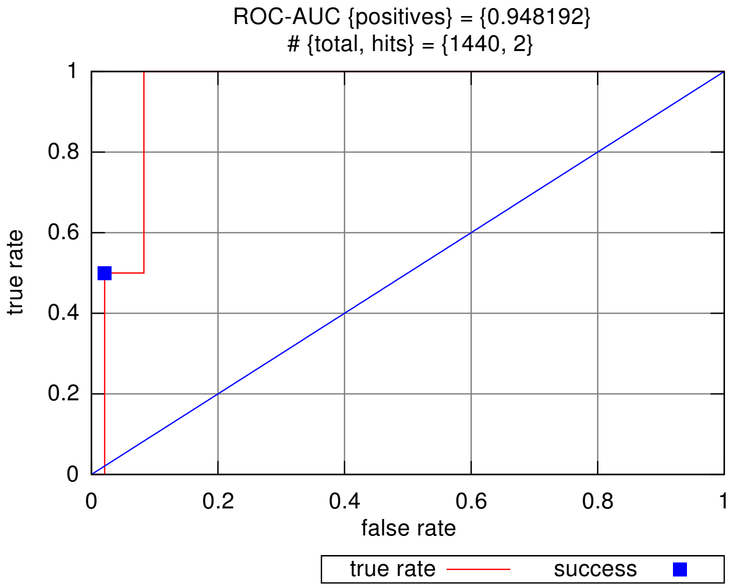

(a)

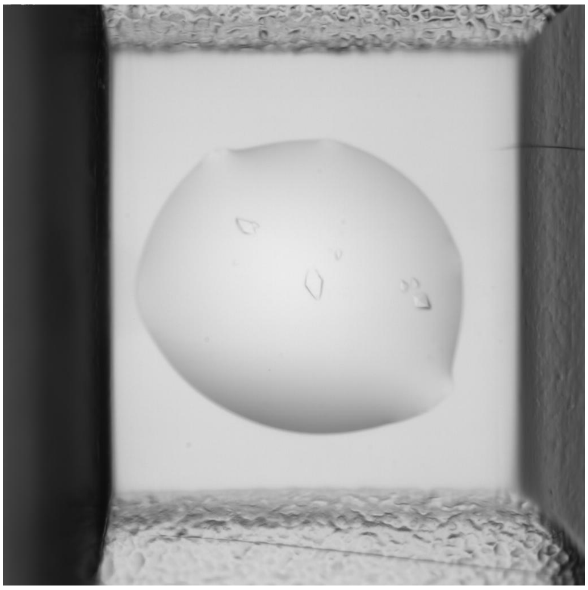

(b)

Figure 53: An ROC Curve for set 'SpeedET-NP_663012.1-ctp-174-1-158' along with its highest ranked diffraction success.

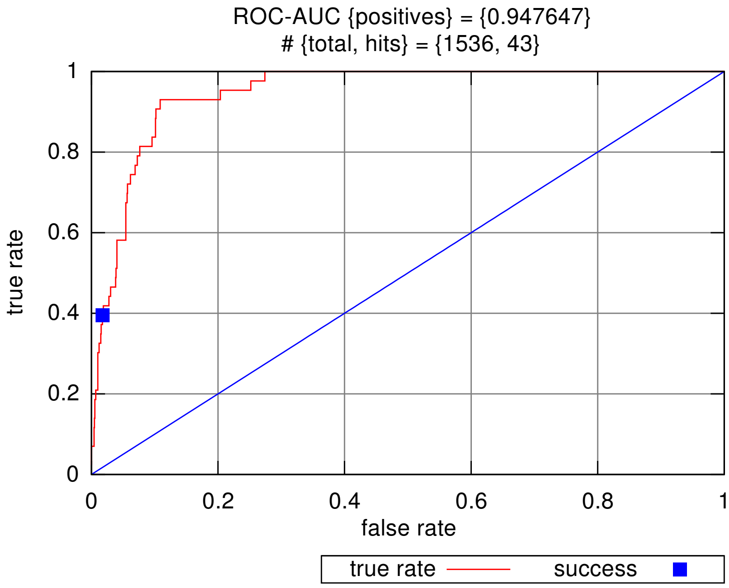

(a)

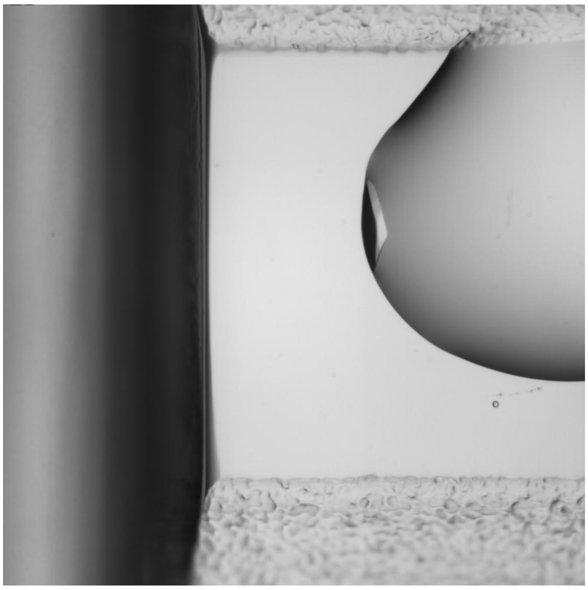

(b)

Figure 54: An ROC Curve for set ‘SpeedET-10175341-bha-295-149-295’ along with its highest ranked diffraction success.

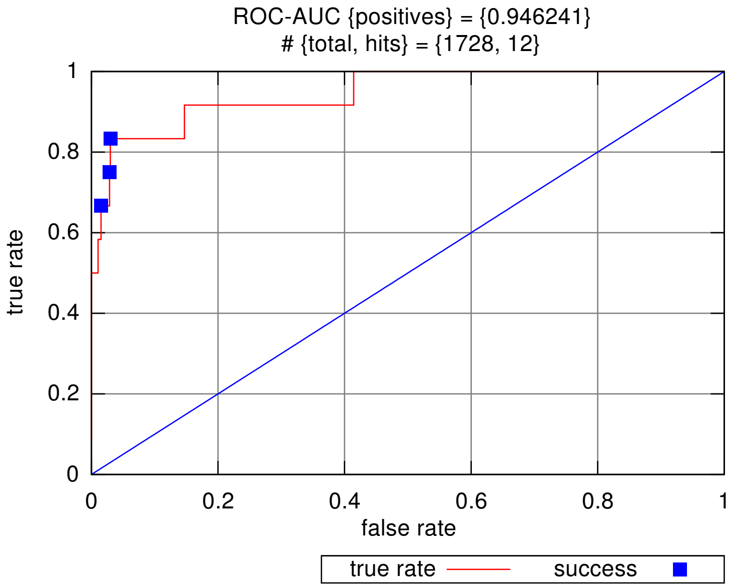

(a)

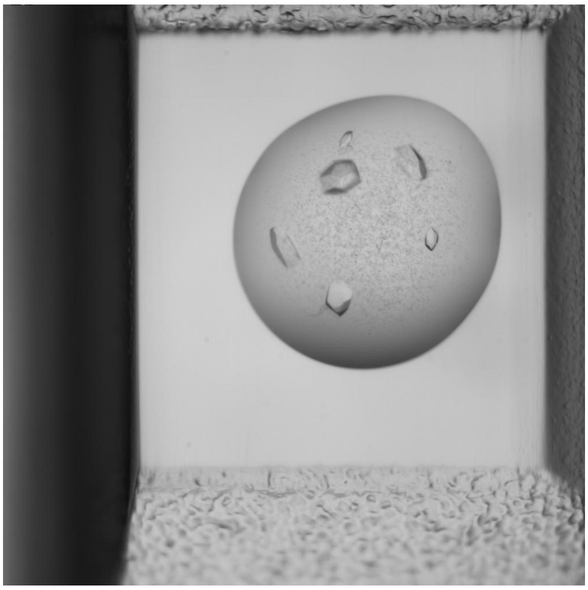

(b)

Figure 55: An ROC Curve for set ‘SpeedET-PG9905A-cac-208-1-208’ along with its highest ranked diffraction success.

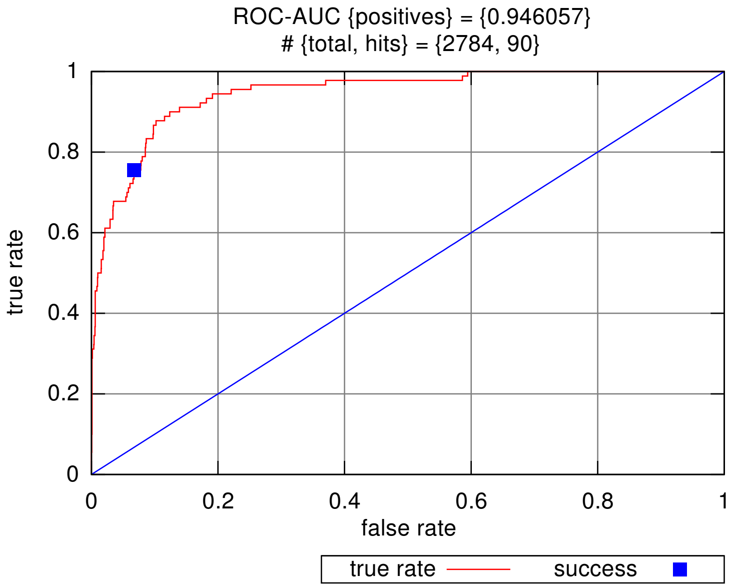

(a)

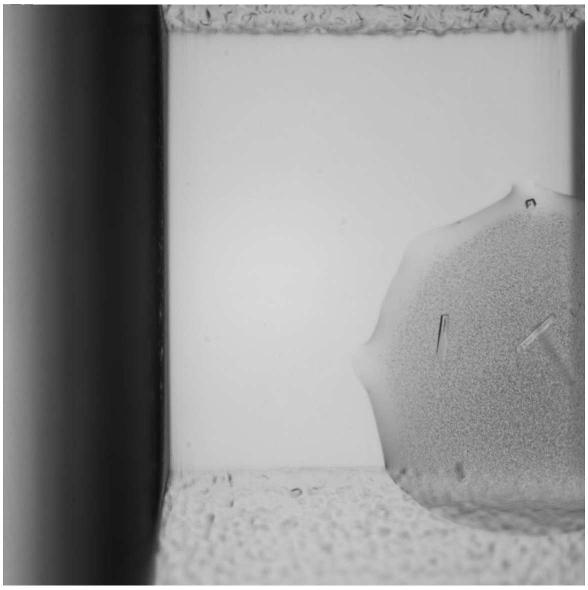

(b)

Figure 56: An ROC Curve for set 'SpeedET-FK9414A-gsu-186-1-186' along with its highest ranked diffraction success.

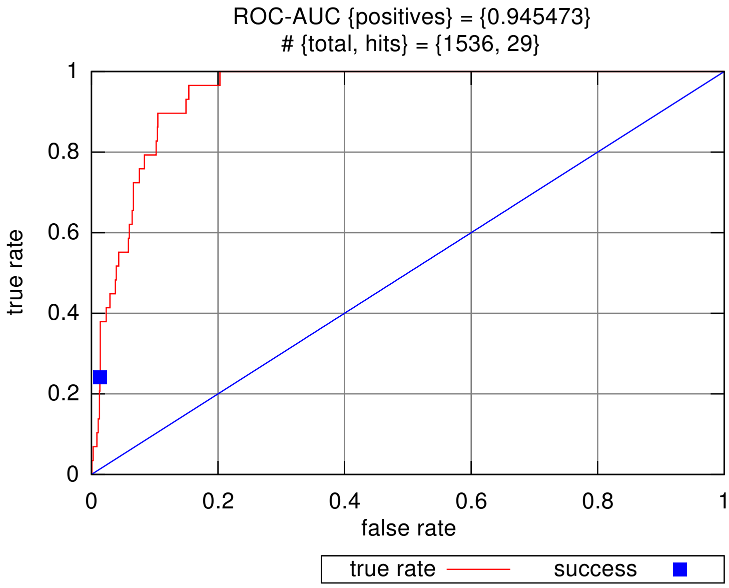

(a)

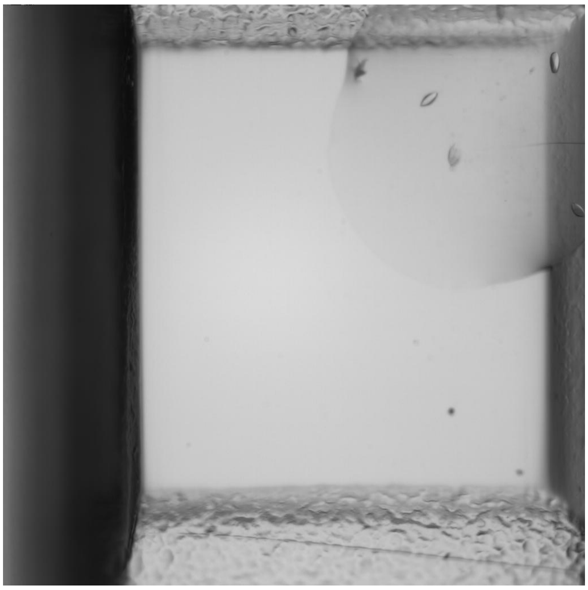

(b)

Figure 57: An ROC Curve for set 'SpeedET-FG7273A-bxe-230-1-230' along with its highest ranked diffraction success.

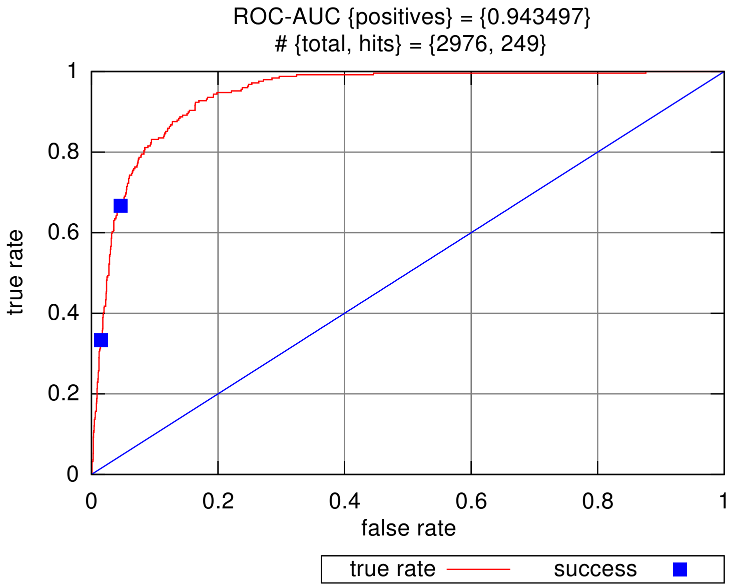

(a)

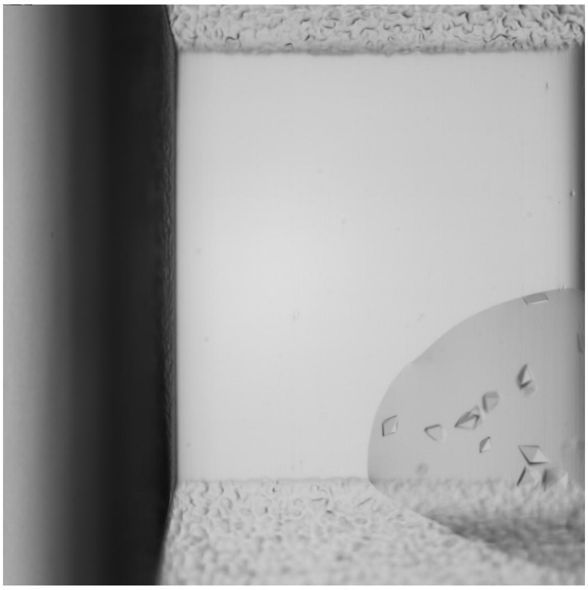

(b)

Figure 58: An ROC Curve for set 'SpeedET-FK9784A-sfr-115-1-115' along with its highest ranked diffraction success.

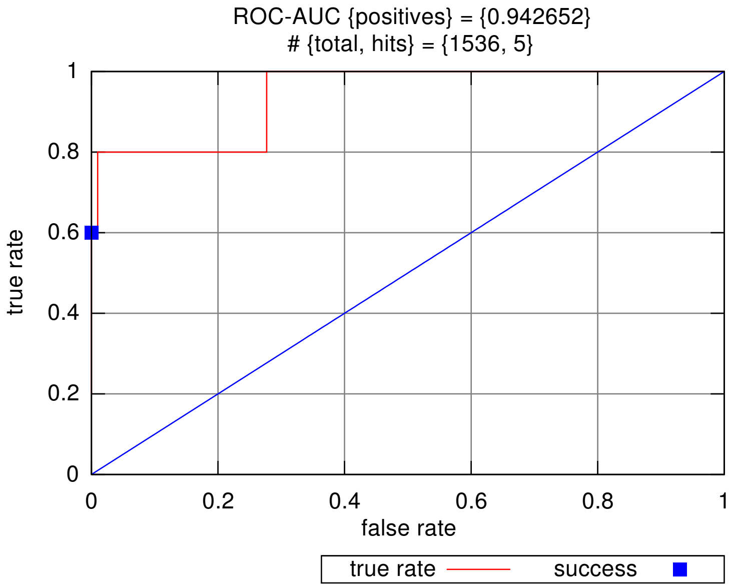

(a)

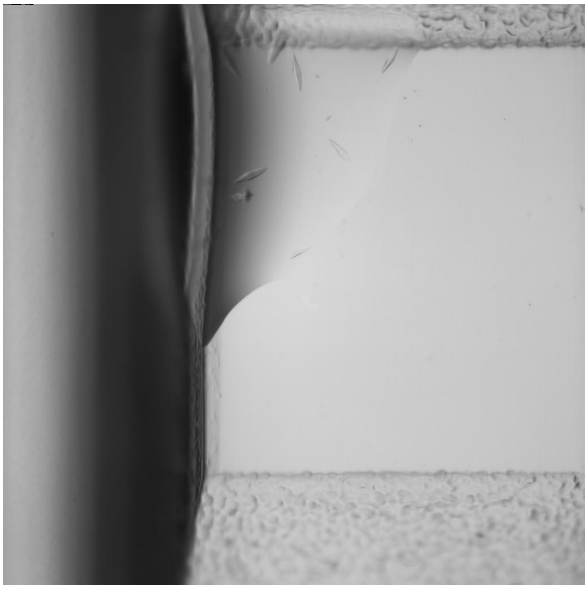

(b)

Figure 59: An ROC Curve for set ‘SpeedET-GN7747A-ocn-115-1-115’ along with its highest ranked diffraction success.

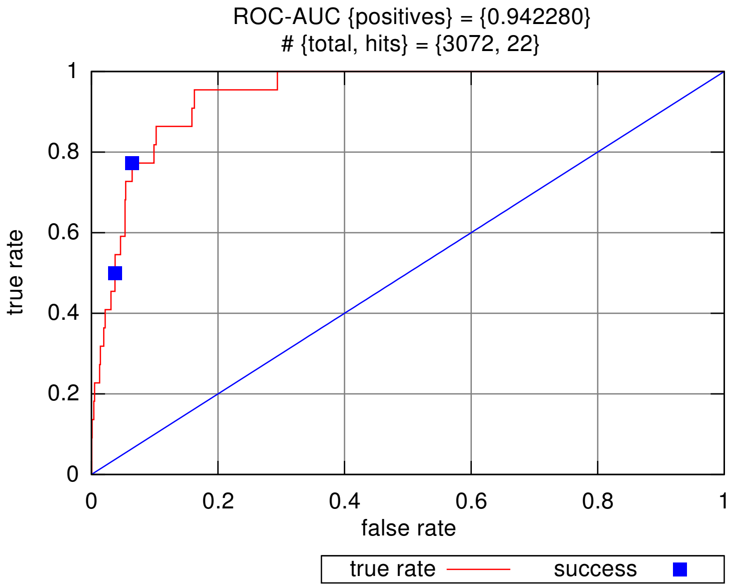

(a)

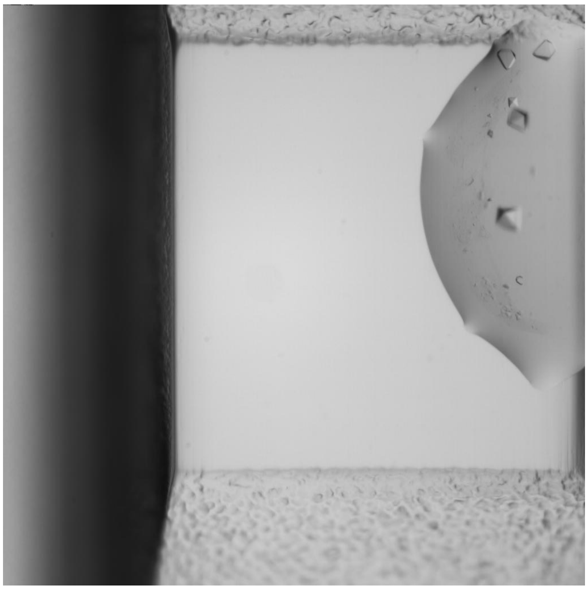

(b)

Figure 60: An ROC Curve for set 'SpeedET-FK9279A-sty-271-1-271' along with its highest ranked diffraction success.

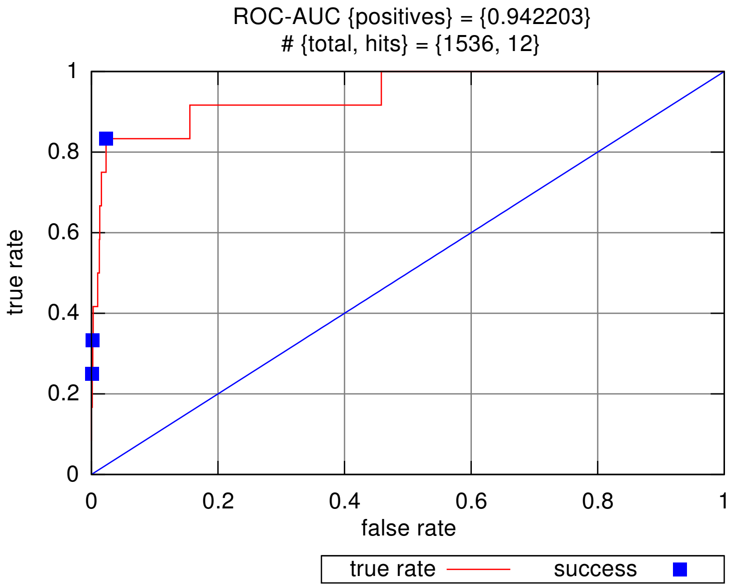

(a)

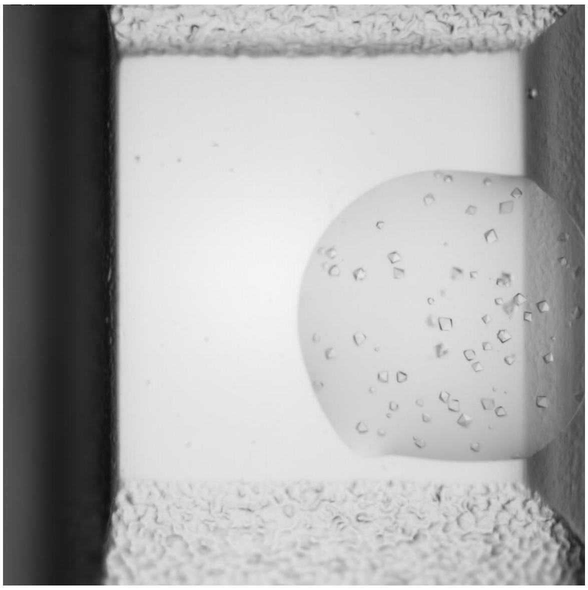

(b)

Figure 61: An ROC Curve for set 'SpeedET-FH7577A-afu-125-1-125' along with its highest ranked diffraction success.

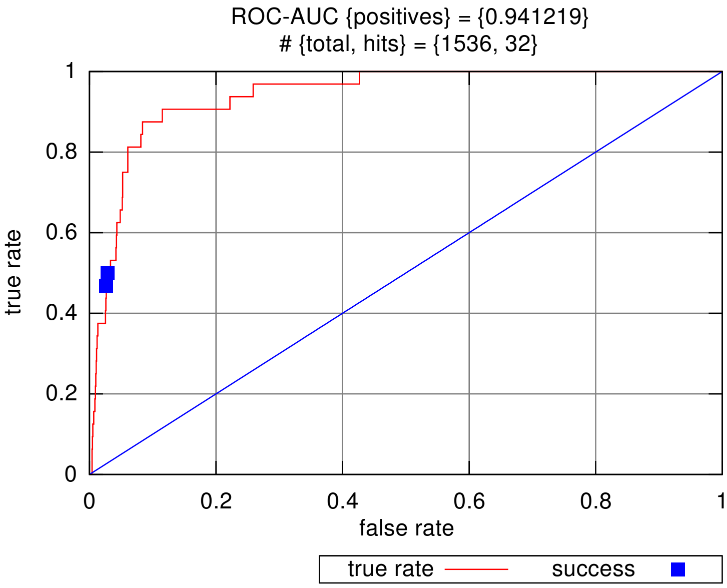

(a)

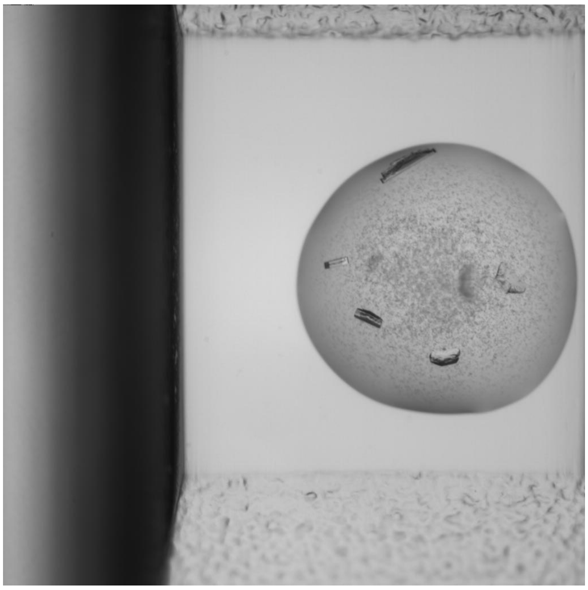

(b)

Figure 62: An ROC Curve for set ‘SpeedET-FJ5490A-rru-137-1-137’ along with its highest ranked diffraction success.

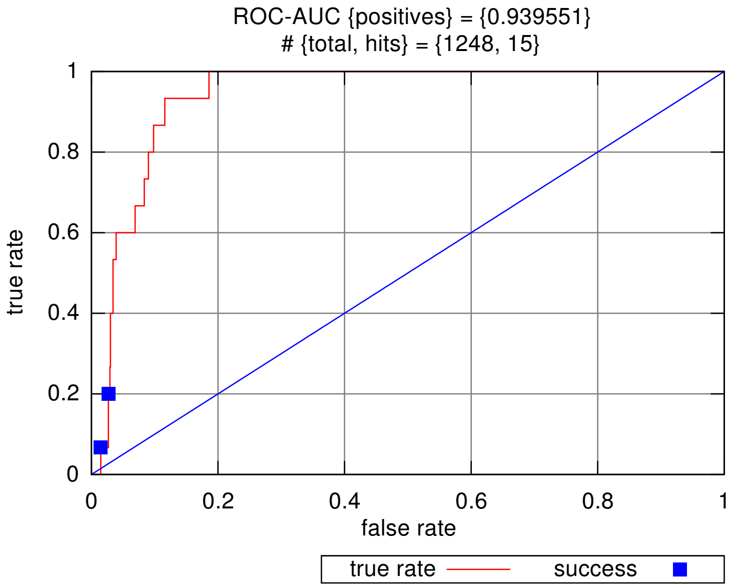

(a)

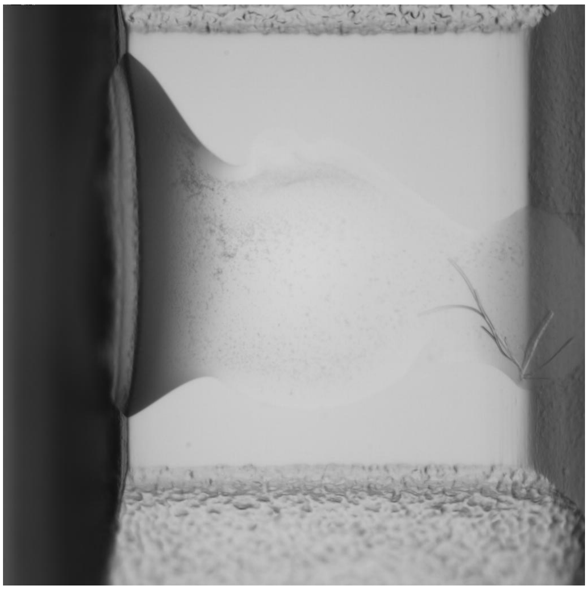

(b)

Figure 63: An ROC Curve for set 'SpeedET-PJ05013D-reu-289-1-289' along with its highest ranked diffraction success.

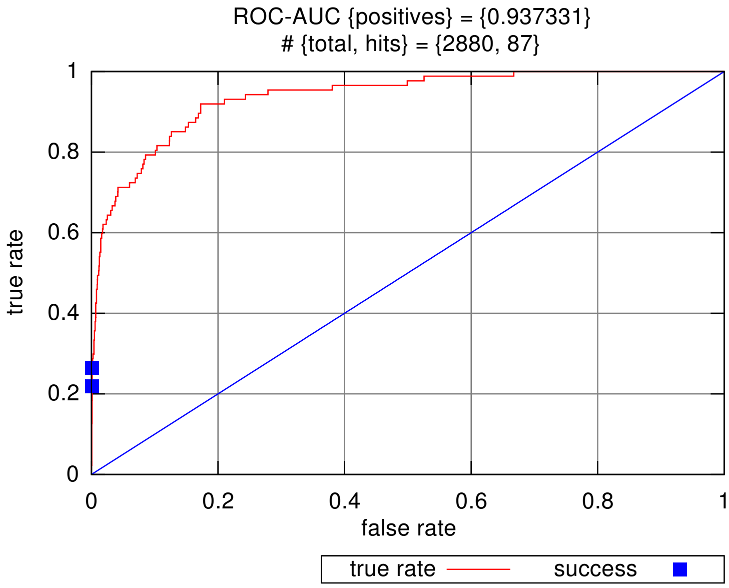

(a)

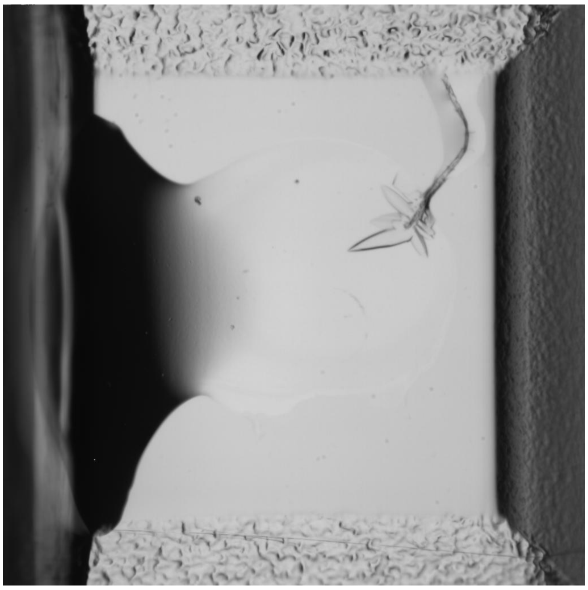

(b)

Figure 64: An ROC Curve for set 'SpeedET-10173191-bha-184-1-184' along with its highest ranked diffraction success.

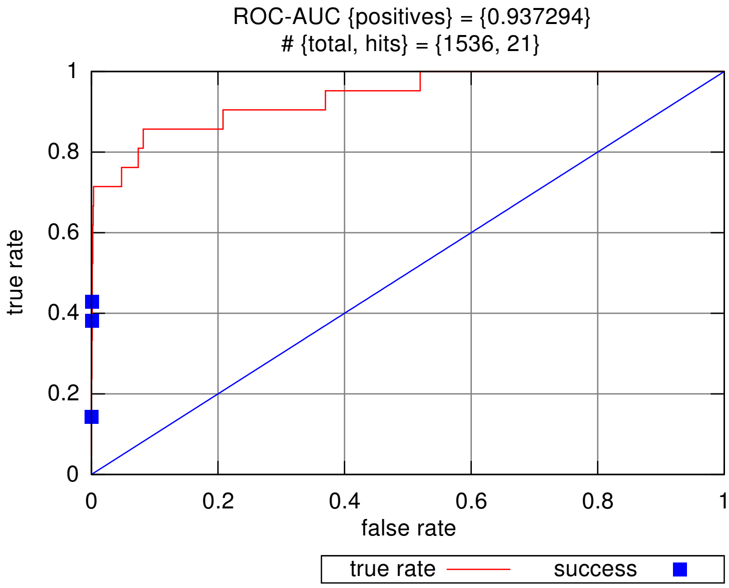

(a)

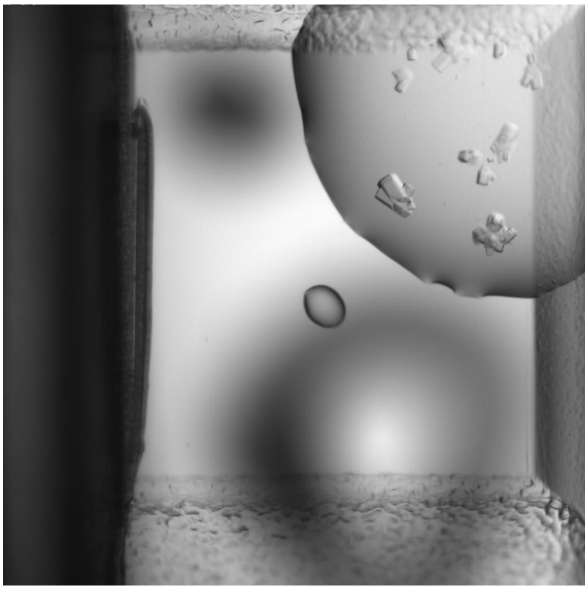

(b)

Figure 65: An ROC Curve for set ‘SpeedET-RK10652A-bha-331-1-331’ along with its highest ranked diffraction success.

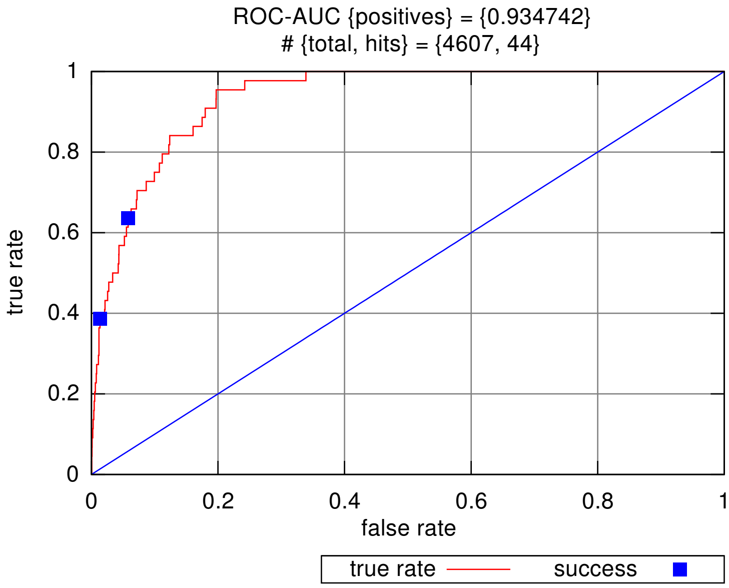

(a)

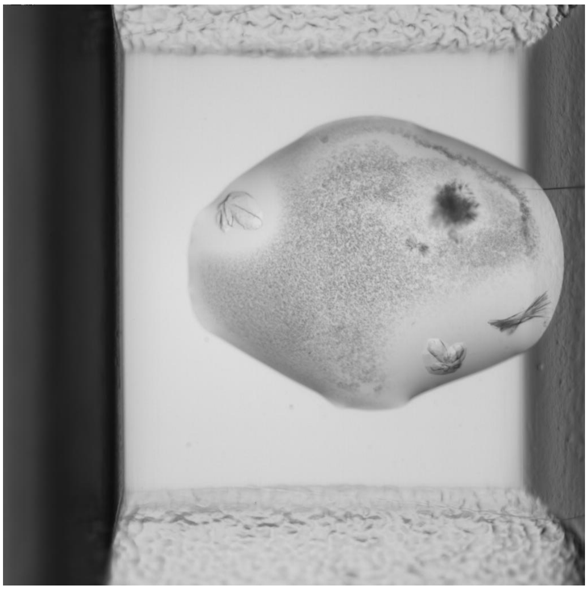

(b)

Figure 66: An ROC Curve for set ‘SpeedET-FG7283A-cdi-269-1-269’ along with its highest ranked diffraction success.

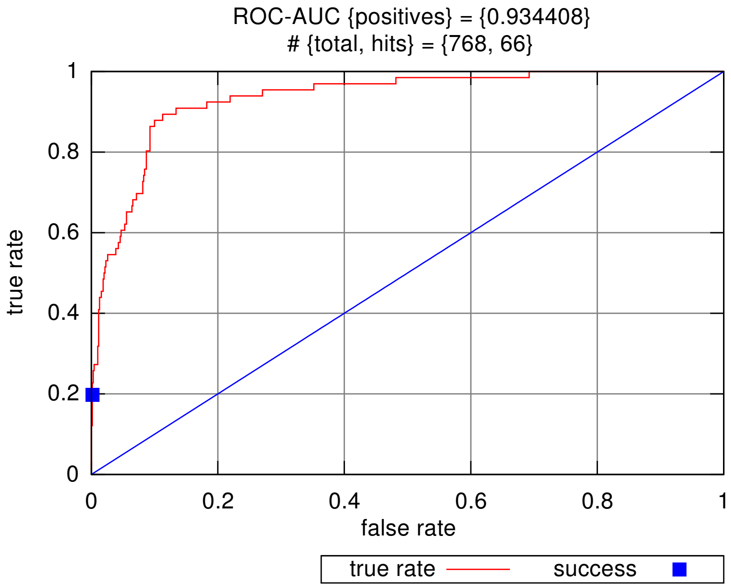

(a)

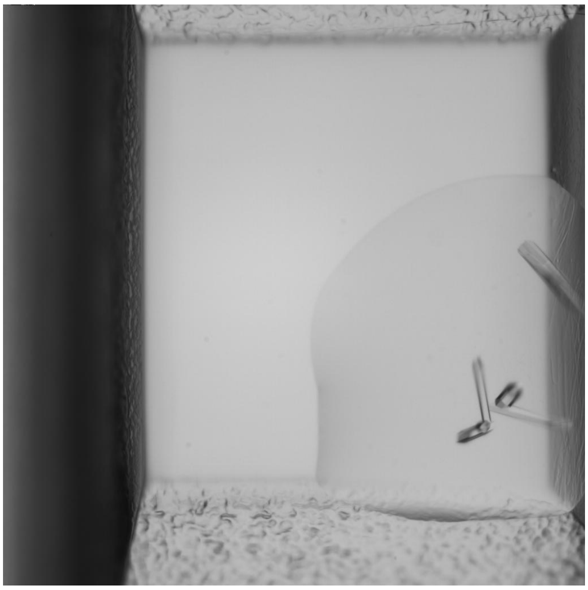

(b)

Figure 67: An ROC Curve for set ‘SpeedET-PJ04820A-sfr-525-1-525’ along with its highest ranked diffraction success.

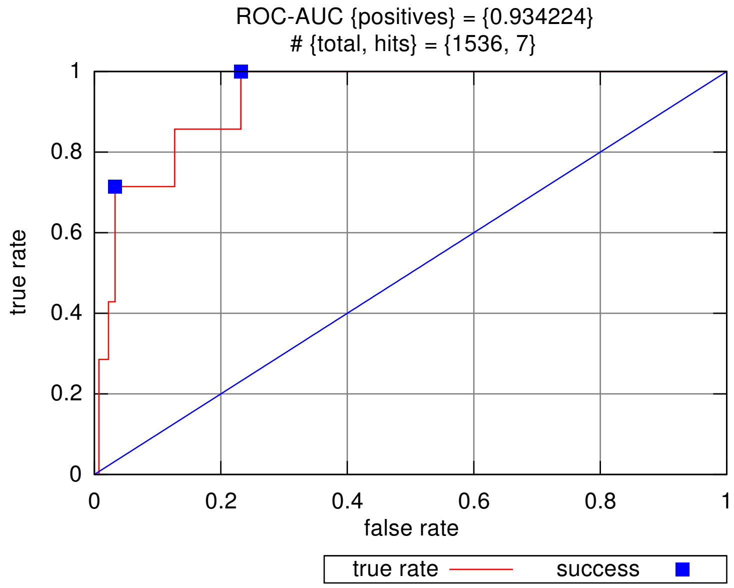

(a)

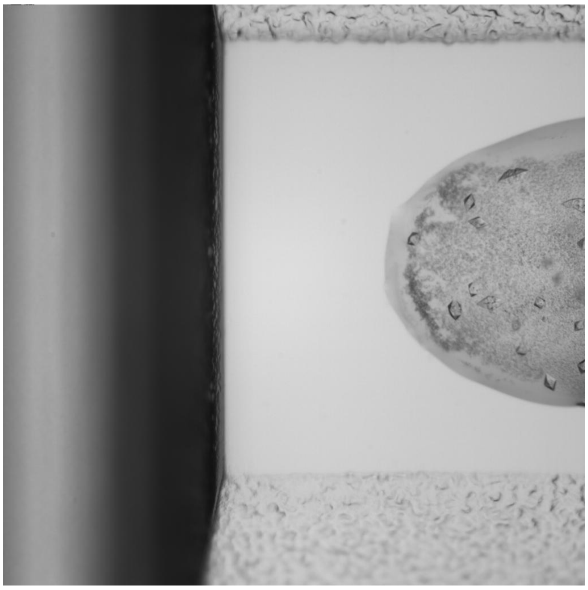

(b)

Figure 68: An ROC Curve for set 'SpeedET-PC02830C-mja-162-1-162-K132Y-K133Y-K134Y' along with its highest ranked diffraction success.

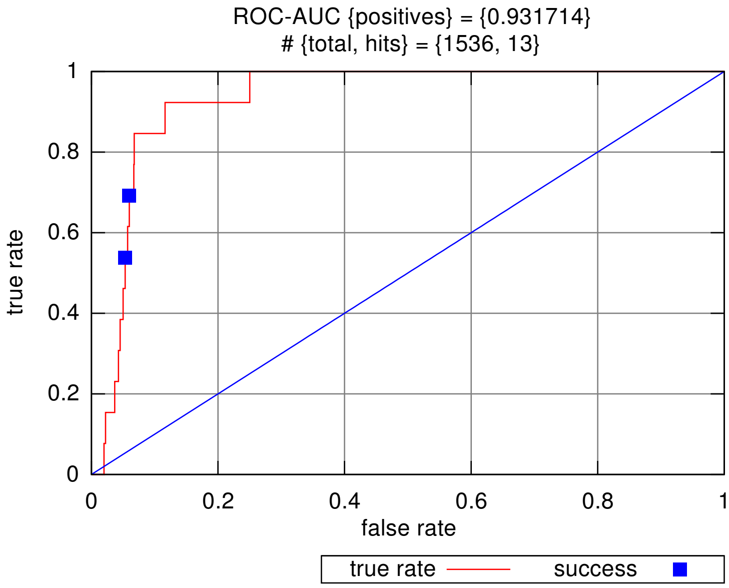

(a)

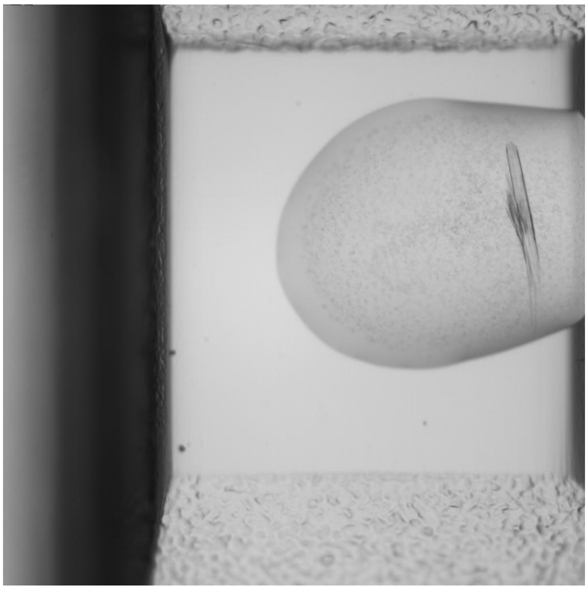

(b)

Figure 69: An ROC Curve for set 'SpeedET-PE00057B-ava-113-1-113' along with its highest ranked diffraction success.

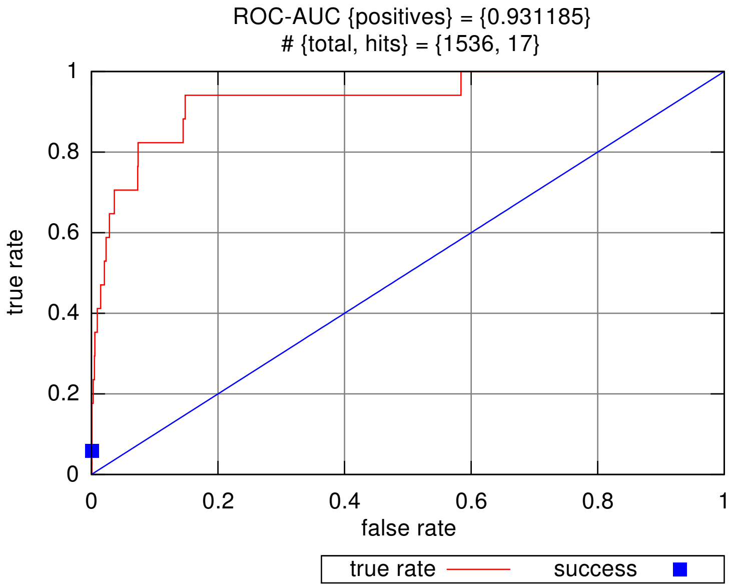

(a)

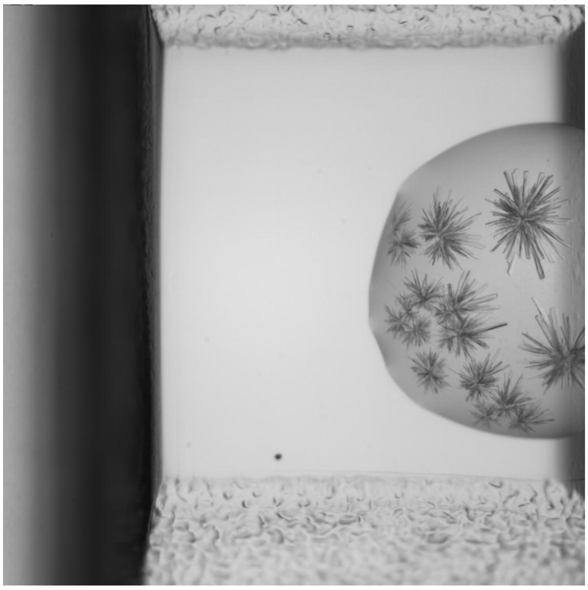

(b)

Figure 70: An ROC Curve for set 'SpeedET-FJ9503A-bxe-262-1-262' along with its highest ranked diffraction success.

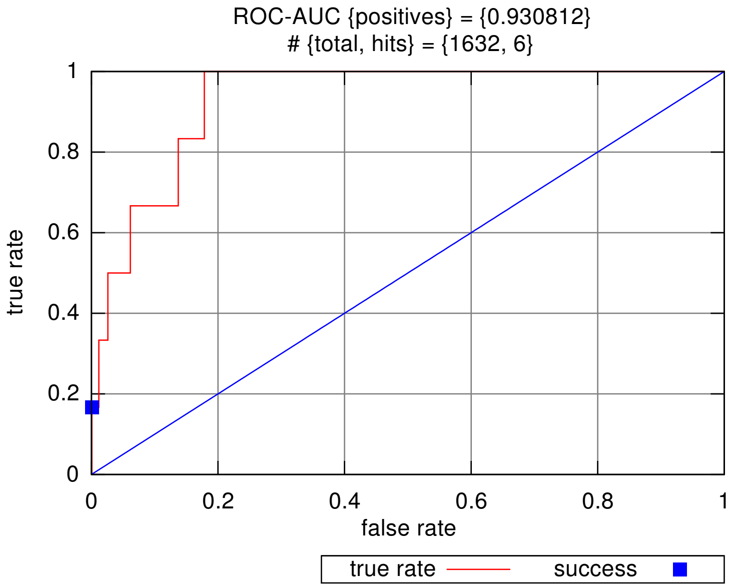

(a)

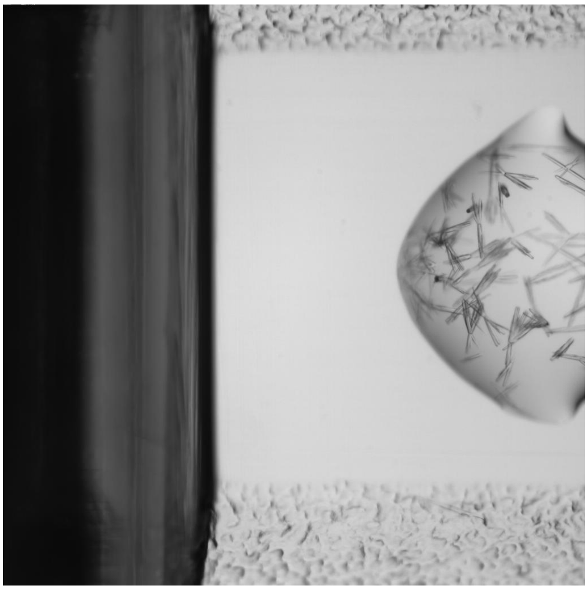

(b)

Figure 71: An ROC Curve for set 'SpeedET-PG8359F-bxe-128-1-128' along with its highest ranked diffraction success.

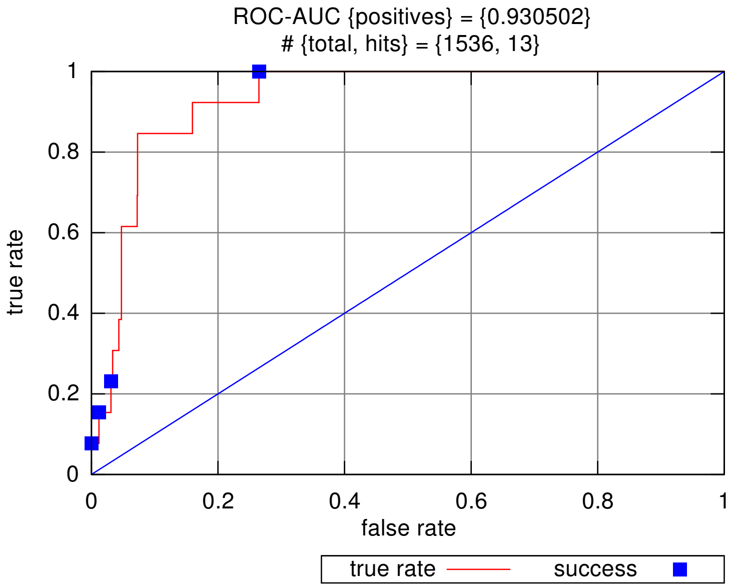

(a)

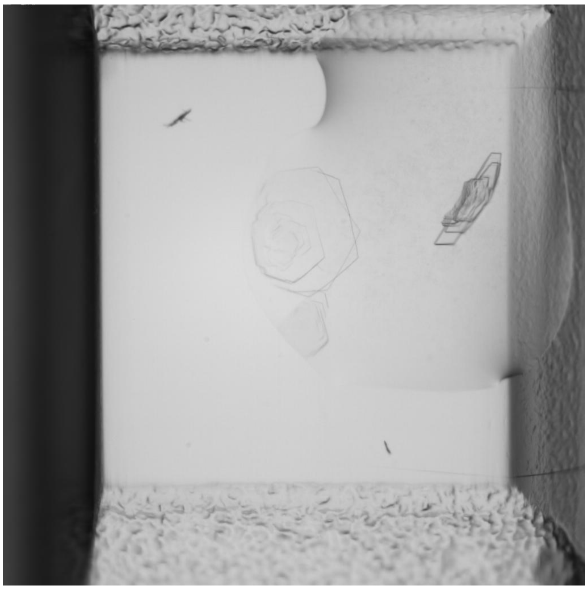

(b)

Figure 72: An ROC Curve for set 'SpeedET-FH7486A-npu-147-1-147' along with its highest ranked diffraction success.

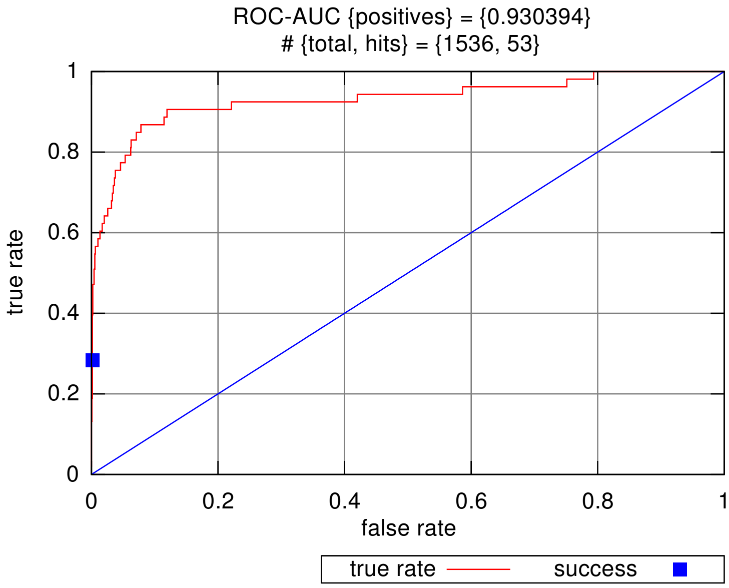

(a)

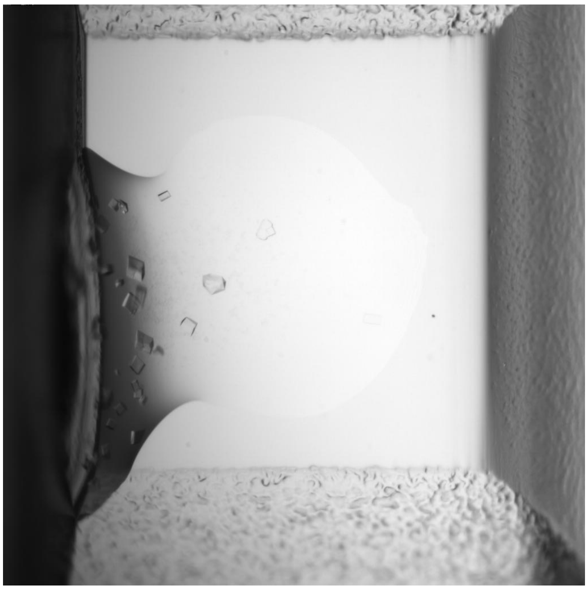

(b)

Figure 73: An ROC Curve for set 'SpeedET-PJ04222C-sba-199-1-199' along with its highest ranked diffraction success.

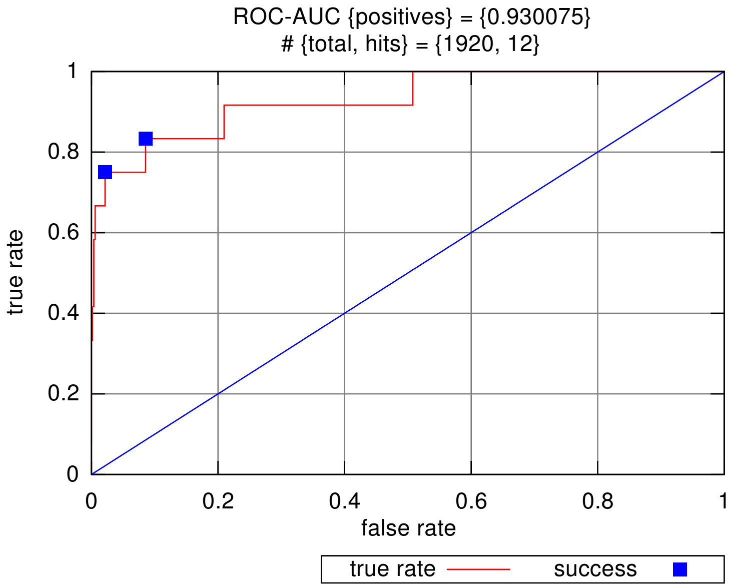

(a)

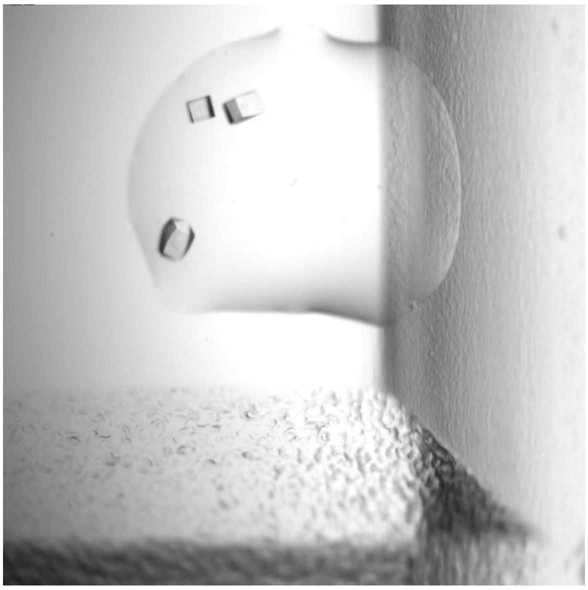

(b)

Figure 74: An ROC Curve for set ‘SpeedET-PJ05163B-bsu-159-1-159’ along with its highest ranked diffraction success.

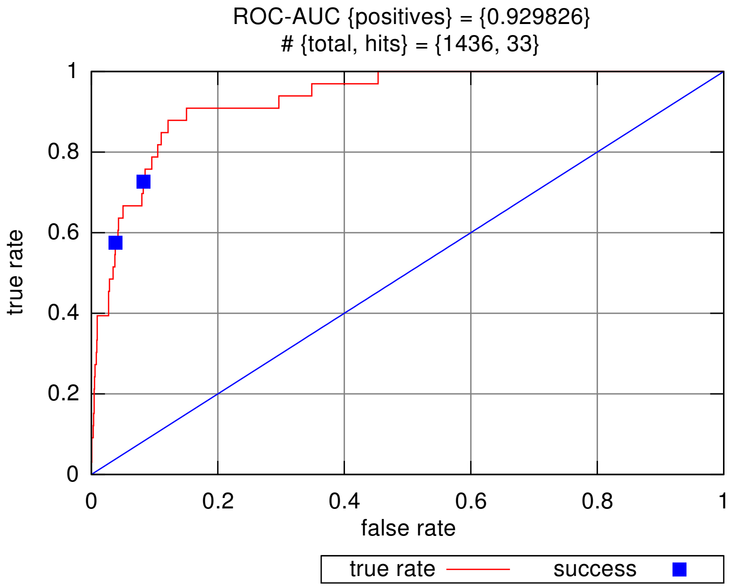

(a)

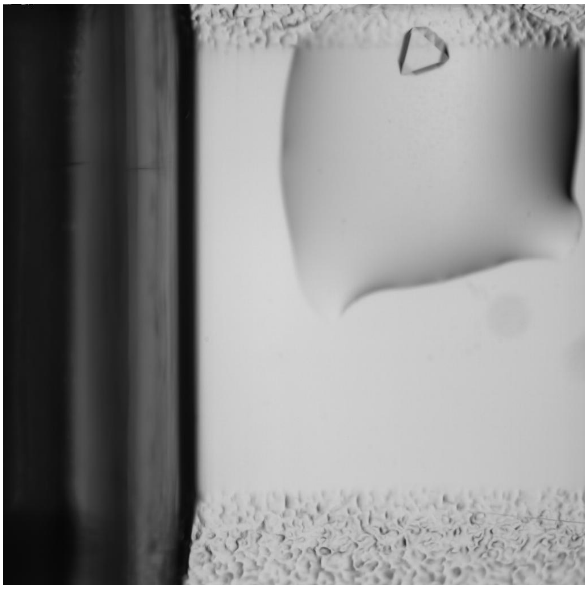

(b)

Figure 75: An ROC Curve for set 'SpeedET-FJ8786A-pma-243-1-243' along with its highest ranked diffraction success.

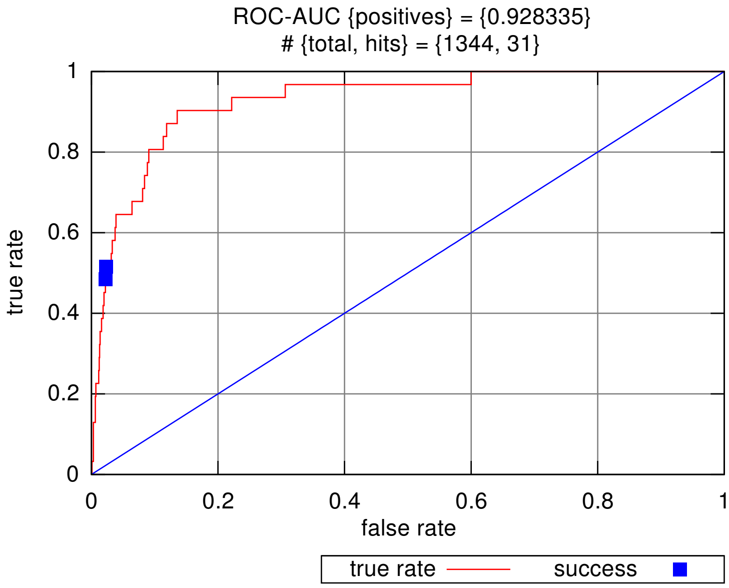

(a)

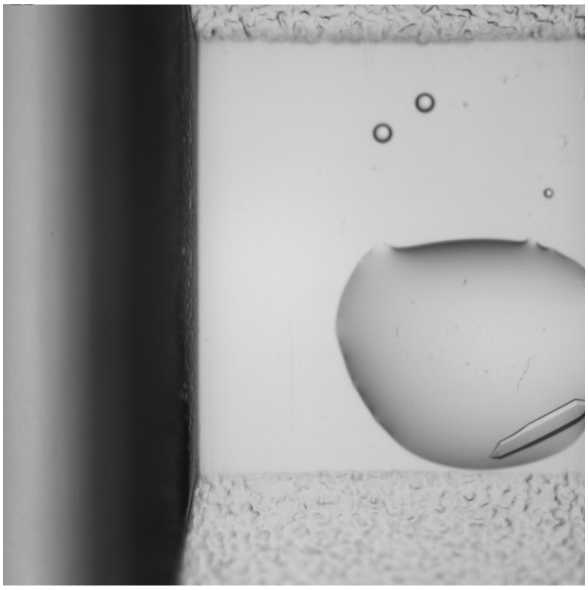

(b)

Figure 76: An ROC Curve for set ‘SpeedET-FJ9546A-sag-162-1-162’ along with its highest ranked diffraction success.

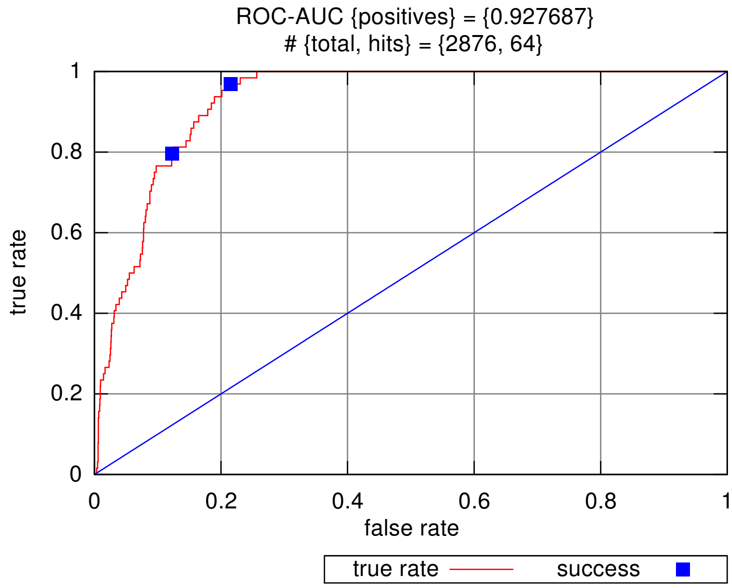

(a)

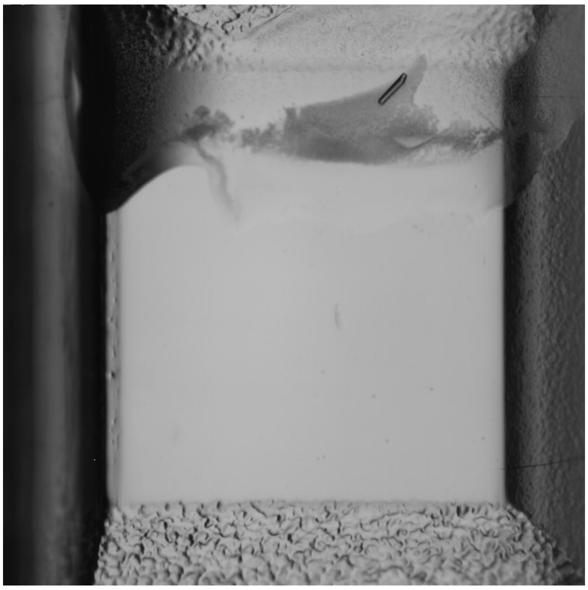

(b)

Figure 77: An ROC Curve for set 'SpeedET-FK8798A-lmo-153-1-153' along with its highest ranked diffraction success.

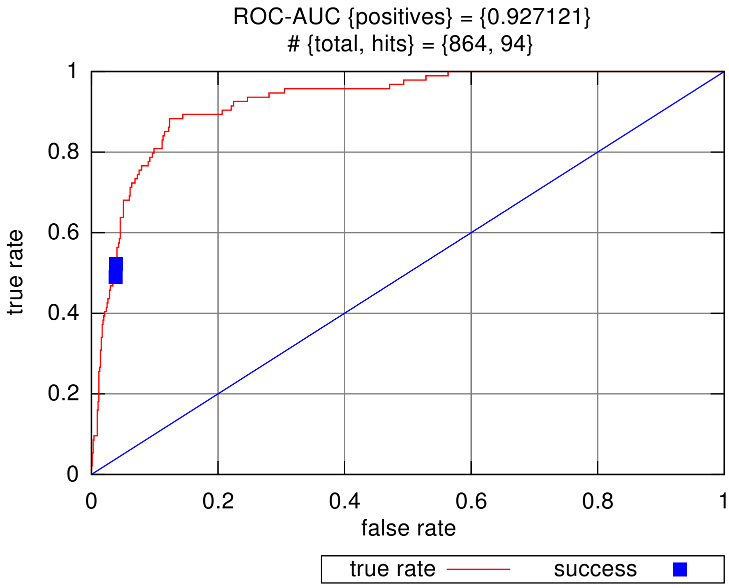

(a)

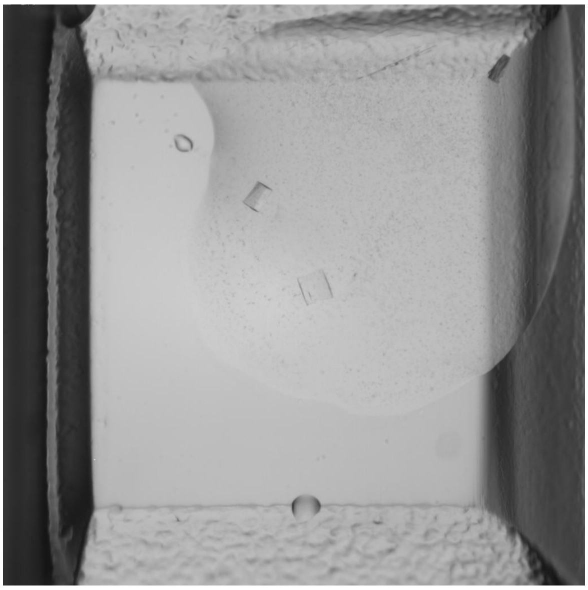

(b)

Figure 78: An ROC Curve for set ‘SpeedET-PC06175E-ppu-205-1-205’ along with its highest ranked diffraction success.

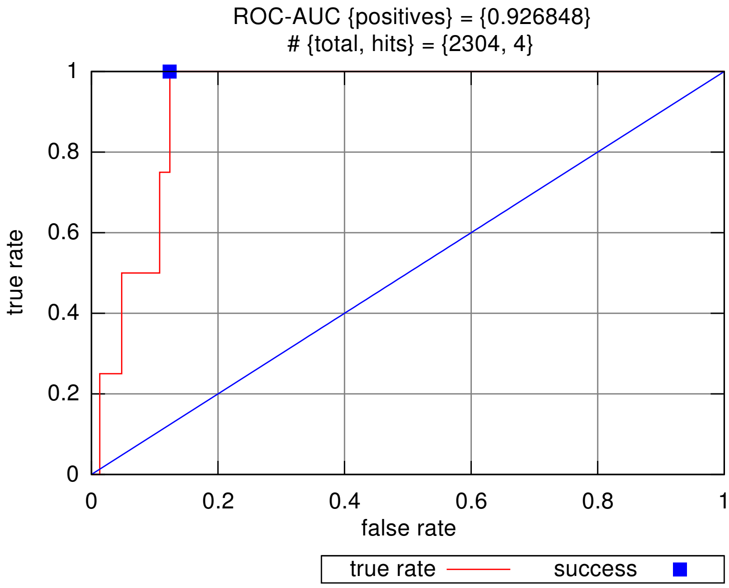

(a)

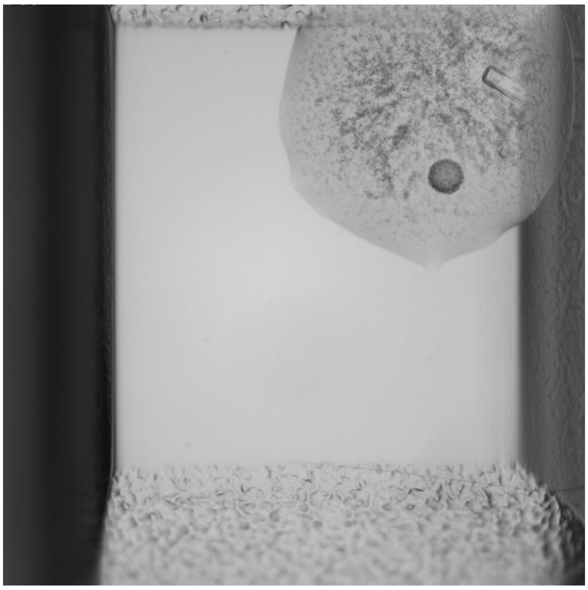

(b)

Figure 79: An ROC Curve for set 'SpeedET-FJ9548A-reu-140-1-140' along with its highest ranked diffraction success.

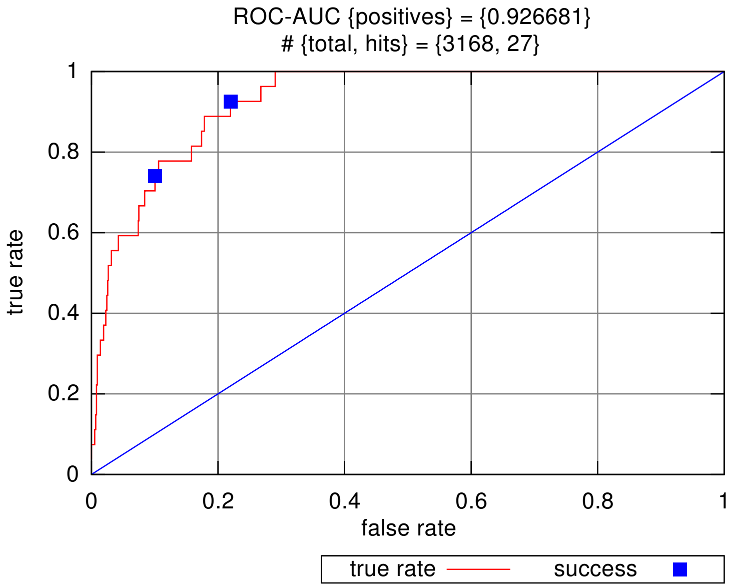

(a)

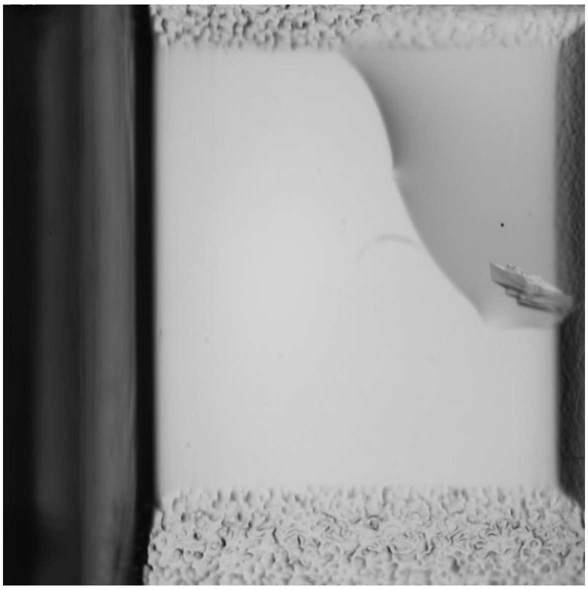

(b)

Figure 80: An ROC Curve for set 'SpeedET-FJ9446A-jsp-174-1-174' along with its highest ranked diffraction success.

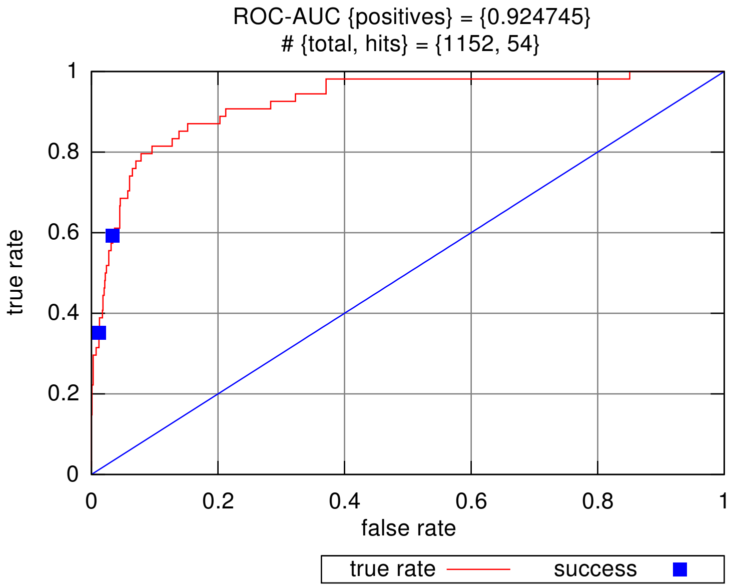

(a)

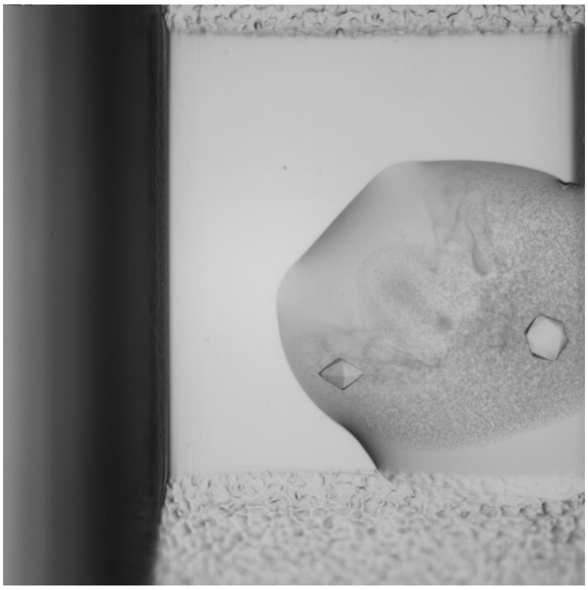

(b)

Figure 81: An ROC Curve for set ‘SpeedET-FJ9319A-cgl-203-1-203-A29T’ along with its highest ranked diffraction success.

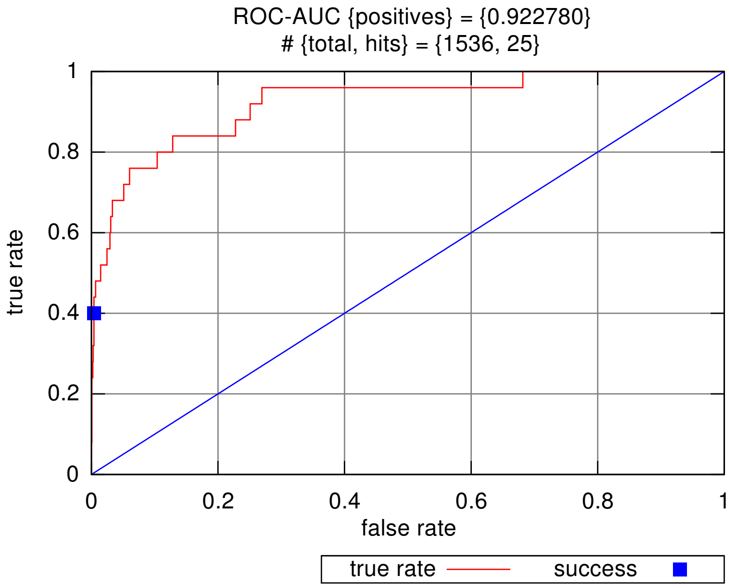

(a)

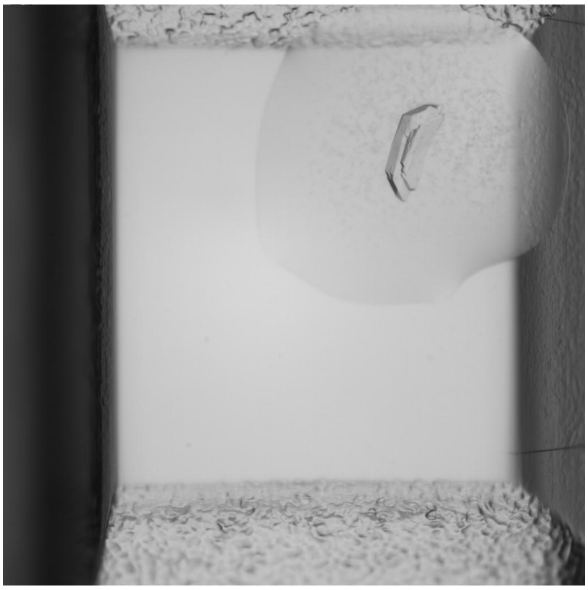

(b)

Figure 82: An ROC Curve for set 'SpeedET-FK9665C-ssp-159-1-159' along with its highest ranked diffraction success.

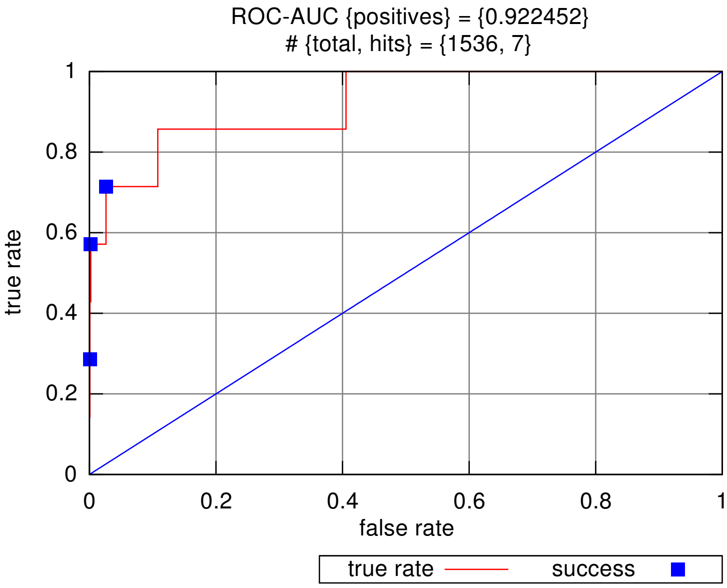

(a)

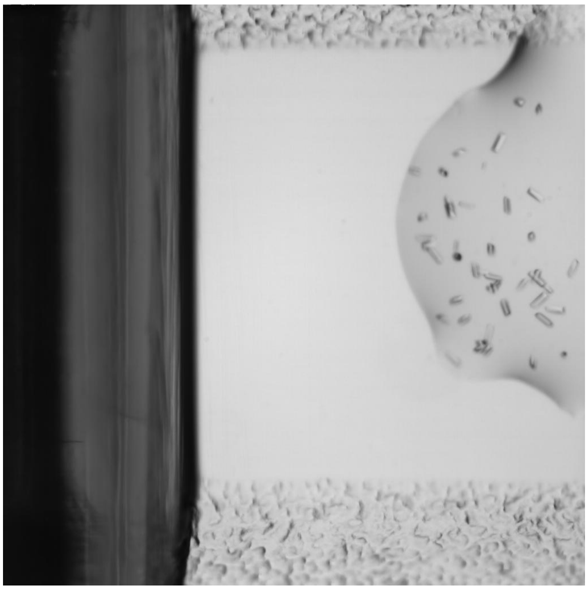

(b)

Figure 83: An ROC Curve for set ‘SpeedET-FJ9219A-ava-222-1-222’ along with its highest ranked diffraction success.

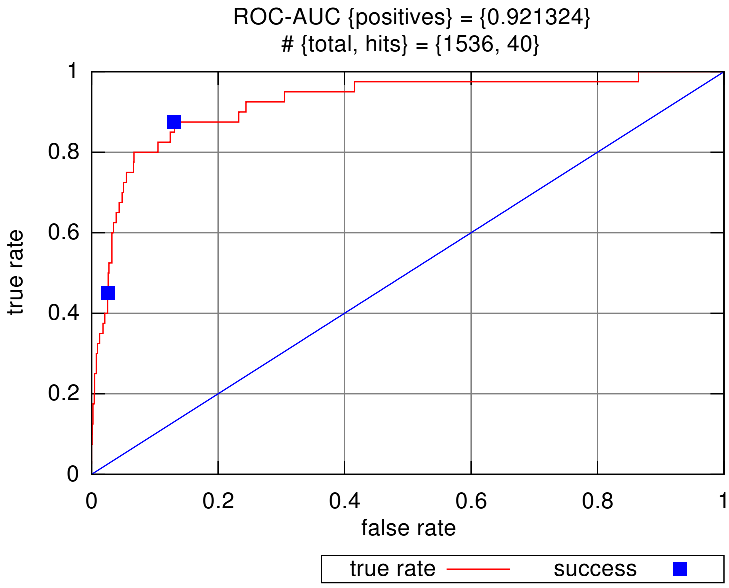

(a)

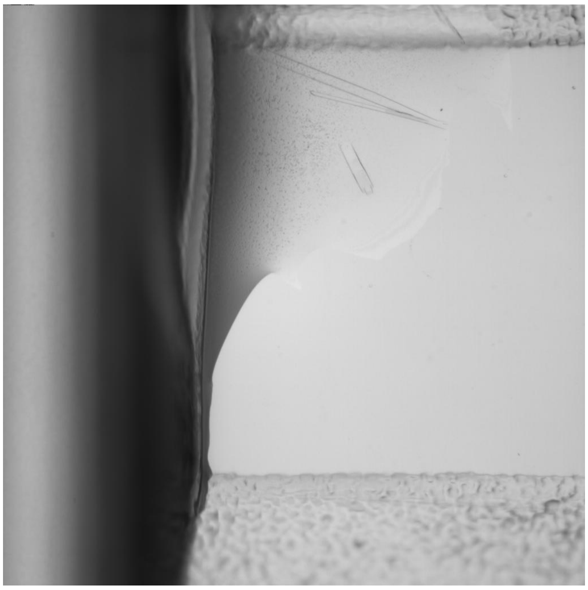

(b)

Figure 84: An ROC Curve for set ‘SpeedET-GN7773A-ocn-206-1-206’ along with its highest ranked diffraction success.

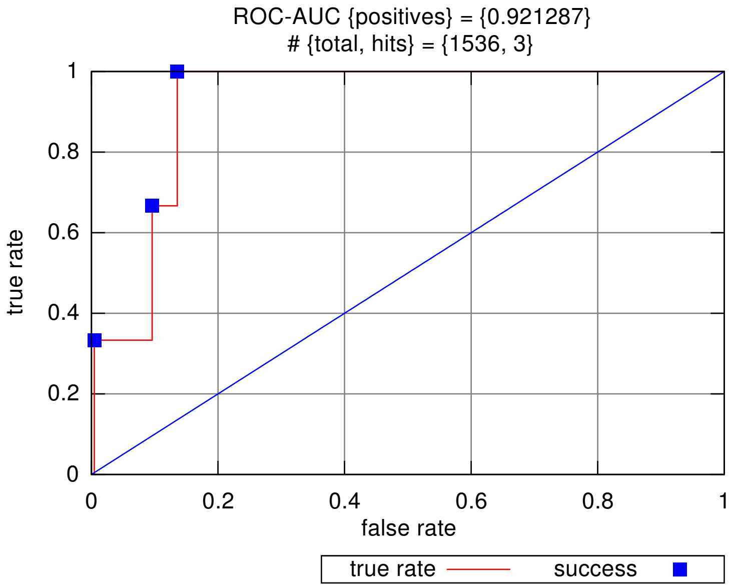

(a)

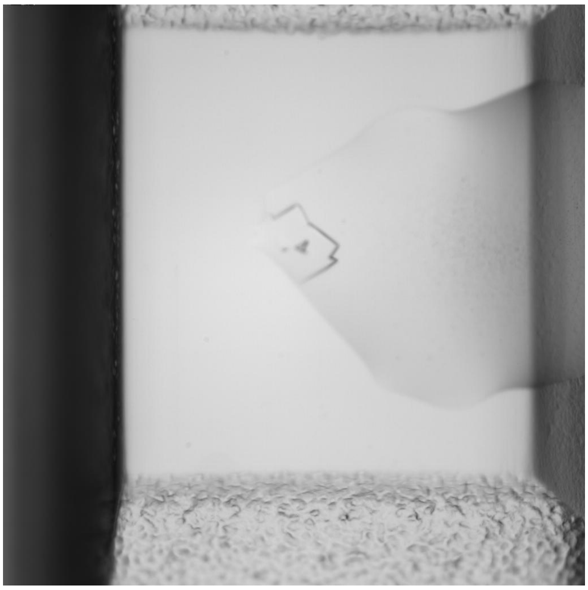

(b)

Figure 85: An ROC Curve for set 'SpeedET-PG9878A-son-140-1-140' along with its highest ranked diffraction success.

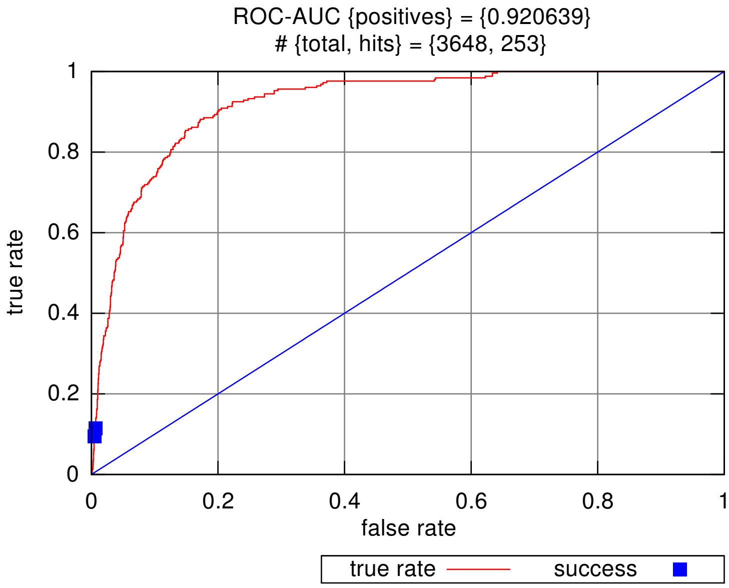

(a)

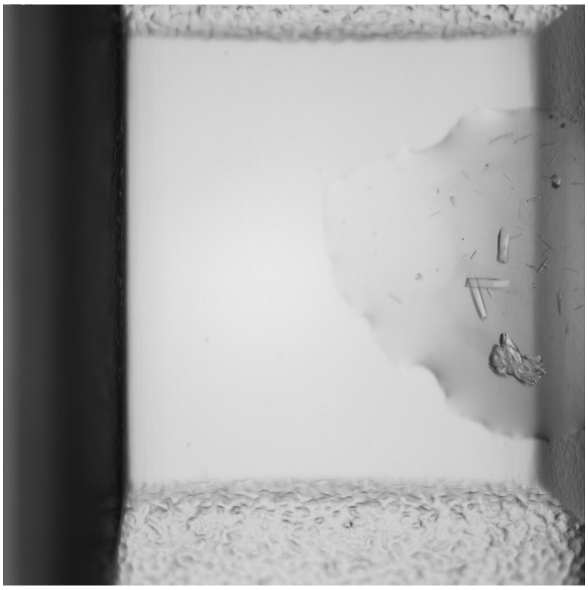

(b)

Figure 86: An ROC Curve for set ‘SpeedET-PD01933B-bha-322-1-322-V7I’ along with its highest ranked diffraction success.

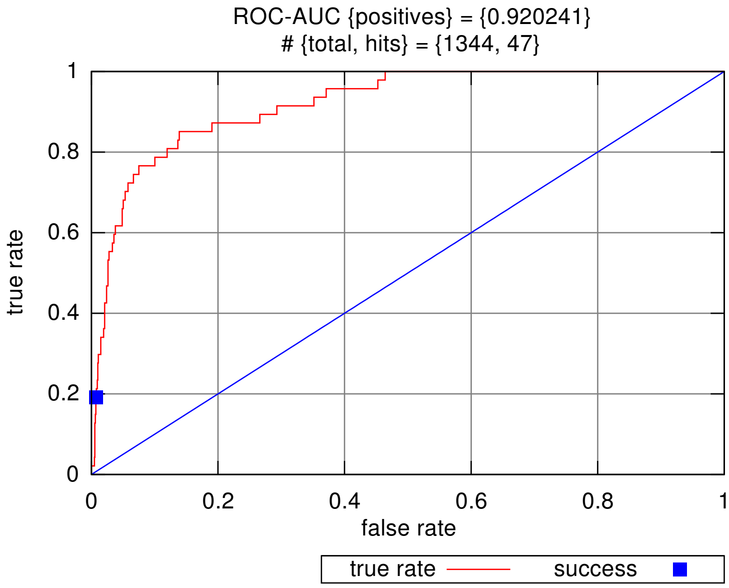

(a)

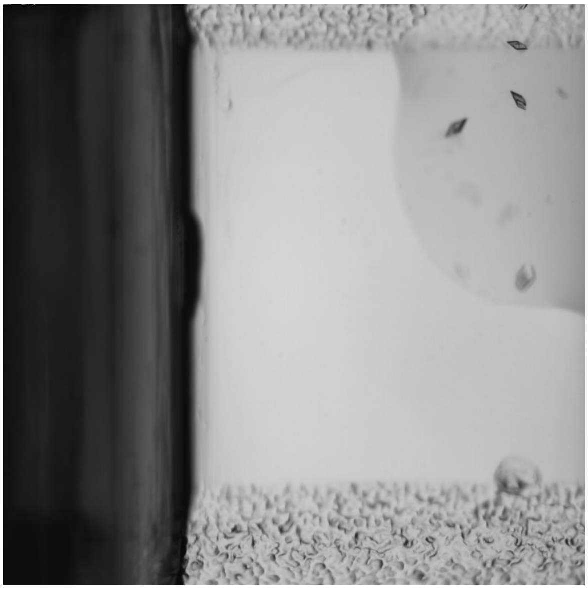

(b)

Figure 87: An ROC Curve for set 'SpeedET-FK5490E-dge-195-1-195' along with its highest ranked diffraction success.

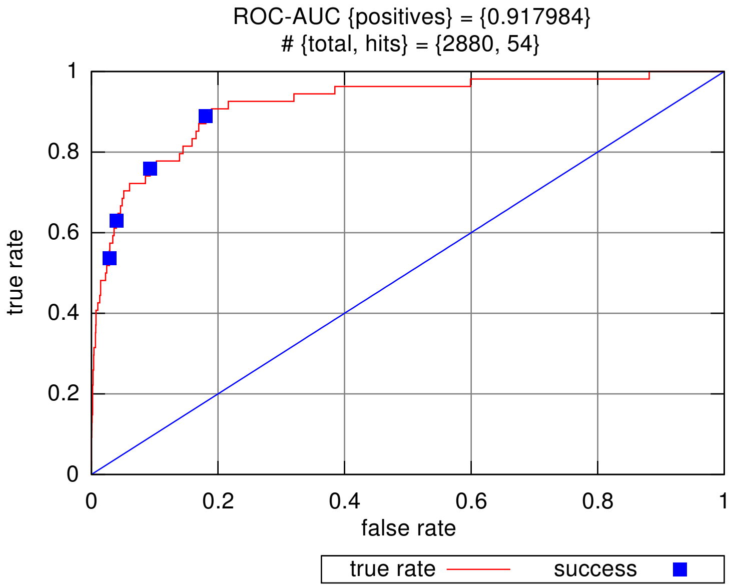

(a)

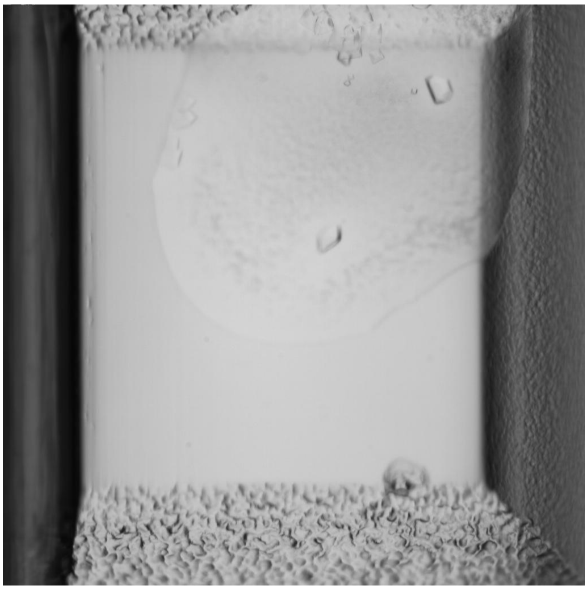

(b)

Figure 88: An ROC Curve for set 'SpeedET-FK4030A-eca-249-1-249' along with its highest ranked diffraction success.

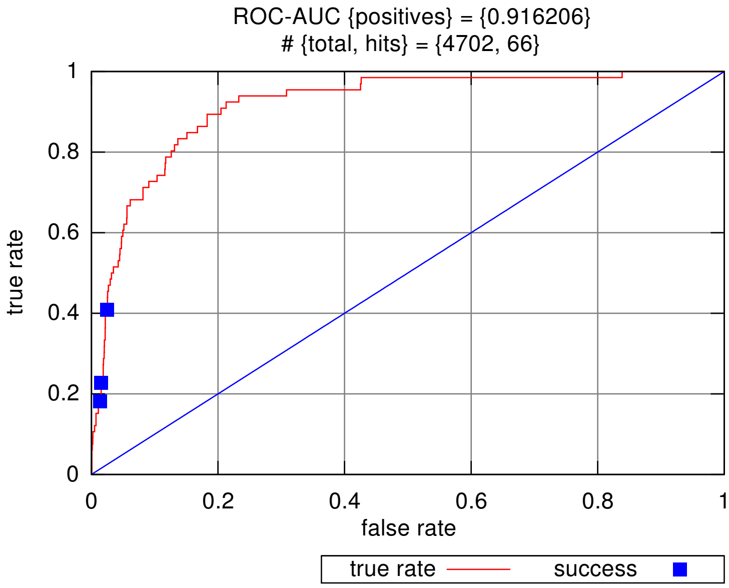

(a)

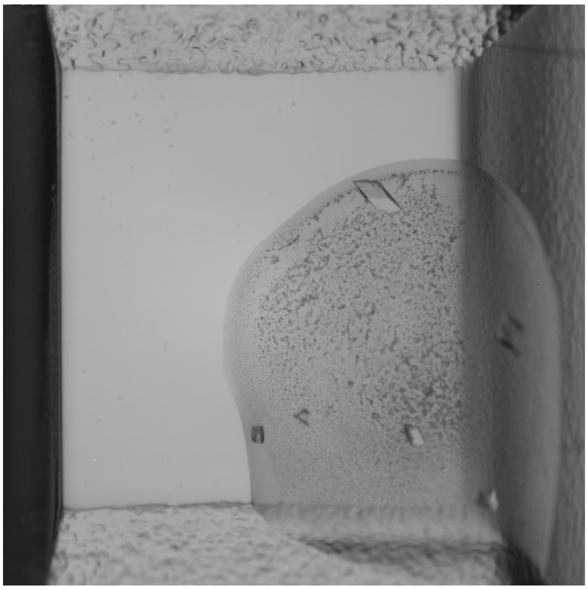

(b)

Figure 89: An ROC Curve for set 'SpeedET-PG9933A-sso-143-1-143' along with its highest ranked diffraction success.

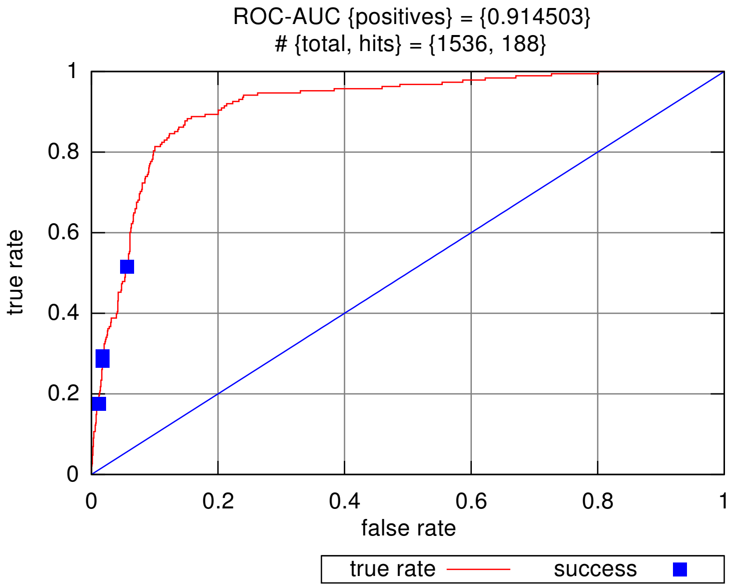

(a)

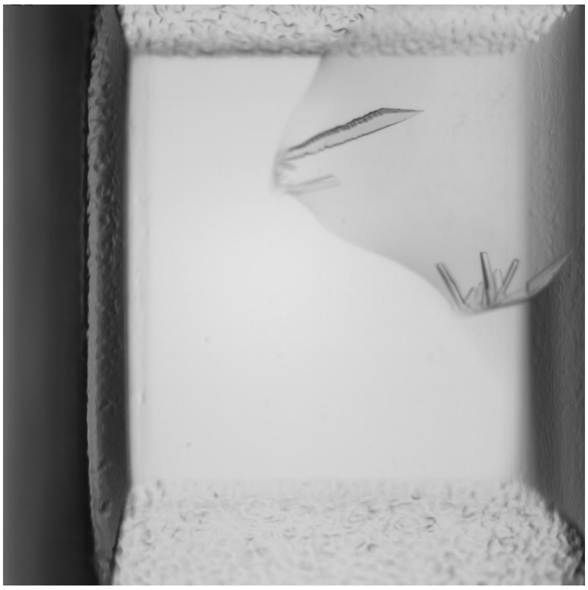

(b)

Figure 90: An ROC Curve for set 'SpeedET-PE00035B-ava-111-1-111' along with its highest ranked diffraction success.

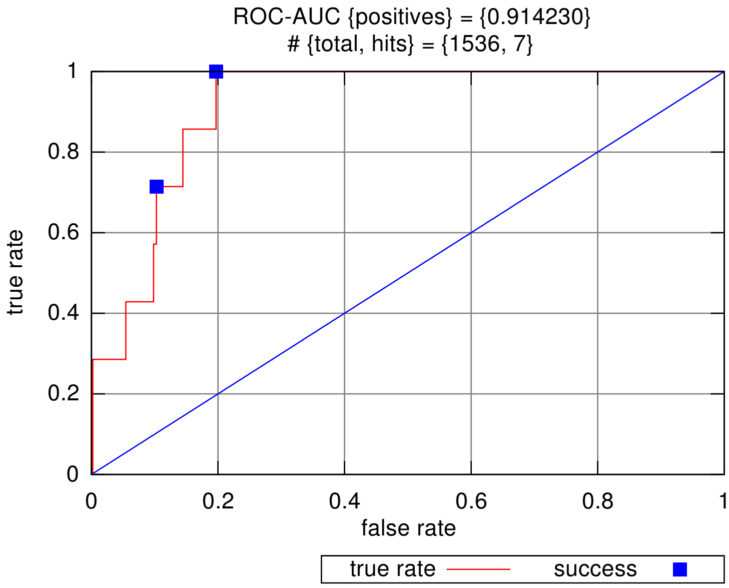

(a)

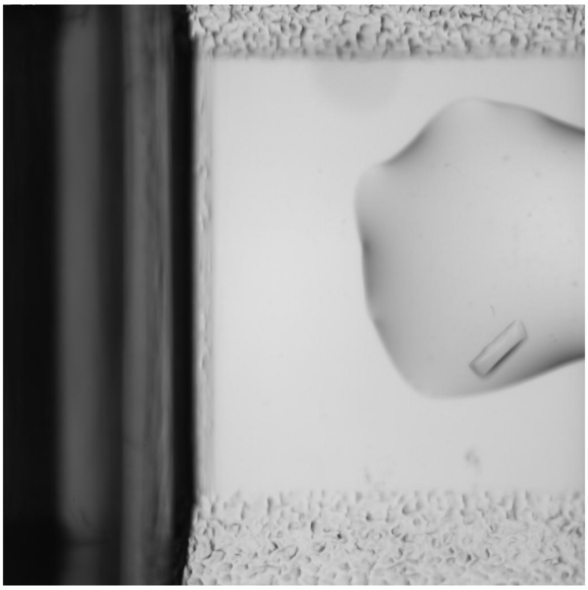

(b)

Figure 91: An ROC Curve for set ‘SpeedET-FG7255A-bce-153-1-153’ along with its highest ranked diffraction success.

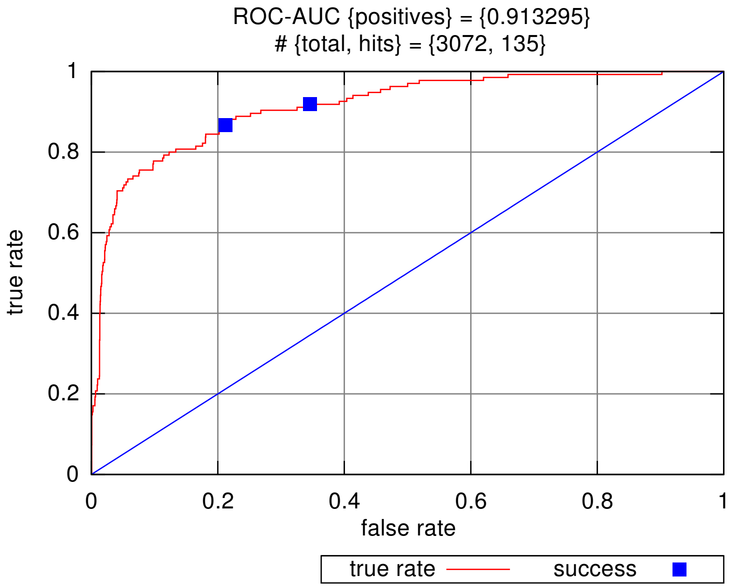

(a)

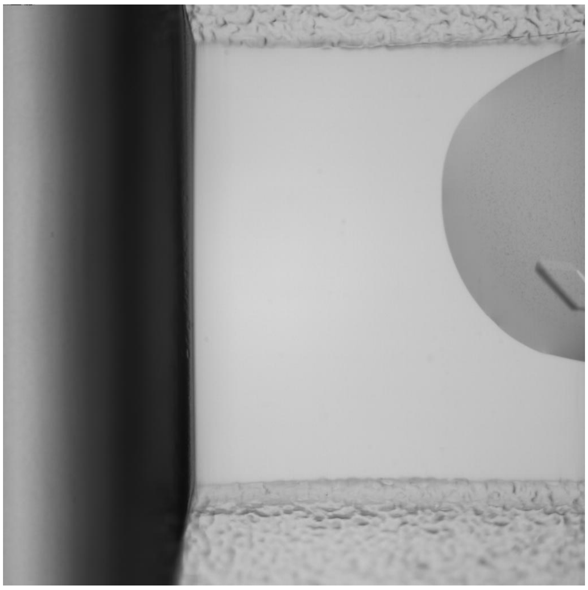

(b)

Figure 92: An ROC Curve for set ‘SpeedET-PE00025A-bsu-123-1-123’ along with its highest ranked diffraction success.

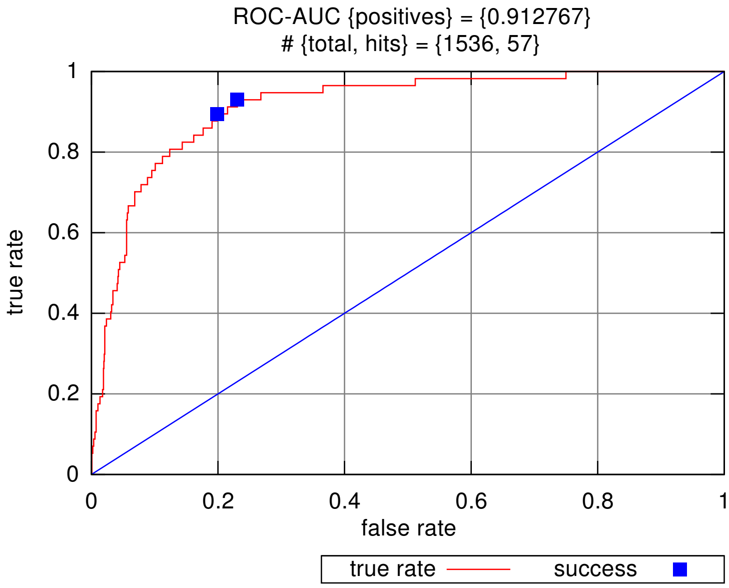

(a)

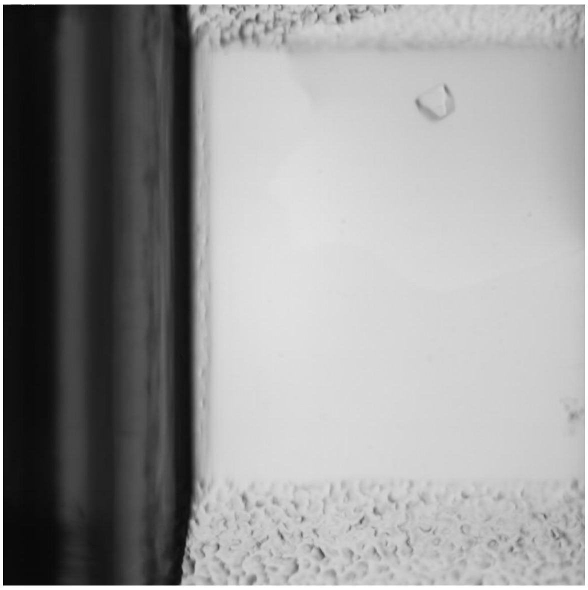

(b)

Figure 93: An ROC Curve for set ‘SpeedET-FJ8994A-bxe-133-1-133’ along with its highest ranked diffraction success.

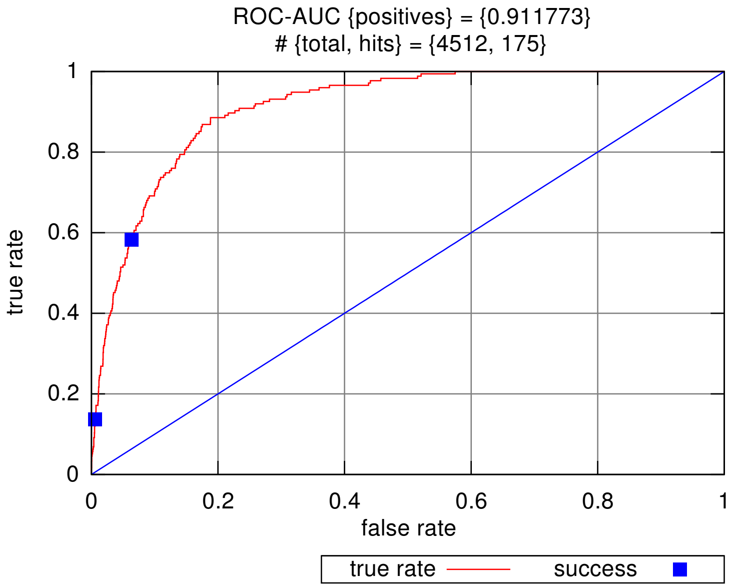

(a)

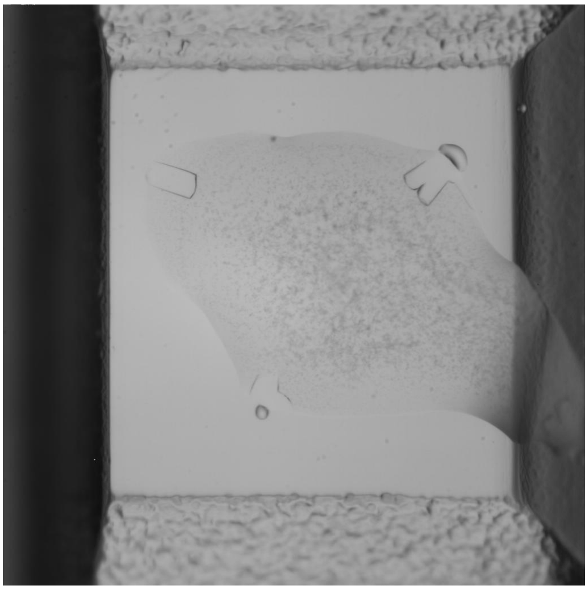

(b)

Figure 94: An ROC Curve for set 'SpeedET-PE00044A-ava-138-1-138' along with its highest ranked diffraction success.

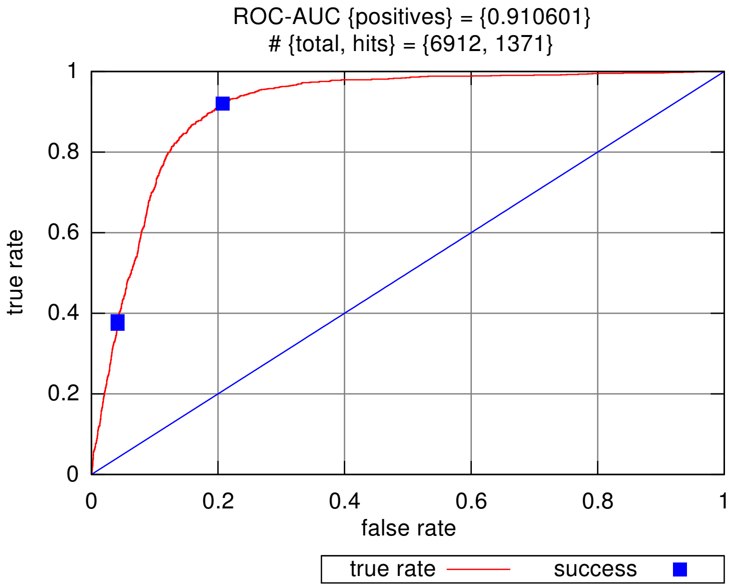

(a)

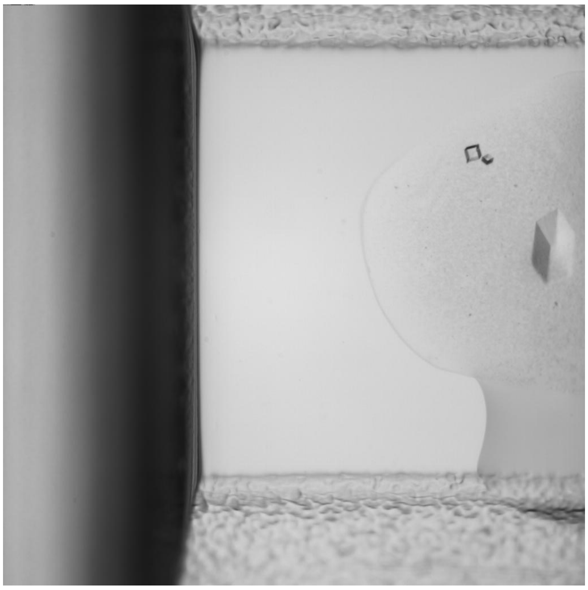

(b)

Figure 95: An ROC Curve for set 'SpeedET-PD06751F-lmo-454-1-454' along with its highest ranked diffraction success.

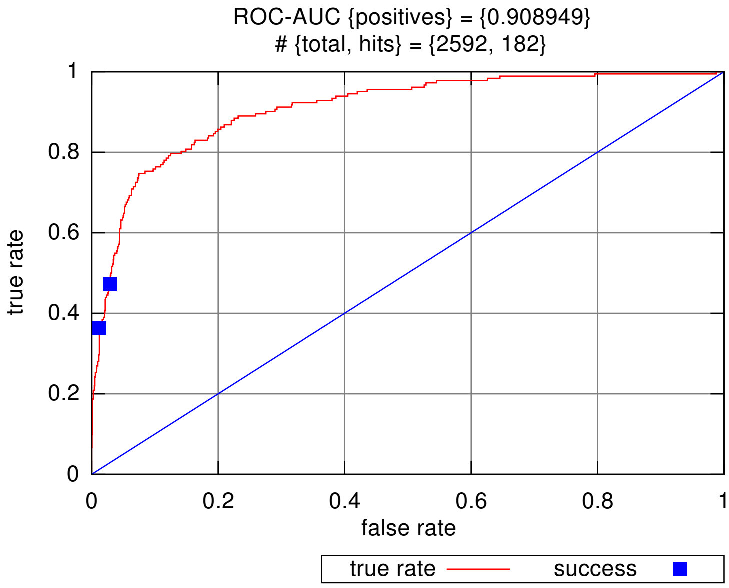

(a)

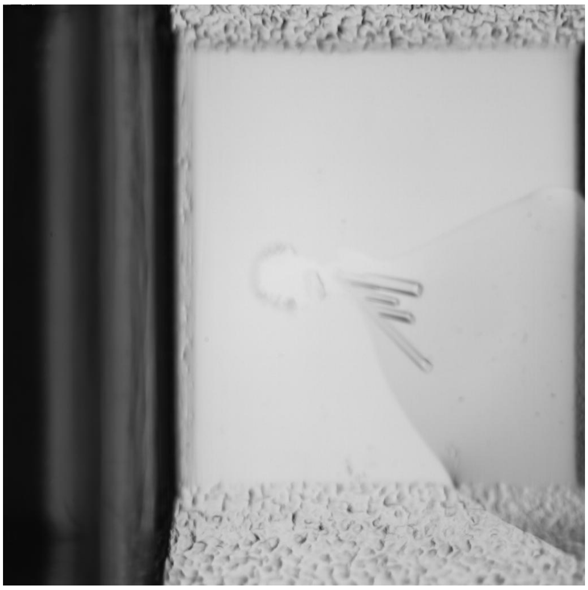

(b)

Figure 96: An ROC Curve for set 'SpeedET-FK9428A-mfl-153-1-153' along with its highest ranked diffraction success.

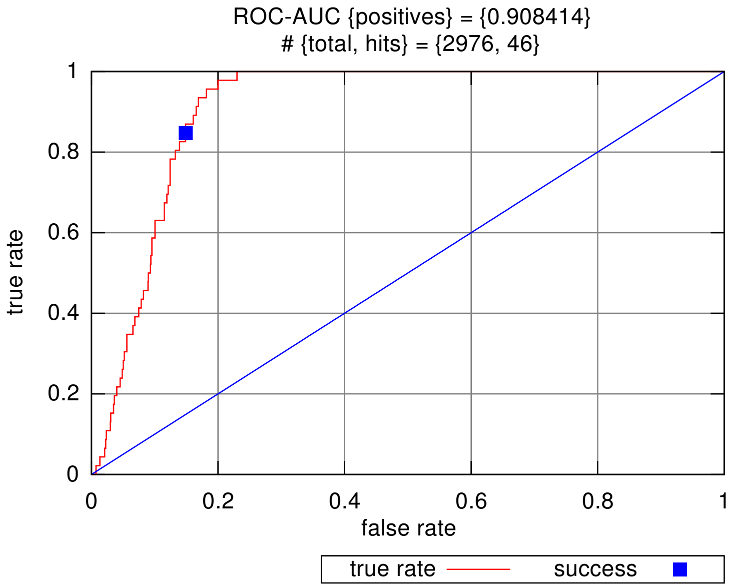

(a)

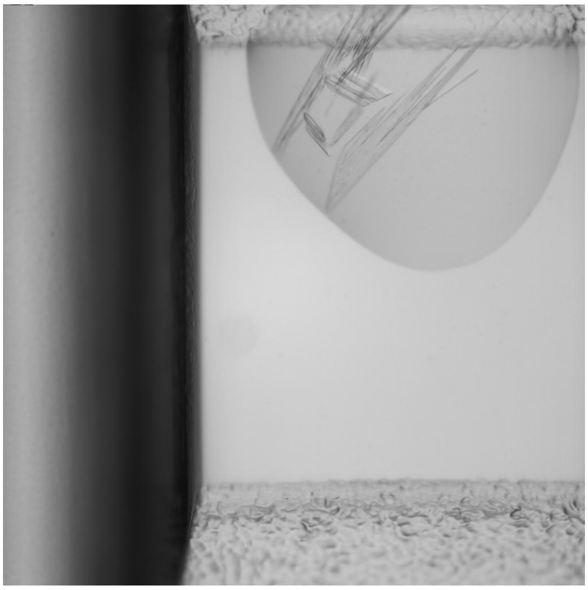

(b)

Figure 97: An ROC Curve for set 'SpeedET-NP_813429.1-bth-472-1-444' along with its highest ranked diffraction success.

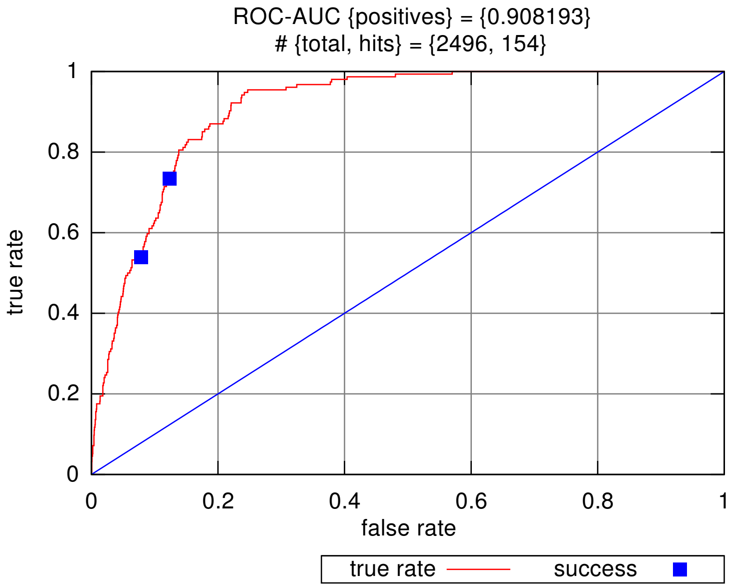

(a)

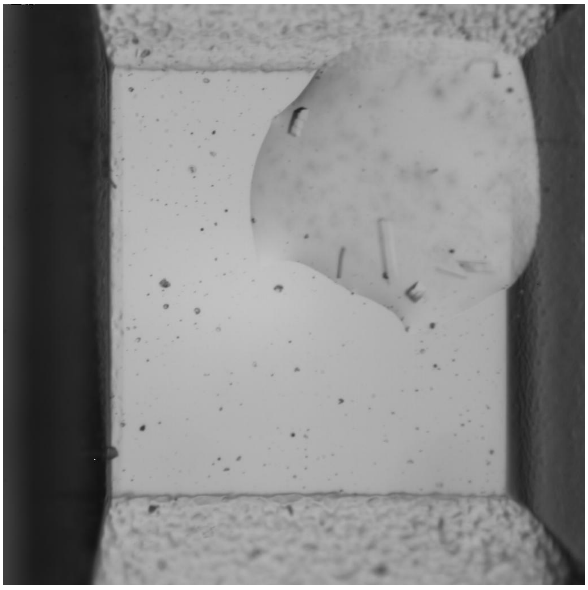

(b)

Figure 98: An ROC Curve for set 'SpeedET-FJ9081A-eca-335-1-335' along with its highest ranked diffraction success.

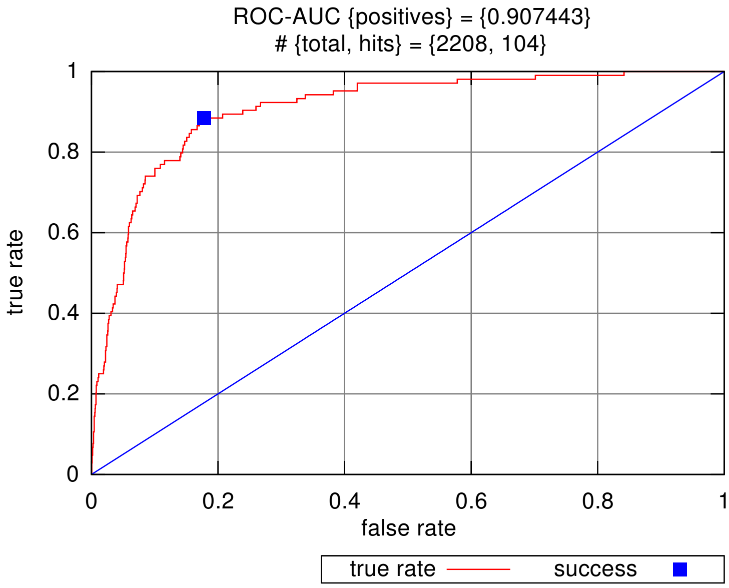

(a)

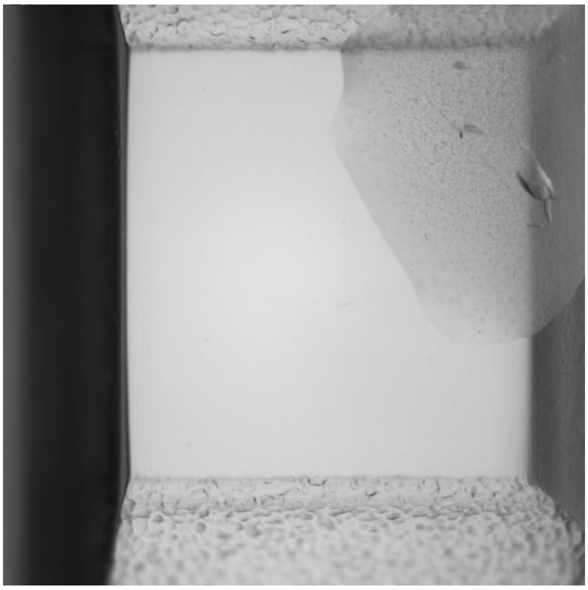

(b)

Figure 99: An ROC Curve for set ‘SpeedET-FH7599A-bha-427-1-427’ along with its highest ranked diffraction success.

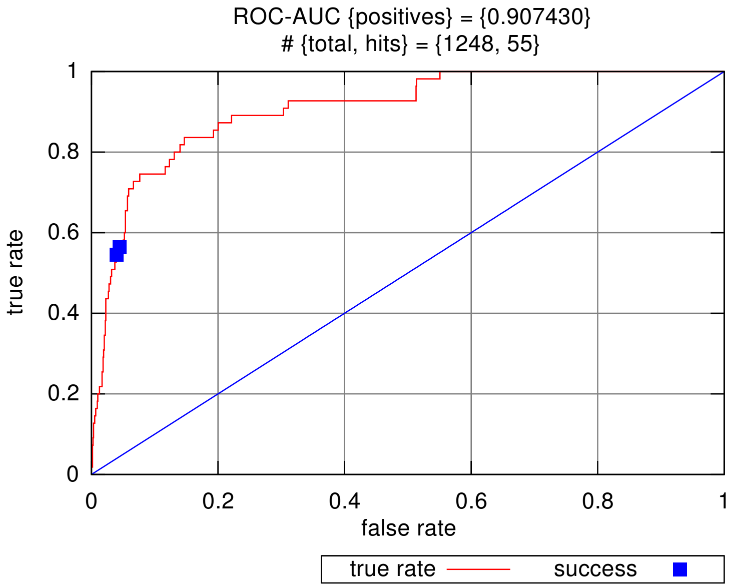

(a)

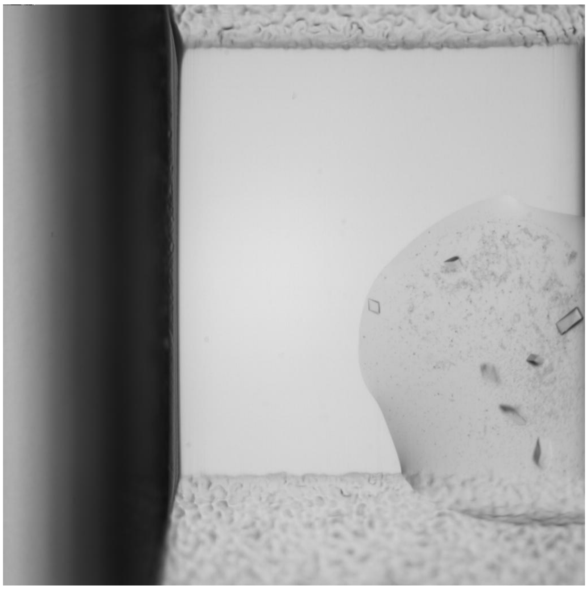

(b)

Figure 100: An ROC Curve for set 'SpeedET-FJ1648A-sag-195-1-195' along with its highest ranked diffraction success.

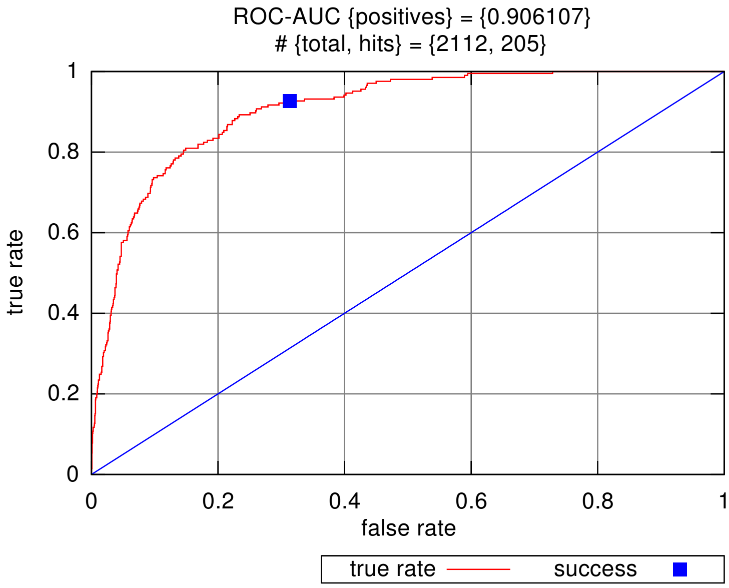

(a)

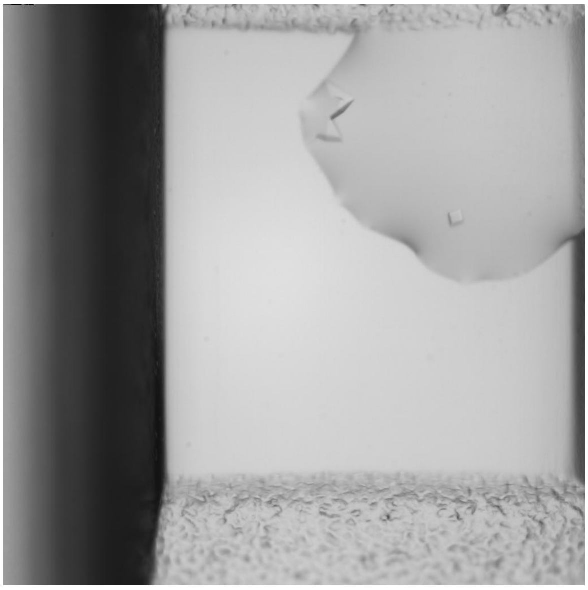

(b)

Figure 101: An ROC Curve for set 'SpeedET-FJ9248A-esp-210-1-210' along with its highest ranked diffraction success.

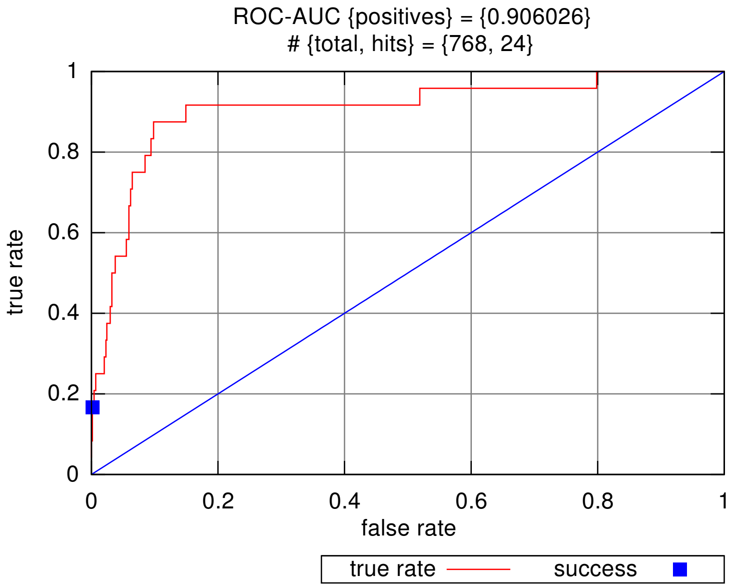

(a)

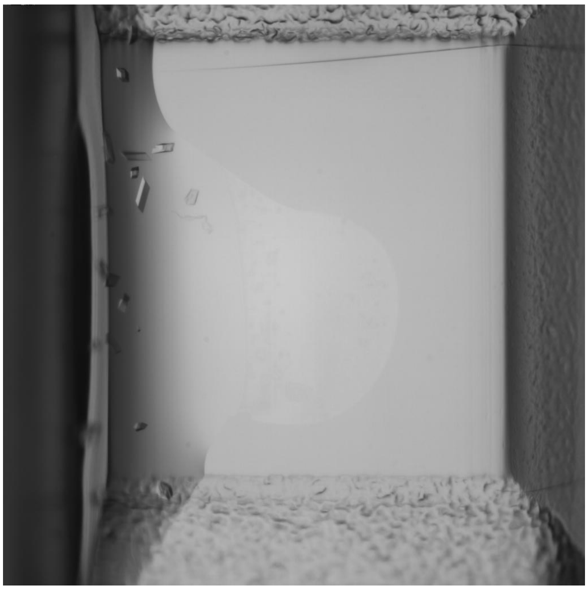

(b)

Figure 102: An ROC Curve for set 'SpeedET-PJ07336D-jsp-187-1-187' along with its highest ranked diffraction success.

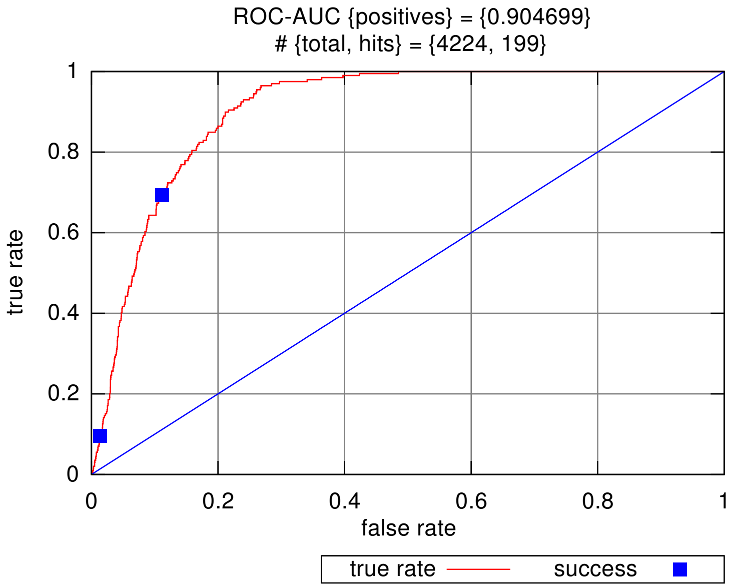

(a)

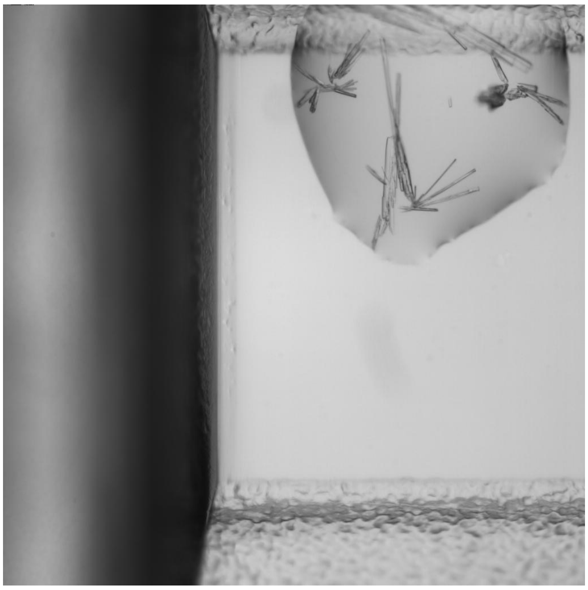

(b)

Figure 103: An ROC Curve for set 'SpeedET-FG7355A-mlo-217-1-217' along with its highest ranked diffraction success.

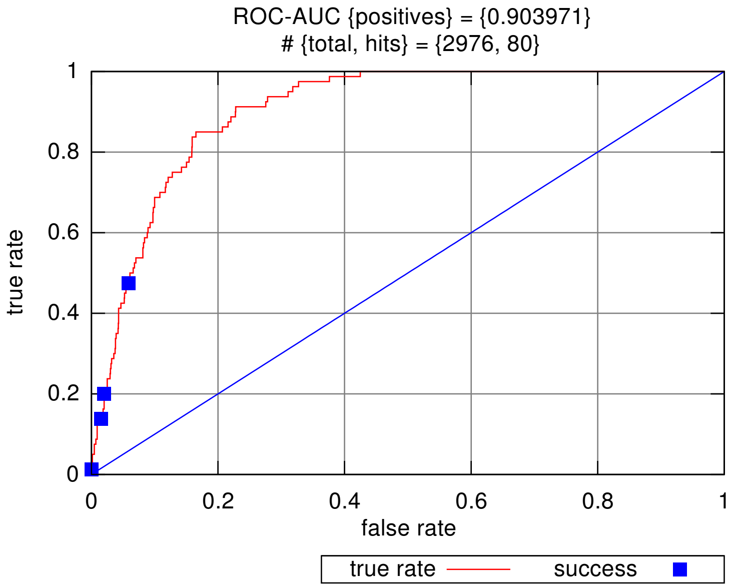

(a)

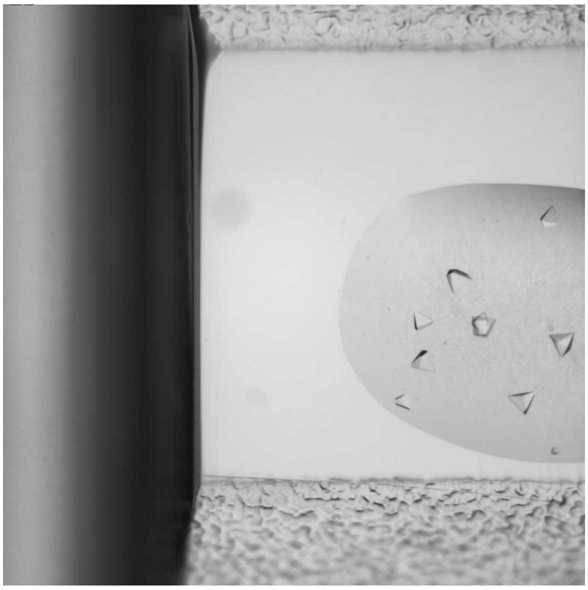

(b)

Figure 104: An ROC Curve for set 'SpeedET-PE00293D-mlo-285-1-285' along with its highest ranked diffraction success.

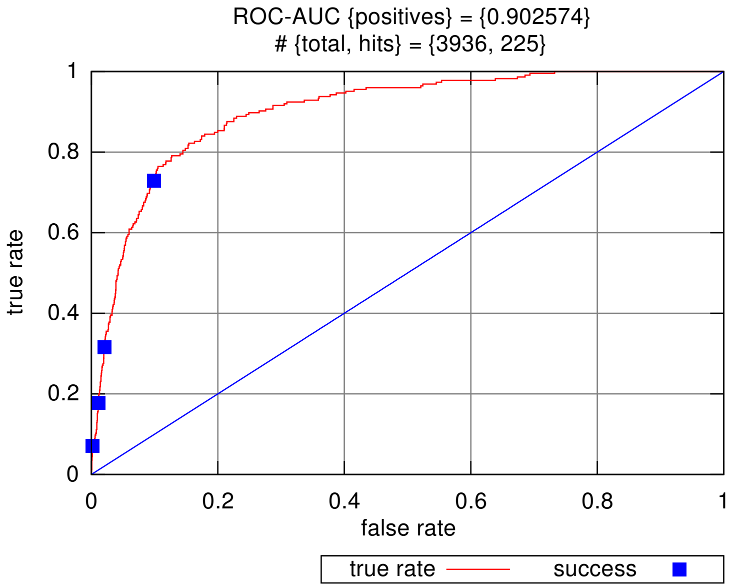

(a)

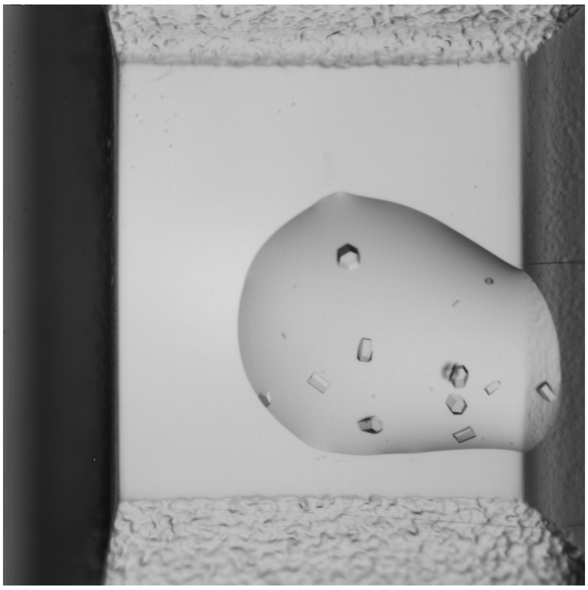

(b)

Figure 105: An ROC Curve for set 'SpeedET-PC05163A-esp-148-1-148' along with its highest ranked diffraction success.

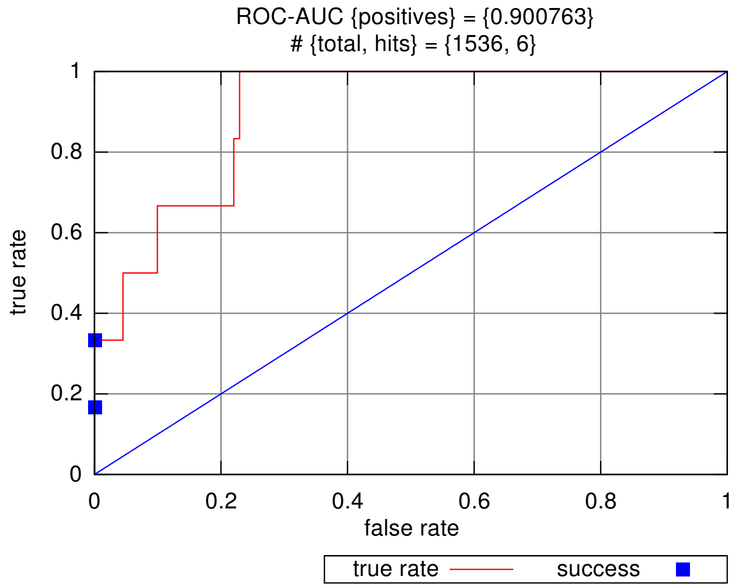

(a)

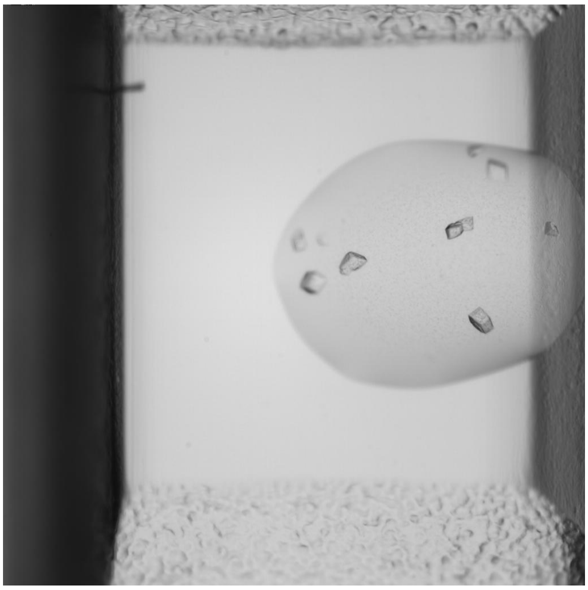

(b)

Figure 106: An ROC Curve for set ‘SpeedET-FH7681A-lbu-121-1-121’ along with its highest ranked diffraction success.

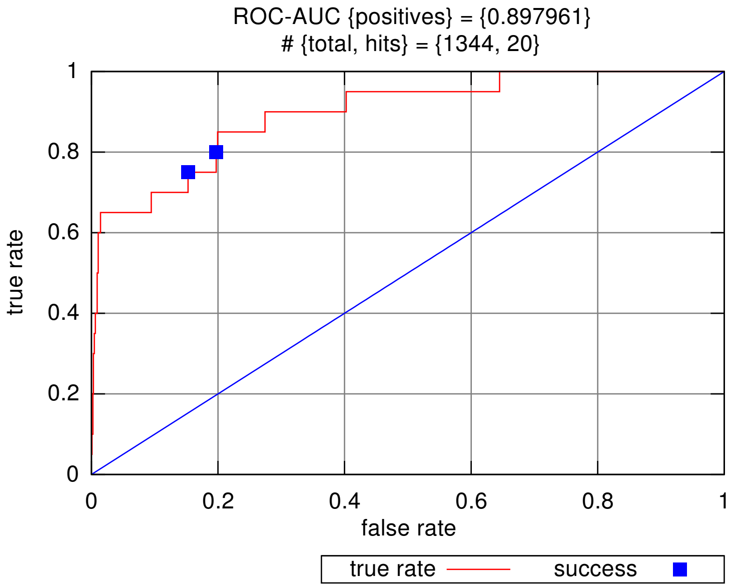

(a)

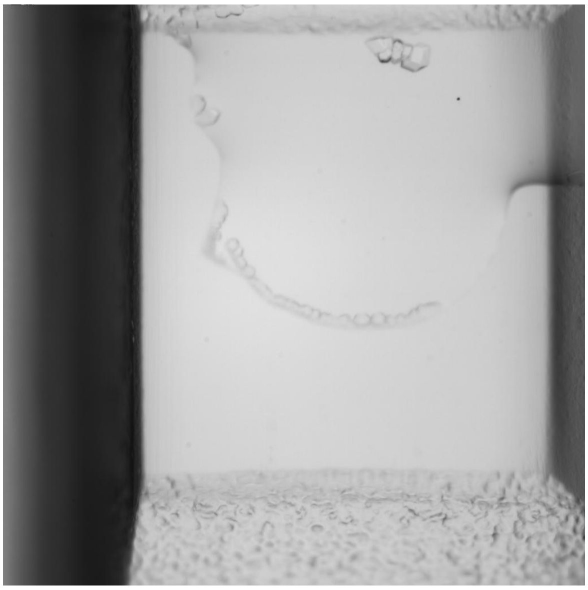

(b)

Figure 107: An ROC Curve for set ‘SpeedET-FJ8827A-mpe-144-1-144’ along with its highest ranked diffraction success.

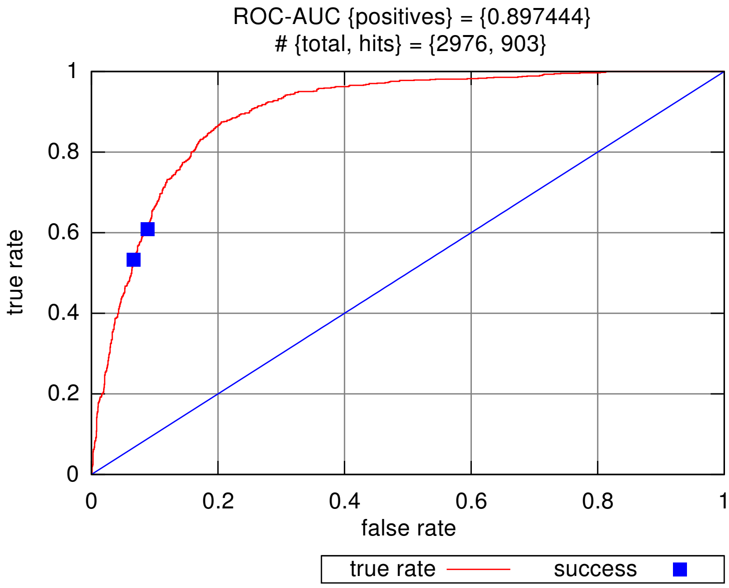

(a)

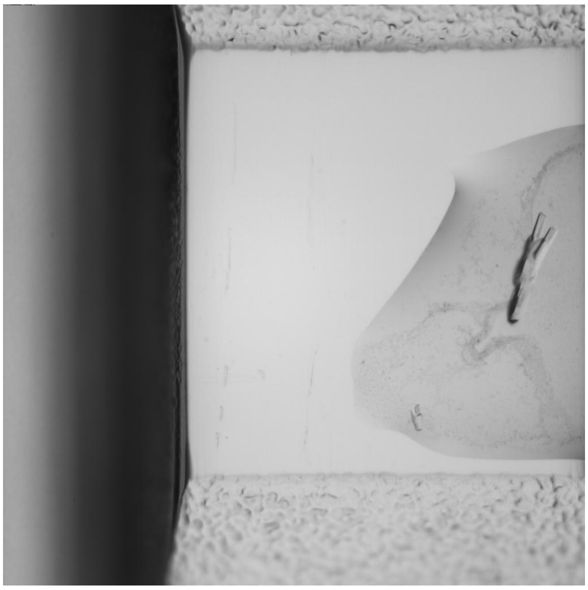

(b)

Figure 108: An ROC Curve for set 'SpeedET-FK9452E-son-153-1-153' along with its highest ranked diffraction success.

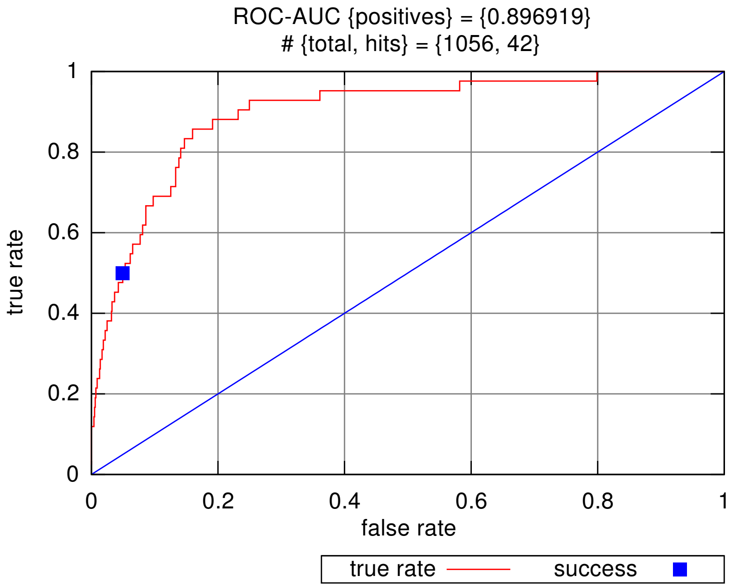

(a)

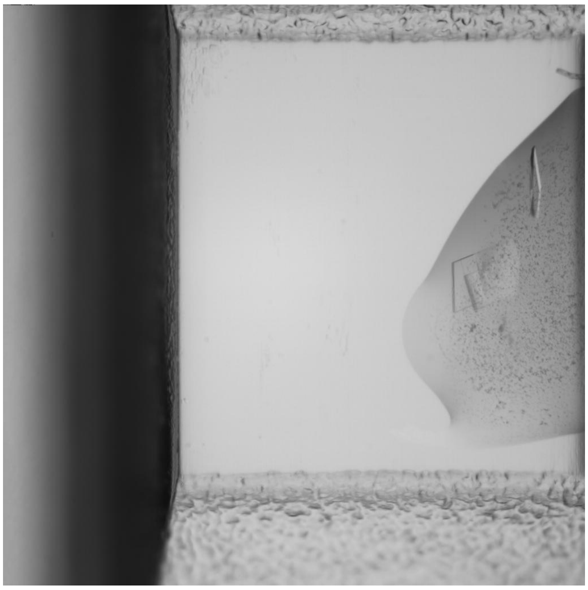

(b)

Figure 109: An ROC Curve for set ‘SpeedET-FJ8835A-tac-139-1-139’ along with its highest ranked diffraction success.

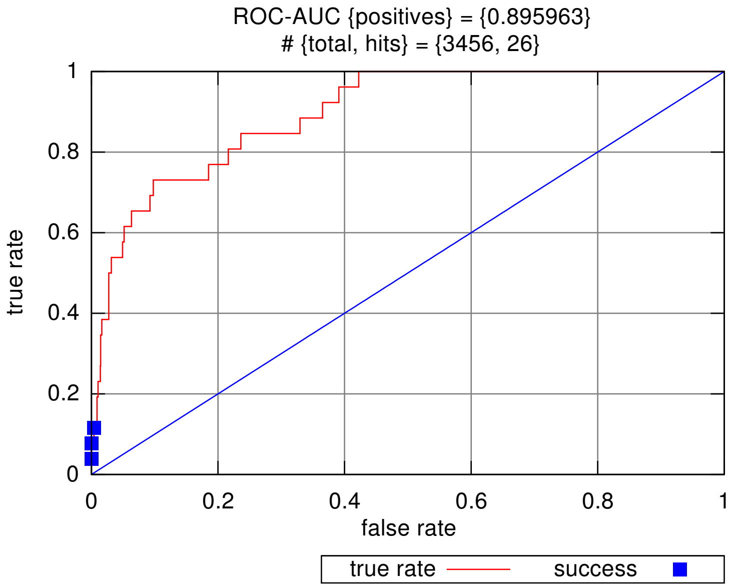

(a)

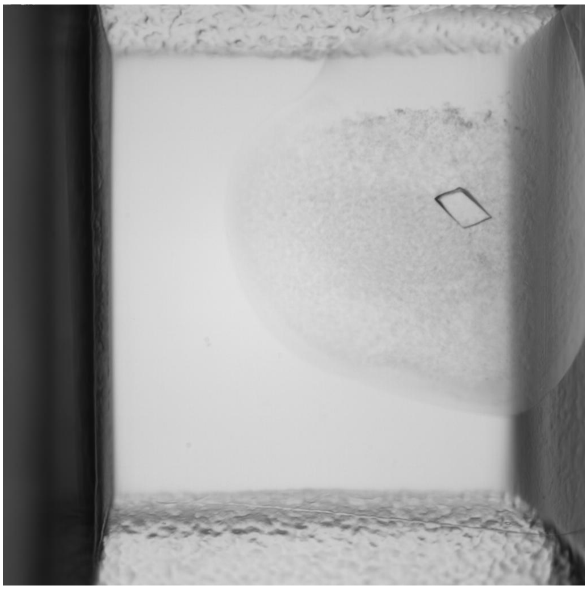

(b)

Figure 110: An ROC Curve for set ‘SpeedET-PC07755A-ava-348-1-348-K56Y-D58Y’ along with its highest ranked diffraction success.

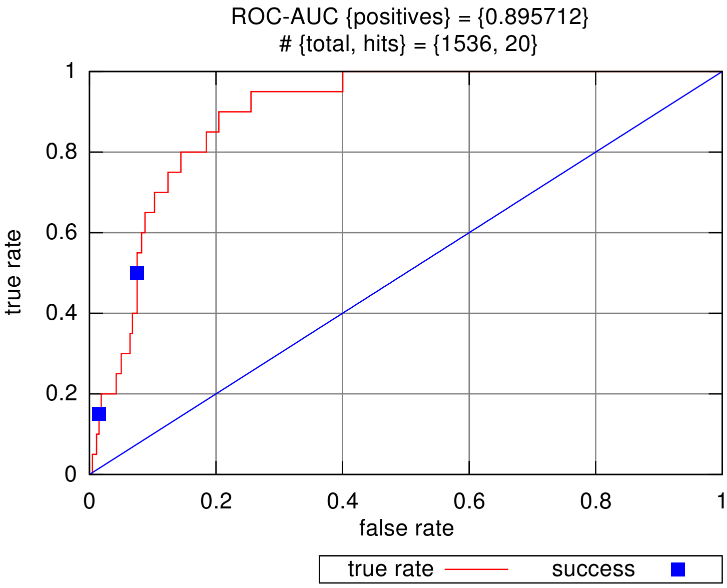

(a)

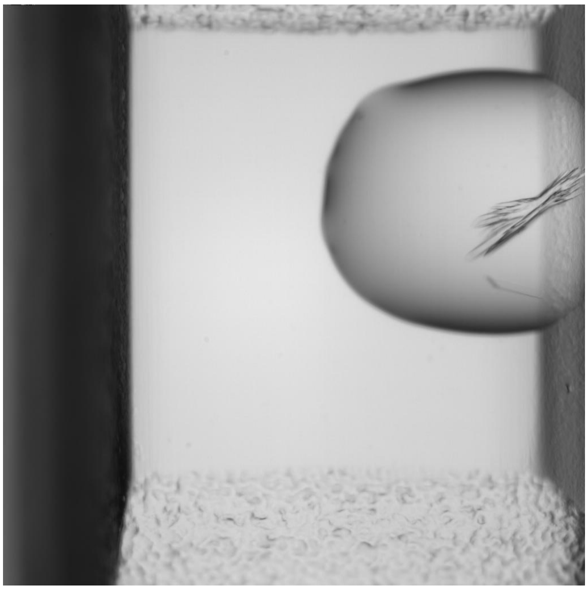

(b)

Figure 111: An ROC Curve for set 'SpeedET-PE00092A-bxe-116-1-116' along with its highest ranked diffraction success.

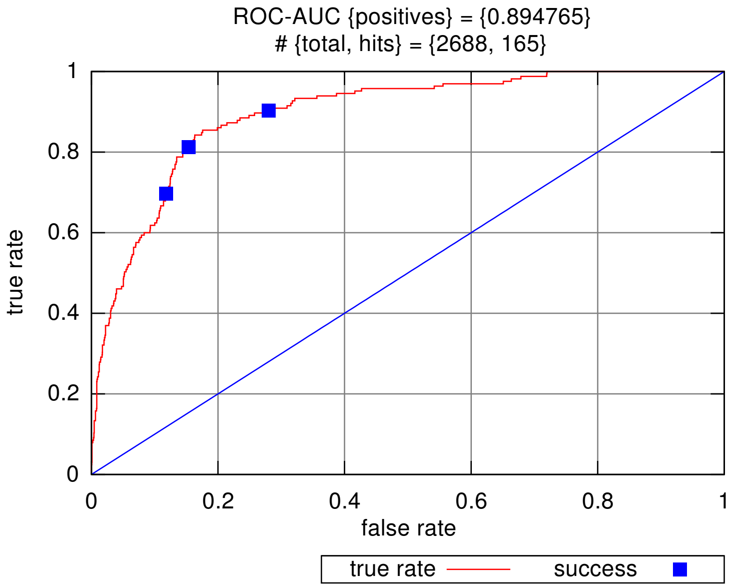

(a)

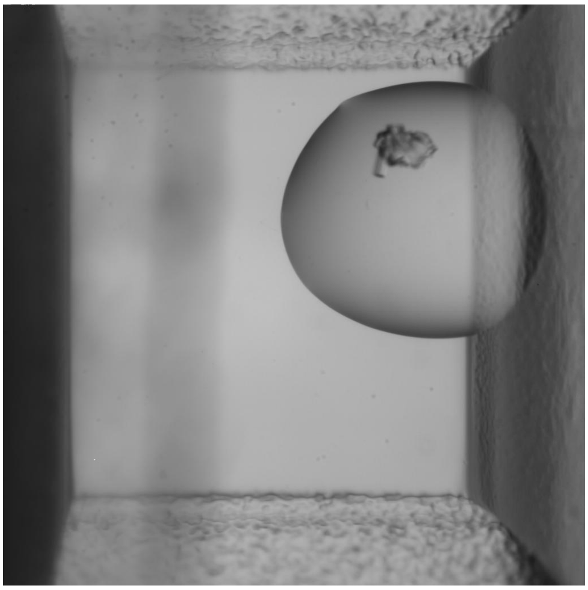

(b)

Figure 112: An ROC Curve for set 'SpeedET-PE00238G-sdn-173-1-173' along with its highest ranked diffraction success.

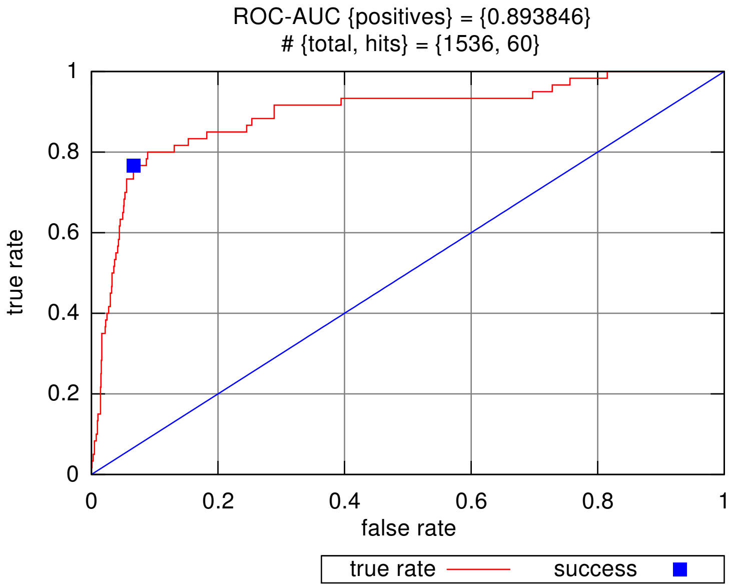

(a)

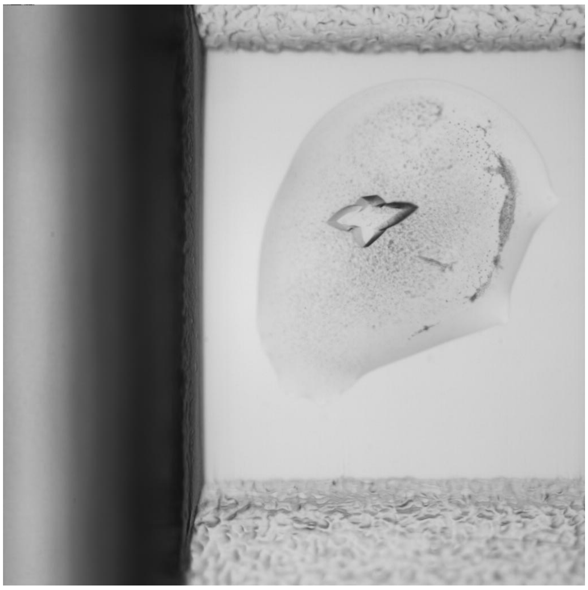

(b)

Figure 113: An ROC Curve for set ‘SpeedET-GN7757A-ocn-109-1-109’ along with its highest ranked diffraction success.

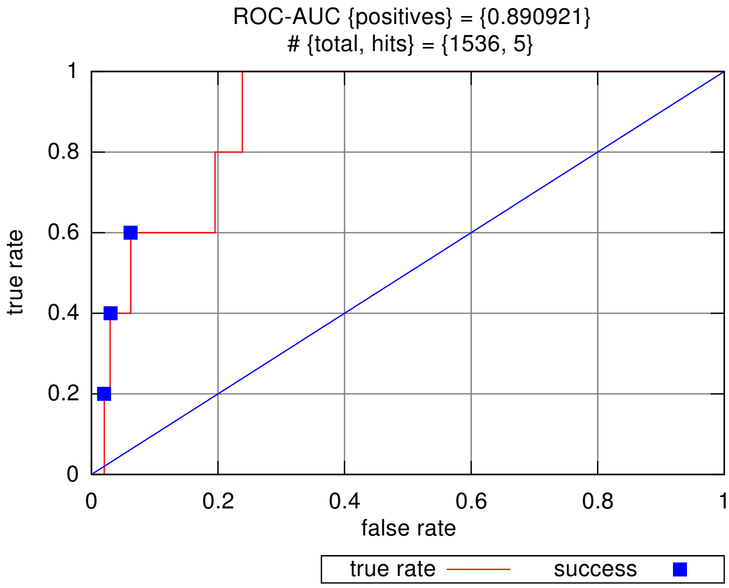

(a)

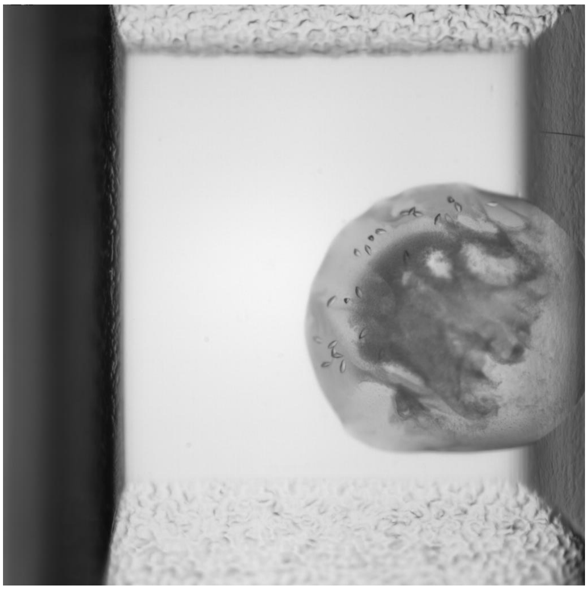

(b)

Figure 114: An ROC Curve for set 'SpeedET-FG7279A-cac-145-1-145' along with its highest ranked diffraction success.

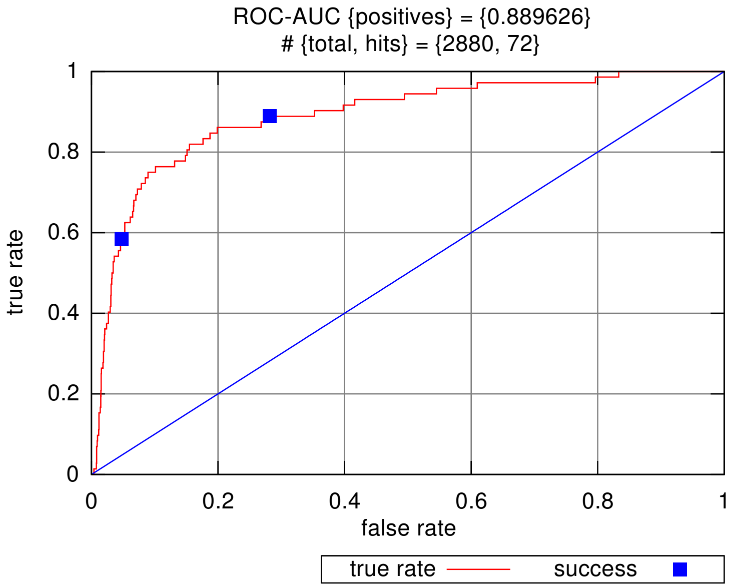

(a)

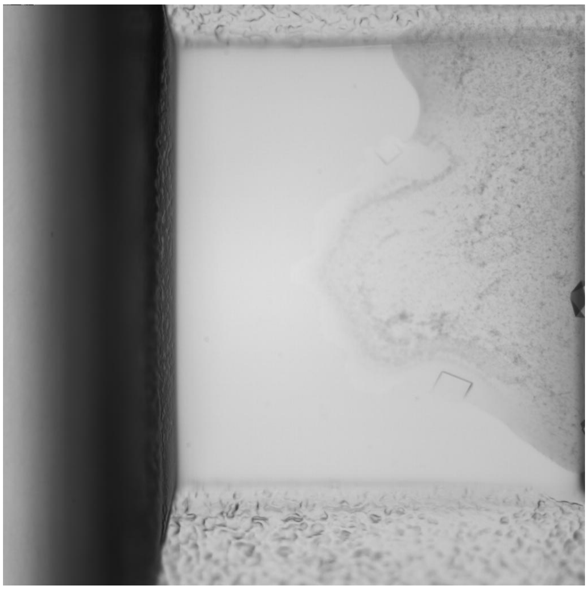

(b)

Figure 115: An ROC Curve for set 'SpeedET-PE00035A-ava-139-1-139' along with its highest ranked diffraction success.

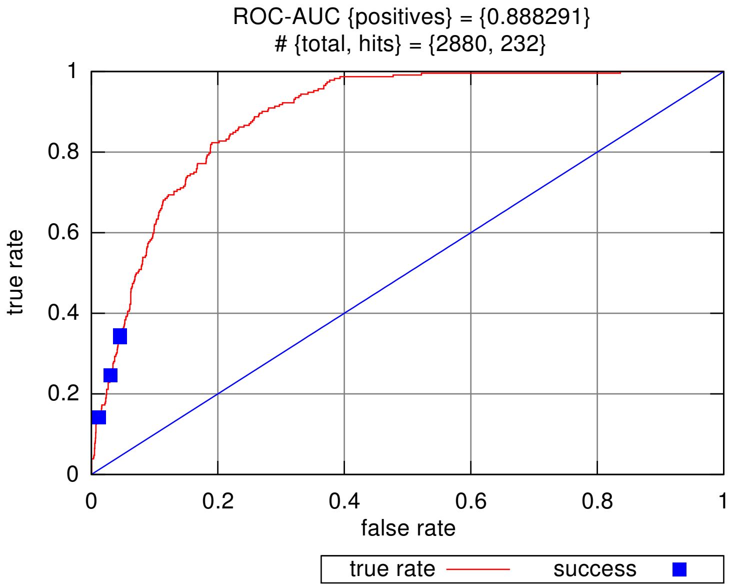

(a)

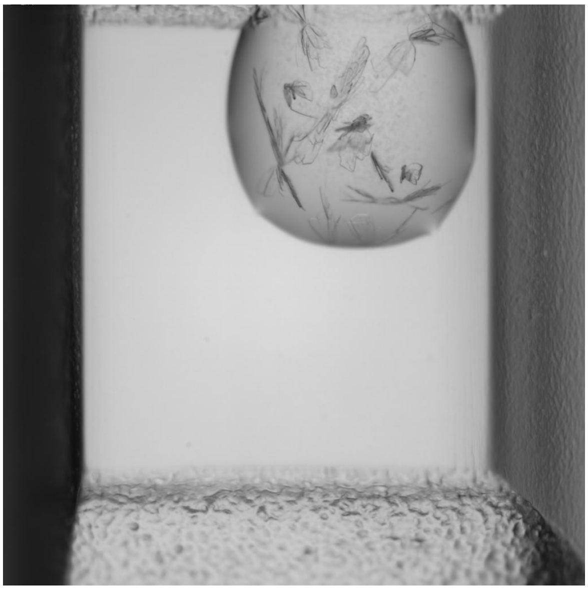

(b)

Figure 116: An ROC Curve for set 'SpeedET-FJ9092A-esp-330-1-330' along with its highest ranked diffraction success.

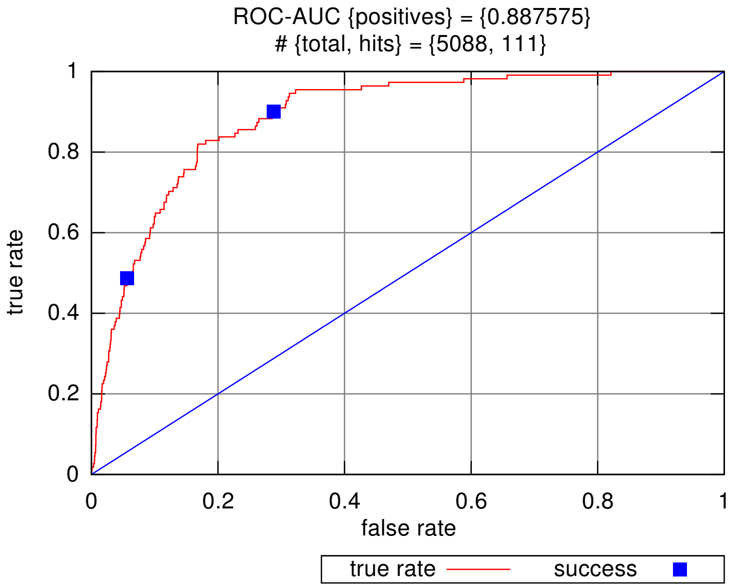

(a)

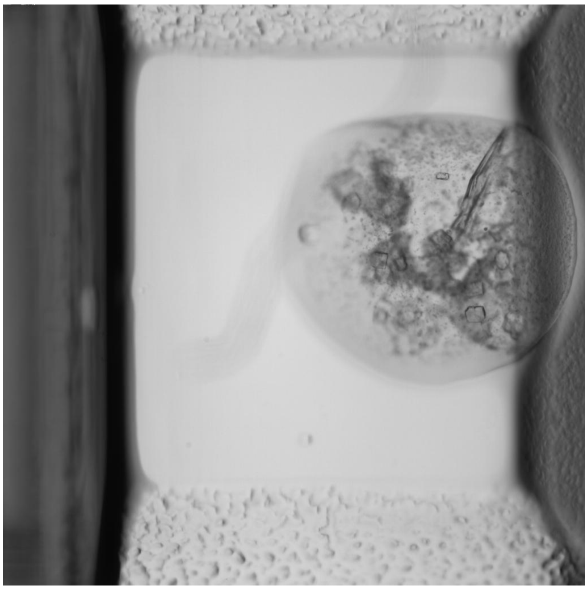

(b)

Figure 117: An ROC Curve for set 'SpeedET-PD01933E-sep-331-1-331' along with its highest ranked diffraction success.

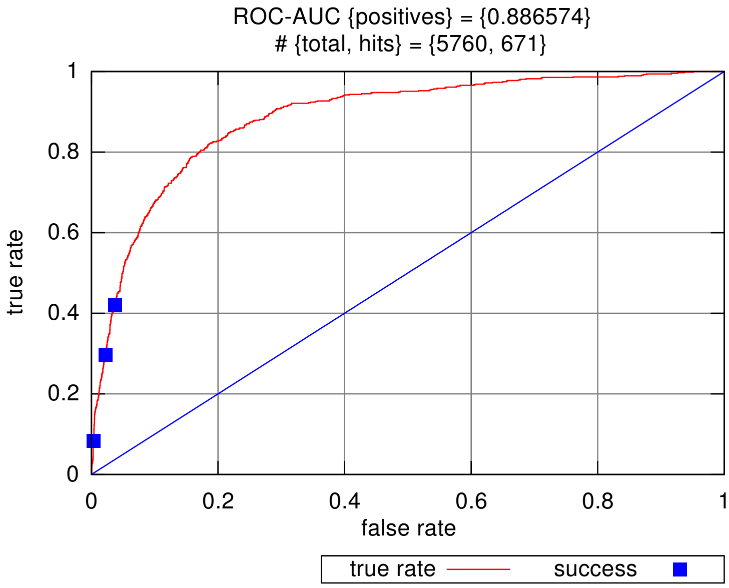

(a)

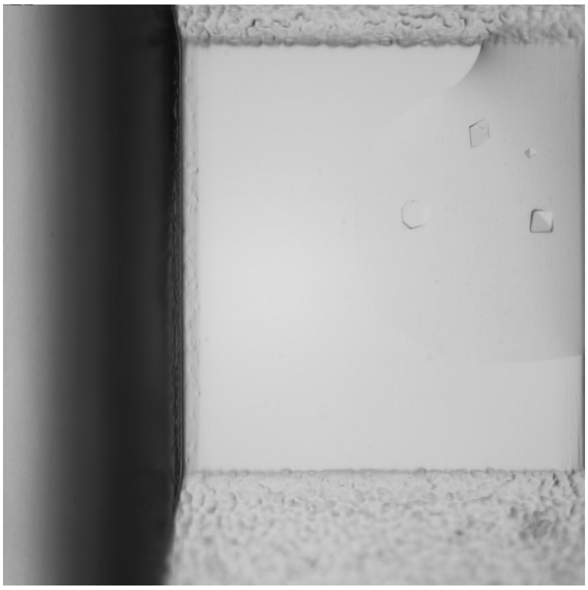

(b)

Figure 118: An ROC Curve for set ‘SpeedET-PE00037E-syn-121-1-121’ along with its highest ranked diffraction success.

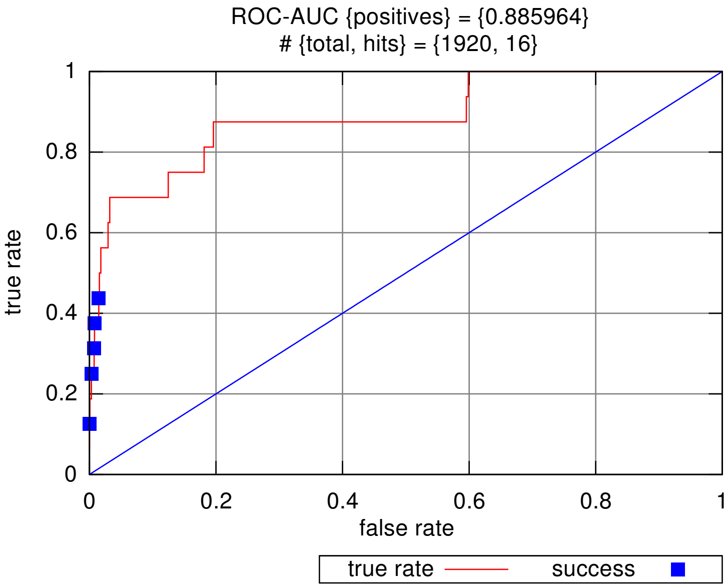

(a)

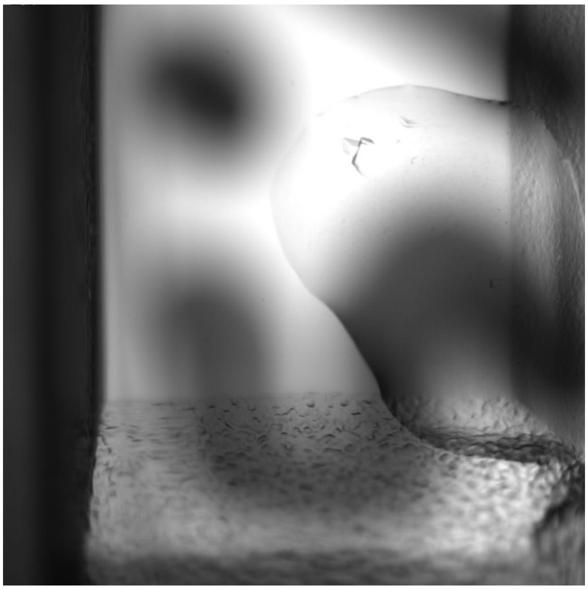

(b)

Figure 119: An ROC Curve for set 'SpeedET-HP9625C-hso-486-1-486' along with its highest ranked diffraction success.

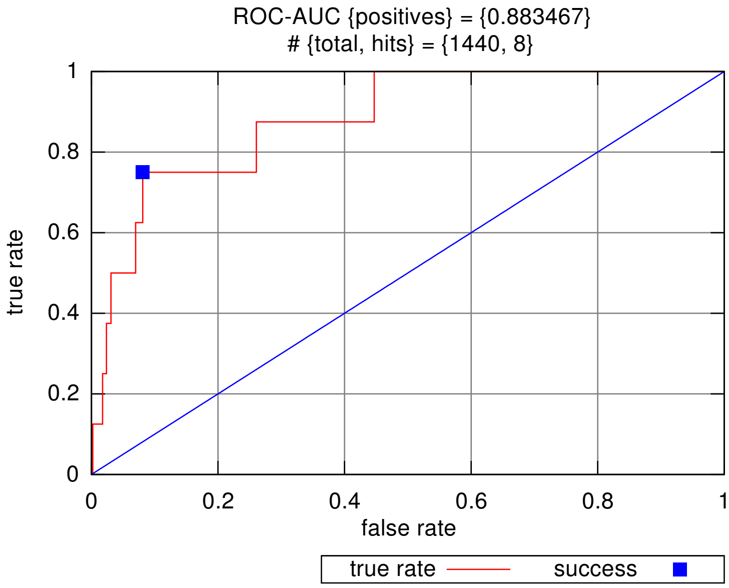

(a)

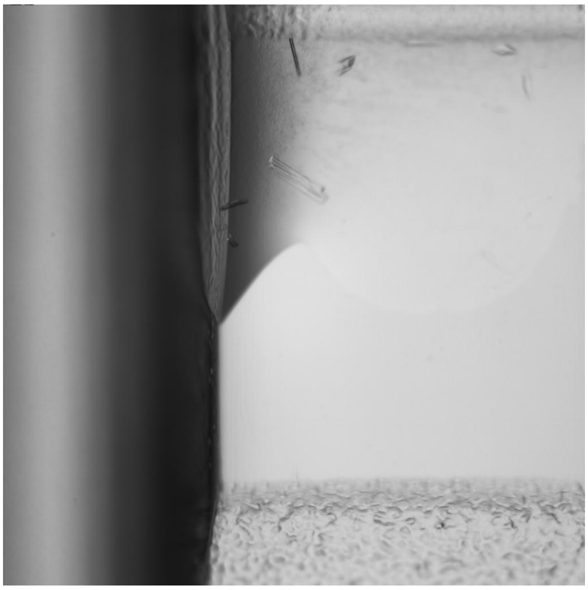

(b)

Figure 120: An ROC Curve for set 'SpeedET-GN7738A-ocn-100-1-100' along with its highest ranked diffraction success.

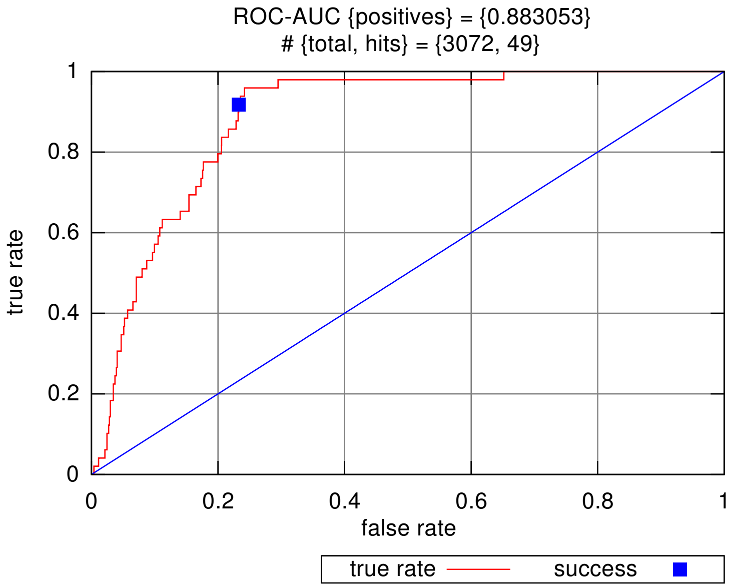

(a)

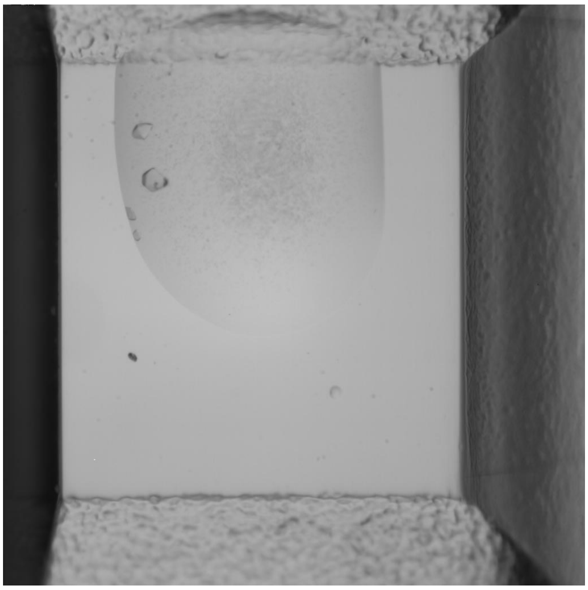

(b)

Figure 121: An ROC Curve for set 'SpeedET-CM7979A-npu-302-1-302' along with its highest ranked diffraction success.

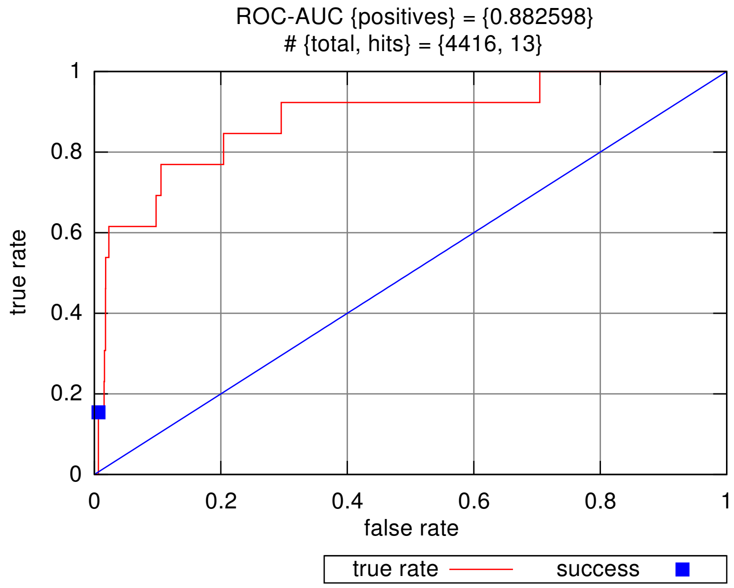

(a)

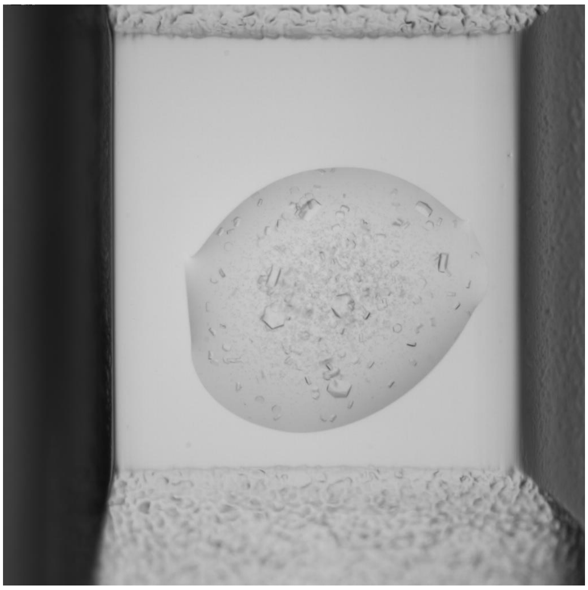

(b)

Figure 122: An ROC Curve for set 'SpeedETS-2635576-bsu-357-13-357' along with its highest ranked diffraction success.

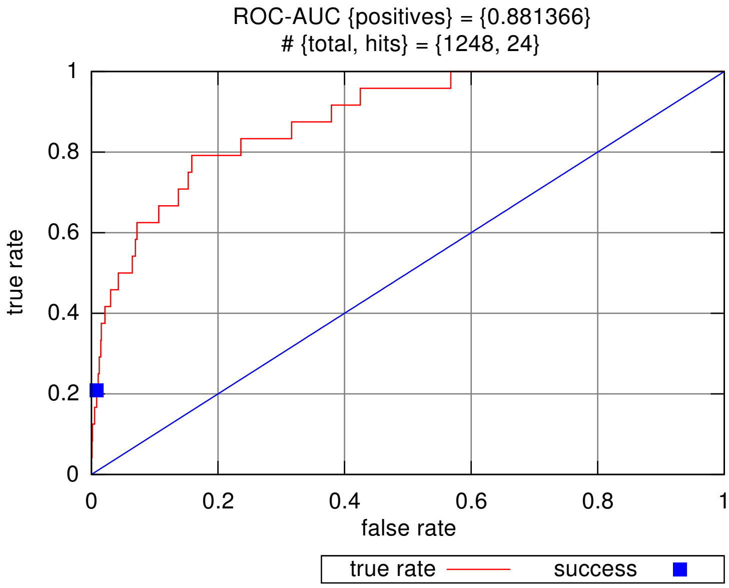

(a)

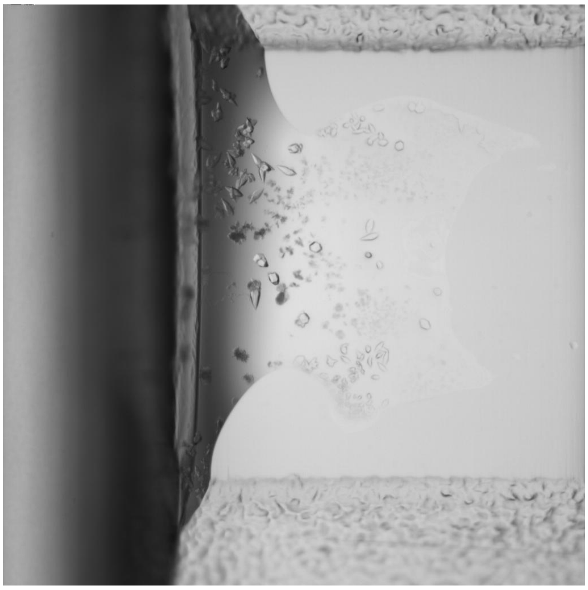

(b)

Figure 123: An ROC Curve for set 'SpeedET-FJ9230A-cgl-198-1-198' along with its highest ranked diffraction success.

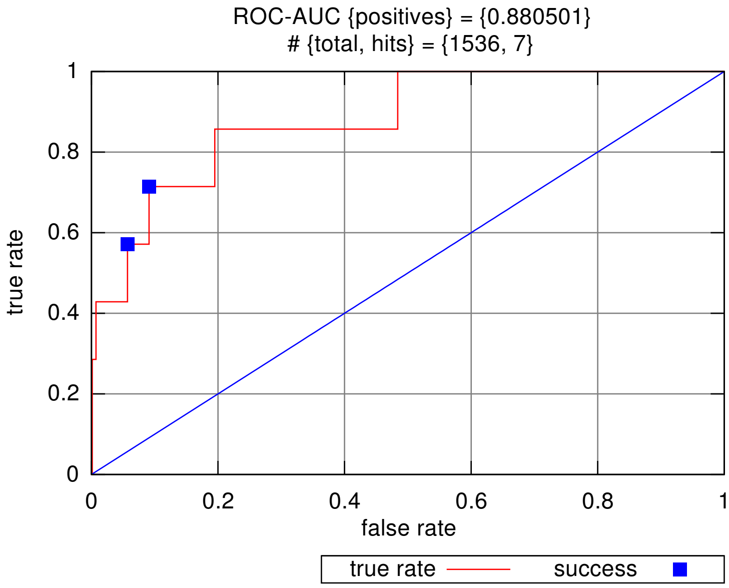

(a)

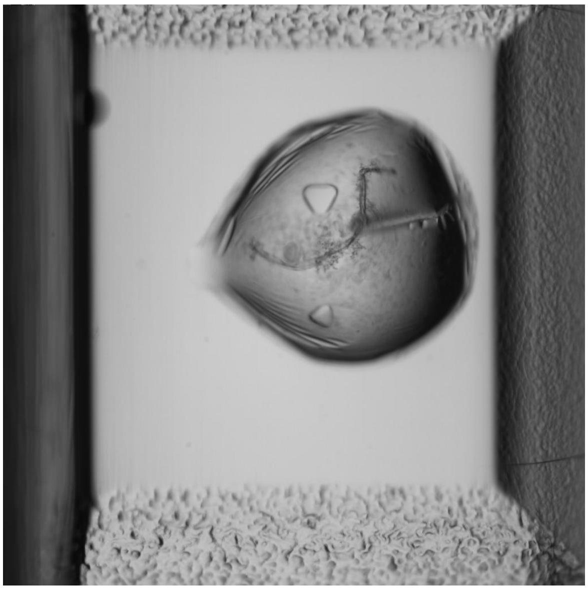

(b)

Figure 124: An ROC Curve for set 'SpeedET-FK8795C-xca-141-1-141' along with its highest ranked diffraction success.

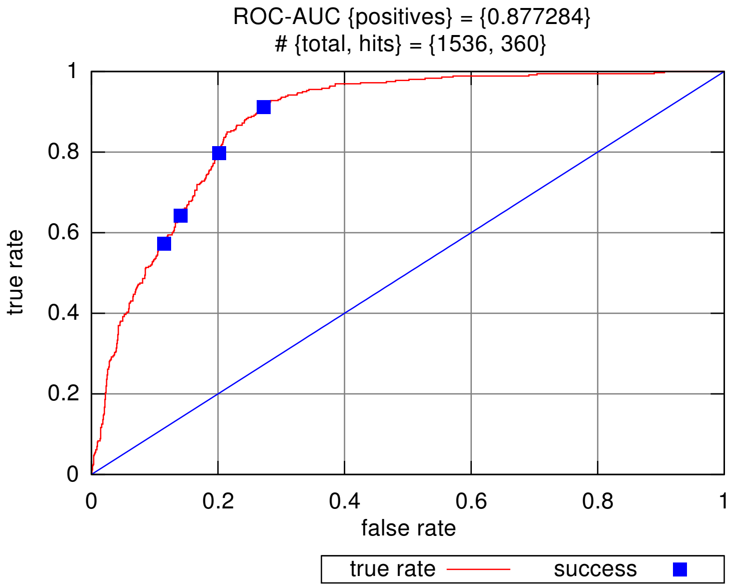

(a)

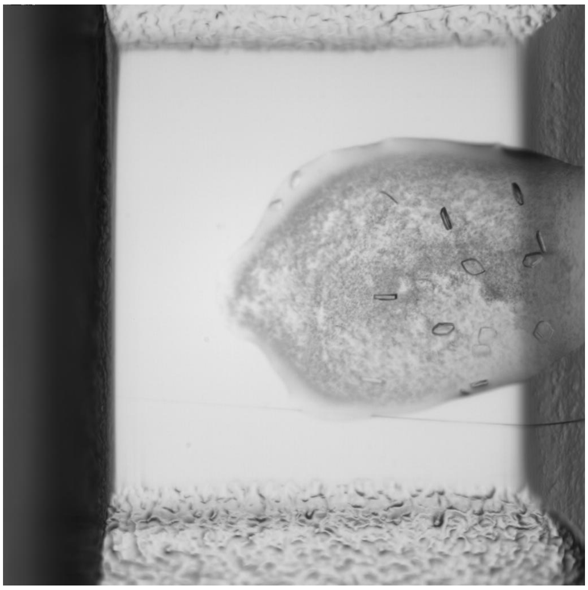

(b)

Figure 125: An ROC Curve for set 'SpeedET-FH7700A-mlo-148-1-148' along with its highest ranked diffraction success.

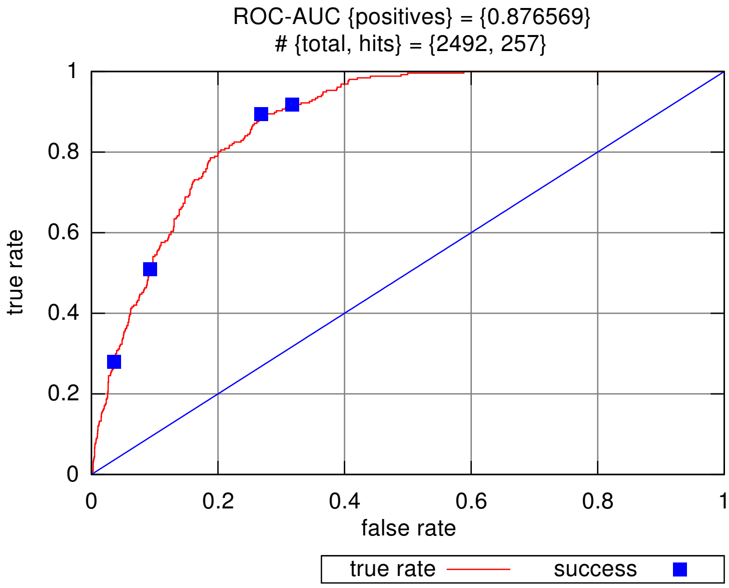

(a)

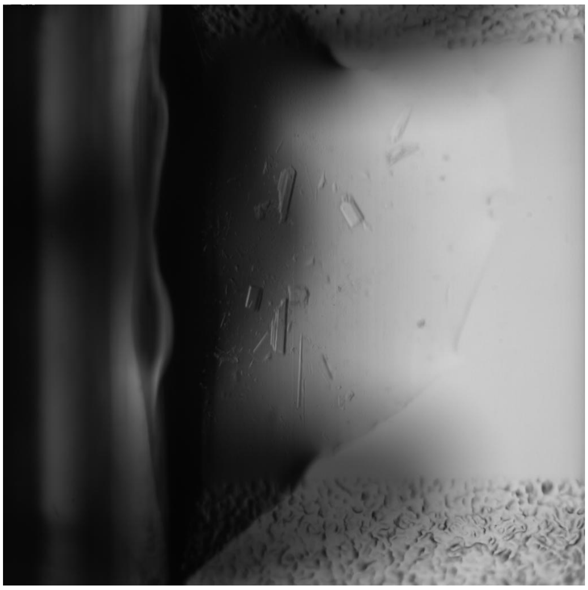

(b)

Figure 126: An ROC Curve for set 'SpeedET-FJ5490C-reu-196-1-196' along with its highest ranked diffraction success.

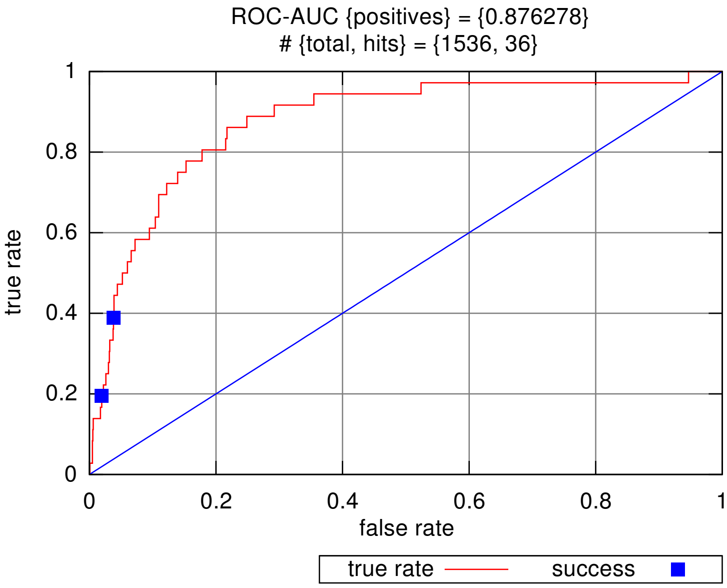

(a)

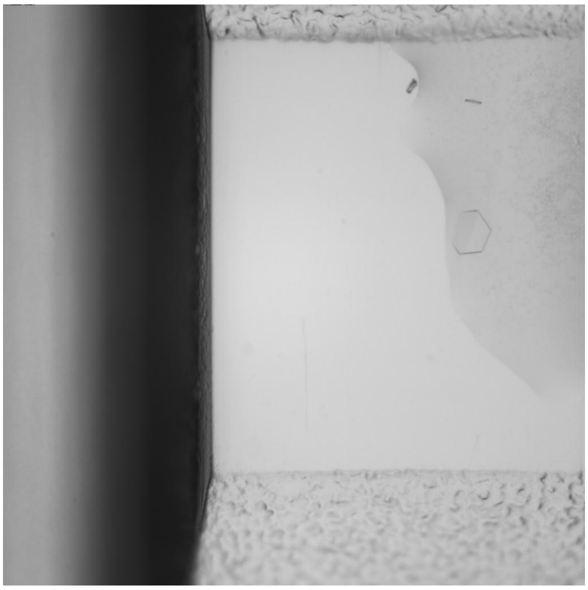

(b)

Figure 127: An ROC Curve for set 'SpeedET-FJ9406A-dra-189-1-189' along with its highest ranked diffraction success.

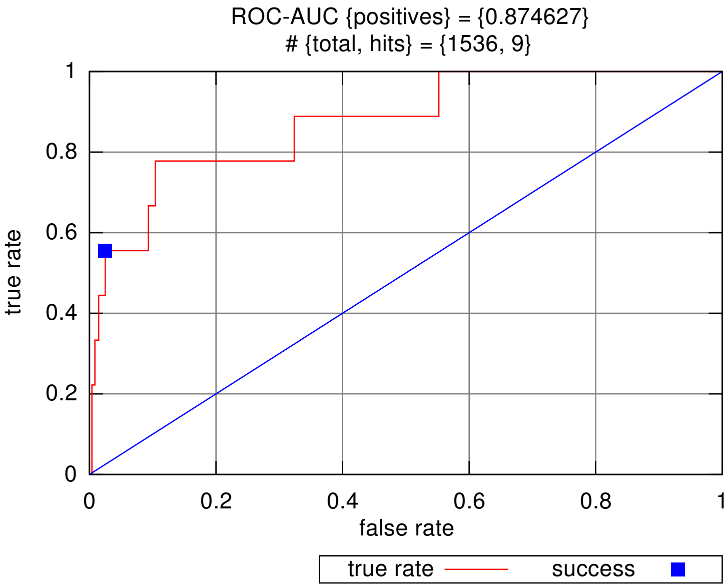

(a)

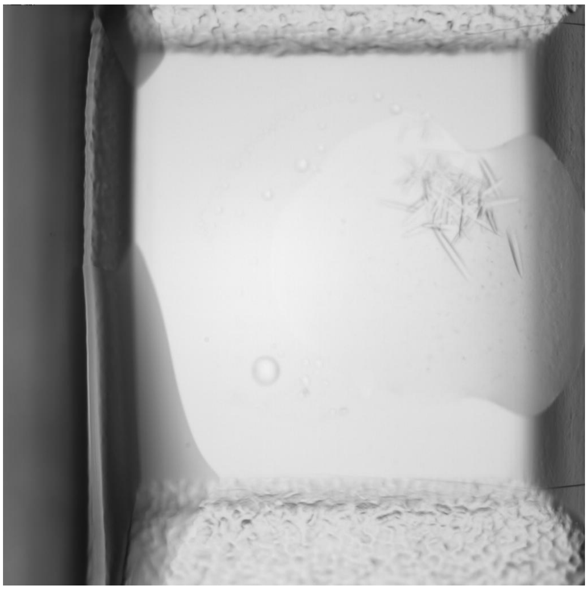

(b)

Figure 128: An ROC Curve for set 'SpeedET-FG7328A-jsp-162-1-162' along with its highest ranked diffraction success.

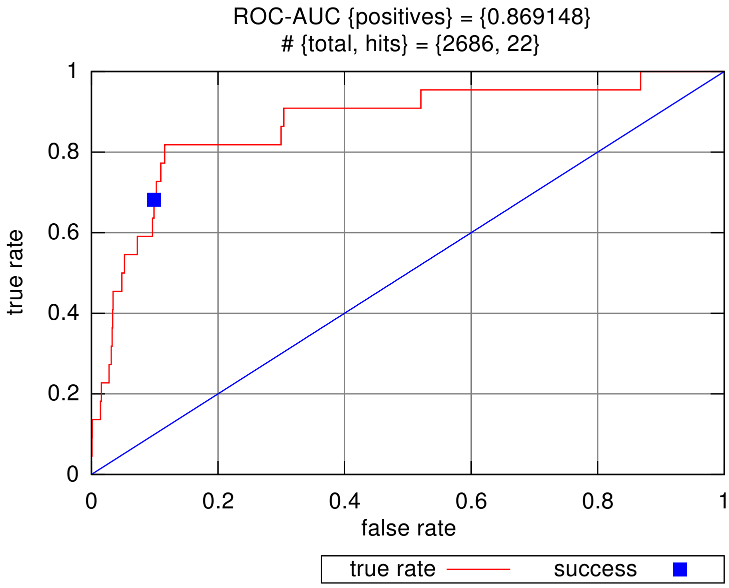

(a)

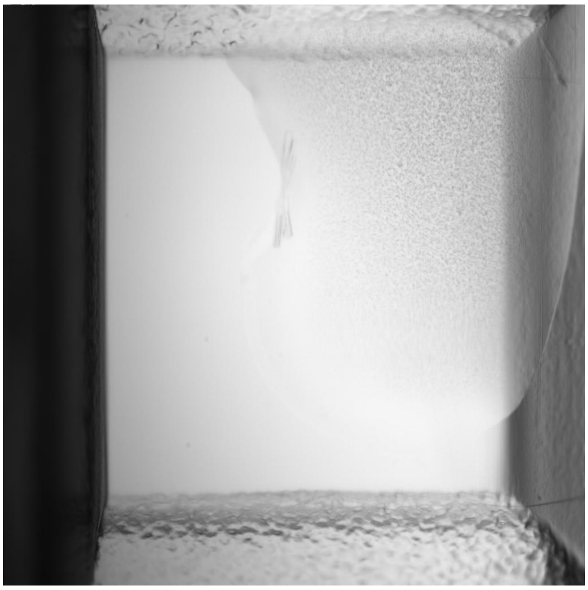

(b)

Figure 129: An ROC Curve for set ‘SpeedET-PG9822C-ssp-125-1-125’ along with its highest ranked diffraction success.

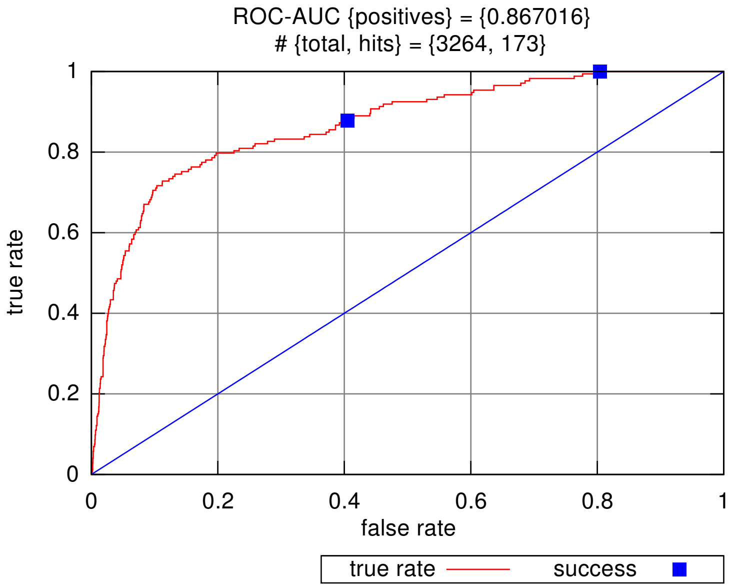

(a)

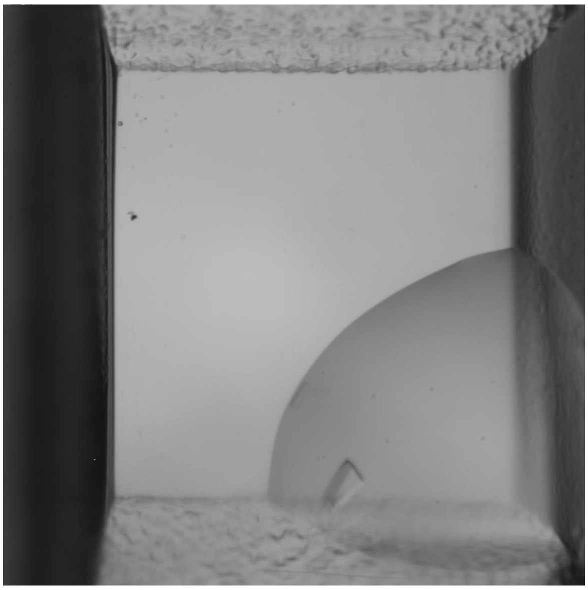

(b)

Figure 130: An ROC Curve for set ‘SpeedET-PG9920A-xca-175-1-175’ along with its highest ranked diffraction success.

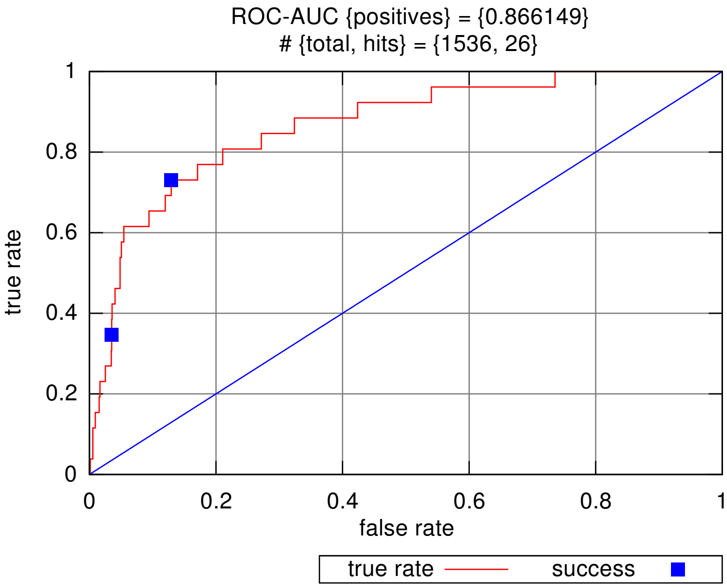

(a)

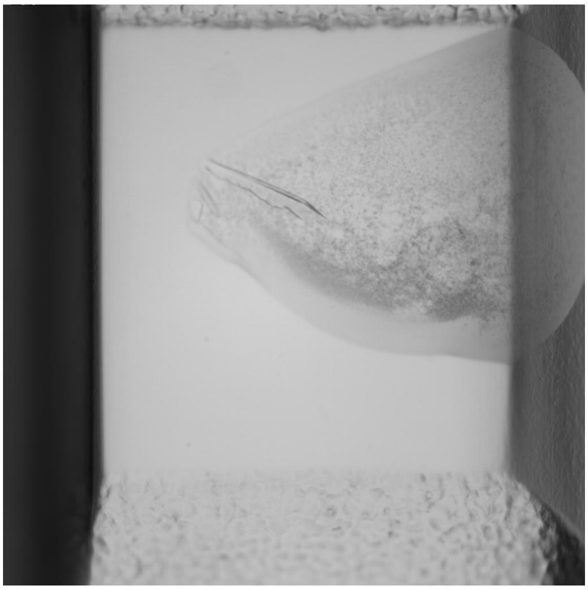

(b)

Figure 131: An ROC Curve for set 'SpeedET-PH10071A-ape-94-1-94' along with its highest ranked diffraction success.

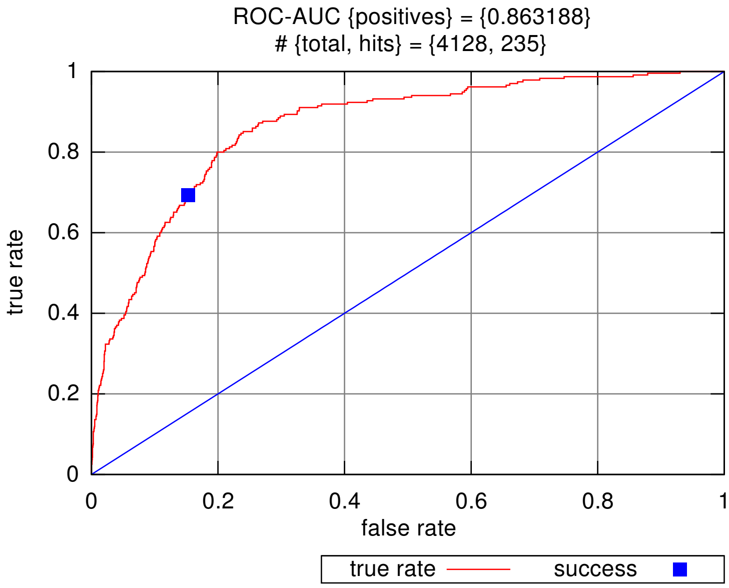

(a)

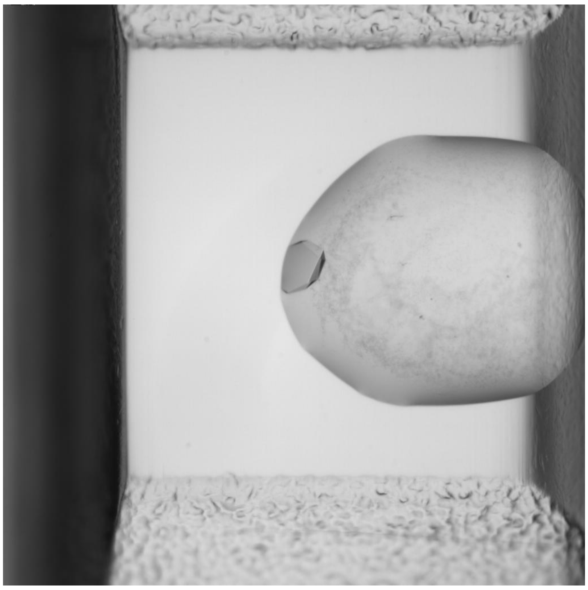

(b)

Figure 132: An ROC Curve for set 'SpeedET-FJ9329A-ava-200-1-200' along with its highest ranked diffraction success.

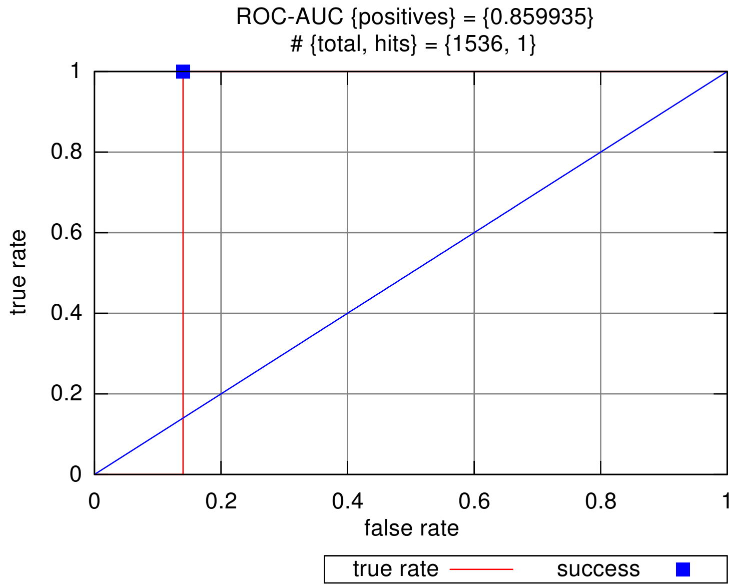

(a)

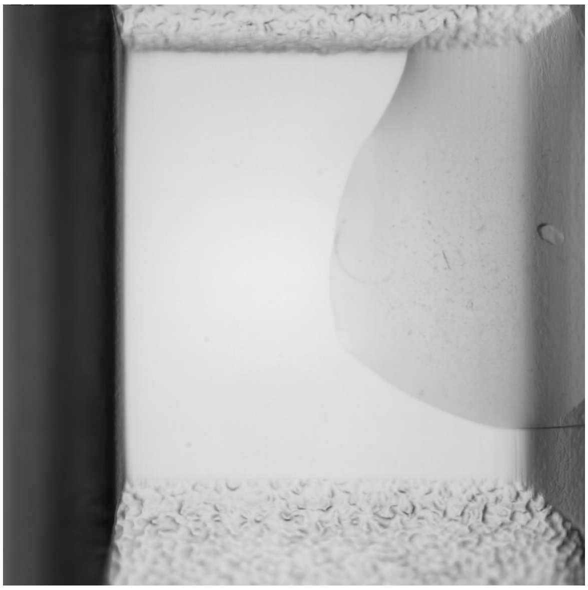

(b)

Figure 133: An ROC Curve for set 'SpeedET-PE00139F-gsu-133-1-133' along with its highest ranked diffraction success.

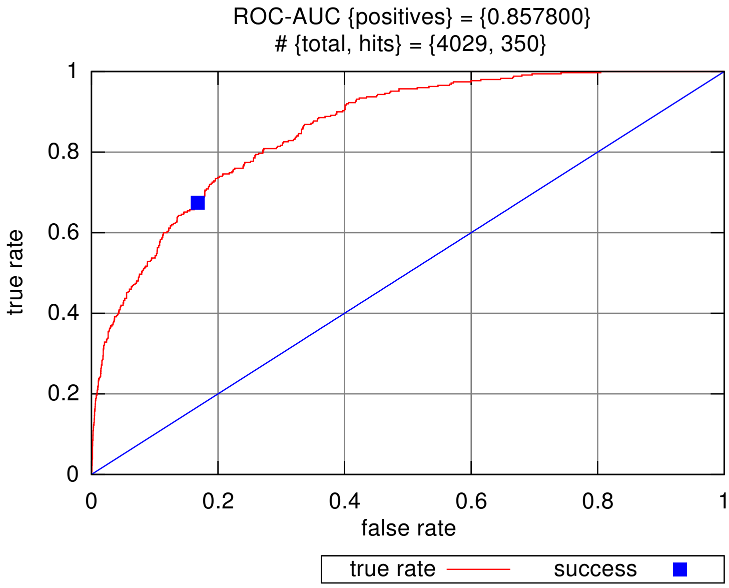

(a)

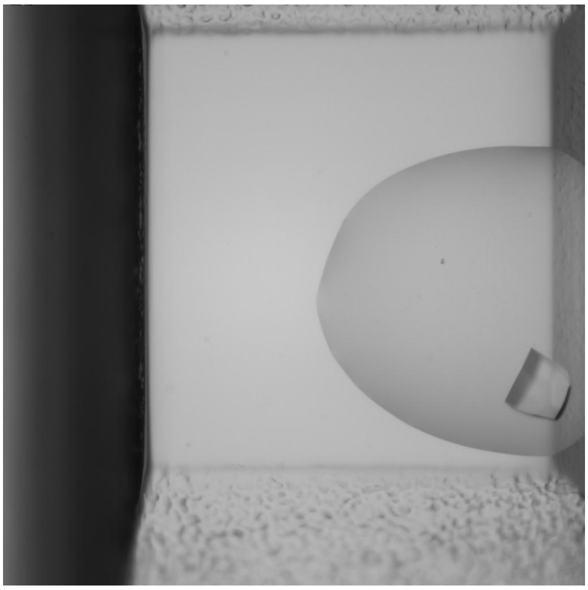

(b)

Figure 134: An ROC Curve for set 'SpeedET-PG9969A-sdn-137-1-137' along with its highest ranked diffraction success.

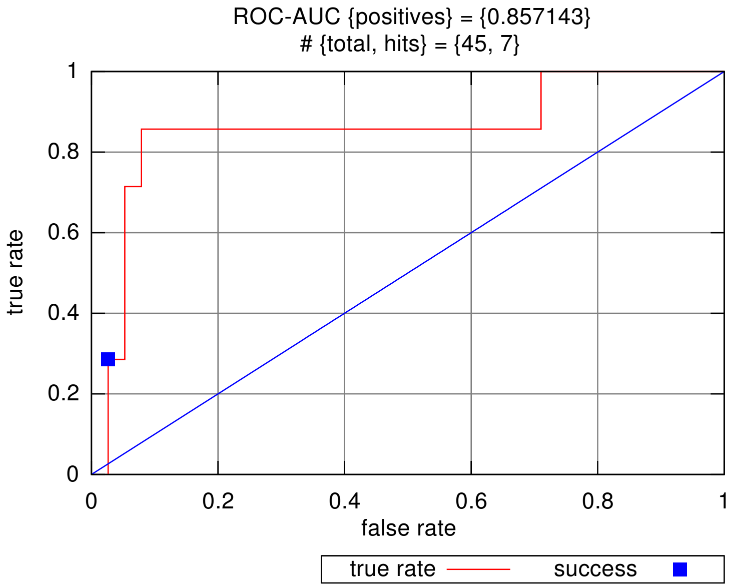

(a)

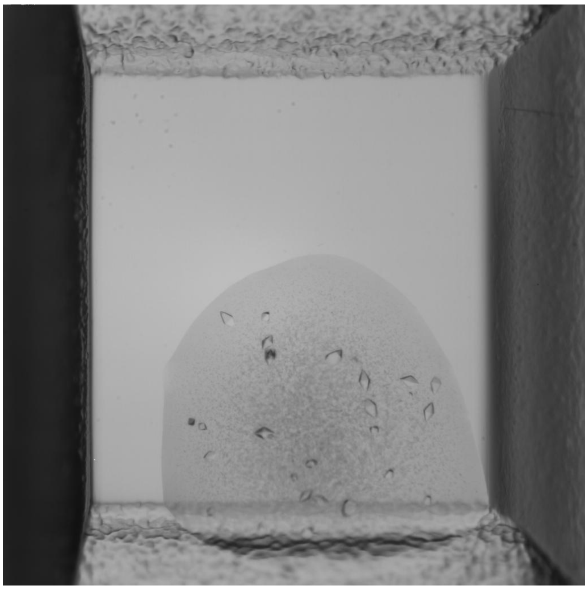

(b)

Figure 135: An ROC Curve for set ‘MH1-TM0015-tma-192-1-192’ along with its highest ranked diffraction success.

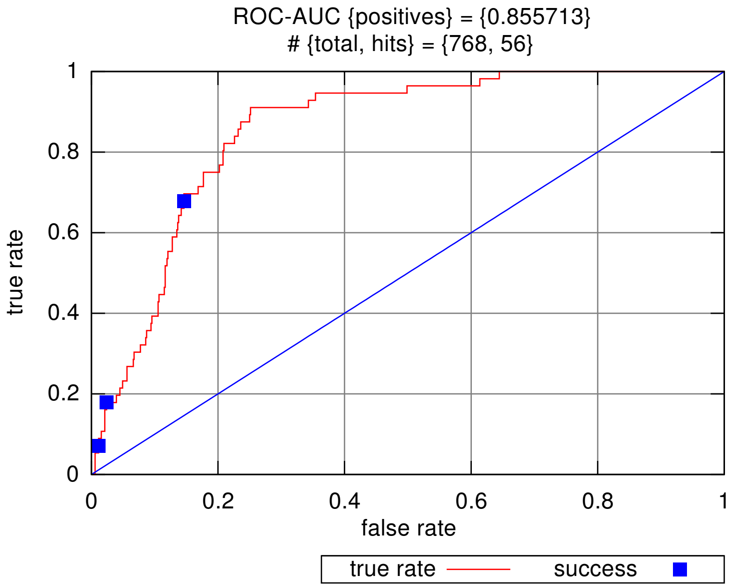

(a)

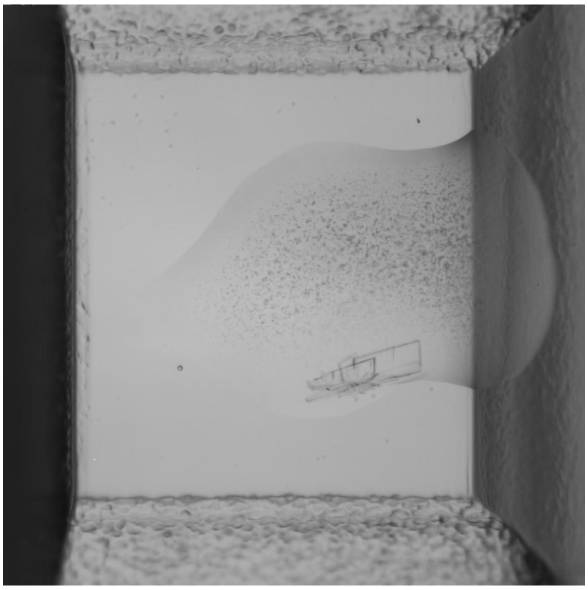

(b)

Figure 136: An ROC Curve for set ‘SpeedET-PE00012A-bsu-152-1-152’ along with its highest ranked diffraction success.

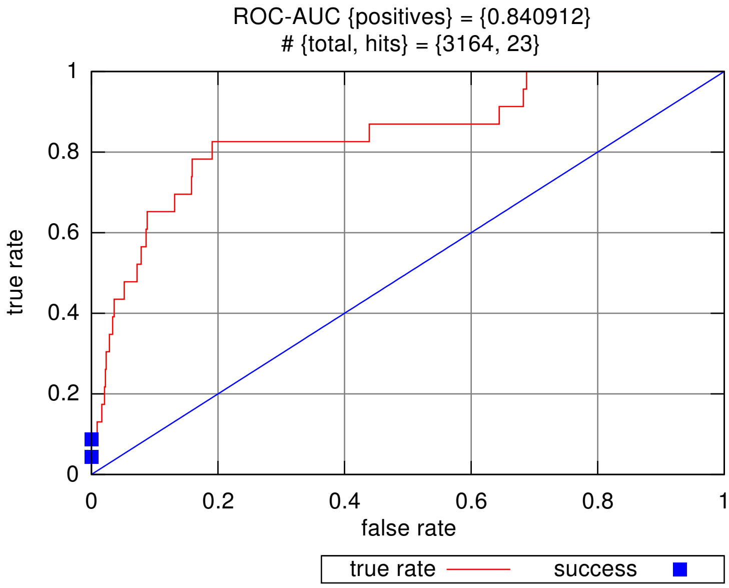

(a)

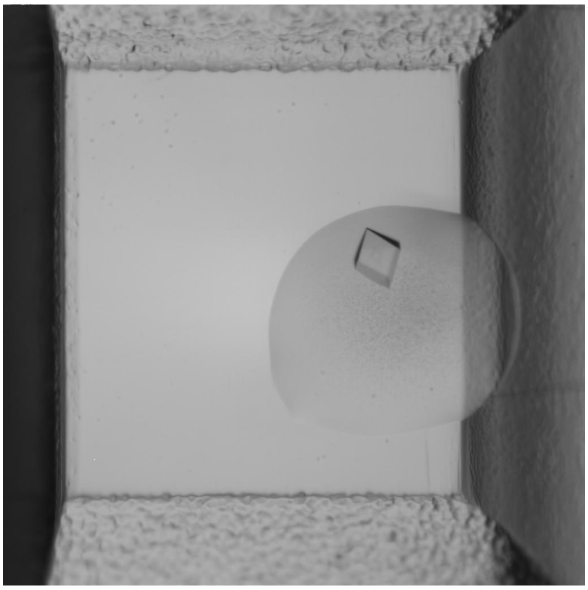

(b)

Figure 137: An ROC Curve for set ‘SpeedET-FJ8839A-par-136-1-136’ along with its highest ranked diffraction success.

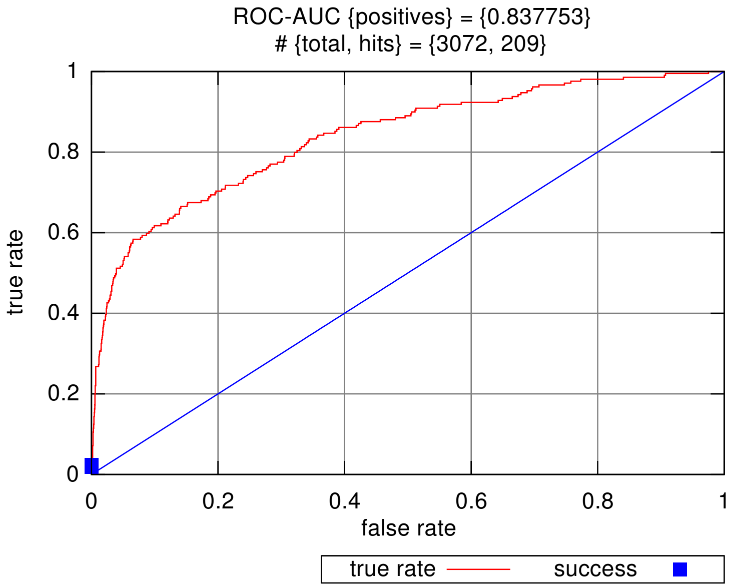

(a)

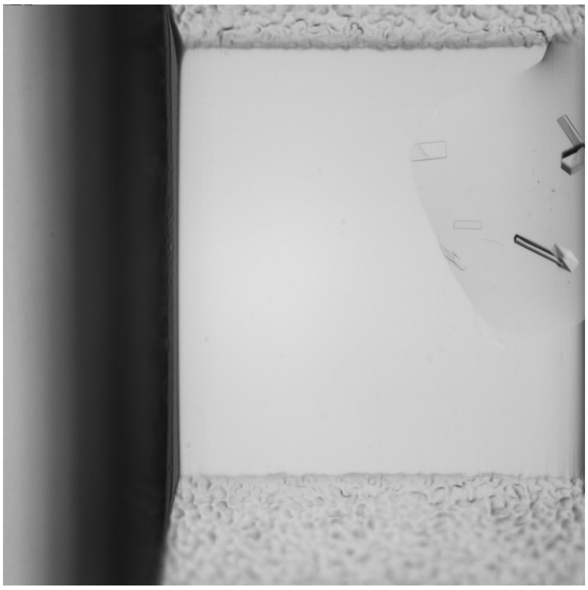

(b)

Figure 138: An ROC Curve for set 'SpeedET-FG7303A-dha-113-1-113' along with its highest ranked diffraction success.

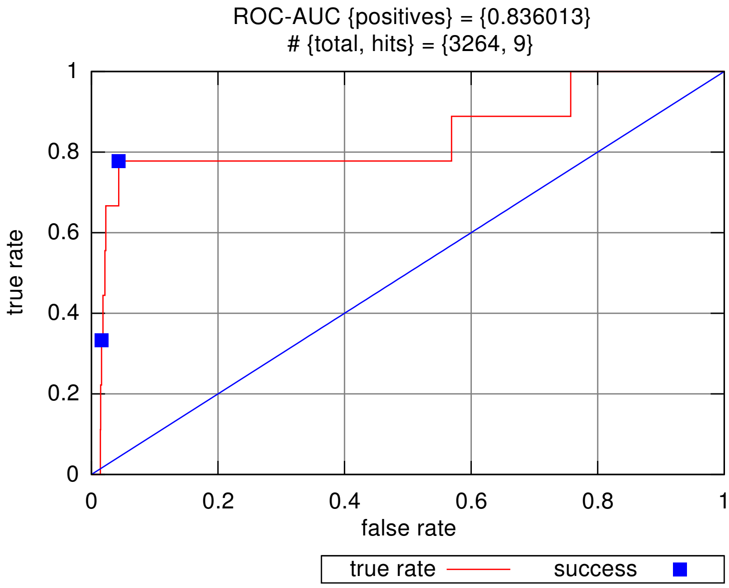

(a)

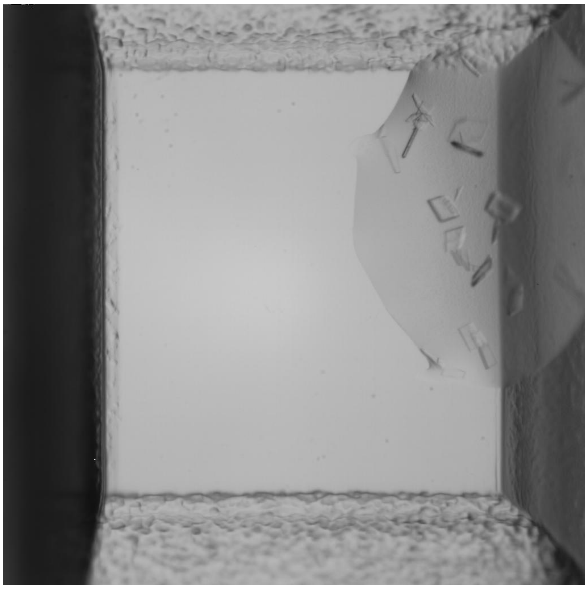

(b)

Figure 139: An ROC Curve for set ‘SpeedET-FG7298A-dde-109-1-109’ along with its highest ranked diffraction success.

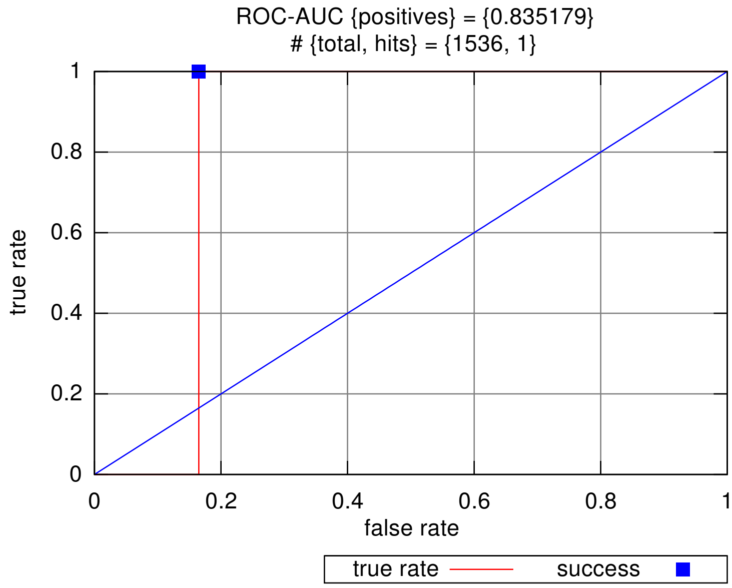

(a)

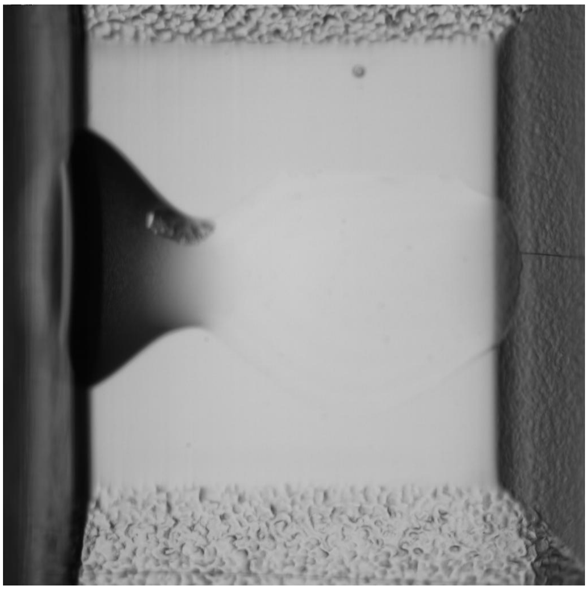

(b)

Figure 140: An ROC Curve for set ‘SpeedET-PC06249B-sty-229-98-229’ along with its highest ranked diffraction success.

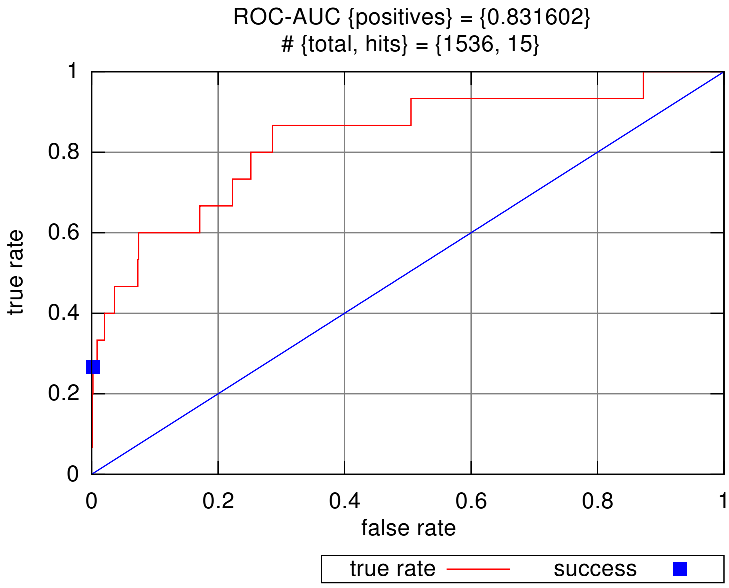

(a)

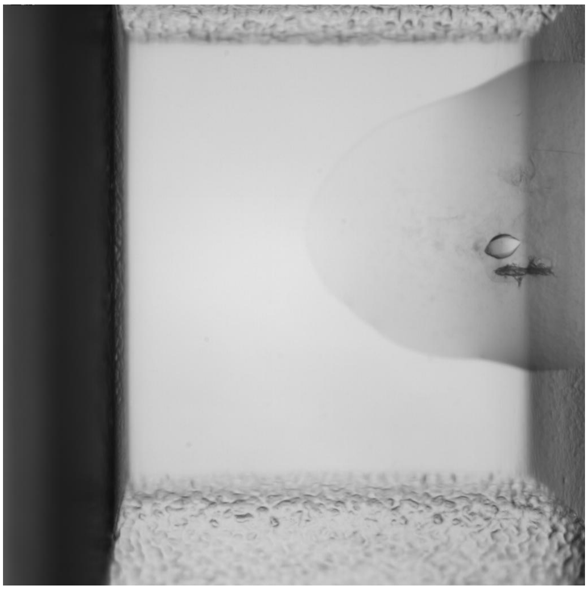

(b)

Figure 141: An ROC Curve for set ‘SpeedET-FH7684A-lic-121-1-121’ along with its highest ranked diffraction success.

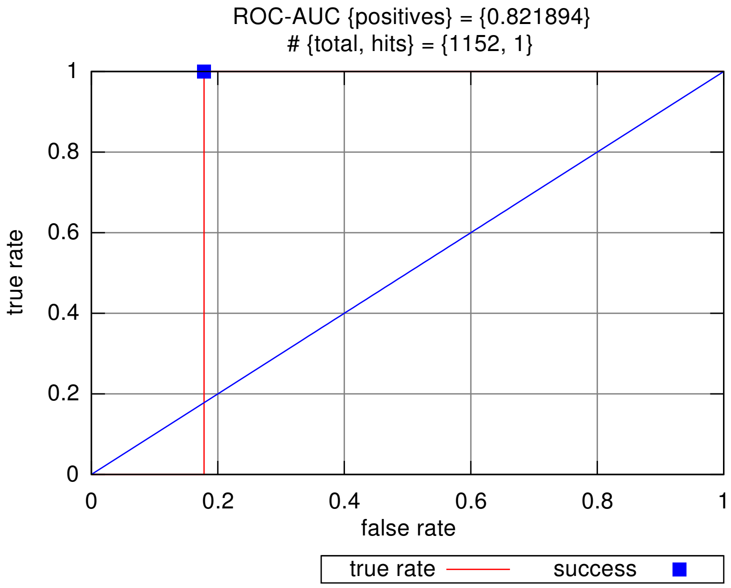

(a)

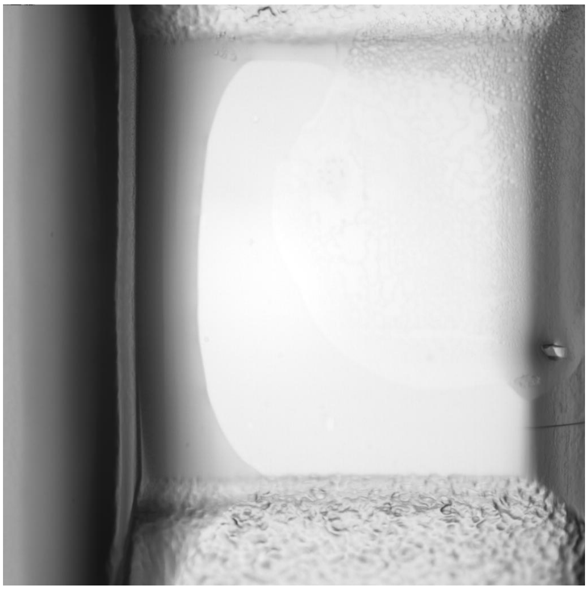

(b)

Figure 142: An ROC Curve for set 'SpeedET-PJ04672A-tfu-273-1-273-A25V' along with its highest ranked diffraction success.

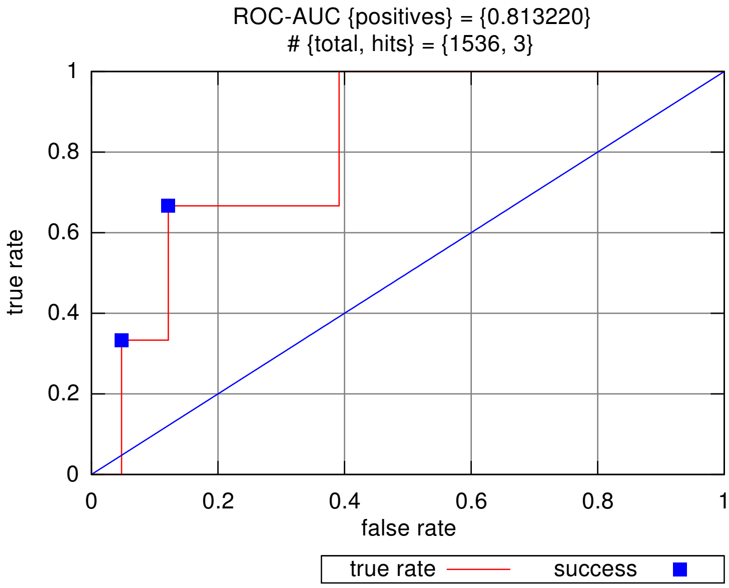

(a)

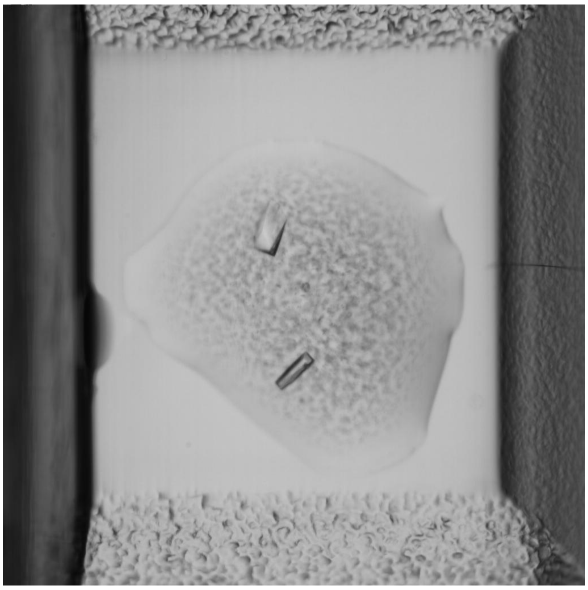

(b)

Figure 143: An ROC Curve for set ‘SpeedET-PH10070D-sam-144-1-144’ along with its highest ranked diffraction success.

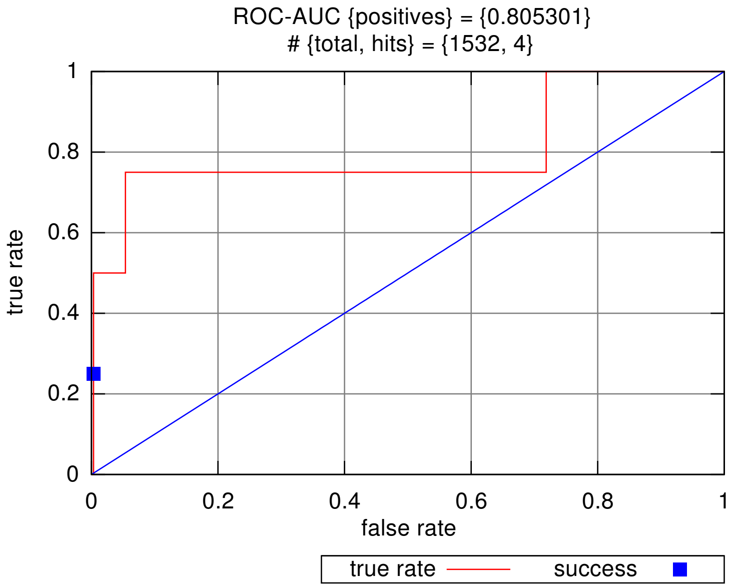

(a)

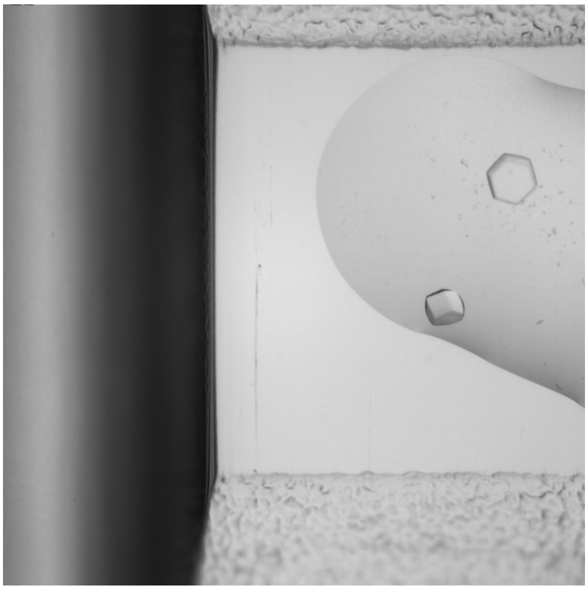

(b)

Figure 144: An ROC Curve for set 'SpeedET-FJ8891A-reu-95-1-95' along with its highest ranked diffraction success.

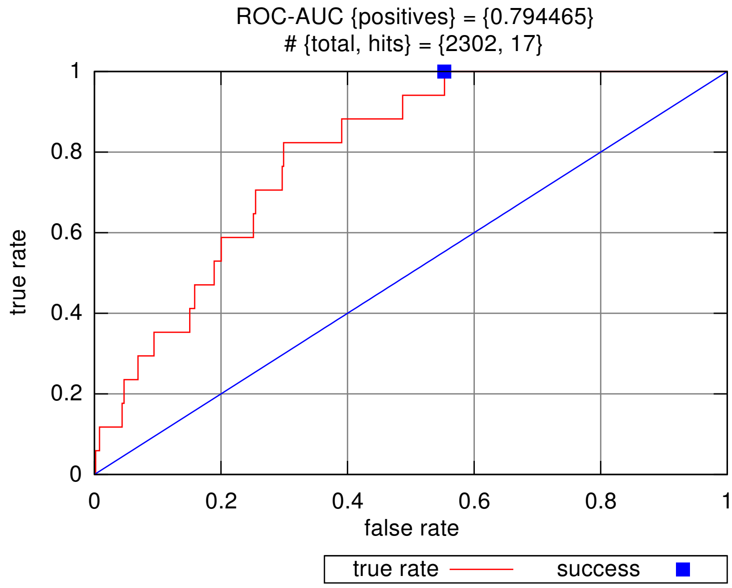

(a)

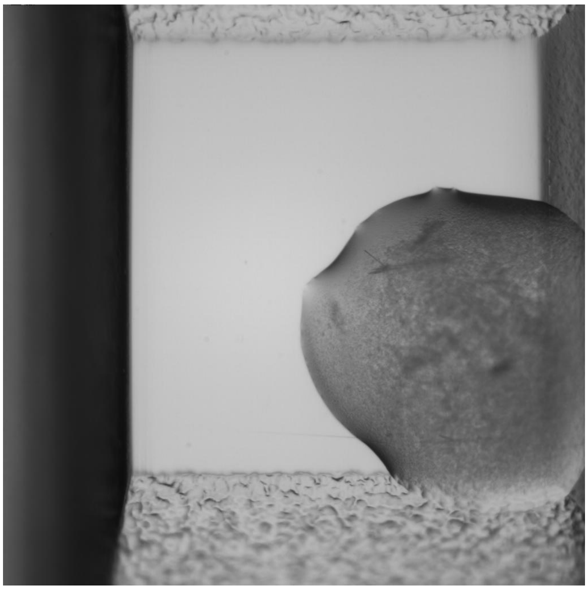

(b)

Figure 145: An ROC Curve for set 'SpeedET-CM8004D-mfl-408-1-408' along with its highest ranked diffraction success.

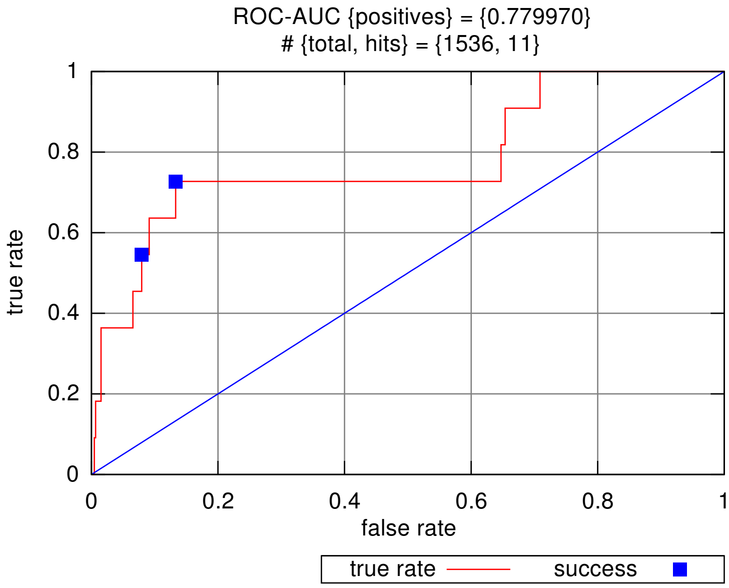

(a)

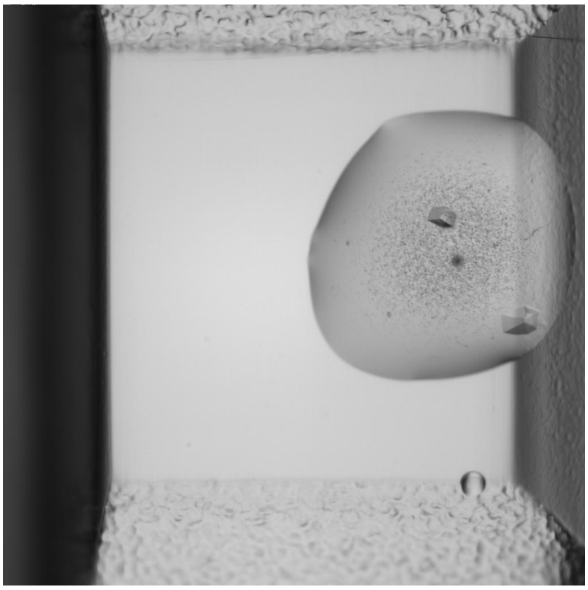

(b)

Figure 146: An ROC Curve for set 'SpeedET-FH7614A-cac-131-1-131' along with its highest ranked diffraction success.

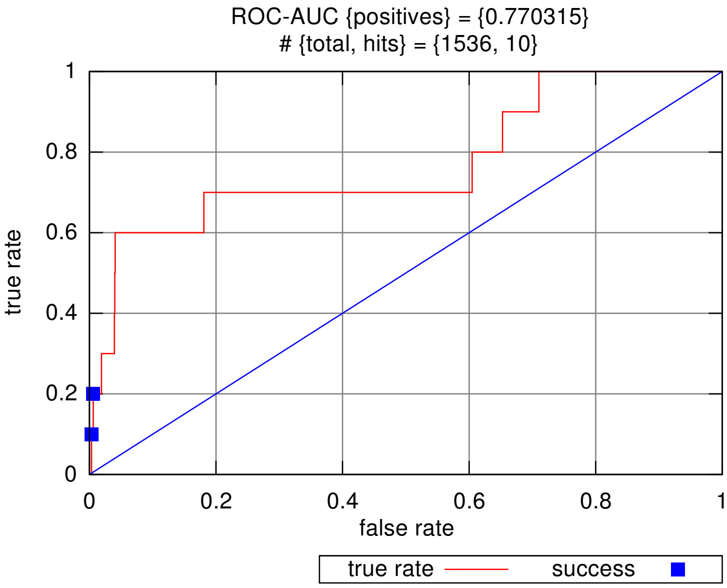

(a)

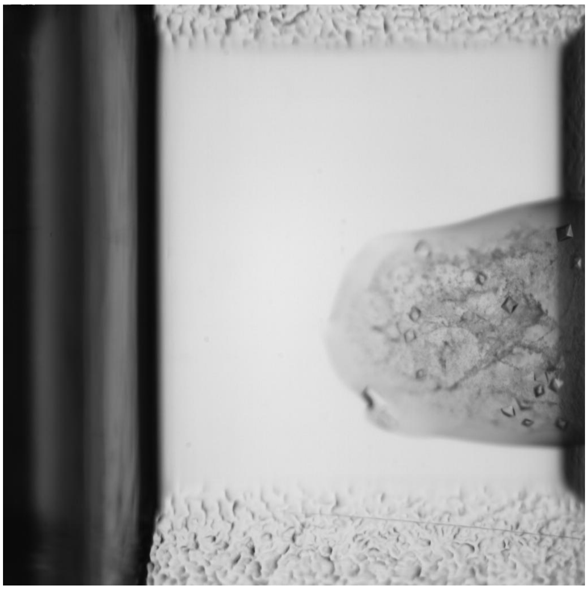

(b)

Figure 147: An ROC Curve for set 'SpeedET-FK9098B-eca-231-1-231' along with its highest ranked diffraction success.

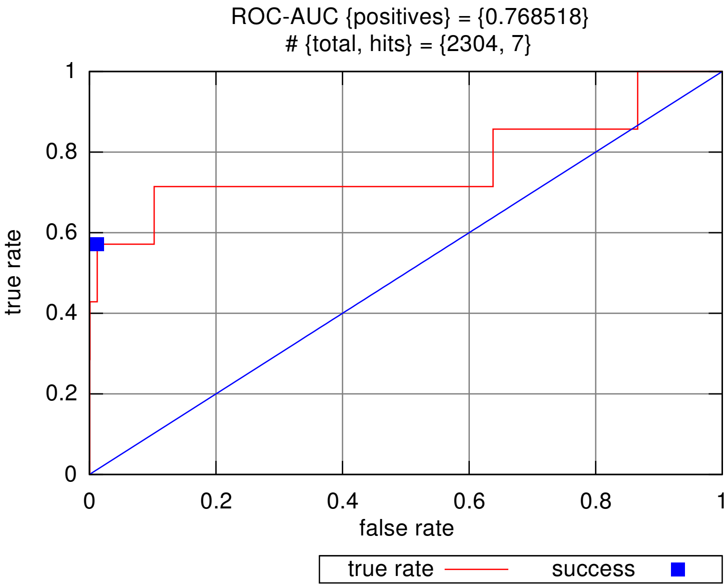

(a)

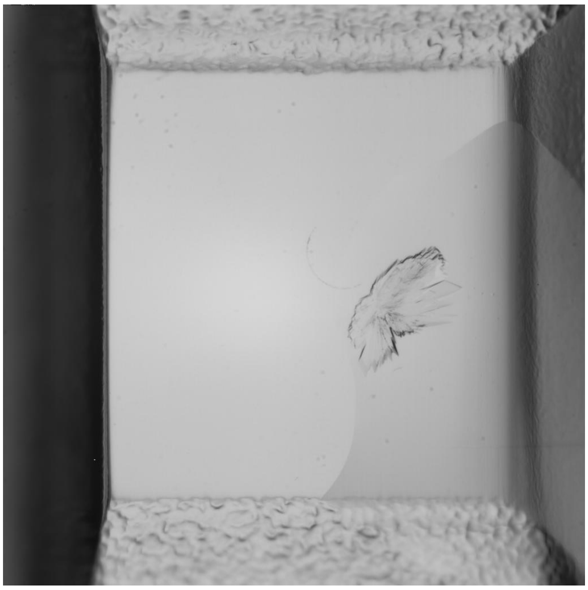

(b)

Figure 148: An ROC Curve for set ‘SpeedET-NP_841447.1-neu-356-23-356’ along with its highest ranked diffraction success.

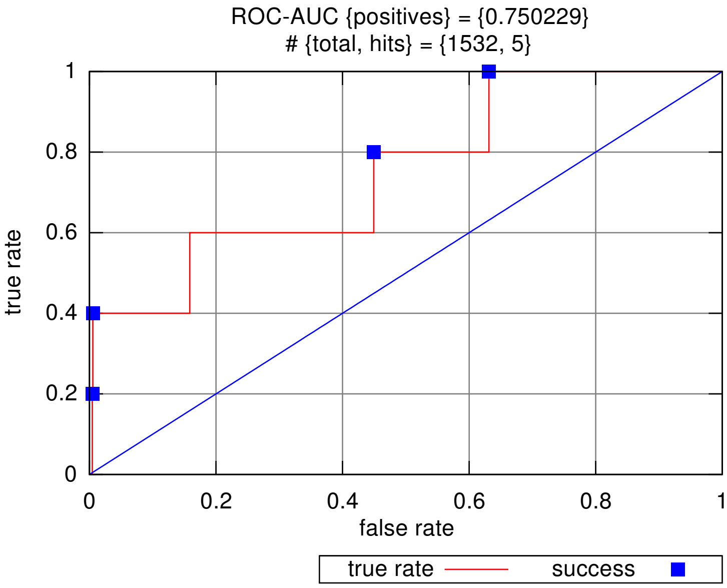

(a)

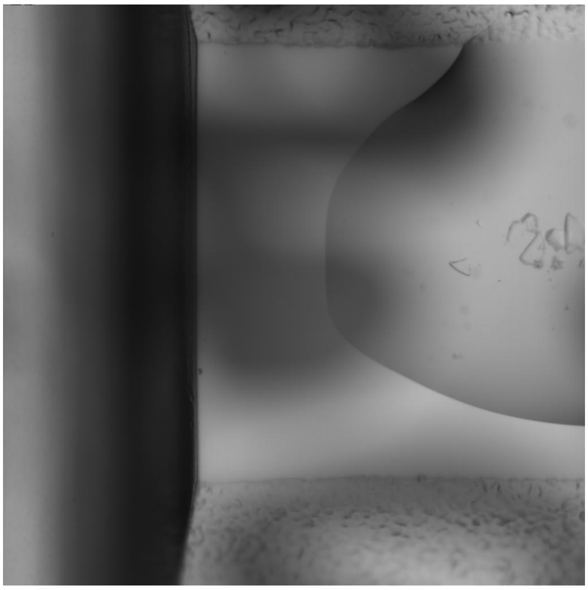

(b)

Figure 149: An ROC Curve for set 'SpeedET-FK9436A-lmo-178-1-178' along with its highest ranked diffraction success.

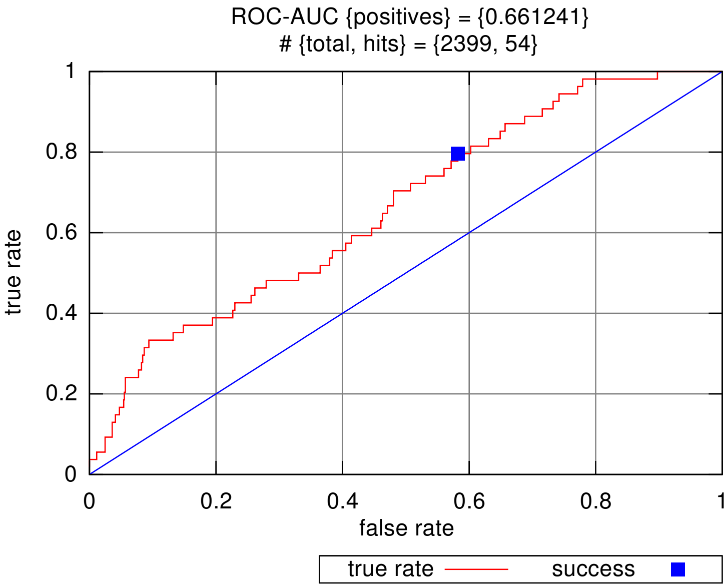

(a)

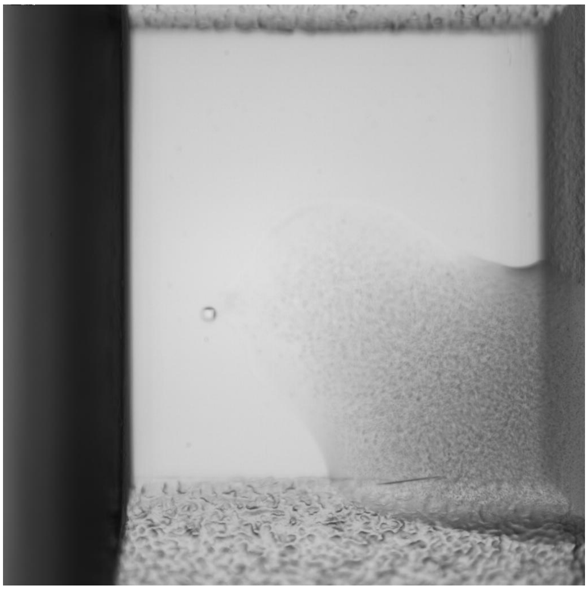

(b)

Figure 150: An ROC Curve for set ‘SpeedET-ME9797A-tba-219-1-219’ along with its highest ranked diffraction success.
