# Supplementary material for: Image-based crystal detection: a machine-learning approach
Source: Acta Crystallogr D Biol Crystallogr. 2008 Nov 18;64(Pt 12):1187–95. doi: 10.1107/S090744490802982X (PMC2585161; doi:10.1107/S090744490802982X)
Supplement: Supplementary file 3 [file d-64-01187-sup3.pdf]

Supplementary Data for "Image-Based Detection of Crystals: A Machine Learning Approach"

Roy Liu
University of California at San Diego
royliu@cs.ucsd.edu

Yoav Freund
University of California at San Diego
yfreund@cs.ucsd.edu

Glen Spraggon
The Joint Center for Structural Genomics
& The Genomics Institute of the Novartis Research Foundation
spraggon@gnf.org

1. System Handbook

1.1 Obtaining Components

The Microcrystals image analysis system consists of four groups of components, listed in increasing order of generality:

- Benchmark image sets available on request from the authors, along with training images and annotations. Use these to replicate the simulations in the paper and/or benchmark the system before adaptation to your laboratory's images. **We look to have a freely accessible web-based repository of all the data within two months.**
- Subversion repository code that one can anonymously check out from http://shared.googlecode.com/svn/trunk/. You will find two subdirectories there, Microcrystals and Shared – the first consists of core code for scoring images, training classifiers, and annotating training examples; the second consists of support code from the Shared Scientific Toolbox (SST, http://shared.sourceforge.net/). Alternatively, one may contact the authors and request an archive. Note that JBoost (http://jboost.sourceforge.net/), the machine learning package behind the boosting algorithm described in the paper, is bundled with the core code, and does not require a separate download.
- A Java Development Kit (JDK) from Sun Microsystems of version 1.6 or greater. Dependencies of the SST (FFTW [1], C++, Make, Perl) are required by extension. To render figures, our system uses Gnuplot and Graphviz. We strongly recommend a Unix-like operating system simply because of the ubiquity of the above components; a Windows installation is entirely feasible, and requires no modifications to the actual source code. We assume the availability of a MySQL database, although most other transactional databases will do.

1.2 Installation Steps

Once the user has assembled the required components, installation is straightforward:

1. Check out or unpack the Microcrystals core code into work/Microcrystals/, where work/ is a directory of your choosing. Likewise, check out or unpack the SST support code into work/Shared/.
2. Follow the installation instructions in the SST manual to build everything in work/Shared/. Be sure to run the JUnit testing script test.pl afterward.
3. Change into work/Microcrystals/ and copy src/db.cfg.xml to src_custom/my.cfg.xml. Edit the *following fields only*. For further assistance, please consult the Hibernate literature.

```
<!-- Change this only if you use a non-MySQL transactional database. -->
<property name="dialect">org.hibernate.dialect.MySQLInnoDBDialect</property>

<property name="hibernate.connection.url">jdbc:mysql://HOST_NAME/DB_NAME</property>
<property name="hibernate.connection.username">USER_NAME</property>
<property name="hibernate.connection.password">USER_PASS</property>
```

4. Type make. This will pull SST class files from ../Shared/bin/, compile your customizations in src_custom, and compile core code found in src. Note that src_custom contains code specific to the benchmark image sets. Once you are ready to adapt the system to laboratory-specific conditions, see Section 1.3. Typing make javadoc will create documentation in

javadoc/. Run the 0-demo.pl script and verify that image processing modules are working, and that a directory demo/ exists with demonstration outputs in it.

5. Type make ddl. This will generate a SQL script db.sql specifying the database schema (see Figure 3) used for managing image annotations and scores. Apply this script to your database server.

1.3 Customizing the System

To adapt Microcrystals to laboratory-specific conditions, users will need to compile customizations into the system. We have set aside a directory, src_custom/, for holding the most common types of customizations – namely, machine generated classifier code (obtained from Section 3) and handcrafted preprocessor code (described in Section 5). Simply place your files in their appropriate places in the package hierarchy (Java source files belonging to package foo.bar.baz should go in src_custom/foo/bar/baz) and type make to rebuild everything.

1.4 Usage

The system uses Perl scripts to start up various administration programs. We describe of each script along with the arguments it recognizes, and encourage users to follow cross references for a more complete understanding.

0-demo.pl demonstrates image processing routines.

1-extraction.pl extracts features for learning. Uses parallel data flow techniques (Section 2.2) and registers feature vectors with the database as entries of the FeatureVector table (Figure 3). Runs until all user-generated square annotations have an associated feature vector.

- **–mode** required; sets the utility program run mode. Use the extraction switch for feature extraction.
- **–configuration** required; tells Hibernate the custom configuration file location on the Java class path. In other words, if you named it src_custom/my.cfg.xml (Section 1.2), then the argument would be my.cfg.xml.
- **–wisdom** optional; points FFTW to a file where precomputed wisdom from the past may be stored. Using wisdom is highly recommended; although precomputation times are long, they result in potential future speedups of 100%. Upon exit of the virtual machine, the utility program will write accumulated "wisdom" back to said file.

2-xvalidation.pl performs k-fold cross validation. Reads from the FeatureVector table to retrieve extracted feature vectors. Outputs a learned classifier source file and performance statistics for each fold (Section 3). One such machine-generated classifier may be installed as a user customization (Section 1.3) and used for image scoring.

- **–mode** required; use the xvalidation switch.
- **–configuration** required.
- **–work** required.
- **–nrounds** required; the number of boosting rounds.
- **–nfolds** required; the number of cross validation folds.

3-registration.pl commits an image set folder to the database. Registers an entry in the WorkingSet table and then adds images to the Image table, along with their associated WorkingSetImage table entries (Figure 3).

- **–mode** required; use the registration switch.
- **–configuration** required.
- **–work** required; the image set folder you wish to commit. The name of the folder will serve as the name of the newly added WorkingSet entry.
- **–regex** optional; only images with names matching the regular expression are considered.

4-scoring.pl starts a scoring daemon (Figure 2).

- **–mode** required; use the scoring switch.
- **–configuration** required.
- **–wisdom** optional.
- **–threshold** required; an image scoring above this threshold will be put forth for human validation with the user interface (Figure 1).
- **–nthreads** optional; the number of threads to use.
- **–predictor** required; the Java class name of the classifier (Section 1.3).
- **–pp** optional; the Java class name of the preprocessor (Section 1.3).

5-reporting.pl reports scoring performance statistics for all image sets. Generates figures like the ones seen in the paper.

–**mode** required; use the reporting switch.
–**configuration** required.
–**ratingThreshold** required; the integer-valued ground truth threshold above which an image is considered as a diffraction candidate.
–**ratingDiffract** required; the integer-valued ground truth threshold above which an image is considered as a diffraction success.
–**groundTruth** optional; instructs the reporter to read ground truth labels from the command line according to the template "image name" "ground truth score" for every line.

6-applet.pl starts the training image annotator (Figure 1). Be sure to edit the "configuration" parameter field in src_custom/applet.html to point to your Hibernate configuration file, as the user interface interacts with the database.

One can score images and train classifiers in a few easy steps. To score images:

1. For a folder of images, call it image_set, type ./3-registration.pl image_set. The result will be a WorkingSet structure in the database named image_set joined to its Image entries via intermediate WorkingSetImage entries.
2. Denote that you would like all images associated with the newly created WorkingSet entry to be eligible for scoring by setting its active field to the value T for true.
3. Start up as many daemon processes as you'd like by invoking the command 4-scoring.pl. The daemon will attempt to find work by joining any working sets with active field set to T to images that have not yet been scored. Once an image has been scored, its associated WorkingSetImage entry will have its active field toggled from F to T (the term "active" here is overloaded to indicate whether or not an image has been scored).

To train:

1. Type ./6-applet.pl to start the training image annotator. You will be presented with all images associated training annotations. Using the program, you may delete, add, and modify annotations.
2. Human generated postives and negatives will serve as training examples for machine learning algorithms. Use the 1-extract.pl script to process training images and their annotations.
3. Type ./2-xvalidate.pl to perform k-fold cross validation over the extracted features. The results should be a multitude of cross validation fold performance statistics (Figure 5), as well as learned classifiers (Figure 6), which can then be used to score more images.

1.5 The Graphical User Interface

The graphical user interface included in our system, captured in Figure 1, serves both as a visualization tool for training image annotations and as part of a process to quickly generate new, informative training examples. As scoring algorithms sift through a novel image set, they attach computer-generated crystal positive square annotations to potentially interesting images. Such images, usually determined by their scoring over a user-defined threshold, appear to the user for validation. In the absence of ground truth labels, the above policy, when executed with generation i of classifiers, aims to find images with crystalline material that would yield an abundance of training examples for generation $i + 1$ of classifiers. Thus, we envision that users would engage the interface to bootstrap specialized classifiers from training examples put forth by initial, suboptimal classifiers.

Although still under development, the user interface is sufficient for the purposes of annotating training examples. Notice that the detached menu at the top of Figure 1 (shown on top of the annotated image because of space constraints) has three divisions – "training" for images with associated human positives and negatives; "validation" for images with associated computer proposed positives; and "empty" for orphaned images that, during some time in their lifecycle, had all of their annotations removed.

Selecting one of the sets above will load all of its images, which are browsable with the "next" and "previous" buttons. Clicking on the image in an area with annotations with the left mouse button creates a positive annotation. Conversely, clicking with the right mouse button (or equivalent) will create a negative annotation. One may also drag to create a selection box and delete annotations using the "delete" key on the keyboard. Note that computer positives are placeholders only – they do not substantively affect training procedures; our system leaves the user to validate every computer flagged image with annotations of his or her own choosing.

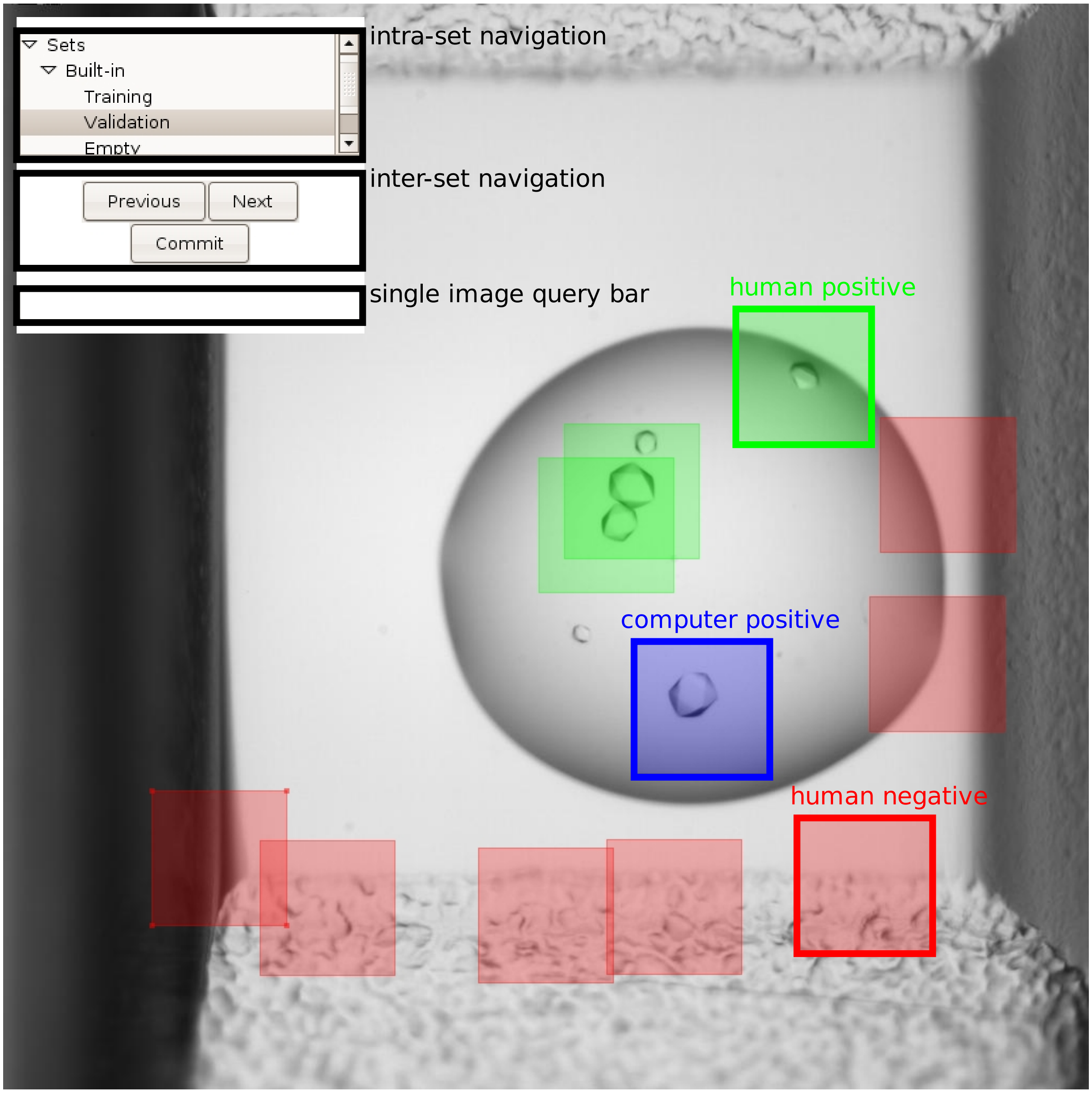

Figure 1. The user interface provides a convenient way to manage current training annotations (human positives and negatives), as well as validate computer-proposed positives. The minimalist design enables browsing among image sets in the "intra-set navigation" window, browsing within image sets with the "inter-set navigation" buttons, and retrieval of individual images with the "single image query bar".

2. System Organization

2.1 Distributed Computing

Our system maintains a centralized, transactional database, appearing as "database" in Figure 2, to coordinate score submissions by individual machines. The internal organization of the database, shown in Figure 3, centers on image sets, and contains tables corresponding to the concept of sets, images, and the intermediaries joining the two. Users start as many distributed jobs as needed per the instructions in Section 1.4, and throughput scales proportionately to the number of participating machines. To make sure that high level concept met real world practice, we carried out simulations in the paper according to this workflow.

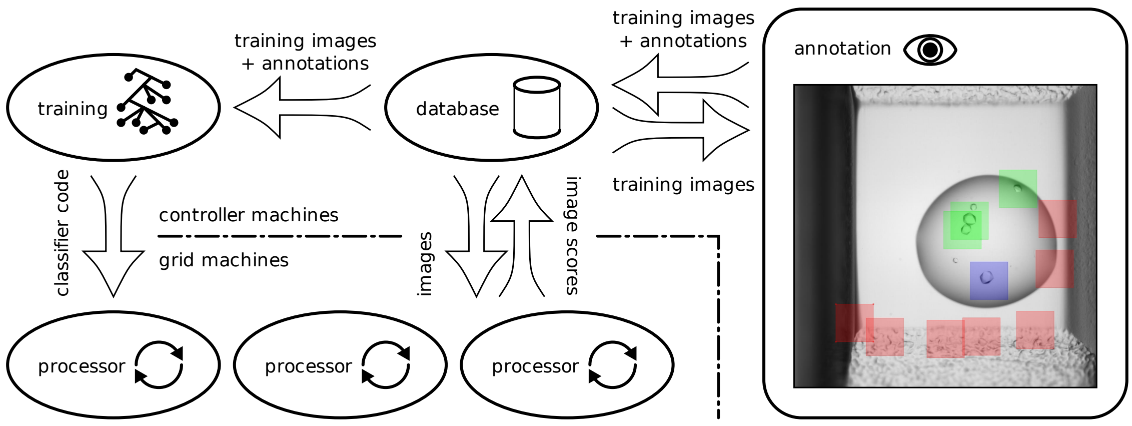

Figure 2. The system architecture. **database)** the database is the heart of the system. It stores common bookkeeping for scoring and training processes; **training)** training processes read images and their annotations from the database, and output classifier code that is compiled and loaded into scoring processes; **processor)** a single processor, indistinguishable from all other processors, reads images from the database and commits them back with scores; **annotation)** the user interface interacts closely with the database so that it can load existing human annotations, while making computer flagged images available to the user for validation.

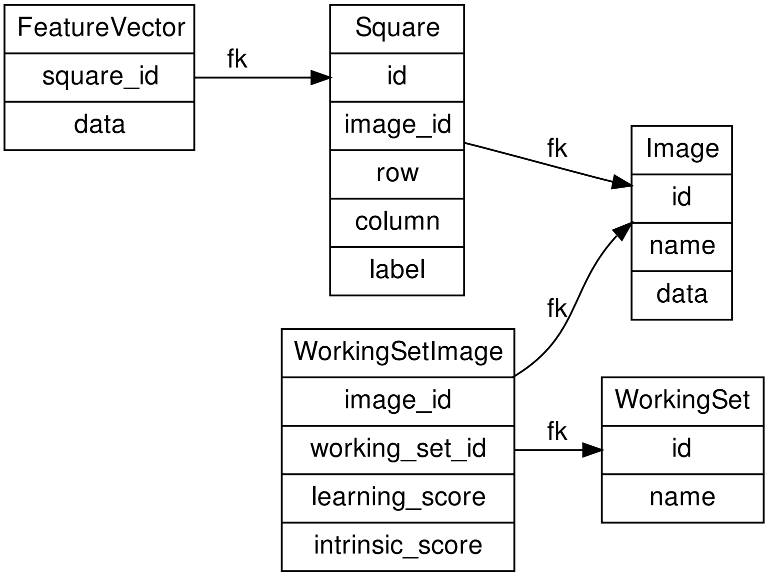

Figure 3. The database schema. **WorkingSet)** a set of images; **Image)** a singleton image, with the image data stored as a blob; **WorkingSetImage)** the join table between WorkingSet and Image. The learning_score field contains the real-valued score assigned by machine learning algorithms, while the intrinsic_score field contains the integer-valued score assigned by human annotators; **Square)** a human positive/negative or computer positive square annotation associated with an image. The row and column fields represent its location in the image.

2.2 Parallel Data Flow

Our system uses a parallel data flow model for feature extraction that takes advantage of the growing number of processing cores on commodity hardware [2]. Simply put, the underlying feature extraction algorithms execute as much of their work in parallel as the data interdependencies allow, freeing the user to specify a computation, rather than having to worry about optimizing its execution. The advantage of a parallel data flow approach goes beyond speed – algorithms like ours

demand the computation of hundreds of features, and hand coding quickly becomes unmanageable. Instead of hand-coding a system, we first break it down into its simplest calculations consisting of Fast Fourier Transforms (FFT) and their aggregations, as seen in Figure 4. We then connect these components together by indicating that the outputs of some are the inputs to others. Aside from their specification, parallel data flow engines require no additional implementation details; given some input, they transparently handle its transformation into observable output, all while exploiting available multi-core parallelism.

We hope that our paradigm encourages future development of feature extraction methods by providing the means to decompose the most complex calculations into their simplest, irreducible components. One can find a more complete description of parallel data flow engines in the SST manual.

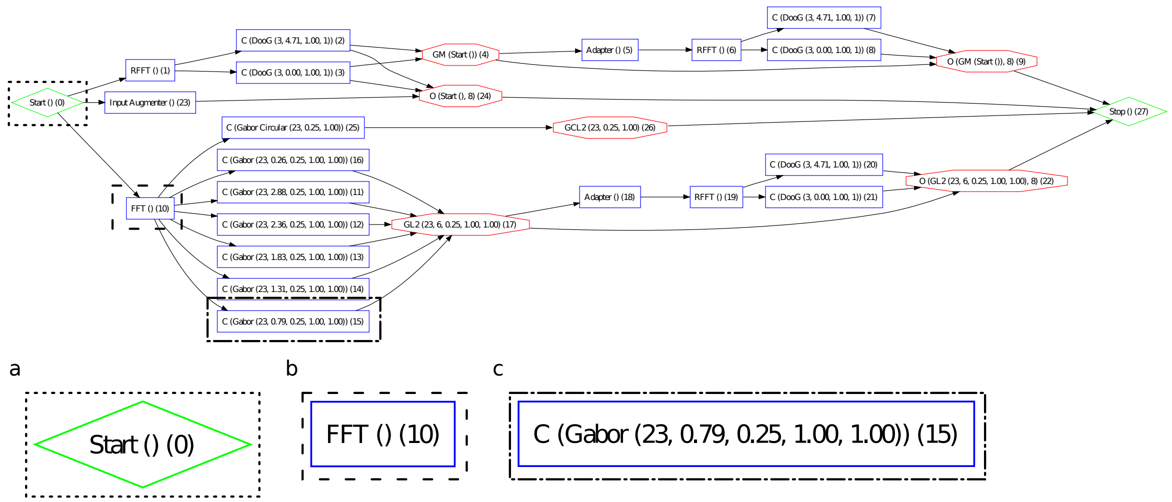

Figure 4. Components of a parallel data flow engine. **a)** the starting node, which distributes the input image to its dependents; **b)** an FFT node, which computes the forwards transform of the image; **c)** a convolution node, which takes the already-transformed image and convolves it with a complex-valued Gabor filter.

3. Analysis

3.1 ROC Analyses

We derive ROC curves by considering image sets in decreasing rank-order – a diffraction candidate increments the true positive rate (TPR) and a discarded trial increments the false positive rate (FPR). We estimate the ROC-AUC score, equivalent to the Wilcoxon statistic, with trapezoidal integration. We carried out one-tailed signicance analyses to calibrate the “worst case” ROC curve via Parzen window analysis with Gaussian kernel (s.d. 0.02) at every FPR for all signicance thresholds to determine the maximum achievable TPR. Note that we do not make any scientic claims here, and use signicance testing as a measurement calibration tool.

3.2 Cross Validation Performance Statistics

Our system auto-generates performance statistics on each fold as a side effect of the cross validation process. Figure 5 depicts the results for a sample fold. These plots aim to help users make decisions on how to tune the boosting algorithm and feature selection. For example, in the boosting history plot, a test set error that suddenly turns upward after many successive rounds of boosting probably means that the algorithm has overfitted the training data. In the score margins CDF, a steep upwards slope means that the boosting algorithm has difficulty discriminating crystals from non-crystals; a low ROC-AUC also is indicative of this problem.

The performance statistics we have derived need not remain dependent on boosting as the core learning algorithm – in fact, any algorithm that outputs a real-valued prediction score falls within its scope. Thus, one may replace boosting with, for example, support vector machines, in a straightforward manner.

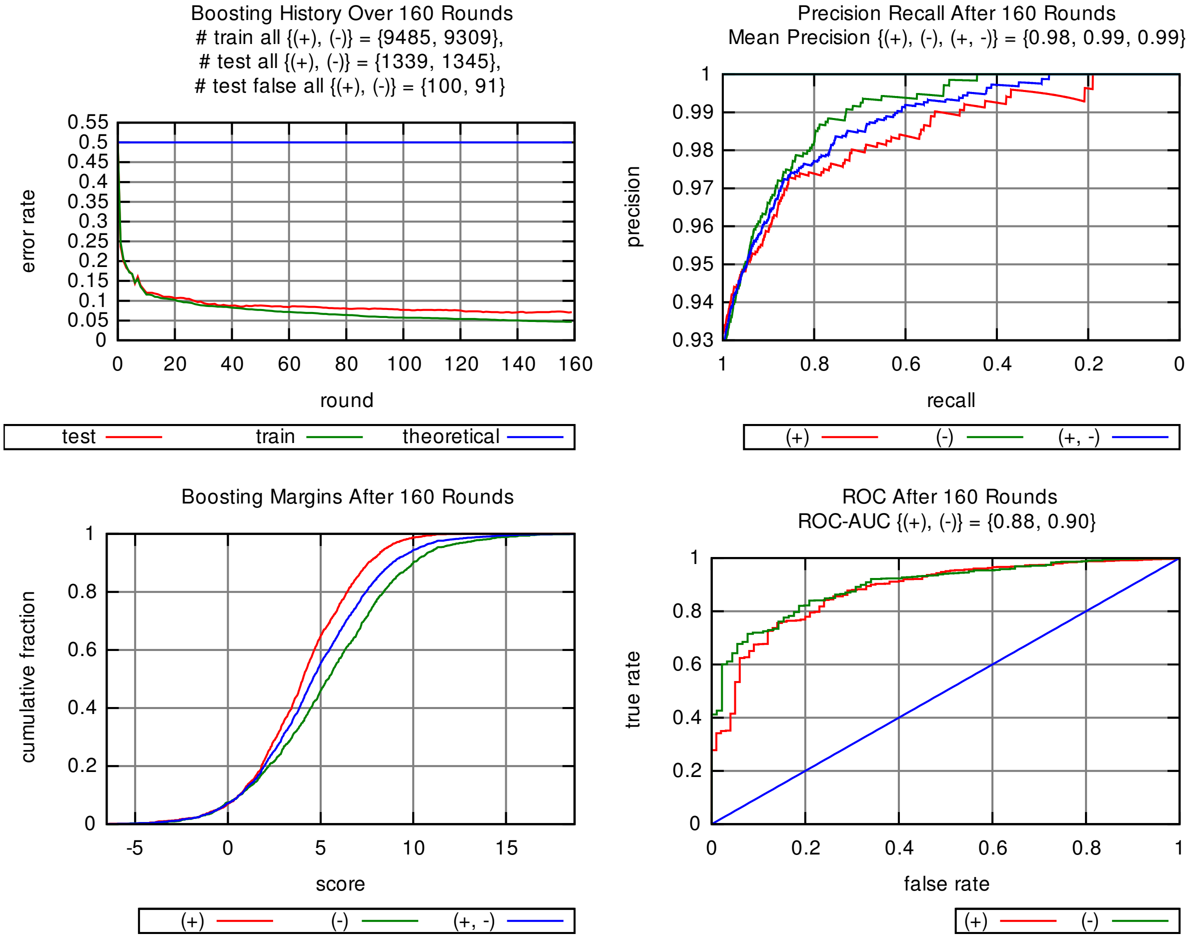

Figure 5. Performance statistics for a single fold. **boosting history)** the test and train set errors as a function of the number of rounds of boosting; **precision-recall)** the precision (accuracy) as a function of recall (the fraction of examples considered); **score margins)** the cumulative distribution function (CDF) of scores. A correct prediction results in a contribution of s to the CDF, where s is the score, while an incorrect prediction results in a contribution of $-s$. Ideally, one would like the CDF to tend to the right and cover no values below 0; **ROC)** an application of ROC analysis as described in the paper. Curves for positives and negatives are shown.

4. Comments on Feature Extraction and Classification

Given a collection of features, the boosting algorithm has the ability to identify and combine a subset of them to create highly accurate classifiers. In light of this property, we engineer features with quantity, rather than quality, in mind – so long as *some* discriminate among crystals and non-crystals, *most* can be of little to no use. The alternating decision tree variant of boosting at the core of our system consists of the two parts implied by its name – guided by boosting rules, the algorithm builds an alternating decision tree incrementally in rounds. The mechanics of how boosting determines a decision rule of the form “feature value $<$ threshold” at each round is beyond the scope of this work, and we refer the reader to a survey [3] and the specific algorithm [4]; however, we do illustrate the mechanics of alternating decision trees in Figure 7-j. Note that these classifiers output real-valued predictions, the signedness of which represent the label and the magnitude of which represent the confidence. Thus, the scores determined for squares and images by the system are, in a sense, a byproduct of the learning process.

4.1 Learned AD Tree

In addition to performance statistics, each cross validation fold also yields an alternating decision tree learned from training examples. The classifier itself is a piece of Java source code; one compiles and incorporates it into the system by following

the installation instructions for customizations in Section 1.2. Recall from the paper that each round of boosting adds a decision node, along with its two scoring node children, to the previous iteration's tree; consequently, 160 rounds of boosting would create a tree of 480 nodes. For simplicity's sake, we present a much simplified tree in Figure 6, and encourage the user to run the cross validator script in Section 1.4 to view trees in their full complexity.

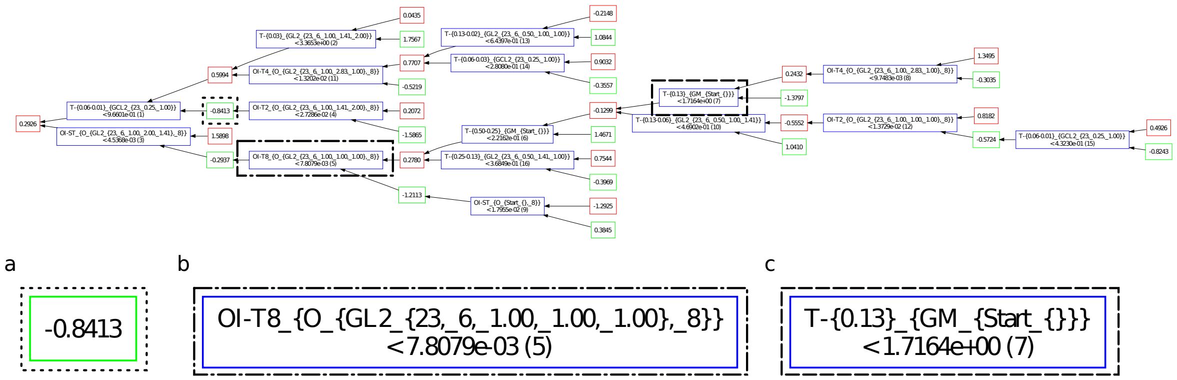

Figure 6. A simplified alternating decision tree. **a)** a scoring node, as described in the paper; **b)** descriptor for a decision node, as described in the paper. This particular node reads "The size of the top 8th bin of the orientation histogram derived from the $(23, 6, 1, 1, 1)$ Gabor feature is less than the threshold value 0.0078"; **c)** descriptor for another decision node. This particular node reads "The 12.5 percentile threshold of the gradient magnitude response on the original image is less than the threshold value 1.7".

5. Preprocessing

As mentioned in the paper, laboratory-specific imaging conditions may introduce troublesome artifacts that unnecessarily confuse classifiers. Although one can, in theory, overcome such issues with an abundance of crystal-negative examples trained on the artifacts along with appropriate feature design, a simpler solution often exists. To keep the user from having to delve into many layers of source code for potentially trivial customizations, we introduce a preprocessor interface that takes as input an image and outputs a collection of square offsets that potentially restrict the algorithm's scanning area.

In the current iteration of the system, any Java class wishing to qualify as a preprocessor simply provides an implementation to the method below.

```
/**
 * Calculates scan offsets.
 *
 * @param m
 *               the intensity image.
 * @param ws
 *               the window size.
 * @param spacing
 *               the scan spacing.
 * @return an array of offsets where the <tt>(row, column)</tt> offsets are given as rows.
 */
public IntegerArray getScanOffsets(RealArray m, int ws, int spacing);
```

That is, a preprocessor is under contract to return an IntegerArray of (row, column) offsets (inducing a scanning subregion) upon being given an intensity image represented as a two-dimensional RealArray. Users may install preprocessors as described in Section 1.2. As a design guideline, classes implementing the interface should be simple and require little to no machine learning. We demonstrate the use of a simple preprocessor to detect beveled well boundaries in Figure 8.

References

[1] Matteo Frigo and Steven Johnson. The design and implementation of FFTW3. *Proceedings of the IEEE*, 93(2):216–231, 2005.

[2] David Patterson. The berkeley view: A new framework and a new platform for parallel research, 2007. Various invited talks.

[3] Robert Schapire. The boosting approach to machine learning: an overview, 2001.

[4] Yoav Freund and Llew Mason. The alternating decision tree learning algorithm. In *ICML '99: Proceedings of the Sixteenth International Conference on Machine Learning*, pages 124–133, San Francisco, CA, USA, 1999. Morgan Kaufmann Publishers Inc.

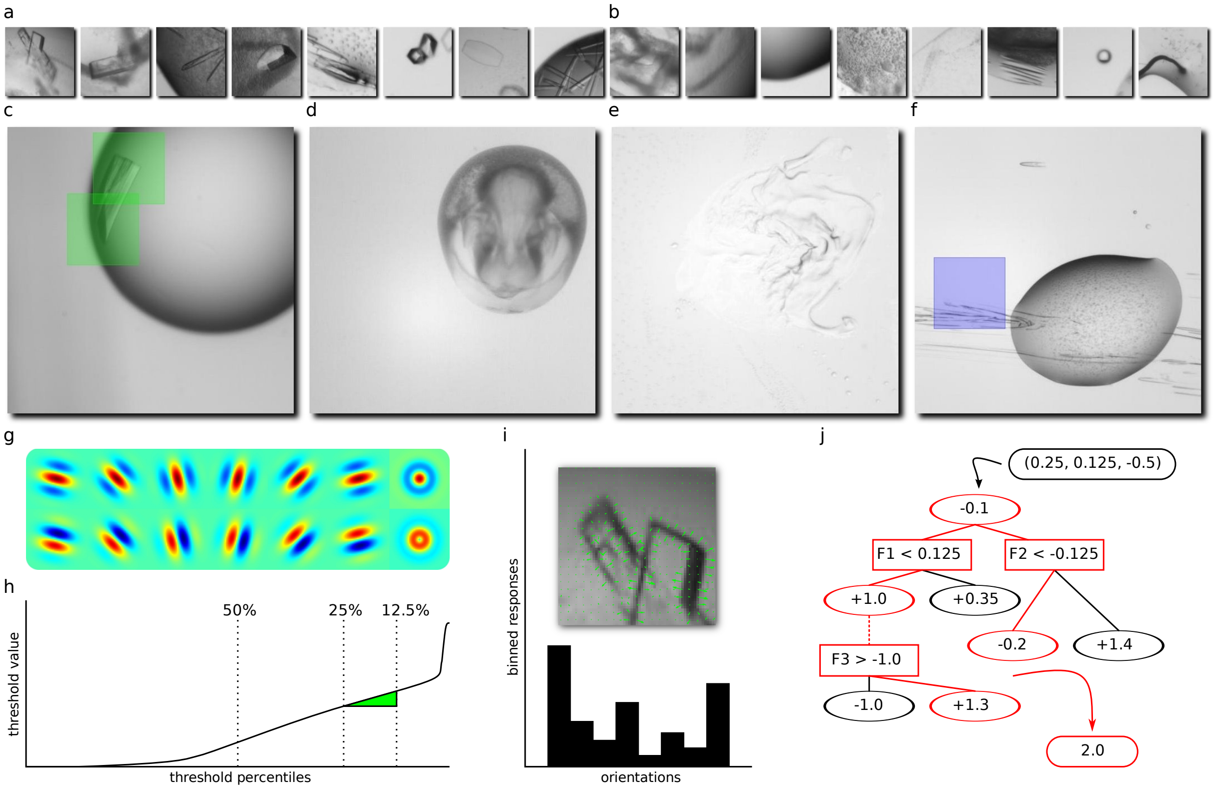

Figure 7. An illustrated guide to crystals, feature extraction, and prediction. **a, b)** putative crystals and non-crystals, as they would seen by the algorithm; **c)** a diffraction candidate overlaid with positive annotations; **d)** cloudy precipitant; **e)** clear; **f)** a false positive annotation made by the algorithm; **g)** Gabor filters. The first six columns depict a complex-valued filter at 6 orientations visualized by real parts on the top row and imaginary parts on the bottom row. The seventh column depicts a non-oriented, complex-valued filter; **h)** calculation of a threshold delta statistic. To compute the 0.250-0.125 statistic, the response values are sorted in increasing order. The "delta" shown in green is the total change in threshold values between the top 25 and 12.5 percentiles; **i)** the orientation histogram of a square. Each pixel's gradient direction is quantized into one of 8 bins, and its gradient magnitude is the contribution to the target bin; **j)** the alternating decision tree prediction procedure. Begin traversal at the root. Upon encountering a circular scoring node, mark it and recurse to all children. Upon encountering a rectangular decision node, take the left branch if its predicate evaluates to false. Otherwise, take the right branch. The resulting prediction is the sum of the values contained in all marked scoring nodes, colored in red. The dashed line depicts how a round of boosting might have attached a decision node along with its two children to the tree.

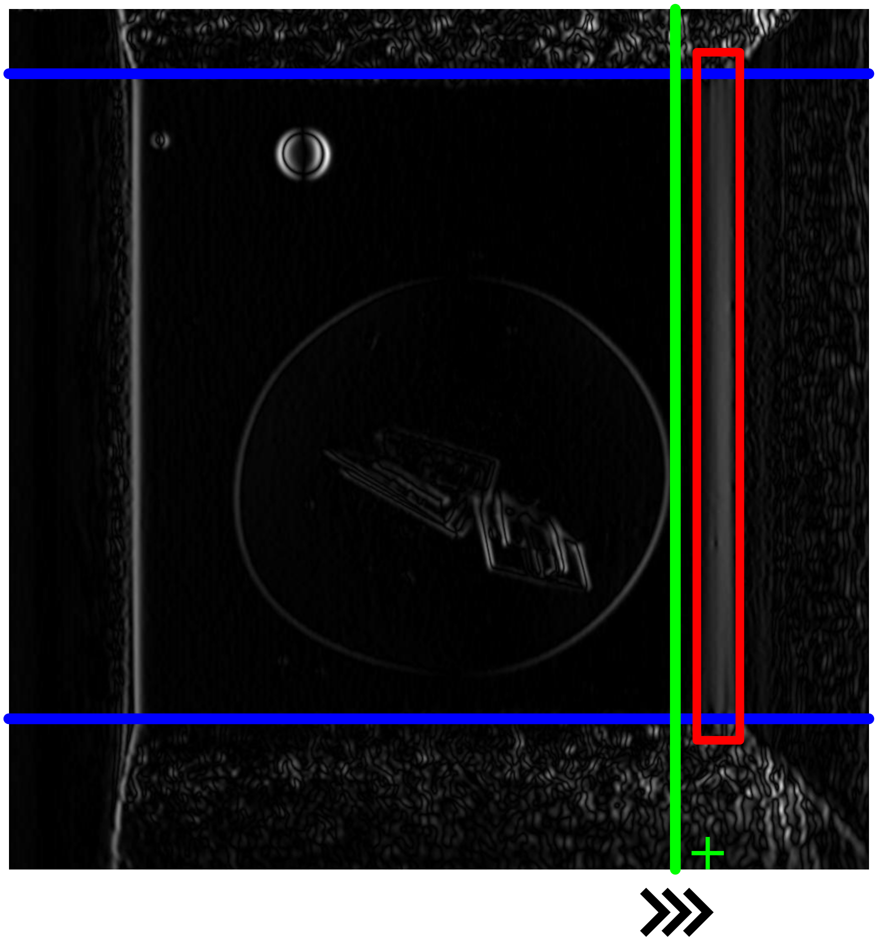

Figure 8. A simple edge detector for determining beveled well boundaries. Suppose that the top and bottom bounds, shown in blue, have already been determined. To find the right and left bounds, we convolve the image with a horizontally oriented edge filter. We then calculate the maximum over the sums of the absolute values of responses along vertical lines, exemplified by the green line. The right well bound discovered by the preprocessor is delimited by the red bounding box.
